# Triazolinedione protein modification: from an overlooked off-target effect to a tryptophan-based bioconjugation strategy†

**DOI:** 10.1039/d1sc06942j

**Published:** 2022-03-15

**Authors:** Klaas W. Decoene, Kamil Unal, An Staes, Olivier Zwaenepoel, Jan Gettemans, Kris Gevaert, Johan M. Winne, Annemieke Madder

**Affiliations:** Department of Organic and Macromolecular Chemistry, Ghent University Krijgslaan 281 S4 9000 Ghent Belgium Annemieke.Madder@UGent.be; Department of Biomolecular Medicine, Ghent University Ghent Belgium; VIB Center for Medical Biotechnology Technologiepark-Zwijnaarde 75 9052 Ghent Belgium; VIB Core Facility, VIB Center for Medical Biotechnology Technologiepark-Zwijnaarde 75 9052 Ghent Belgium

## Abstract

Labelling of tyrosine residues in peptides and proteins has been reported to selectively occur *via* a ‘tyrosine-click’ reaction with triazolinedione reagents (TAD). However, we here demonstrate that TAD reagents are actually not selective for tyrosine and that tryptophan residues are in fact also labelled with these reagents. This off-target labelling remained under the radar as it is challenging to detect these physiologically stable but thermally labile modifications with the commonly used HCD and CID MS/MS techniques. We show that selectivity of tryptophan over tyrosine can be achieved by lowering the pH of the aqueous buffer to effect selective Trp-labelling. Given the low relative abundance of tryptophan compared to tyrosine in natural proteins, this results in a new site-selective bioconjugation method that does not rely on enzymes nor unnatural amino acids and is demonstrated for peptides and recombinant proteins.

## Abstract 2

A new strategy for selective tryptophan modification using triazolinedione (TAD) chemistry at pH 4 is shown on peptides and proteins. Additionally, off-target modification of tryptophan residues during the classical TAD-Y click reaction is uncovered.

## Graphical abstract

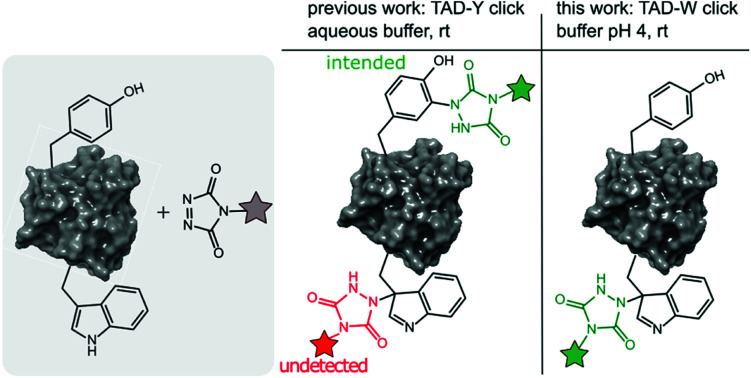

## Introduction

**Scheme 1:**
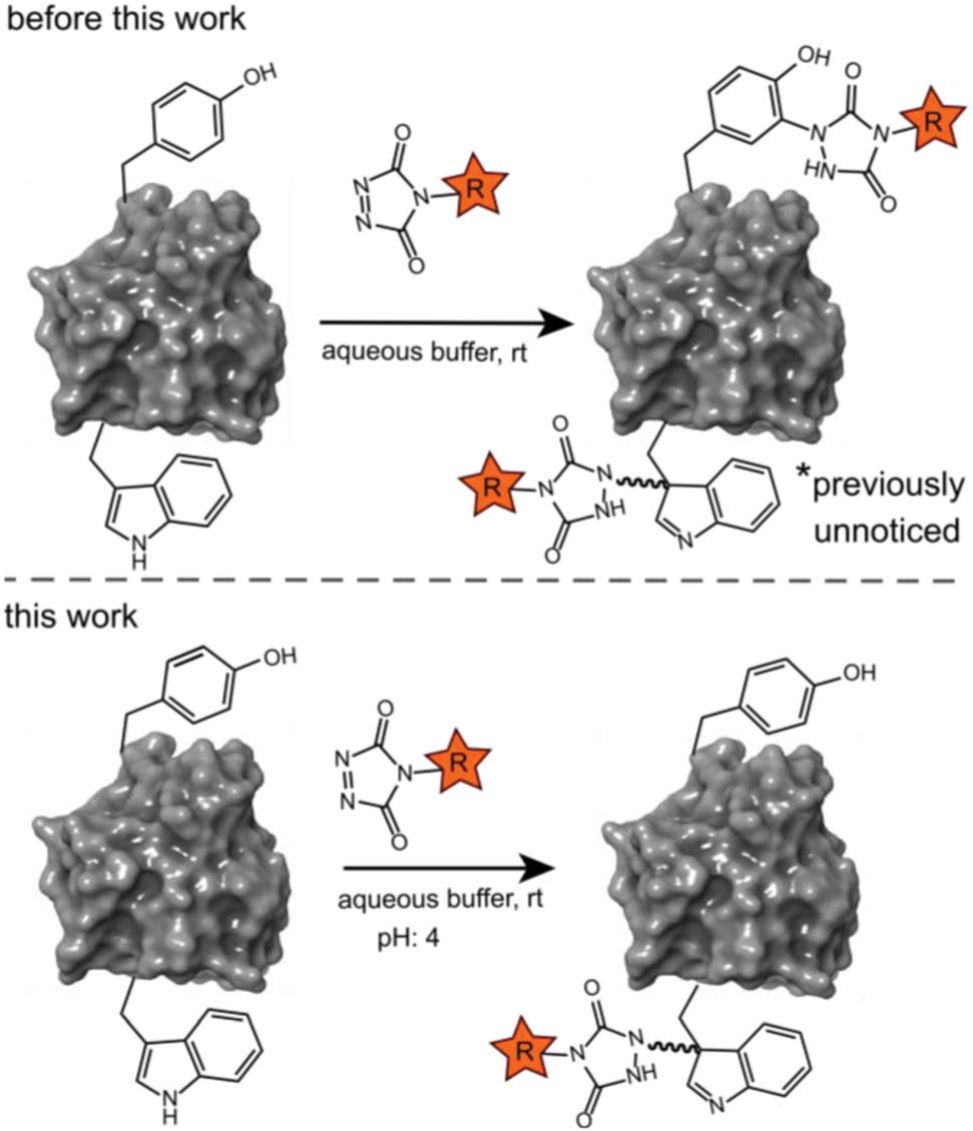
Prototype reaction for the TAD-Y click (previous work) and selective TAD tryptophan labelling (this work).

Site-selective protein modification reactions are highly sought after by researchers in both academia and industry. Site-selectivity is of crucial importance for many applications from fundamental biology (fluorescent tagging) to therapeutic development (antibody–drug conjugates).^1–4^ While amino acid selectivity can be achieved by exploiting the nucleophilic functionalities of *e.g.* lysines and cysteines,^5,6^ genuine site selectivity depends on their representation density on the protein surface. In this regard, tryptophan (Trp) is an interesting target for native conjugation strategies, with an abundance of only just over 1% in proteins.^7^ Despite the indole side chain not being the most chemically tractable target, several groups have reported methodologies for selective modification of tryptophan in peptides and proteins.^8–11^ Many of these strategies employ transition metal catalysed reactions and/or conditions limiting downstream biochemical applications. These reactions are typically alkynylations and C–H arylations of the indole.^12–16^ Also, Trp sulfenylation was demonstrated for peptide ligation.^17^ While Francis and co-workers showed rhodium carbenoid-based Trp labelling at mild pH,^18^ this method is dependent on transition metal catalysis and requires long reaction times. An organoradical Trp conjugation was demonstrated on peptides and proteins^19^ and even if the method is devoid of transition metals, it requires acidic conditions and is not compatible with aqueous buffers. Recently, a novel biomimetic approach for the selective conjugation of tryptophan was developed, the original method however employs UV irradiation and needs to be performed in absence of oxygen.^20^ This approach was further refined and now allows for the use of visible light in presence of ambient air.^21^ In 2010, Barbas and co-workers reported a click like reaction for the more abundant tyrosine (Tyr, 3.3% abundance^7^) using triazolinedione chemistry,^22^ after which many applications and refinements for protein conjugation followed.^23–29^ Interestingly, when exploring this powerful Tyr click reaction on Trp containing peptides, we observed a high degree of off-target labelling on Trp residues, even in aqueous buffers. In the initial paper reporting the development of the TAD tyrosine click like reaction, Barbas and co-workers reported that the TAD reaction is amino acid selective for tyrosine in aqueous buffers. In this work we prove that this is in fact not the case and we demonstrate how competitive Trp-labelling remained under the radar for over a decade (Scheme 1). We show that tryptophan-TAD modifications, while stable at ambient temperature in buffered conditions, can reverse under commonly used HCD and CID MS/MS conditions rendering their detection more tedious. Thus, while often remaining unnoticed, off-target Trp-labelled proteins can be present in labelled samples. Additionally, we show on the peptide, protein and proteome level that by lowering the pH of the buffer, the TAD protein conjugation reaction becomes amino acid selective for tryptophan. These findings constitute a new modification method for tryptophan residues applicable to peptide and protein substrates in buffered solutions at lower pH.

## Results and discussion

### Intermolecular Tyr *vs.* Trp competition for TAD

**Fig. 1:**
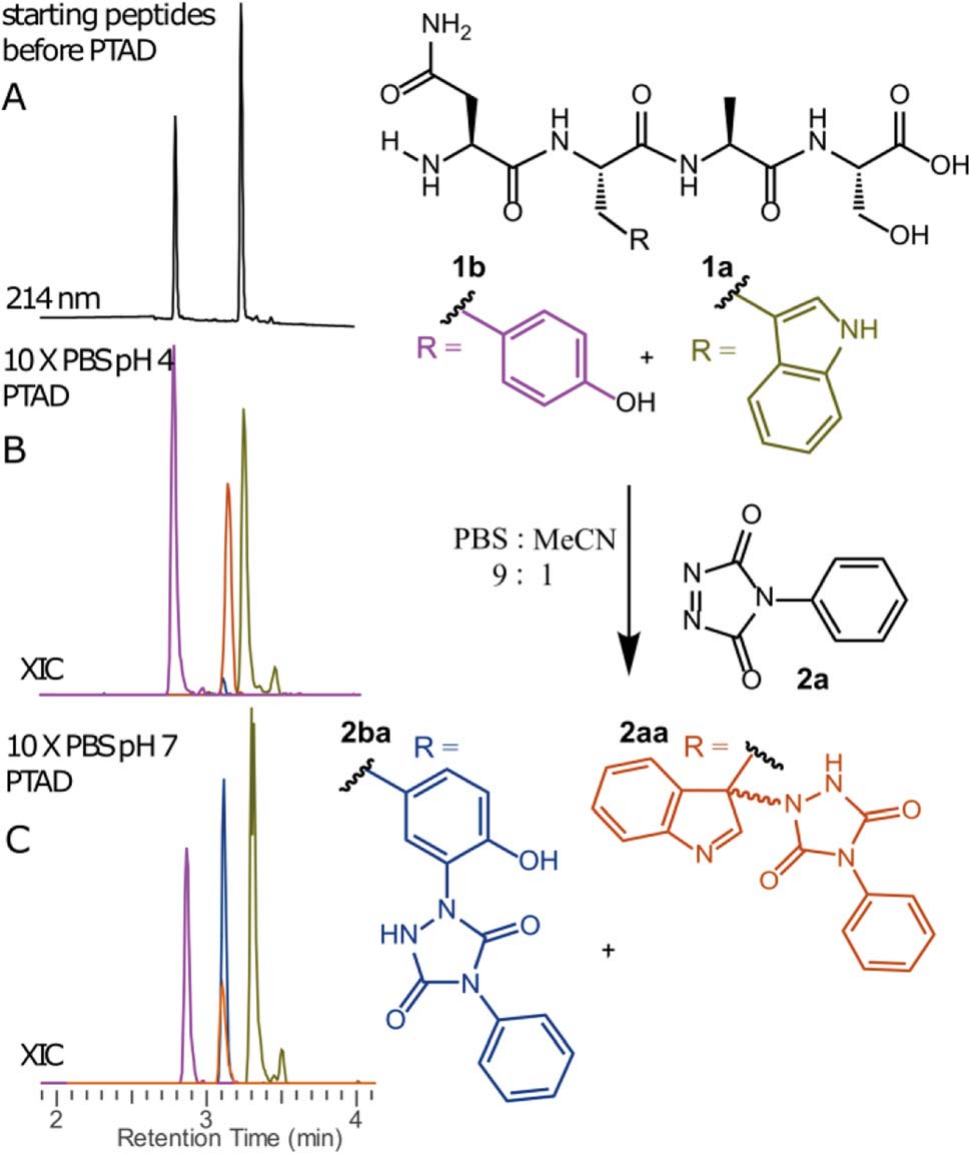
Competition experiment between NWAS (1a, 0.3 mM) and NYAS (1b, 0.3 mM) peptides for PTAD (2a, 0.4 mM) in 10x PBS buffers at pH 4 and pH 7. HPLC chromatogram before reaction (A). Extracted ion chromatograms (XIC) of both starting peptide ions 1a (green) and 1b (pink) and conjugated peptide ions 2aa (orange) and 2ba (blue) for reaction in 10x PBS at pH 4 (B) or pH 7 (C). Based on the area under the curve of the starting peptide XIC chromatograms before and after conjugation, 33% of 1a was converted and only 1% of 1b at pH 4. On the other hand, at pH 7, 35% of 1b and around 11% of 1a was converted.

We decided to more closely examine the competition between Trp and Tyr labelling by TADs in order to probe the potential of TAD reagents for selective Trp-bio-conjugation (Scheme 1). For that purpose, tetrapeptides NWAS 1a and NYAS 1b were tested in intermolecular competition experiments with phenyltriazolinedione (PTAD 2a) in PBS-buffer at two different pH values, allowing for head to head comparison between Tyr and Trp side chains embedded in the exact same chemical environment (Fig. 1). Signals for peptide conjugates 2aa and 2ba overlap on the HPLC UV chromatogram, therefore extracted ion chromatograms (XIC's) were used for the analysis. When analysing the XIC's of the starting peptide-ions NWAS 1a (green) and NYAS 1b (pink) and conjugated peptide-ions NWAS-PTAD 2aa (orange) and NYAS-PTAD 2ba (blue), a pronounced difference can be observed between the reaction at pH 4 and pH 7. Indeed, at pH 4 Trp conjugate 2aa was detected nearly exclusively while at pH 7 a mixture of conjugates was obtained with the Tyr conjugate 2ba as the major product. This observed pH-dependent reactivity of TADs with Tyr is in accord with previous mechanistic studies of the tyrosine-TAD click reaction, which indicate the phenolate species as the prevalent nucleophile, which is more abundantly present at high pH.^30^ Lowering the pH will effectively decrease the amount of tyrosine-phenolate form and thus decrease the extent of reaction of Tyr with TAD. This was further confirmed using additional peptides (1a–1h, Table 1) and TAD-propanol 2b, PTAD-alkyne 2c and fluorescent DMEQ-TAD 2d (ESI Section S2.2.2†). It was also observed that, even without competing Trp-peptide present, lowering of pH causes a significant reduction in Tyr-conjugate formation (ESI Section S2.2.1†). Additionally, the stability of the resulting Trp-TAD conjugates was tested by HPLC analysis of conjugates 2eb, 2ed and 2cd. Conjugate 2eb was left for 2 weeks at room temperature in 10X PBS buffer at pH 7, and stability was checked at several time points through HPLC analysis. Analysis of the chromatograms at 214 nm from these samples demonstrated the 2eb Trp-conjugate signal to remain largely unaltered over prolonged periods (ESI Section S2.2.3†). The stability of fluorescent conjugates 2ed and 2cd was further tested *via* different experiments in PBS buffers with pH values ranging from pH 4 to 9 at room temperature, as well as in 20% human serum at 37 °C. These HPLC analyses show no peak intensity reduction after 24 h in 20% human serum at 37 °C and under 20% reduction after one week at room temperature for all tested pH conditions. These data confirm the Trp conjugate stability under different pH conditions as well as in biologically relevant media at 37 °C. Yield optimisation experiments with tryptophan containing peptide 1e and TAD reagent 2c in PBS at pH 4 demonstrated that 10 equivalents of 2c are sufficient for a conversion of over 90% (ESI Section 2.2.4†).

**Table 1:** Peptide sequences used in this study, structures of TAD reagents 2b, 2c and 2d

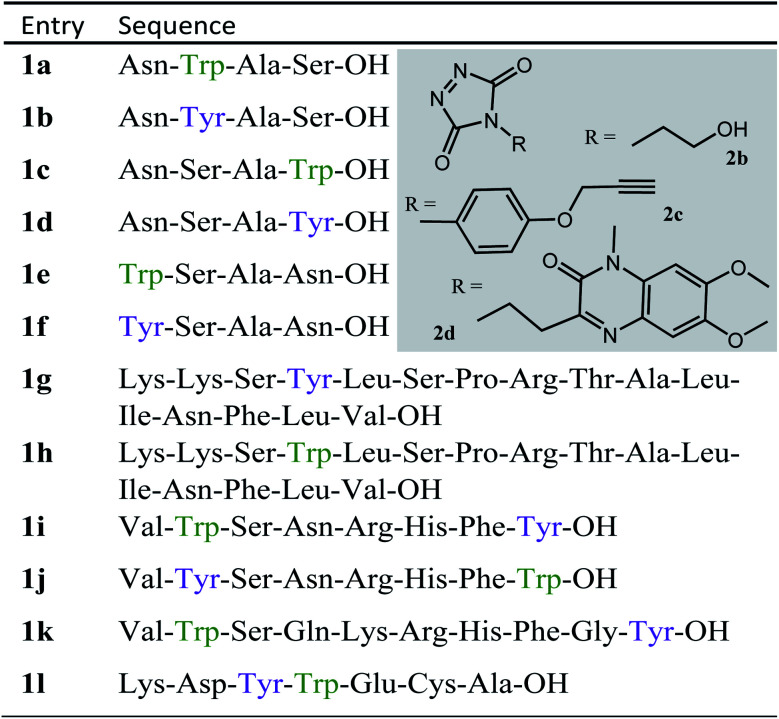

### Influence of the relative Trp position

**Fig. 2:**
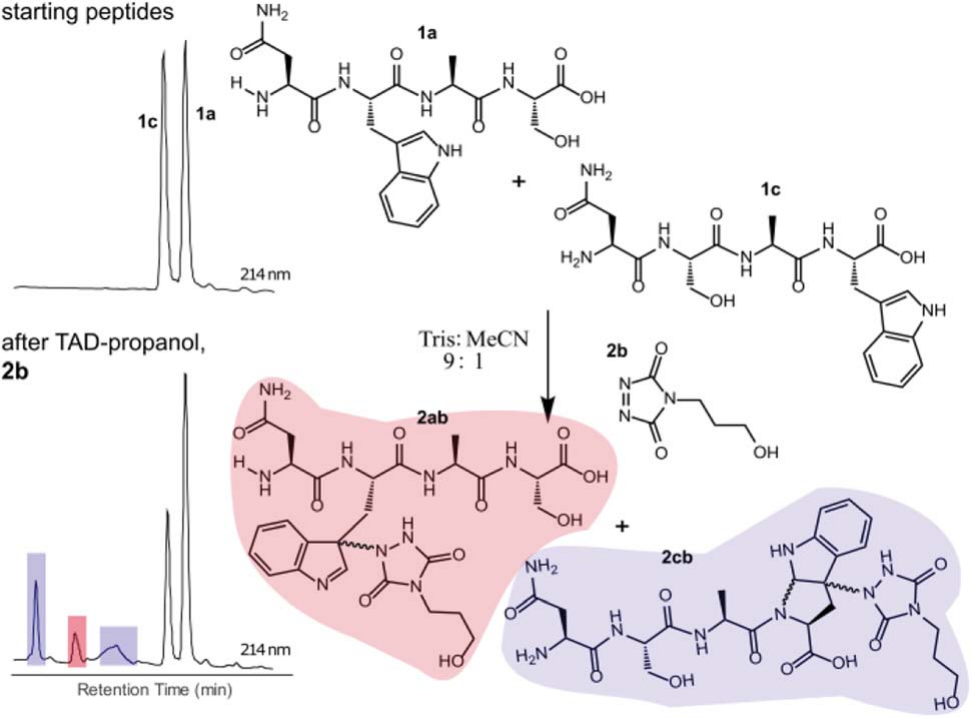
Competition experiment of NWAS (1a, 0.3 mM) and NSAW (1c, 0.3 mM) peptides with TAD-propanol (2b, 0.5 mM) in Tris buffer pH 5.8. Zoom of the HPLC chromatogram at 214 nm of the starting mixture (top left) and after reaction with TAD-propanol 2b (bottom left). Based on peak integration of the starting peptide XIC chromatograms before and after conjugation 25% of 1c was converted while around 8% of 1a was converted.

Triggered by these findings, indicating that a completely Trp-selective modification can be possible, we examined TAD-Trp conjugation in peptides by investigating the influence of the relative amino acid positioning on the outcome of the reaction. Competition experiments between tetrapeptides NWAS 1a and NSAW 1c and TAD-propanol 2b illustrated a remarkable difference in reactivity (Fig. 2). Intermolecular competition between 1a and 1c clearly demonstrates the position-sensitivity of the Trp-TAD reaction: the C-terminal tryptophan in 1c is labelled to a 3 times higher extent compared to its internal tryptophan 1a counterpart. This was calculated *via* HPLC peak integration at 214 nm of the separately eluting 2ab and 2cb products as well as *via* the relative conversion of the starting peptides. This reactivity difference can be attributed to the more exposed reactive centre as well as to the presence of the carboxylic acid which can transiently donate a proton to the TAD moiety rendering it even more electrophilic. A second striking difference resides in the nature of the formed adducts. For the C-terminal tryptophan, two peaks for the labelled product 2cb are observed, indicating the formation of isomers. Indeed, we found this adduct had undergone an additional annulation caused by the reaction of the lone pair on the backbone nitrogen with the indole C2 after reaction of TAD with the indole C3. These findings were confirmed *via* NMR analysis of Boc-Trp-OH and N-Ac-Trp-OMe adducts with TAD-propanol 2b (ESI Section S4†) and are in agreement with the results reported by Baran *et al.*^31^ on non-peptide related TAD-indole reactions. While the reaction of tryptophan with TAD is in theory not influenced by pH, the additional annulation of a C-terminal tryptophan during reaction with TAD might be enhanced at low pH.

### Intramolecular Tyr *vs.* Trp competition for TAD

**Fig. 3:**
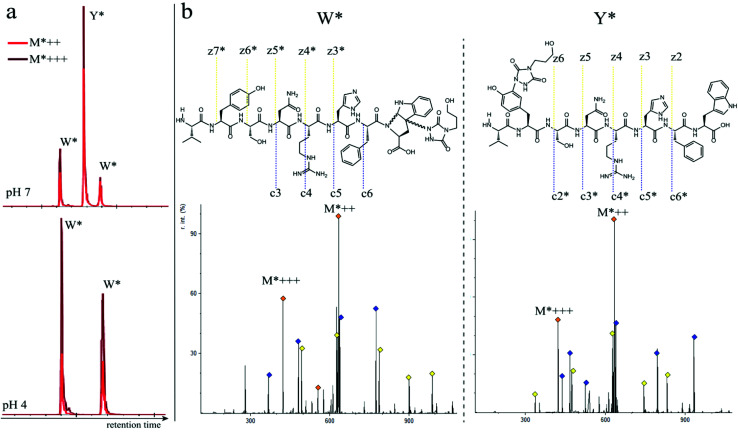
a: Zoom of the ion chromatogram showing the double and triple charged TAD propanol modified peptide ions. The conjugation reaction of peptide 1j with TAD-propanol 2b was performed in 10 X PBS pH 7 (top) or 10X PBS pH 4 (bottom). At pH 7, three peaks with the TAD-modified peptide are detected, two of which correspond to the 2 diastereomers of TAD modification on tryptophan and one peak corresponds to tyrosine modification. At pH 4, only 2 peaks are observed corresponding to the 2 diastereomers of TAD modification on tryptophan. b: ESI ETD MS/MS analysis of VYSNRHFW 1j after modification with TAD-propanol 2b at pH 7, first tryptophan diastereomer peak (left, W*) and tyrosine modified product (right, Y*). Above both MS/MS spectra the chemical structure is depicted with the observed fragment ions. Fragment ions corresponding to TAD-modified peptide fragments are indicated with “*”.

In a subsequent series of experiments, we investigated if the observed intermolecular selectivity, also translates into intramolecular Trp *versus* Tyr selectivity. To this end, competition experiments were performed with peptides containing both tyrosine and tryptophan (1i–1l, Table 1). MS/MS analyses were done to determine the modification site. We found that the modification on tryptophan is unstable in all tested MS/MS conditions except for ESI in combination with electron transfer dissociation (ETD), *vide infra*. ESI-HCD, ESI-CID as well as MALDI-TOF/TOF all largely lead to the loss of the TAD modification on tryptophan (ESI Sections 2.3, 2.6 and 2.7†). The TAD modification on tyrosine was found to be stable in all tested MS/MS conditions. Peptide VYSNRHFW 1j was labelled using TAD-propanol 2b at pH 4 and at pH 7 and analysed *via* ESI-ETD MS/MS (Fig. 3). In Fig. 3a the ion chromatograms of the double and triple charged TAD modified peptide ions are shown for reaction at pH 7 (top) and pH 4 (bottom). Analysis of the ion chromatograms in Fig. 3a (full ion chromatograms ESI Section 2.4.1†) shows that at pH 7 three peaks are visible, the main product peak is accompanied by two smaller peaks. On the other hand, at pH 4 only two peaks are observed with the same elution profile as the two smaller peaks of the pH 7 experiment. Note that regardless of the site of the modification (Y or W), the modified peptide mass will be the same but the exact location of the modification is determined *via* ETD MS/MS analysis of the modified peptide ion in each peak of the ion chromatogram (Fig. 3b).

The triply charged precursor (M*+++: 422.53) was selected for electron transfer dissociation (ETD) MS/MS analysis. In Fig. 3b the ETD MS/MS spectra are compared for the M*+++ ion in the smaller peaks (left) and the main product peak (right) of the 1j modification experiment with 2b at pH 7. The ETD MS/MS analysis confirms the location of the TAD modification in the main product on tyrosine and for both accompanying peaks the TAD modification is unambiguously assigned to tryptophan. This confirms the presence of a tryptophan-TAD (W*) modified peptide next to the tyrosine-TAD modified product (Y*) at pH 7. Additionally, ETD analysis of conjugation experiments performed at pH 4 demonstrate selective Trp-TAD modification (ESI Sections 2.4.1 and 2.5†). Note that tryptophan modification at pH 7 becomes less pronounced, but not blocked, in peptides where the competing tryptophan is not at a C-terminal position (compare Fig. 3 for peptide 1j with Fig. 2.4.1.4 for peptide 1k in ESI†). The reactivity of the tryptophan side chain, and the degree to which it will compete with tyrosine, thus varies with its availability and position in the peptide chain. Further confirmation for the off-target labelling at pH 7 is provided through MS based screening for doubly modified peptides (ESI Fig. 2.4.2.3†) showing double TAD modified peptides only at pH 7, implying additional off-target Trp labelling next to the intended Tyr labelling.

### Proteome-wide selectivity of TADs

**Fig. 4:**
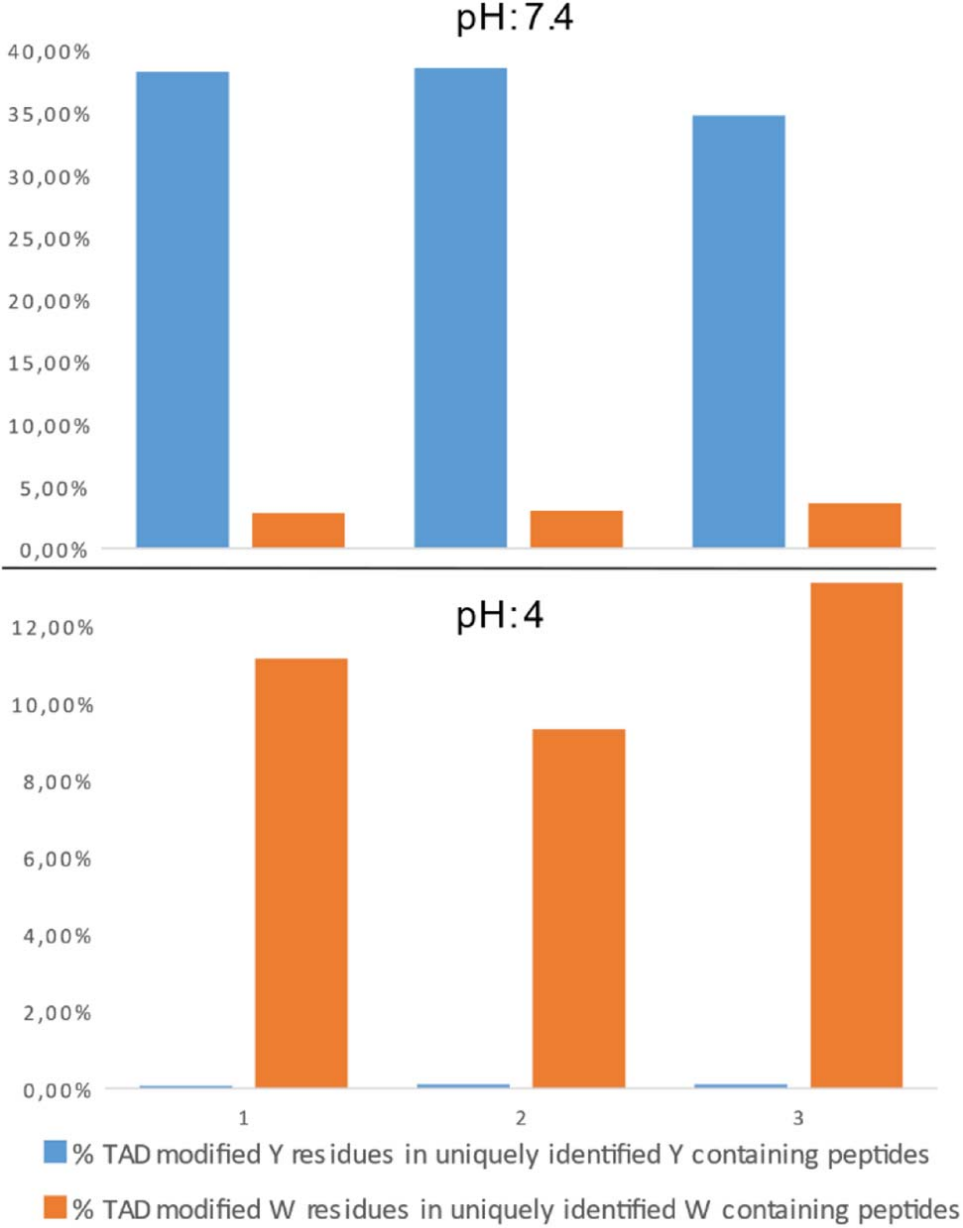
Proteome wide pH analysis: bar graph representation of the percentage of TAD modified peptides in uniquely identified Y/W containing peptides. The modification reaction was performed at pH 7.4 (top panel) or pH 4 (bottom panel). Triplicate experiments were performed for each pH setting.

In a recent report on ChemRxiv, the proteome-wide selectivity of diverse electrophiles, including a TAD reagent (PTAD-alkyne, 2c) was profiled.^29^ The authors report a very elegant approach to interrogate the amino acid selectivity of a wide range of electrophiles. But as they indicate in their report, potential MS/MS lability of modified amino acids during HCD-based peptide fragmentation was not included in their analysis. This entails that the TAD-tryptophan modification can be missed in such studies. We performed a proteome-wide selectivity study with PTAD alkyne 2c reacting with a tryptic digest of a HeLa cellular proteome in PBS buffer at pH 4 or pH 7.2 in triplicate (ESI Section 2.8†). To enable detection of TAD-tryptophan modifications, we included the possibility of neutral loss of the TAD modification on tryptophan residues in the search parameters. On peptides modified at pH 7.2, we found that TAD reagents indeed exhibit a high selectivity for tyrosine over tryptophan residues with around 37% of the tyrosine and over 3% of the tryptophan residues in uniquely identified peptides modified with TAD. On the other hand however, reversed selectivity is observed when peptides were modified at pH 4, only 0.09% of the observed tyrosine and over 11% of the tryptophan residues in uniquely identified peptides were modified (Fig. 4).

### TAD modification of recombinant proteins

We next explored TAD-click reactions for Trp-based protein conjugation. Alphabodies have a triple helical coiled coil structure and are developed for intracellular protein interaction targets by Complix N.V.^32^ Two recombinant alphabodies were used; the valentine alphabody containing no tryptophans and three tyrosines, and alphabody 586D containing one tryptophan residue next to three tyrosines. A competition experiment between the valentine alphabody and tryptophan containing peptide 1c (ESI Section 3.2†) with 2b at pH 4, resulted in 80% conversion of 1c while alphabody conjugation was absent. Protein conjugation with 586D at pH 4 was carried out using fluorescent DMEQ-TAD, 2d. MS/MS analysis of the resulting protein conjugate digest (ESI Section 3.3†) confirms the localization of the TAD modification on tryptophan. Intact protein analysis of the 586D 2d conjugate (30 eq., pH 4) shows 60% conjugation. Together, these results indicate that protein TAD modification can be targeted selectively to a Trp side chain, in the presence of tyrosines. As a negative control the valentine alphabody was reacted with 48 eq. of 2b at pH 4 and no conjugation was observed. Additionally, the stability of the alphabody DMEQ-TAD conjugate 2d was investigated by HPLC analysis at 370 nm (DMEQ-TAD absorption). HPLC signal integration of samples did not show any sign of reduction after 24 hours at room temperature in 10X PBS pH 7 thus confirming the Trp-conjugate stability also at protein level.

**Fig. 5:**
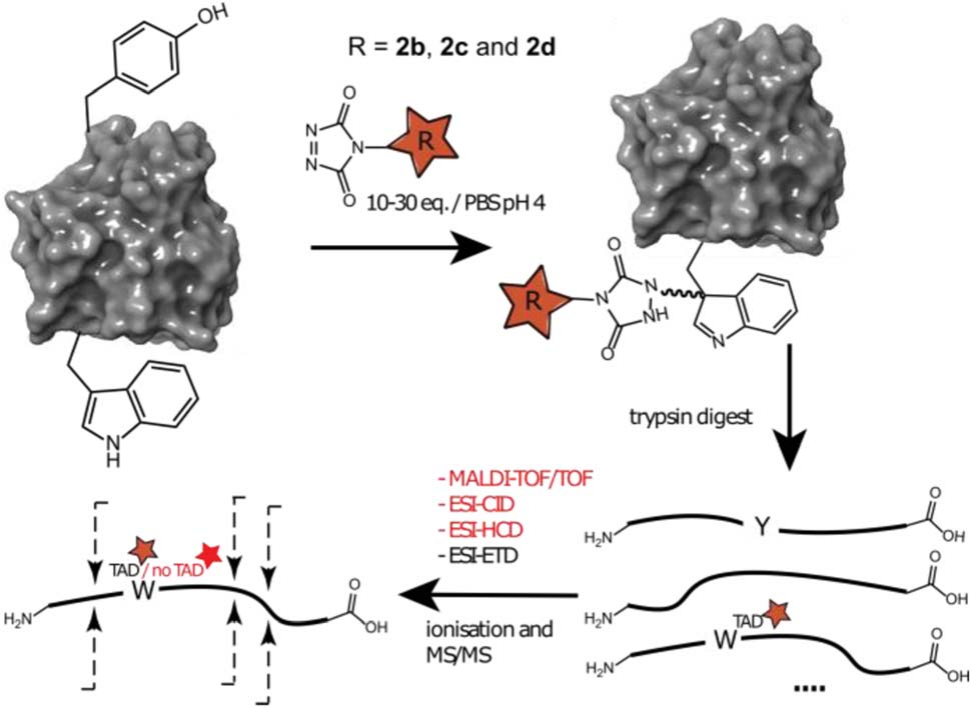
Schematic representation of protein conjugation reaction of alphabody 586D and galectin-7 with 2b, 2c and 2d, subsequent trypsin digest followed by different ionization and MS/MS fragmentation of the modified tryptic fragment. Depending on the applied technique the TAD modification on W can be observed or not.

Additionally, human galectin-7,^33^ containing one tryptophan and one tyrosine residue was treated with 2b, 2c and 2d at pH 4. The conjugated proteins were observed for all TAD reagents. Intact protein analysis of the galectin-7 2d conjugate (10 eq. pH 4, ESI Section 3.4†) shows over 50% conjugation. Analyses of the galectin-7 TAD propanol conjugate digests confirm the localization of the TAD moiety on tryptophan (Fig. 5). Furthermore, the MS/MS analyses of conjugation experiments with 2b (20 eq.; pH 4 and 7) demonstrate that at pH 4 the tryptophan has almost exclusively reacted with TAD while at pH 7 both the tyrosine and the tryptophan had reacted. Additionally, in accord with the findings on the peptide level we found the TAD modification on tryptophan to be labile under the HCD MS/MS conditions used in these experiments.

**Fig. 6:**
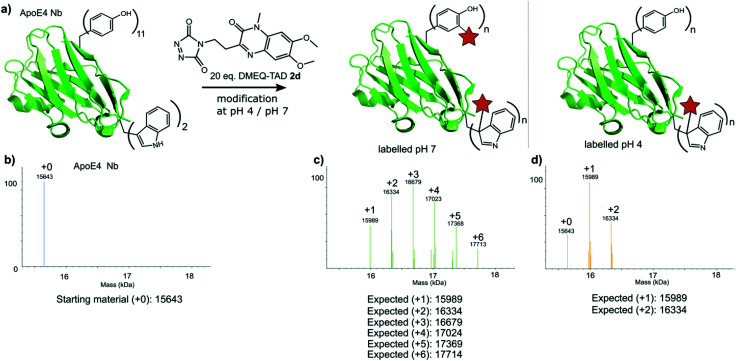
a) Schematic representation of the modification reaction of the ApoE4 Nb with 20 eq. DMEQ-TAD either at pH 4 or pH 7. (b) Deconvoluted mass spectrum of the ApoE4 Nb control sample (no DMEQ-TAD added). (c) Deconvoluted mass spectrum of the ApoE4 Nb labelled with 20 eq. DMEQ-TAD at pH 7. (d) Deconvoluted mass spectrum of the ApoE4 Nb labelled with 20 eq. DMEQ-TAD at pH 4.

Finally, a nanobody against apolipoprotein E4 (apoE4), the most prevalent risk factor of sporadic Alzheimer's disease,^34^ was included in this study. The apoE4 nanobody contains two tryptophan moieties and was labelled with 20 eq. fluorescent DMEQ-TAD 2d in PBS at pH 4. Intact mass analysis of the nanobody conjugate demonstrated 86% modification, 58% single modification and 28% double modification (Fig. 6 and ESI Section 3.5†). After the modification reaction, the binding affinity of the labelled apoE4 nanobody for its antigen was measured and a *K*_D_ value of 8.74 × 10^−9^ M for the apoE4 target protein was measured. The *K*_D_ of the not labelled control nanobody was 5.07 × 10^−9^ M. The same labelling reaction, but now at pH 7 yields a more heterogeneous mixture with the apoE4 nanobody modified from 1 up to 6 times. The *K*_D_ value for this labelled nanobody was found to be 1.02 × 10^−8^ M and thus slightly higher than when labelled at pH 4 (ESI Section 3.5†). These data show that the apoE4 nanobody is able to withstand the labelling reaction at pH 4 and retain a *K*_D_ value in the low nanomolar range. During protein modification experiments we observed a certain degree of methionine oxidation. We found that purging the buffer solution with Argon reduces this substantially. On the other hand use of the reported electrochemical oxidation method for TAD reagents^25,35^ allows avoiding this side effect.

### TAD protein modification in literature

These findings prompted us to look in more detail to earlier reports on the tyrosine click protein modification, and especially how an important off-target effect was able to remain unnoticed for over a decade. Careful reinterpretation of the MALDI-TOF MS spectra obtained from a BSA sample labelled with butyl-TAD followed by a protein digest (kindly provided by the authors of Vandewalle *et al.*^24^) was performed. Indeed, when taking the Trp-TAD modification into account the data shows that besides the reported Tyr-TAD modifications, also a Trp-TAD modification was abundantly present in the sample (ESI Section S3.1). Furthermore, in the initial study of Ban *et al.* TAD-modification of a peptide, crucially containing tryptophans and no tyrosine residues, was observed upon myoglobin labelling. In a study by Hu *et al.* A small amount of modification on tryptophan was observed even in the MS/MS analysis of a TAD modified CRM_195_ protein.^26^ Since the TAD modification on tryptophan is largely lost during most MS/MS analysis methods, this was likely originating from a richly tryptophan-modified peptide causing enough traces of the TAD-tryptophan modification to survive MS/MS fragmentation to allow detection. These findings demonstrate that researchers may miss TAD-tryptophan modifications in the analysis of TAD protein modifications.

## Conclusions

We report that competitive tryptophan labelling is liable to have so far been systematically over-looked in the current use of triazolinedione (TAD) chemistry for putative tyrosine-selective protein conjugation, a technique which is growing in popularity. The reversibility of the TAD-tryptophan adducts under MS/MS analysis conditions, in combination with the low abundance and low accessibility of tryptophan side chains likely caused this off-target effect to have remained under the radar. We have found that an exposed tryptophan is in fact kinetically favoured over tyrosine in certain conditions. Lowering the buffer pH further enhanced the selectivity resulting in a transition metal free, buffer-compatible selective labelling method for tryptophan. Thus, in addition to a better understanding of the factors that govern the click-like TAD-based protein conjugation, its scope has been expanded for peptide and protein substrates. The implementation of Trp-substitutions at protein surfaces or loops can thus be an interesting rational design strategy for fully site-selective labelling of native proteins, given they can withstand treatment at pH 4.

## Data availability

Proteomic data are available *via* ProteomeXchange with identifier PXD031607.

## Author contributions

The manuscript was written through contributions of all authors. All authors have given approval to the final version of the manuscript.

## Conflicts of interest

There are no conflicts to declare.

## Supplementary Material

SC-013-D1SC06942J-s001
