# Supplementary material for: Triazolinedione protein modification: from an overlooked off-target effect to a tryptophan-based bioconjugation strategy
Source: Chem Sci. 2022 Mar 15;13(18):5390–7. doi: 10.1039/d1sc06942j (PMC9093138; doi:10.1039/d1sc06942j)
Supplement: SC-013-D1SC06942J-s001 [file SC-013-D1SC06942J-s001.pdf]

Electronic Supplementary Material (ESI) for Chemical Science.
This journal is © The Royal Society of Chemistry 2022

Triazolinedione protein modification: from an overlooked off-target effect to a tryptophan-based bioconjugation strategy

Klaas W. Decoene,[1,2,3] Kamil Unal,[1] An Staes,[3,4] Kris Gevaert,[2,3] Johan M. Winne,[1] Annemieke Madder[1*]

[1] Department of Organic and Macromolecular Chemistry, Faculty of Sciences, Ghent University, Krijgslaan 281 S4, 9000 Ghent, Belgium

[2] Department of Bio-molecular Medicine, Ghent University, Ghent, Belgium

[3] VIB Center for Medical Biotechnology, Technologiepark-Zwijnaarde 75, 9052 Ghent, Belgium

[4] VIB core facility , VIB Center for Medical Biotechnology, Technologiepark-Zwijnaarde 75, 9052 Ghent, Belgium

Supporting information

Table of Contents

1. General experimental procedures 3
1.1 Solid phase peptide synthesis (SPPS) 3
1.2 Preparation of TAD-compounds 5
1.3 Peptide TAD modification 6
2. Analytical data: peptide conjugation 7
2.1 Characterization of starting peptides 7
2.2 LC (MS) data of peptide-TAD conjugates 8
2.2.1 pH dependency of the reaction between tyrosine and TAD 8
2.2.2 Intermolecular Tyr versus Trp competition 10
2.2.3 Peptide conjugate stability 29
2.2.4 Peptide conjugate yield 33
2.3 MALDI TOF/TOF data of peptide conjugates 34
2.3.1 pH dependency of the intramolecular Tyr vs Trp competition: experimental and software setup 34
2.3.2 MS/MS spectra of unmodified peptides 37
2.3.3 MS/MS spectra of modified peptides 41
2.3.4 MS/MS tryptophan-TAD fragmentation patterns 70
2.4 LC (ESI) MS Fusion Lumos 72
2.4.1 VWSQKRHFGY peptide **1k** with TAD-propanol **2b** 72
2.4.2 VWSNRHFY peptide **1i** and VYSNRHFW peptide **1j** with TAD-propanol **2b**. 77
2.5 LC (ESI) ETD MS/MS 80
2.6 LC (ESI) CID MS/MS 85
2.7 LC (ESI) HCD MS/MS 88
2.8 pH dependent proteome wide tyrosine versus tryptophan selectivity 90
3. Protein conjugation 93
3.1 Bovine serum albumin (reinterpretation of data from Vandewalle *et al.*) 93
3.2 Valentine alphabody 96
3.3 Alphabody 586D 101
3.4 Human galectin-7 109
3.5 ApoE4 nanobody 117
4. Small molecule Trp-TAD conjugation and NMR structural analyses of the obtained adducts 122
4.1 Boc-Trp-OH 122
4.2 N-Acyl-Trp-OMe 125
5. NMR spectra of compounds 128
6. References 136

1. General experimental procedures

1.1 Solid phase peptide synthesis (SPPS)

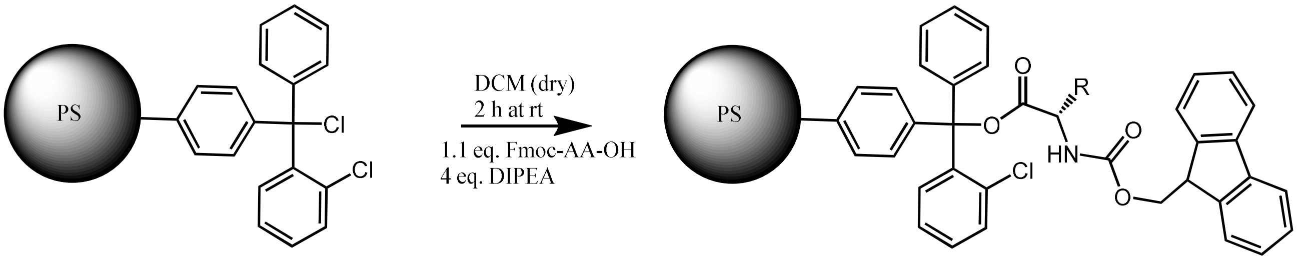

Scheme S1.1.1 Manual loading of the first Fmoc protected amino acid onto the 2-chlorotrityl chloride linker.

Peptides were synthesized using standard Fmoc-chemistry on polystyrene resin with 2-chlorotrityl chloride linker, loading 1.57 mmol/g. The resin was swollen for 20 min in dry DCM, after which the solvent was filtered off. The first (C-terminal) amino acid was coupled manually using 4 equivalents of N,N diisopropylethylamine and 1.1 equivalents of the first (C-terminal) Fmoc protected amino acid in dry DCM (scheme S1.1.1). The reaction mixture was added to resin and shaken for 2 hours at room temperature. Afterwards the reaction mixture is filtered off and the resin is washed with DCM (3x). Unreacted linker functionalities are capped using a DCM/MeOH/DIPEA (17/2/1) mixture, 30 min shaking at room temperature. Subsequently the capping mixture was filtered off and the resin was washed with DCM (3x) and DMF (3x). The remaining amino acids are incorporated using automated peptide synthesis performed on either a MultiPep (Intavis, Tübingen, Germany) or Syro (Multisyntech, Witten, Germany) device. Every cycle in the automated peptide synthesis starts with the Fmoc deprotection of the Fmoc-protected current N-terminus followed by coupling the next Fmoc-protected amino acid. Fmoc deprotection was carried out with piperidine (40%) in DMF, 3 minutes with 40% piperidine in DMF followed by 12 minutes with 20% piperidine in DMF. Synthesis with double couplings was performed with 5 equivalents HBTU (0.5 M) as coupling reagent, 5 equivalents amino acid (0.5 M) and 10 equivalents DIPEA in NMP (2M) as base. Coupling time was 40 minutes. For longer peptides an extra capping step was performed after the double coupling steps of each amino acid. A solution of DMF/acetic anhydride/N-methylmorpholine (90/5/5) was used for capping, 5 minutes reaction time. A mixture of 95% trifluoro acetic acid (TFA), 2.5% tri-isopropyl silane, and 2.5% H_2O was used to cleave the peptide from the resin, 3-hour reaction time. Peptides were subsequently precipitated in cold methyl tert-butyl ether (2x). Purification of the synthesized peptides was done on a semiprep HPLC instrument equipped with a Phenomenex Luna C18 column at 35 °C with a flow rate of 16 mL/min. The column was eluted with a gradient starting at 100% H_2O containing 0.1% TFA to 100% acetonitrile in 20 min. All materials were obtained via commercial suppliers (Sigma Aldrich, Iris Biotech GmbH, Novabiochem, Acros, Fujifilm Wako chemicals) unless otherwise stated. LC-MS analysis was done on a Agilent LCMS system. MALDI TOF and MALDI TOF/TOF spectra were obtained on a 4800 MALDI TOF/TOF instrument (Applied Biosystems).

300 fmol of synthetic peptide was injected for LC-MS/MS analysis on an Ultimate 3000 RSLCnano system in-line connected to a Fusion Lumos mass spectrometer (Thermo). Trapping was performed at 10µl/min for 4 min in loading solvent A (0.1% TFA 98/2 H_2O/ACN) on a trapping column (made in-house, 100 µm I.D. x 20 mm, 5µm beads, C18 Reprosil-HD, Dr.Maisch,

Germany). The peptides were eluted from the trapping column and further separated on a 200cm µPAC™ column (C18-endcapped functionality, 300 µm wide channels, 5µm porous-shell pillars, inter pillar distance of 2.5 µm and a depth of 20 µm, Pharmafluidics, Belgium). Peptides were eluted by a gradient starting at 1% solvent B (0.1% FA in H_2O/ACN 80/20), reaching 9% solvent B in 15 min, 33% solvent B after 30 min, 55% solvent B after 35 min and eventually reaching 99% solvent B after 38 min followed by a 10 min wash and re-equilibration with 99% solvent A (0.1% FA in H_2O). The flow rate was increased to 750 nl/min for the first 15 min, after which it was decreased to 300nl/min and kept constant. The column temperature is set to a constant value of 50°C.

The mass spectrometer was operated in data-dependent mode, automatically switching between MS and MS/MS acquisition in TopSpeed mode using cycle time of 3s. Full-scan MS spectra (300-2000 m/z) were acquired at a resolution of 60,000 in the Orbitrap analyzer after accumulation to a target AGC value of 400,000 with a maximum injection time of 50 ms. The precursor ions were filtered for charge states (2-7 required), MIPS set to peptide and a minimum intensity of 5E4. The precursor ions were selected in the ion routing multipole with an isolation window of 1.6 Da and accumulated to an AGC target of 1E4 or a maximum injection time of 35 ms. Three different activation types were applied for each selected precursor. A first activation was done using HCD fragmentation (NCE 34%). A second parallel activation was done using CID fragmentation (35% CE) with an activation time of 10 ms. A third parallel activation was done by ETD fragmentation using the calibrated charge dependent ETD parameters combining 3 microscans. Fragments of the different fragmentation modes were each analyzed in the ion trap analyzer at normal scan rate.

1.2 Preparation of TAD-compounds

PTAD, **2a**

Synthesized as described elsewhere.[1]

Urazole precursor: TAD-propanol **2b**

5 g diphenyl carbonate (1 eq., 0.023 mol) was melted in a 250 mL flask and 5 g ethyl carbazate (2 eq., 0.047 mol) was added. The mixture stirred under inert atmosphere for 1 h at 90 °C. 2 ml 3-Amino-1-propanol (1,1 eq., 0,026 mol) was added into this molten mixture and stirred for additional 15 minutes without removing oil bath. Subsequently, the product was precipitated with addition of 150 ml ethyl acetate to bulk mixture while it was cooling down. 4,61 g (97%) pure white powder was obtained via filtration and dried in overnight under vacuum

1H-NMR (300MHz, DMSO-d6): δ (ppm) = 1.17 (t, 3H, O-CH2-CH3), 1.52 (quint, 2H, CH2-CH2-CH2), 3.05 (q, 2H, CH2-NH), 3.38 (q, 2H, HO-CH2), 4.03 (q, 2H, O-CH2-CH3), 4.41 (t, 1H, HO-CH2), 6.31 (s, 1H, CH2-NH),7.68 (s, 1H, O-CO-NH), 8.75 (s, 1H, NH-NH-CO-NH).

2 g of the semicarbazide (1 eq., 0,0097 mol), 60 ml ethanol and 6,80 g potassium carbonate (5 eq., 0,0487 mol) were added into 250 ml round bottom flask and the mixture was refluxed overnight. he reaction mixture was cooled to room temperature and acidified to pH 1 with HCl in propanol (5-6 N). The precipitate was filtered off, the reaction mixture was concentrated in vacuo. 1,55 g (95%) propanol urazole was obtained as pure white powder and dried in vacuum oven.

1H-NMR (300MHz, DMSO-d6): δ (ppm) = 1.67 (quint, 2H, CH2-CH2-CH2), 3.40 (m, 4H, CH2-CH2-CH2), 10.02 (s, 2H, NH-NH).

Urazole precursor: PTAD-alkyne **2c**

Purchased from Sigma-Aldrich and used without further purification.

DMEQ-TAD, **2d**

Purchased from Fujifilm Wako Chemicals and used without further purification. DMEQ-TAD is received in the TAD form and does not require oxidation.

Trichloroisocyanuric acid oxidation

Urazole precursors of TAD-propanol **2b** and PTAD-alkyne **2c** are oxidized using trichloroisocyanuric acid (TCCA). To this end 0.33 equivalents of trichloroisocyanuric acid is added to a slight excess: 1.x equivalents of the urazole precursor in acetonitrile, the reaction mixture is shaken for 2 hours at room temperature. Upon the addition of TCCA to the urazole MeCN suspension the mixture turns pink (TAD-propanol, **2b**) or red (PTAD-alkyne, **2c**). After completion the reaction mixture is centrifuged shortly to spin down all insoluble compounds. Afterwards the desired TAD derivative solution in MeCN is pipetted directly out of the supernatant. The TAD derivative is used without further purification.

It is worth noting that there can be trace amounts of HCl left in the TAD solution. This is due to oxidation with trichloroisocyanuric acid. This causes a lower effective pH value in peptide-TAD conjugation samples using no or weak buffers. This effect is minimal in 10x PBS buffers or 100 mM Tris-HCl buffers.

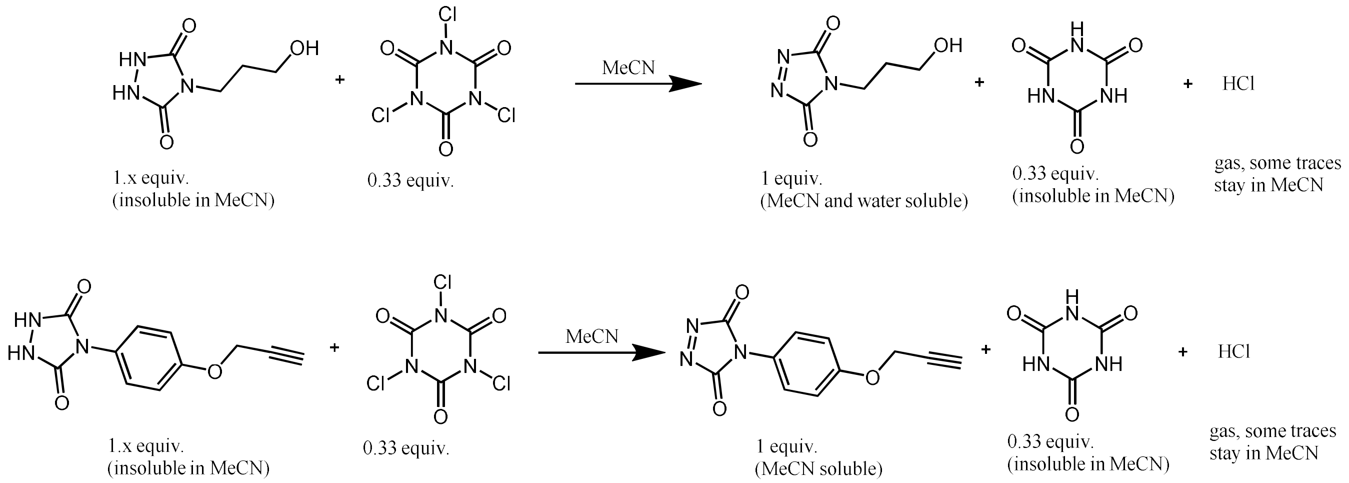

Figure S1.2.1 Synthesis of TAD-propanol **2b** and PTAD-alkyne **2c**.

1.3 Peptide TAD modification

The purified peptides were dissolved in milliQ H_2O to make stock solutions of 3 mM. Typically, 4 mM TAD solutions are prepared in MeCN. 2 µL (6 nmol) of the peptide stock solution is added to 16 µL buffer solution (10 X PBS) with a known pH value. To this mixture 2 µL of the 4 mM TAD solution (8 nmol) is added mixed immediately by pipetting up and down several times. In competition experiments 2 µL of both peptide 3 mM stock solutions are added to 14 µL buffer solution (10 X PBS). To this mixture 2 µL of the 4 mM TAD solution (8 nmol) is added mixed immediately by pipetting up and down several times. The reaction of TAD with both tyrosine and tryptophan containing peptides is completed in a matter of seconds, that is why it is important to mix the reaction mixture immediately after addition of TAD reagents. The resulting reaction mixture is analyzed via HPLC and/or LC-MS and/or MALDI-TOF(/TOF) and/or LC (ESI) (ETD/CID/HCD) MS/MS.

2. <u>Analytical data: peptide conjugation</u>

2.1 Characterization of starting peptides

LC-MS data (reversed phase) were recorded on an Agilent 1100 Series instrument with diode array detector (set to 214, 254, 280, 310, 360 nm), equipped with a Phenomenex Kinetex C18 100 Å (150 x 4.6 mm, 5 µm, at 35 °C), hyphenated to an Agilent ESI-single quadrupole MS detector type VL. Mass detection operated in the positive mode. Linear gradient elutions were performed by flushing 0.5 min with A followed by 0 to 100% buffer B in 6 minutes and finally by a 2 min flushing with B using a binary solvent system composed of buffer A: 5 mM NH_4OAc (**1a**-**1f**) or 0.1% HCOOH in H2O (**1i**, **1j)** and B: MeCN with a flow of 1.5 mL/min at 35 °C. A solution of 4-5 mg αcyano-4-hydroxycinnamic acid in 500µL MeCN, 490µL mQ, 10µL 1 M ammonium citrate, 1µL TFA was used as a matrix for MALDI-TOF-MS.

Asn-Trp-Ala-Ser-OH (NWAS, 1a). MS (ESI): m/z 477.1 (calcd $[M+H]^+$ 477.2) retention time 3.32 min. Purity: >95 % (HPLC analysis at 214 nm)

Asn-Tyr-Ala-Ser-OH (NYAS, 1b). MS (ESI): m/z 454.1 (calcd $[M+H]^+$ 454.2) retention time 2.86 min. Purity: >95 % (HPLC analysis at 214 nm)

Asn-Ser-Ala-Trp-OH (NSAW, 1c). MS (ESI): m/z 477.1 (calcd $[M+H]^+$ 477.2) retention time 3.26 min. Purity: >95 % (HPLC analysis at 214 nm)

Asn-Ser-Ala-Tyr-OH (NSAY, 1d). MS (ESI): m/z 454.1 (calcd $[M+H]^+$ 454.2) retention time 2.81 min. Purity: >95 % (HPLC analysis at 214 nm)

Trp-Ser-Ala-Asn-OH (WSAN, 1e). MS (ESI): m/z 477.1 (calcd $[M+H]^+$ 477.2) retention time 3.23 min. Purity: >95 % (HPLC analysis at 214 nm)

Tyr-Ser-Ala-Asn-OH (YSAN, 1f). MS (ESI): m/z 454.1 (calcd $[M+H]^+$ 454.2) retention time 2.86 min. Purity: >95 % (HPLC analysis at 214 nm)

Lys-Lys-Ser-Tyr-Leu-Ser-Pro-Arg-Thr-Ala-Leu-Ile-Asn-Phe-Leu-Val-OH (KKSYLSPRTALINFLV, 1g). MS (ESI): m/z 1849.8 (calcd $[M+H]^+$ 1850.1) retention time: 3.95 min. Purity: >95 % (HPLC analysis at 214 nm)

Lys-Lys-Ser-Trp-Leu-Ser-Pro-Arg-Thr-Ala-Leu-Ile-Asn-Phe-Leu-Val-OH (KKSWLSPRITALINFLV, 1h). MS (ESI): m/z 1872.8 (calcd $[M+H]^+$ 1873.1) retention time 4.02 min. Purity: >95 % (HPLC analysis at 214 nm)

Val-Trp-Ser-Asn-Arg-His-Phe-Tyr-OH (VWSNRHFY, 1i). MS (ESI): m/z 1108.4 (calcd $[M+H]^+$ 1108.5) retention time: 3.40 min. Purity: >95 % (HPLC analysis at 214 nm)

Val-Tyr-Ser-Asn-Arg-His-Phe-Trp-OH (VYSNRHFW, 1j). MS (ESI): m/z 1108.4 (calcd $[M+H]^+$ 1108.5) retention time 3.48 min. Purity: >95 % (HPLC analysis at 214 nm)

Val-Trp-Ser-Gln-Lys-Arg-His-Phe-Gly-Tyr-OH (VWSQKRHFGY, 1k). MS (MALDI-TOF): m/z 1307.7 (calcd $[M+H]^+$ 1307.7)

Lys-Asp-Tyr-Trp-Glu-Cys-Ala-OH (KDYWECA, 1l). MS (MALDI-TOF): m/z 914.4 (calcd $[M+H]^+$ 914.4)

2.2 LC (MS) data of peptide-TAD conjugates

2.2.1 pH dependency of the reaction between tyrosine and TAD

pH experiments were performed to investigate the pH dependency of TAD with tyrosine. Several conjugation reactions of TAD-propanol **2b** and PTAD **2a** with peptide **1b** were done in a series of 10x PBS buffers at pH 4, 5, 6 and 7. The conjugation reactions were done similarly as described in section 1.3 with more concentrated TAD solutions (12 mM instead of 4mM). The reaction between TAD and tyrosine is finished in several seconds, the background hydrolysis of TAD in aqueuos solutions is competing with the tyrosine conjugation reaction. The bright purple color of TAD-propanol **2b** and PTAD **2a** swiftly disappears upon addition to the peptide solution. The reaction mixture is analyzed by HPLC (214 nm).

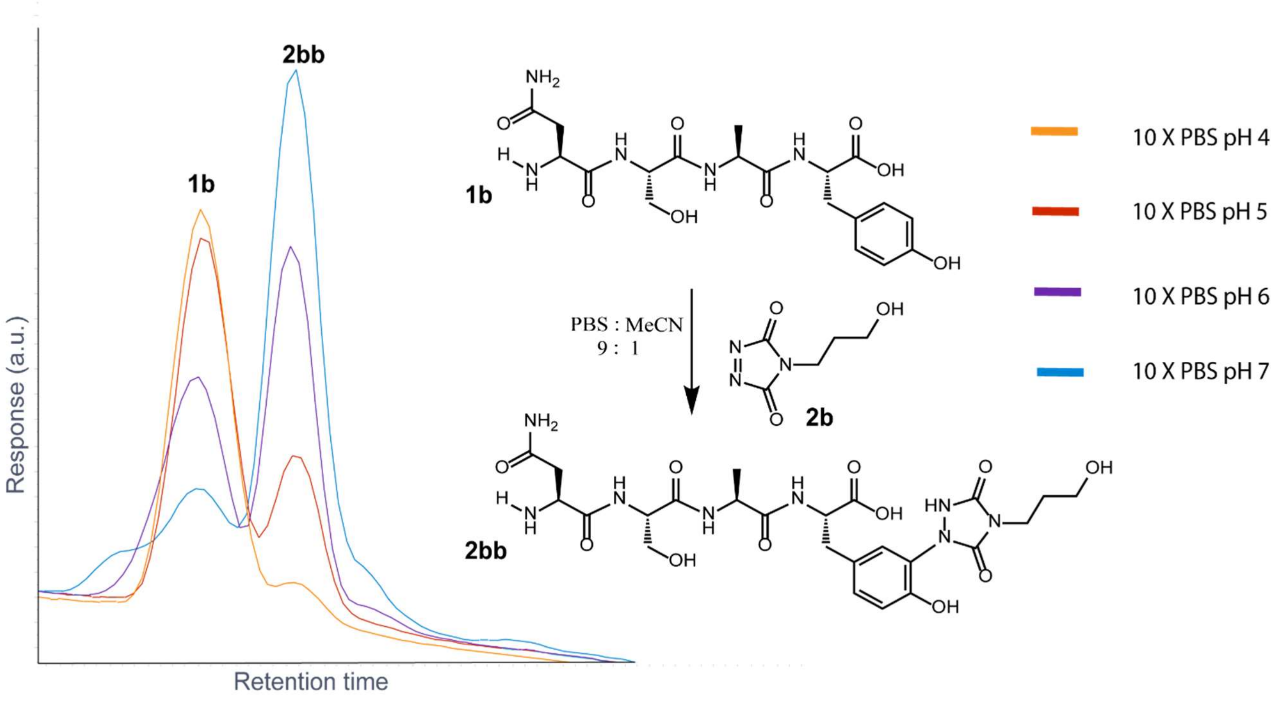

Figure S2.2.1.1 Zoom in of an HPLC chromatogram overlay (214 nm) of the reaction of a peptide **1b** with TAD-propanol **2b** in several 10 X PBS buffers with different pH ranging from pH 4 to pH 7.

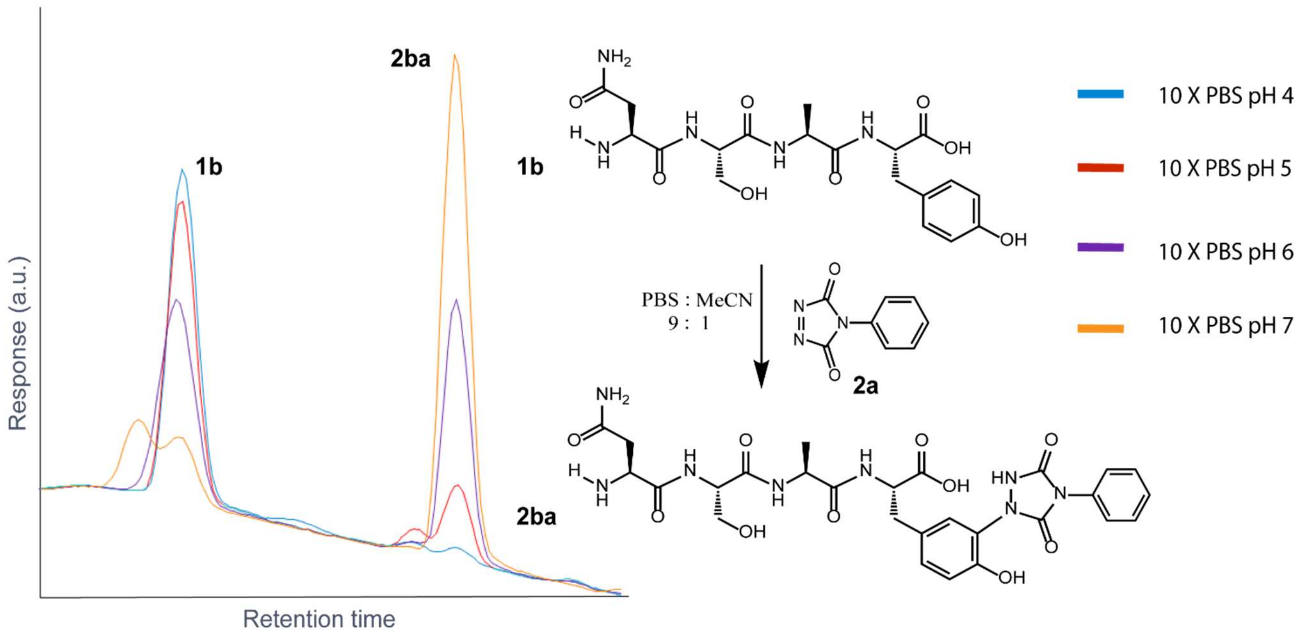

Figure S2.2.1.2 Zoom in of an HPLC chromatogram overlay (214 nm) of the reaction of a peptide **1b** with PTAD **2a** in several 10 X PBS buffers with different pH ranging from pH 4 to pH 7.

For pH 7 we observe almost complete conversion of starting peptide **1b** to give rise to the conjugated product **2bb** (S2.2.1.1) and **2ba** (S2.2.1.2). For the reaction done in pH 4 on the contrary almost no conjugation product is observed. This experiment demonstrates the pH sensitivity of the tyrosine – TAD reaction profile. These results are in accord with the findings on the mechanism of the phenol/phenolate TAD reaction.[2]

2.2.2 Intermolecular Tyr versus Trp competition

The peptide-TAD conjugates are prepared according to the general protocol for peptide-TAD conjugation reported above for the competitive reactions. We found that the reaction of TAD with both tyrosine and tryptophan in buffer is fast (finished in a few seconds). In order to examine the amino acid selectivity of TAD- compounds we performed reactions in a slight excess of TAD (1.25 eq.). Since in buffer there is always background hydrolysis of TAD compounds only a portion of the peptides can react with TAD.

In the previous section the pH dependency of the TAD tyrosine reaction was demonstrated. The generation of TAD-propanol **2b** and PTAD-alkyne **2c** is done via TCCA oxidation and some HCl remains in the MeCN solution with the freshly prepared TAD compound. This HCl can have an impact on the pH of the reaction. The following experiments were carried out in 10 X PBS buffers to ensure the pH is not altered upon addition of the TAD. The LC-chromatogram (214 nm) is provided along with a more detailed zoom of the relevant elution window. Since the conjugated peptide ions often have similar retention times an extraction ion chromatogram (XIC) for the relevant peptide starting material and peptide-TAD conjugate of the same elution window is shown.

Conjugation of Asn-Trp-Ala-Ser-OH (NWAS, 1a) with TAD-propanol 2b (2ab) MS (ESI): m/z 634.2 (calcd $[M+H]^+$ 634.3) retention time 2.85 min.

Conjugation of Asn-Tyr-Ala-Ser-OH (NYAS, 1b) with TAD-propanol 2b (2bb) MS (ESI): m/z 611.1 (calcd $[M+H]^+$ 611.2) retention time 2.83 min.

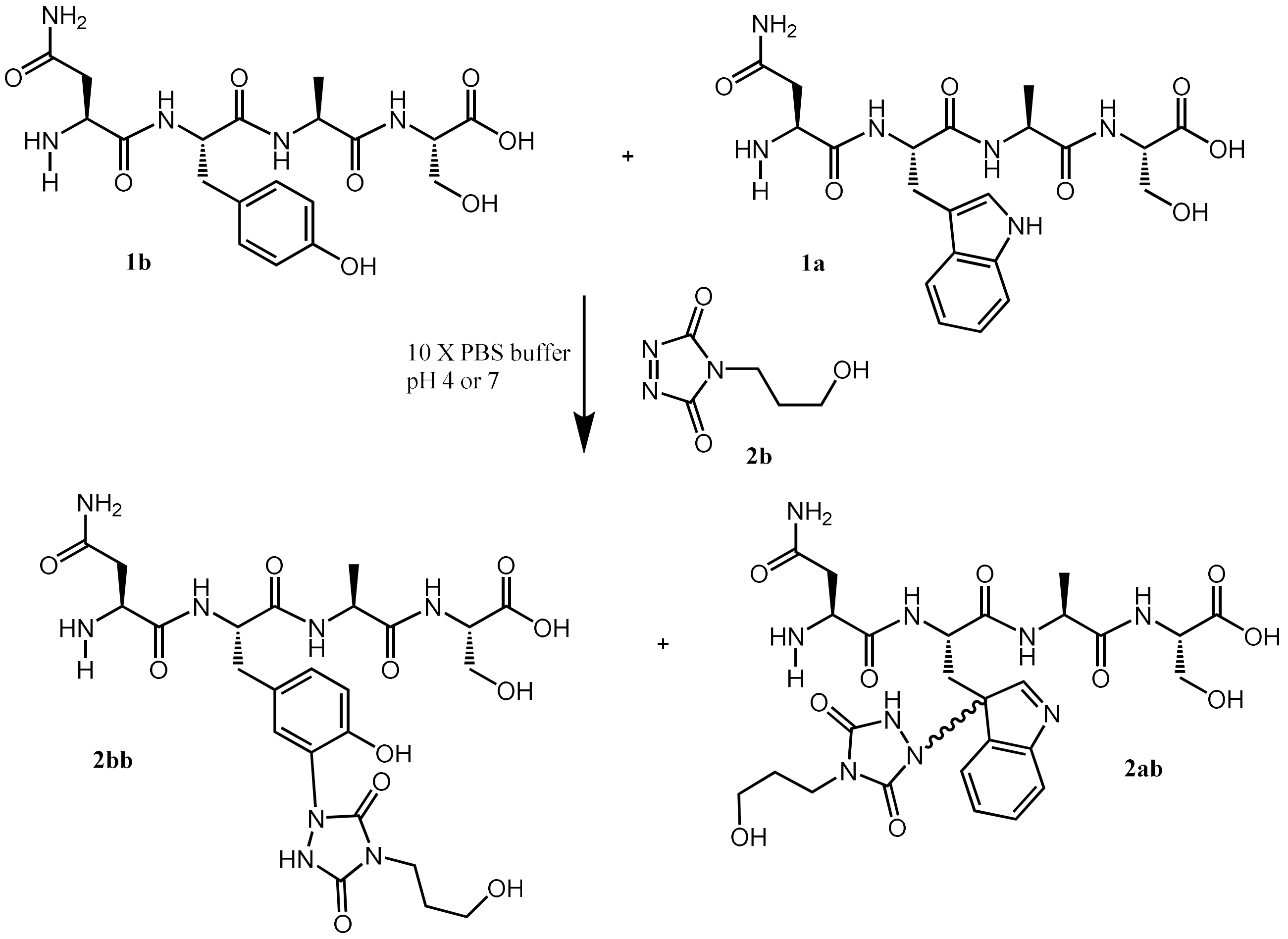

Figure S2.2.2.1 Schematic representation of the reaction of peptides **1b** and **1a** with TAD-propanol **2b** in 10 X PBS buffer pH 4 or 7.

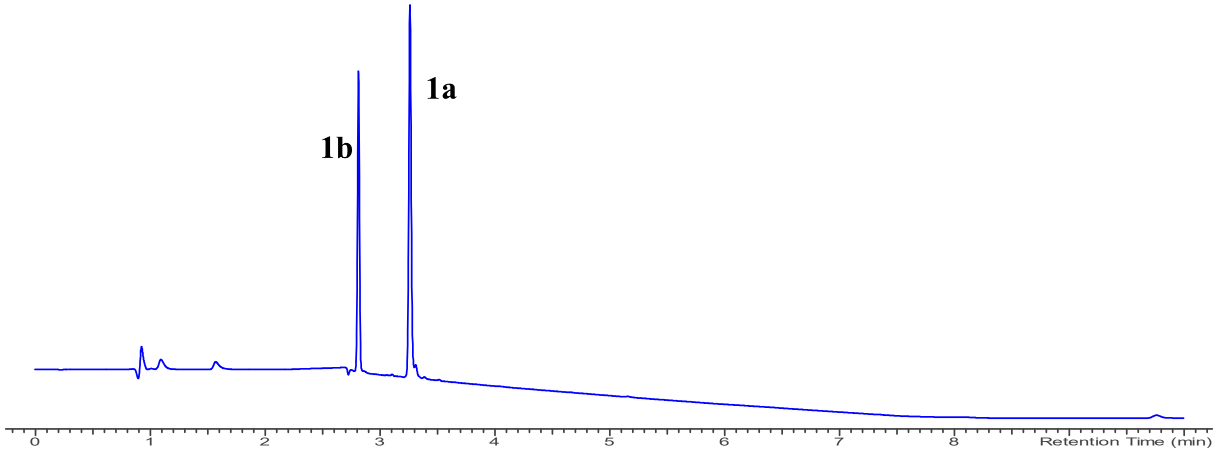

Figure S2.2.2.2 LC chromatogram (214 nm) of a mixture of pure peptides **1a** and **1b**.

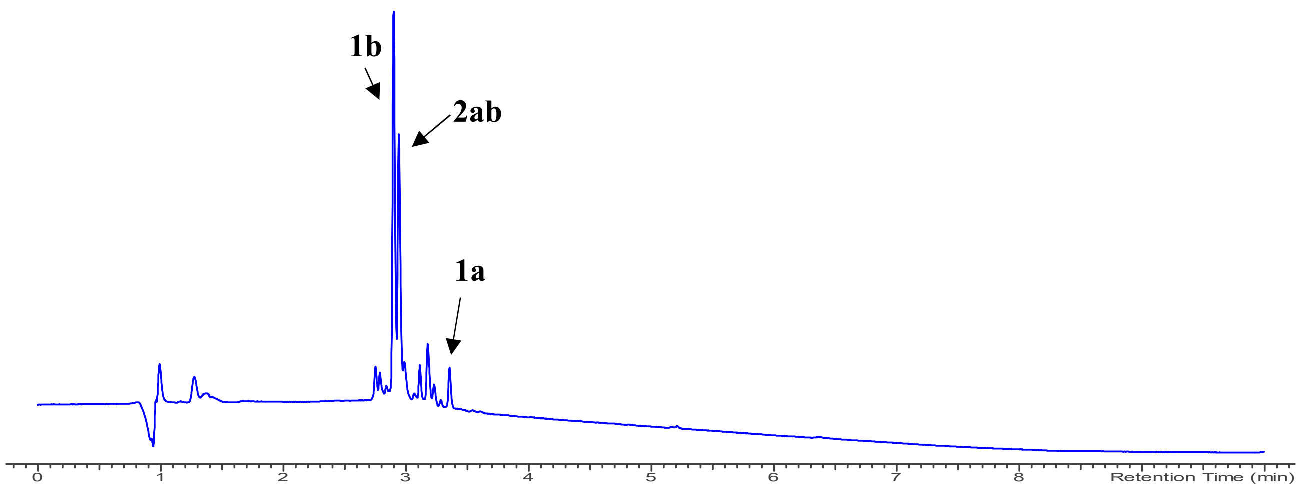

Figure S2.2.2.3 LC chromatogram (214 nm) of **2b** conjugation of a **1a** and **1b** peptide mixture. In this experiment 10 X PBS pH 4 was used as a buffer.

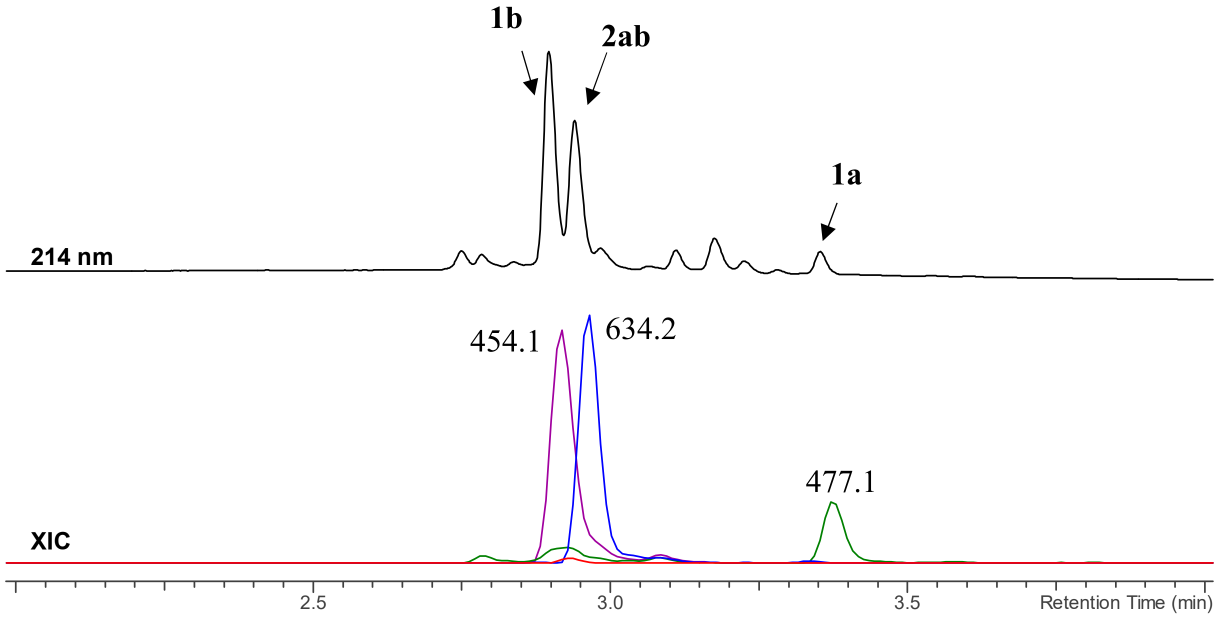

Figure S2.2.2.4 LC chromatogram (214 nm) between t_R = 2 min - 4 min (top) of **2b** conjugation of a **1a** and **1b** peptide mixture in 10 X PBS pH 4. Extracted ion chromatogram (XIC) between t_R = 2 min - 4 min (bottom) for the peptide ions **1a** (477.1 Da, green) **1b** (454.1 Da, purple) and the conjugated peptide ions **2ab** (634.2 Da, blue) and **2bb** (611.1 Da, red). Product **2bb** is not formed at pH 4.

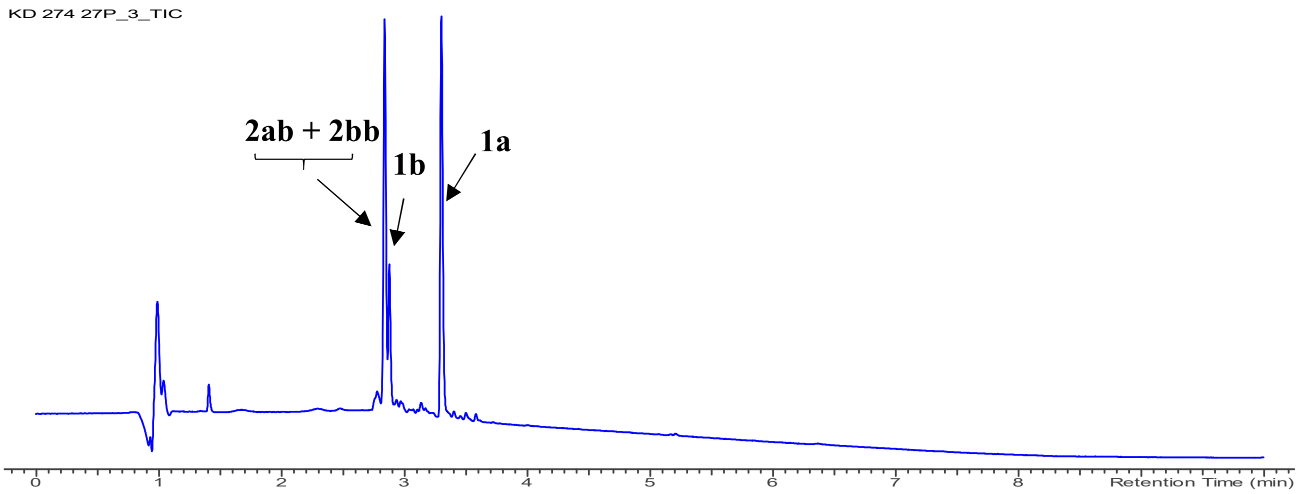

Figure S2.2.2.5 LC chromatogram (214 nm) of **2b** conjugation of a **1a** and **1b** peptide mixture. In this experiment 10 X PBS pH 7 was used as a buffer.

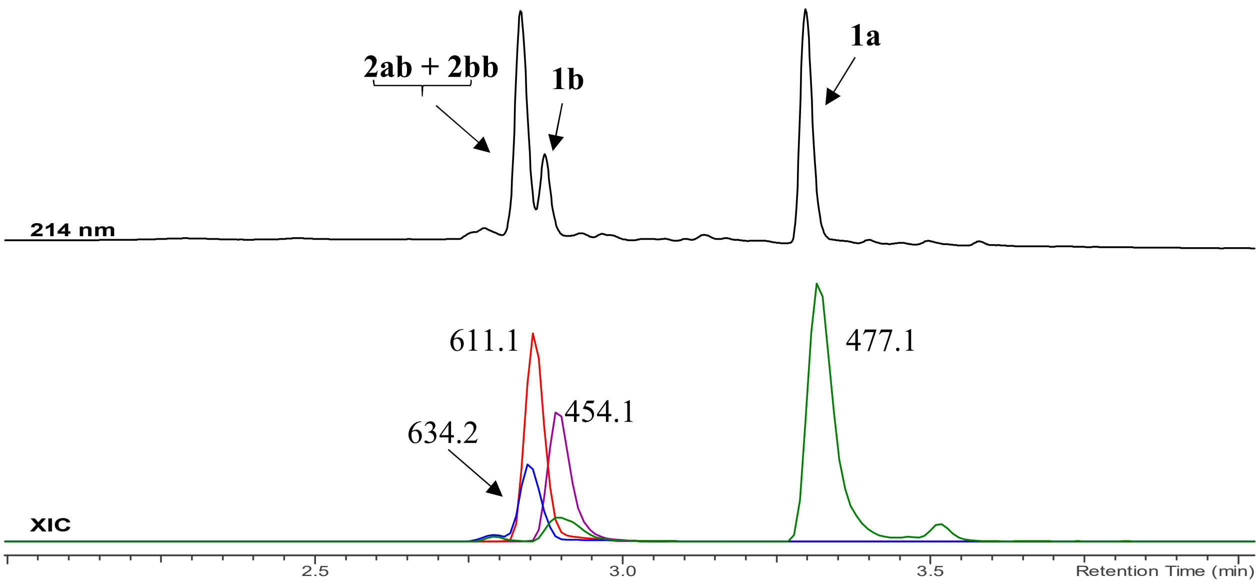

Figure S2.2.2.6 LC chromatogram (214 nm) between t_R = 2 min - 4 min (top) of **2b** conjugation of a **1a** and **1b** peptide mixture in 10 X PBS pH 7. Extracted ion chromatogram (XIC) between t_R = 2 min - 4 min (bottom) for the peptide ions **1a** (477.1 Da, green) **1b** (454.1 Da, purple) and the conjugated peptide ions **2ab** (634.2 Da, blue) and **2bb** (611.1 Da, red). Both products **2ab** and **2bb** are formed at pH 7.

Conjugation of Asn-Trp-Ala-Ser-OH (NWAS, 1a) with PTAD-alkyne 1c (2ac) MS (ESI): m/z 706.2 (calcd $[M+H]^+$ 706.3) retention time 3.51 min.

Conjugation of Asn-Tyr-Ala-Ser-OH (NYAS, 1b) with PTAD-alkyne 1c (2bc) MS (ESI): m/z 683.1 (calcd $[M+H]^+$ 683.2) retention time 3.51 min.

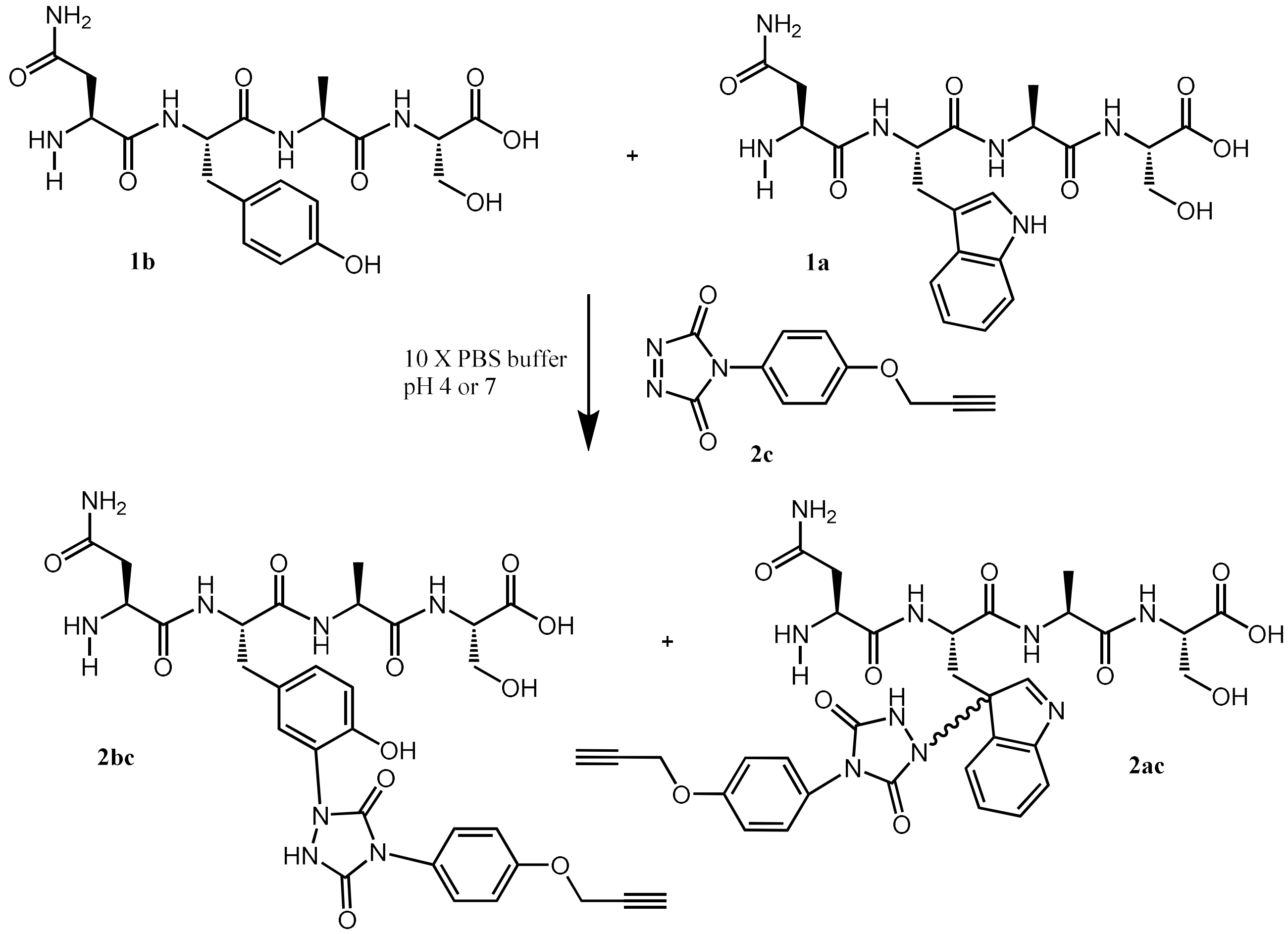

Figure S2.2.2.7 Schematic representation of the reaction of peptides **1b** and **1a** with PTAD-alkyne **2c** in 10 X buffer pH 4 or 7.

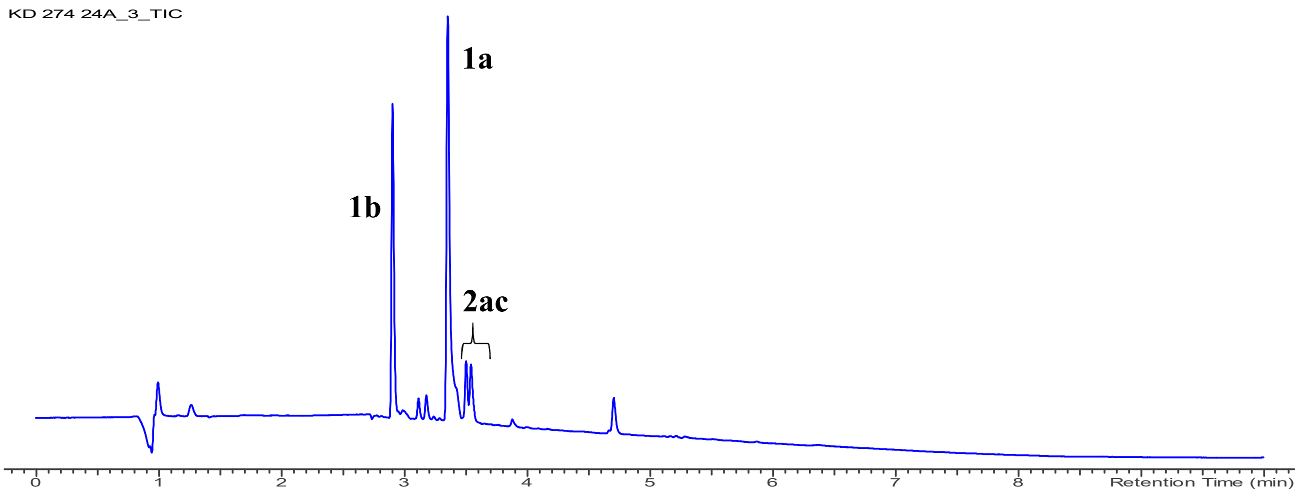

Figure S2.2.2.8 LC chromatogram (214 nm) of **2c** conjugation of a **1a** and **1b** peptide mixture. In this experiment 10 X PBS pH 4 was used as a buffer.

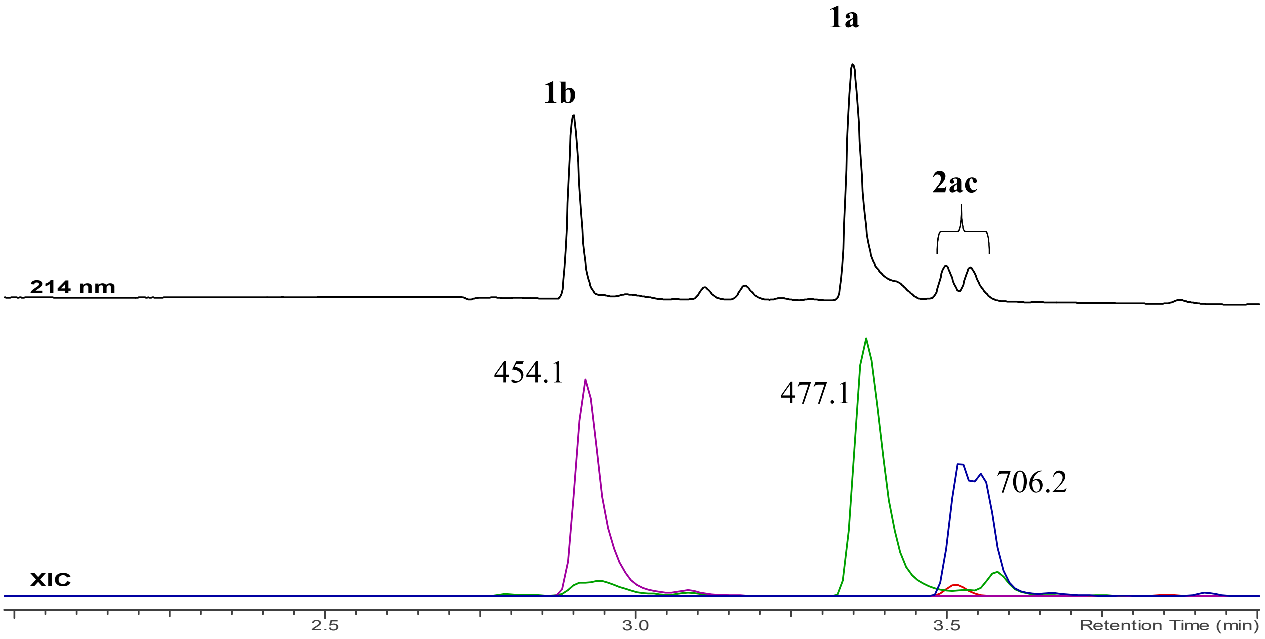

Figure S2.2.2.9 LC chromatogram (214 nm) between t_R = 2 min - 4 min (top) of **2c** conjugation of a **1a** and **1b** peptide mixture in 10 X PBS pH 4. Extracted ion chromatogram (XIC) between t_R = 2 min - 4 min (bottom) for the peptide ions **1a** (477.1 Da, green) **1b** (454.1 Da, purple) and the conjugated peptide ions **2ac** (706.2 Da, blue) and **2bc** (683.1 Da, red). Product **2bb** is almost not formed at pH 4.

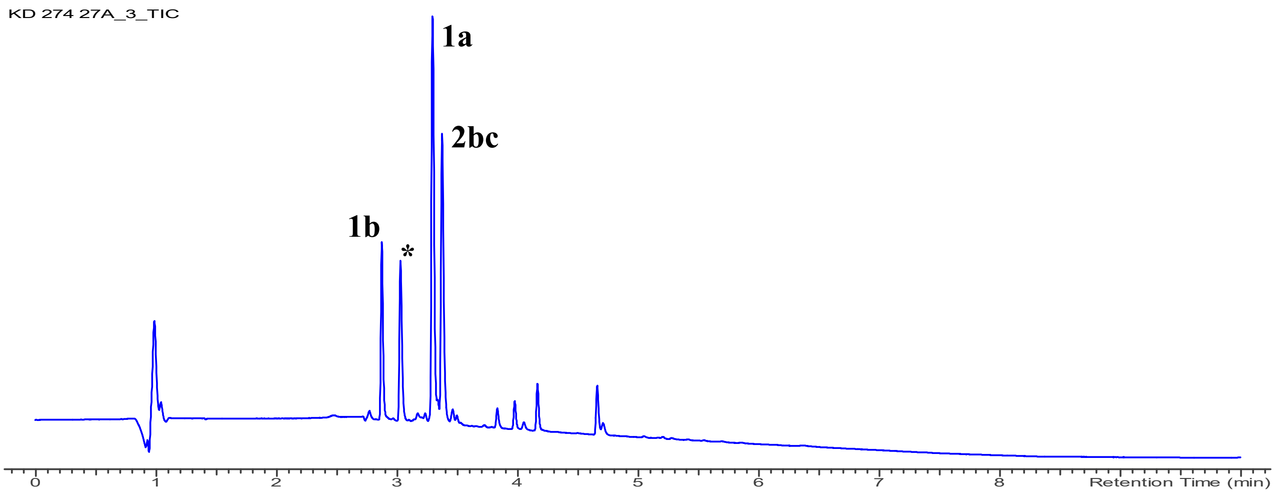

Figure S2.2.2.10 LC chromatogram (214 nm) of **2c** conjugation of a **1a** and **1b** peptide mixture. In this experiment 10 X PBS pH 7 was used as a buffer. The peak annotated with * corresponds with a mass of 232 Da corresponding to the reduced form of **2c**.

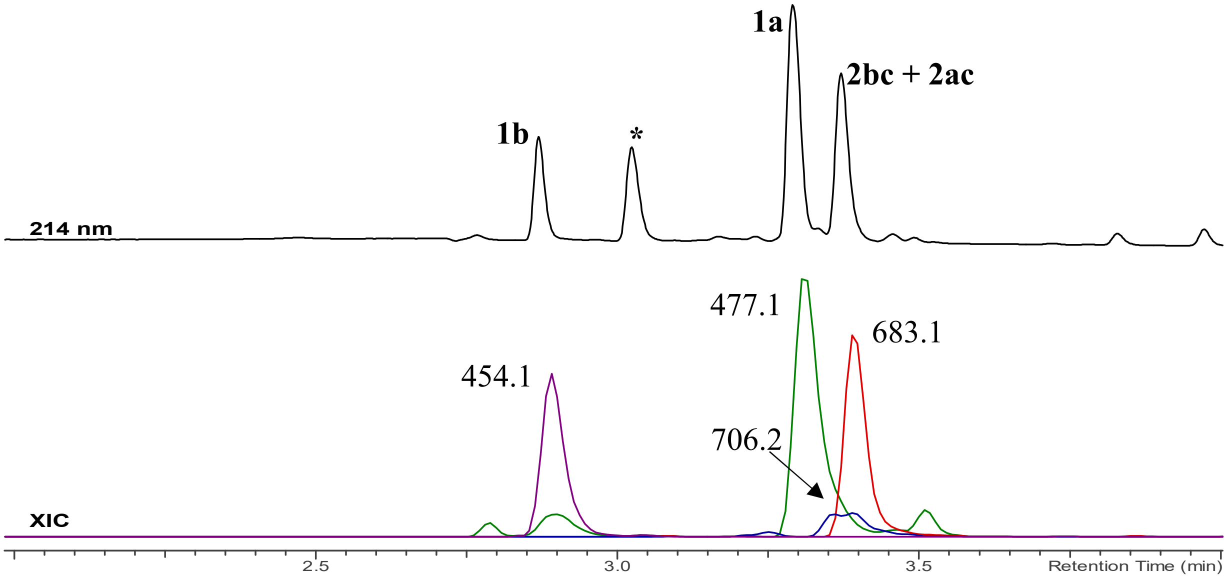

Figure S2.2.2.11 LC chromatogram (214 nm) between t_R = 2 min - 4 min (top) of **2c** conjugation of a **1a** and **1b** peptide mixture in 10 X PBS pH 7. Extracted ion chromatogram (XIC) between t_R = 2 min - 4 min (bottom) for the peptide ions **1a** (477.1 Da, green) **1b** (454.1 Da, purple) and the conjugated peptide ions **2ac** (706.2 Da, blue) and **2bc** (683.1 Da, red). Product **2bc** is clearly the main product but also **2ac** is formed at pH 7.

Conjugation of Asn-Ser-Ala-Trp-OH (NSAW, 1c) with TAD propanol 2b (2cb) MS (ESI): m/z 634.2 (calcd $[M+H]^+$ 634.3) retention time 2.72 min + 2.94 – 3.04 min.

Conjugation of Asn-Ser-Ala-Tyr-OH (NSAY, 1d) with TAD propanol 2b (2db) MS (ESI): m/z 611.2 (calcd $[M+H]^+$ 611.2) retention time 2.78 min.

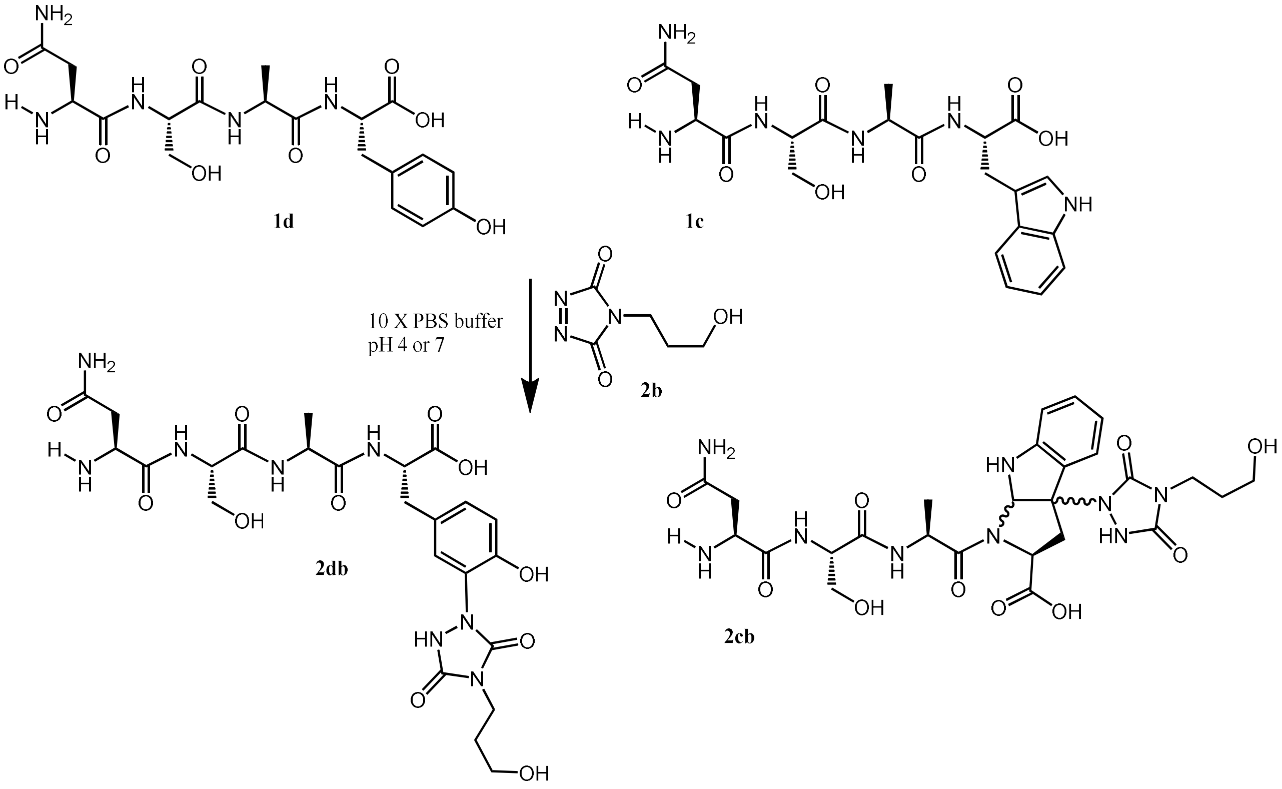

Figure S2.2.2.12 Schematic representation of the reaction of peptides **1c** and **1d** with TAD-propanol **2b** in 10 X PBS buffer pH 4 or 7.

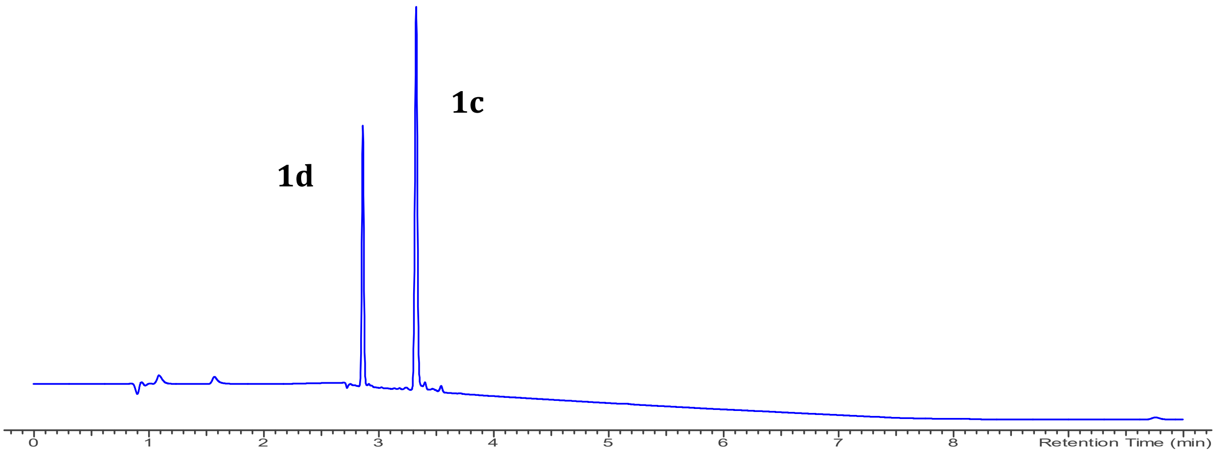

Figure S2.2.2.13 LC chromatogram (214 nm) of a mixture of pure peptides **1c** and **1d**.

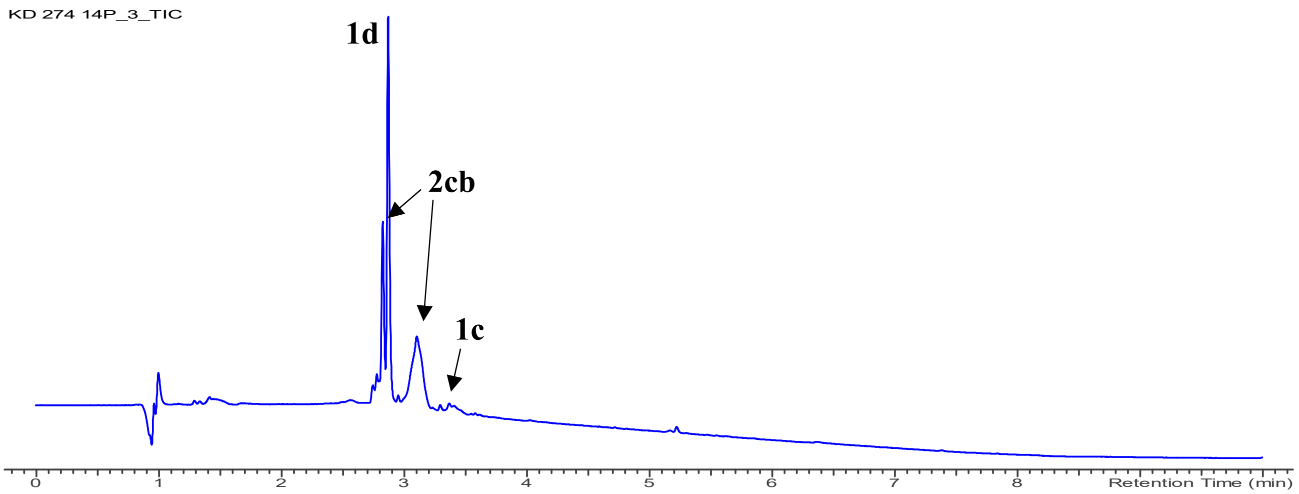

Figure S2.2.2.14 LC chromatogram (214 nm) of **2b** conjugation of a **1c** and **1d** peptide mixture.. In this experiment 10 X PBS pH 4 was used as a buffer.

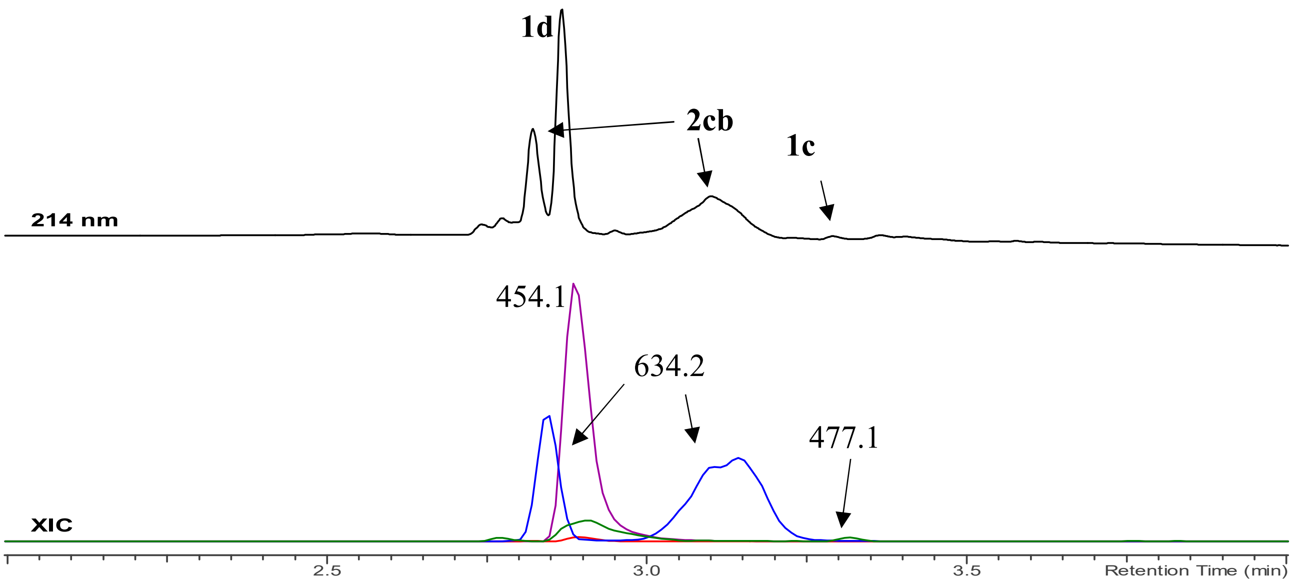

Figure S2.2.2.15 LC chromatogram (214 nm) between t_R = 2 min - 4 min (top) of **2b** conjugation of a **1c** and **1d** peptide mixture in 10 X PBS pH 4. Extracted ion chromatogram (XIC) between t_R = 2 min - 4 min (bottom) for the peptide ions **1c** (477.1 Da, green) **1d** (454.1 Da, purple) and the conjugated peptide ions **2cb** (634.2 Da, blue) and **2db** (611.1 Da, red). Peptide **1c** is almost completely consumed and the corresponding product **2cb** is formed. Product **2db** is not observed at pH 4.

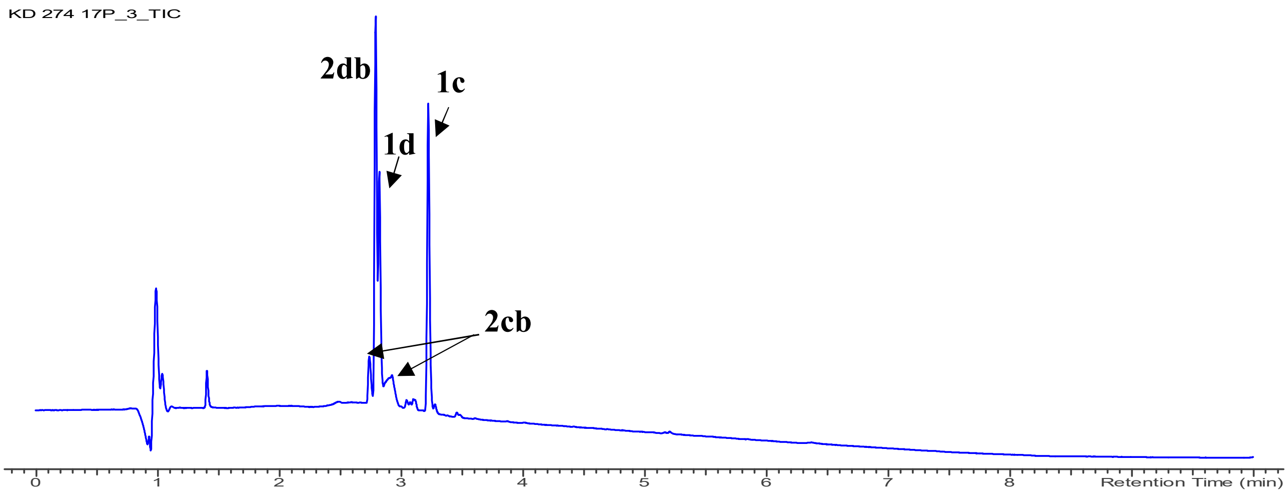

Figure S2.2.2.16 LC chromatogram (214 nm) of **2b** conjugation of a **1c** and **1d** peptide mixture.. In this experiment 10 X PBS pH 7 was used as a buffer.

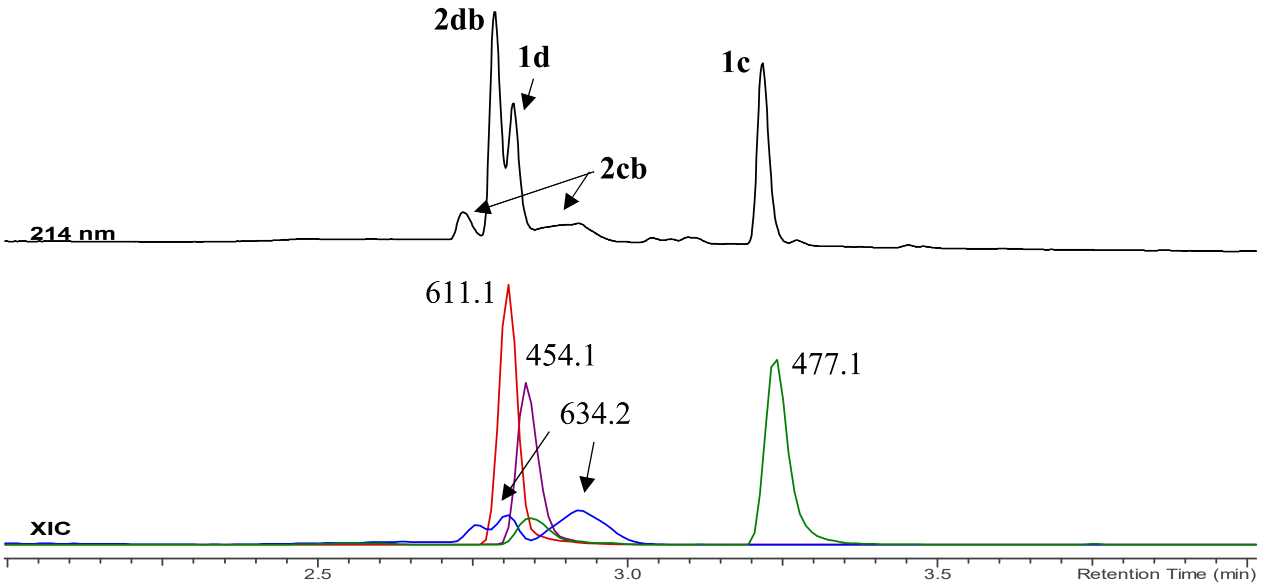

Figure S2.2.2.17 LC chromatogram (214 nm) between t_R = 2 min - 4 min (top) of **2b** conjugation of a **1c** and **1d** peptide mixture in 10 X PBS pH 7. Extracted ion chromatogram (XIC) between t_R = 2 min - 4 min (bottom) for the peptide ions **1c** (477.1 Da, green) **1d** (454.1 Da, purple) and the conjugated peptide ions **2cb** (634.2 Da, blue) and **2db** (611.1 Da, red). Both product **2cb** and **2db** are formed with the main product being **2db** at pH 7.

Conjugation of Asn-Ser-Ala-Trp-OH (NSAW, 1c) with PTAD-alkyne 2c (2cc) MS (ESI): m/z 706.2 (calcd $[M+H]^+$ 706.3) retention time 3.42 min + 3.61 – 6.68min.

Conjugation of Asn-Ser-Ala-Tyr-OH (NSAY, 1d) with PTAD-alkyne 2c (2dc) MS (ESI): m/z 683.1 (calcd $[M+H]^+$ 683.2) retention time 3.44 min.

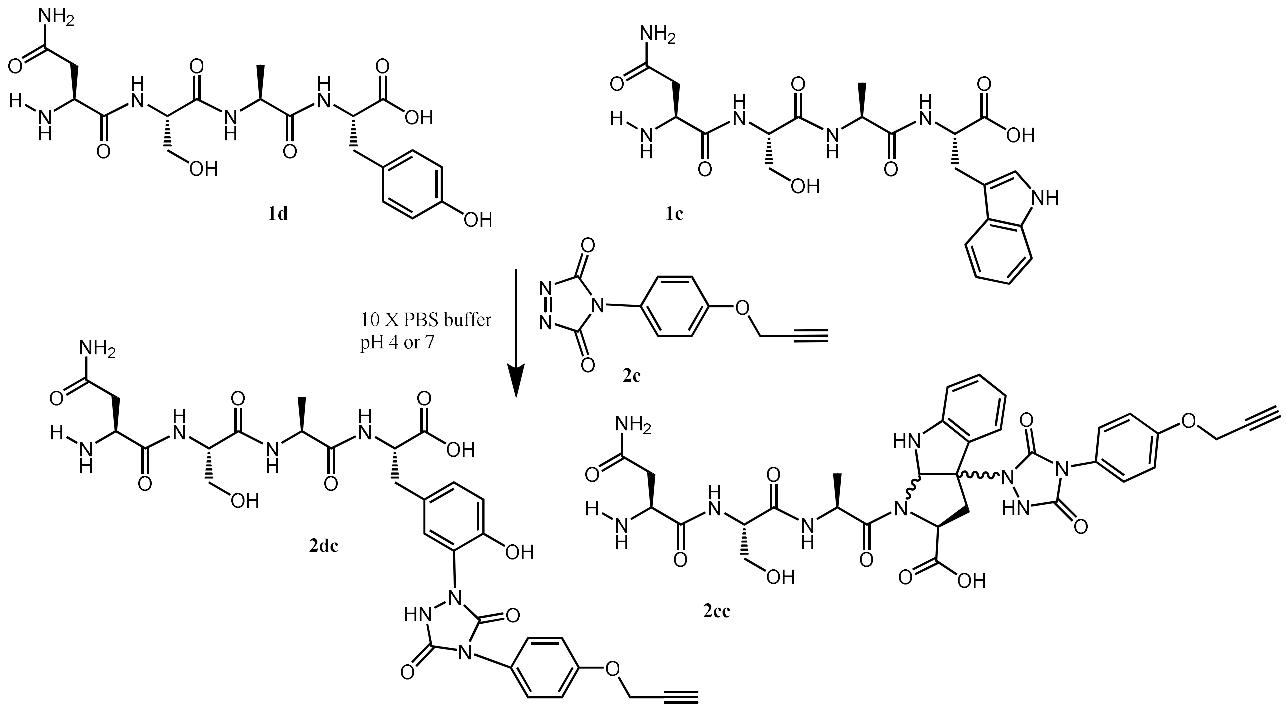

Figure S2.2.2.18 Schematic representation of the reaction of peptides **1c** and **1d** with PTAD-alkyne **2c** in 10 X PBS buffer pH 4 or 7.

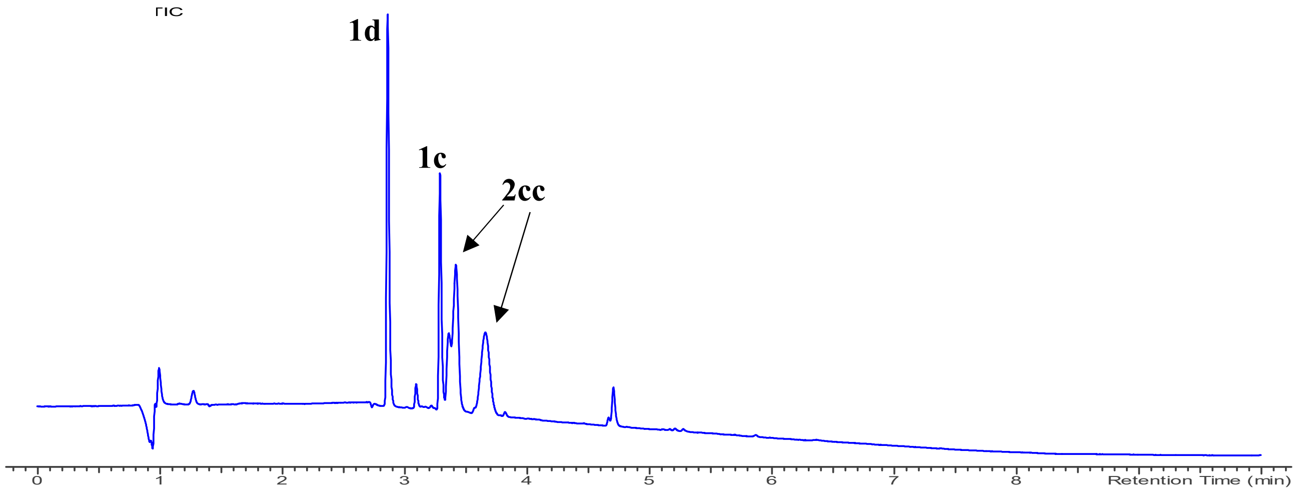

Figure S2.2.2.19 LC chromatogram (214 nm) of **2c** conjugation of a **1c** and **1d** peptide mixture. In this experiment 10 X PBS pH 4 was used as a buffer.

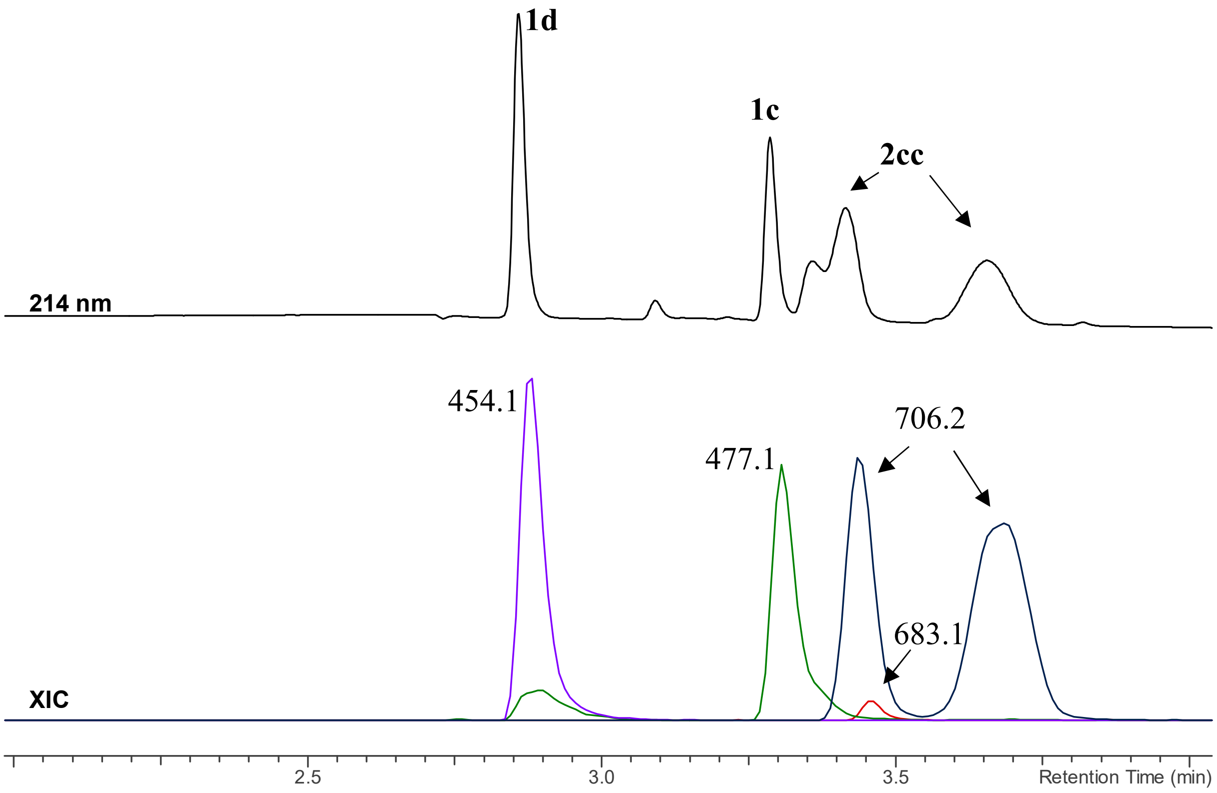

Figure S2.2.2.20 LC chromatogram (214 nm) between t_R = 2 min - 4 min (top) of **2c** conjugation of a **1c** and **1d** peptide mixture in 10 X PBS pH 4. Extracted ion chromatogram (XIC) between t_R = 2 min - 4 min (bottom) for the peptide ions **1c** (477.1 Da, green) **1d** (454.1 Da, purple) and the conjugated peptide ions **2cc** (706.2 Da, blue) and **2dc** (683.1 Da, red). Almost no product **2dc** is observed at pH 4.

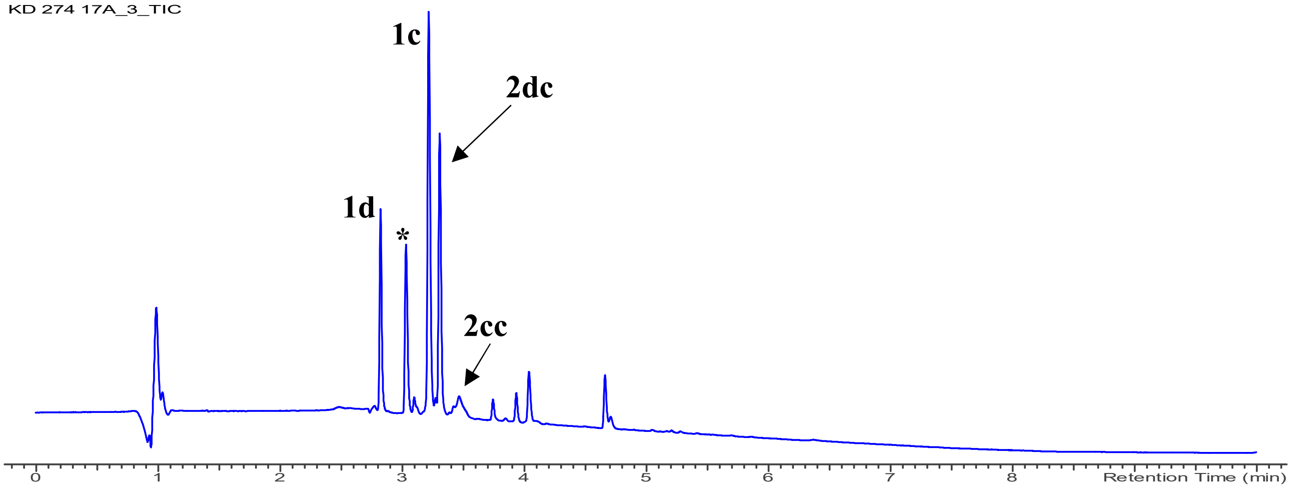

Figure S2.2.2.21 LC chromatogram (214 nm) of **2c** conjugation of a **1c** and **1d** peptide mixture. In this experiment 10 X PBS pH 7 was used as a buffer. The peak annotated with * corresponds with a mass of 232 Da corresponding to the reduced form of **2c**.

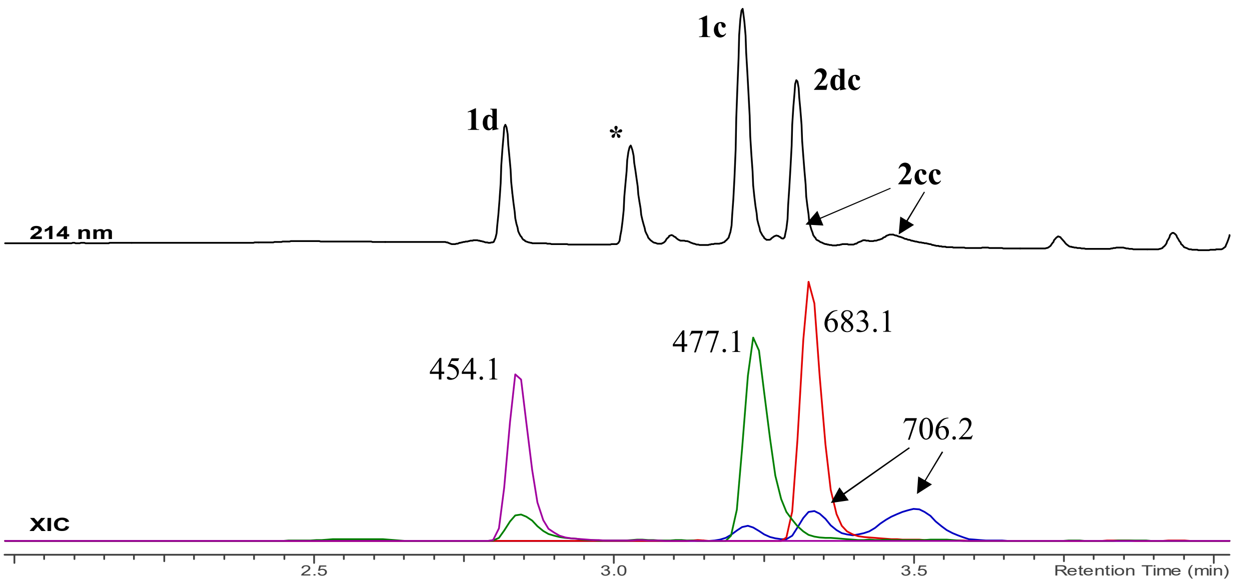

Figure S2.2.2.22 LC chromatogram (214 nm) between t_R = 2 min - 4 min (top) of **2c** conjugation of a **1c** and **1d** peptide mixture in 10 X PBS pH 7. Extracted ion chromatogram (XIC) between t_R = 2 min - 4 min (bottom) for the peptide ions **1c** (477.1 Da, green) **1d** (454.1 Da, purple) and the conjugated peptide ions **2cc** (706.2 Da, blue) and **2dc** (683.1 Da, red). Both products **2dc** and **2cc** are formed with **2dc** as the main product at pH 7.

Remarkably for peptide with a C-terminal tryptophan amino acid NSAW **1c** the reaction mixture after reaction with TAD-propanol **2b** or PTAD-alkyne **2c** contained several reaction product peaks in the LC-MS analysis. These peaks correspond to diastereoisomers of reaction product **2cc** or **2cb**, this product has undergone an additional annulation caused by the reaction of the lone pair on the backbone nitrogen with the indole C2 after reaction of TAD with the indole C3. These structural findings were confirmed via NMR analysis of Boc-Trp-OH and N-Ac-Trp-OMe adducts with TAD-propanol 2b (SI 4). These findings are in agreement with the results reported by Baran et al.[3] on non-peptide related TAD-indole reactions.

Conjugation of Trp-Ser-Ala-Asn-OH (WSAN, 1e) with TAD-propanol 2b (2eb). MS (ESI): m/z 634.2 (calcd $[M+H]^+$ 634.3) retention time 2.97 min.

Conjugation of Tyr-Ser-Ala-Asn-OH (YSAN, 1f) with TAD-propanol 2b (2fb). MS (ESI): m/z 611.2 (calcd $[M+H]^+$ 611.2) retention time 2.72 min.

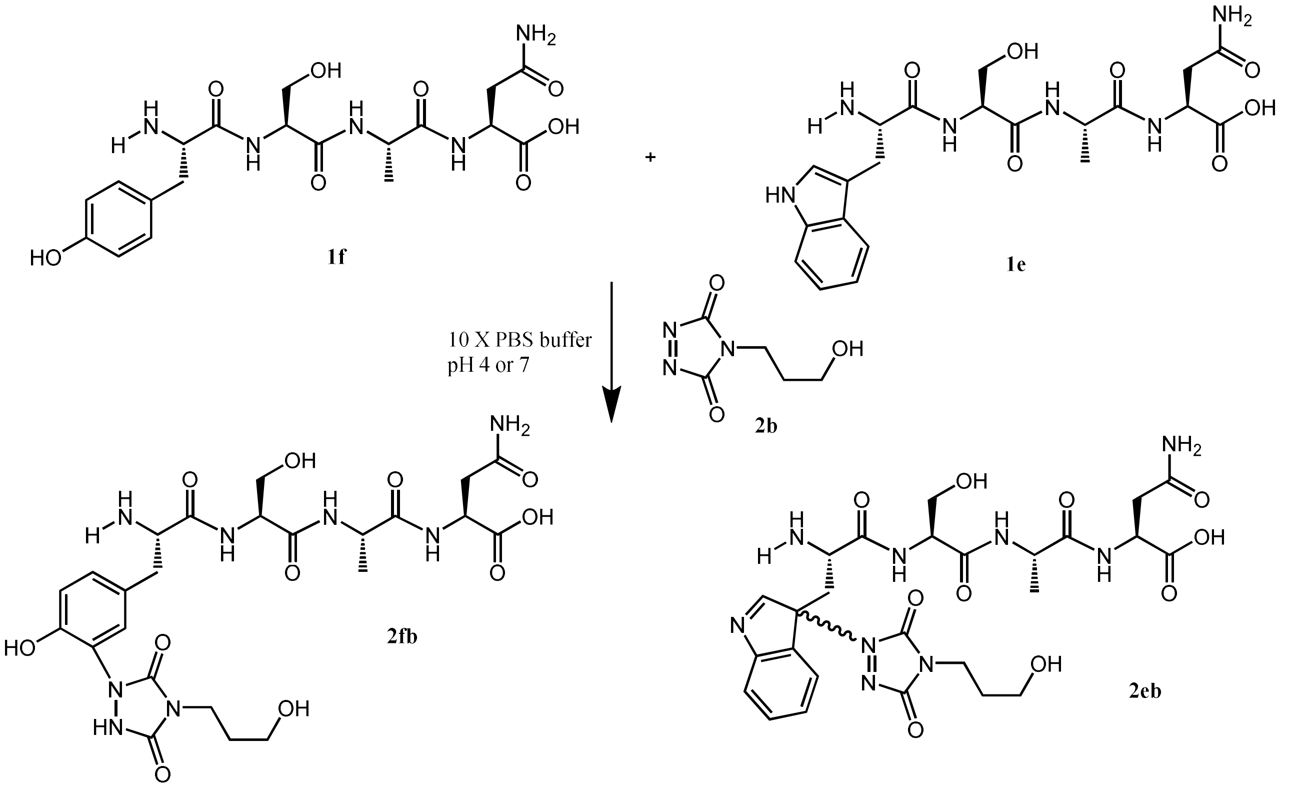

Figure S2.2.2.23 Schematic representation of the reaction of peptides **1e** and **1f** with TAD-propanol **2b** in 10 X PBS buffer pH 4 or 7.

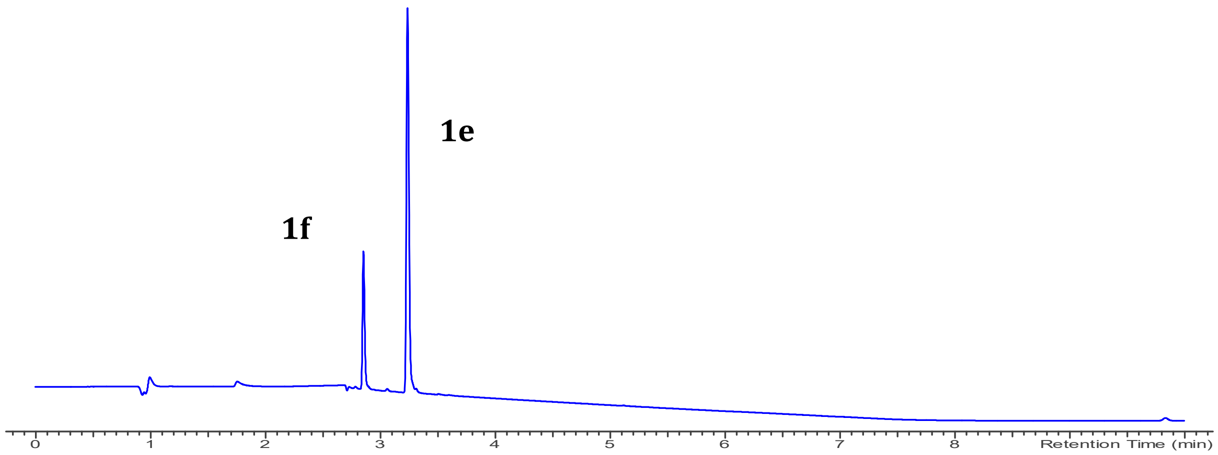

Figure S2.2.2.24 LC chromatogram (214 nm) of a mixture of pure peptides **1e** and **1f**.

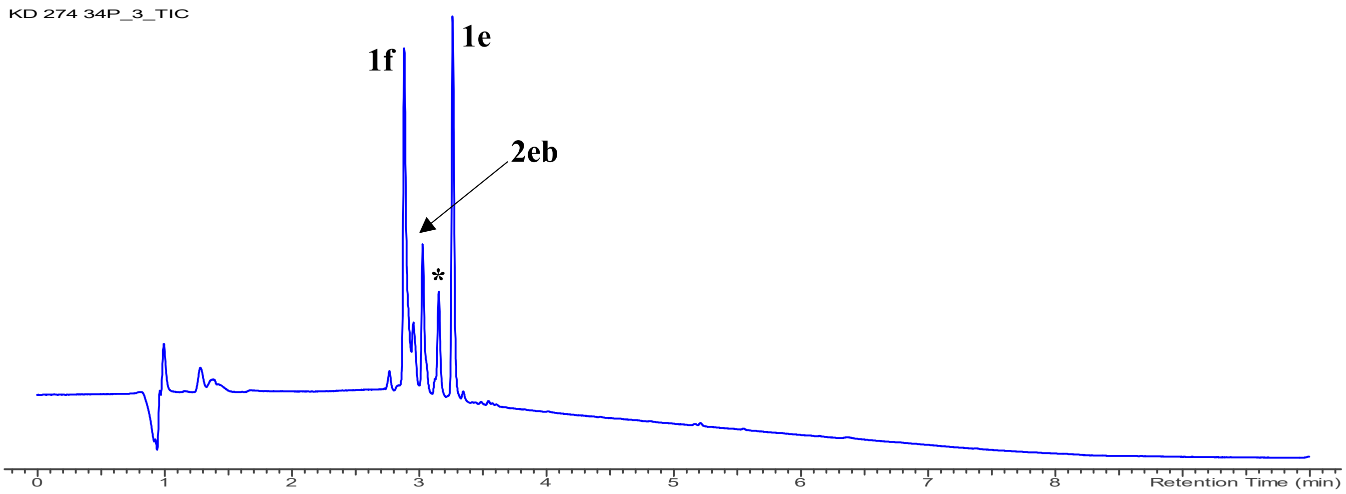

Figure S2.2.2.25 LC chromatogram (214 nm) of **2b** conjugation of a **1e** and **1f** peptide mixture. In this experiment 10 X PBS pH 4 was used as a buffer. The peak annotated with * corresponds with a mass of 509 Da.

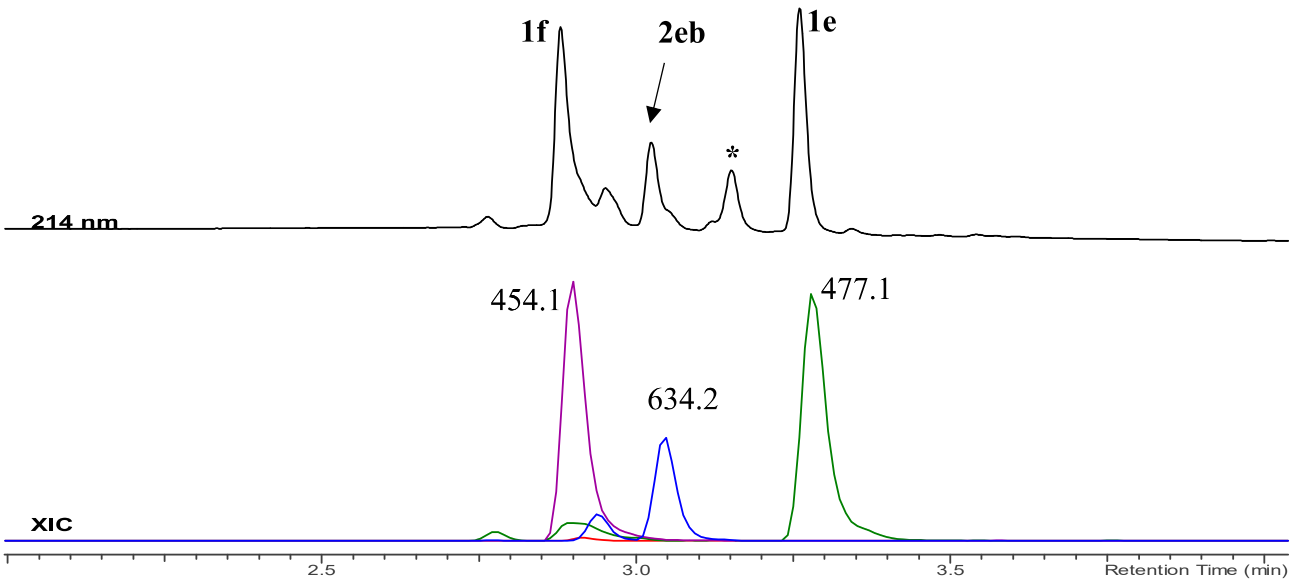

Figure S2.2.2.26 LC chromatogram (214 nm) between t_R = 2 min - 4 min (top) of **2b** conjugation of a **1e** and **1f** peptide mixture in 10 X PBS pH 4. Extracted ion chromatogram (XIC) between t_R = 2 min - 4 min (bottom) for the peptide ions **1e** (477.1 Da, green) **1f** (454.1 Da, purple) and the conjugated peptide ions **2eb** (634.2 Da, blue) and **2fb** (611.1 Da, red). Product **2fb** was not observed at pH 4.

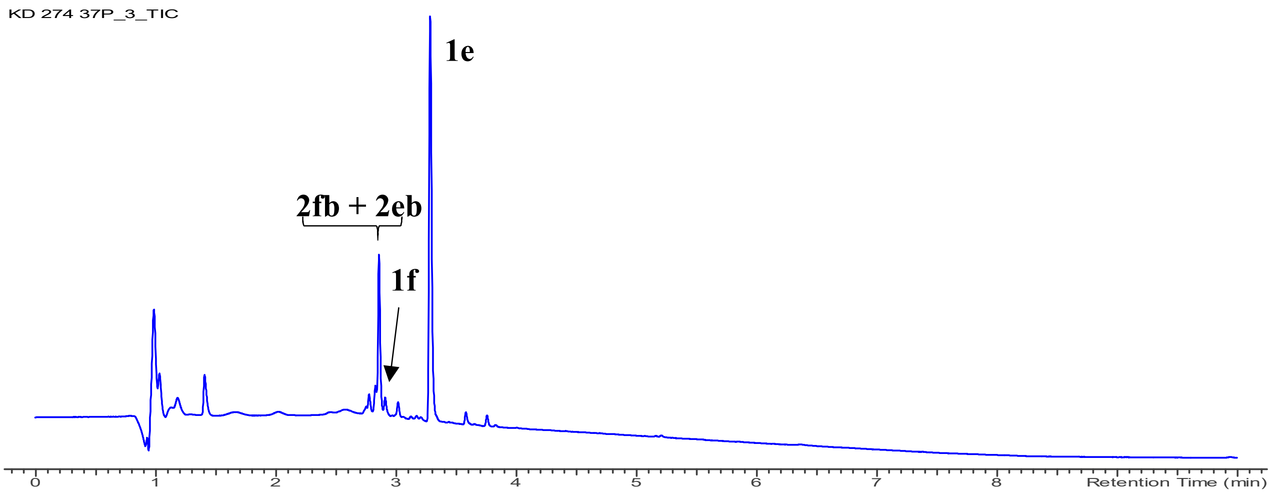

Figure S2.2.2.27 LC chromatogram (214 nm) of **2b** conjugation of a **1e** and **1f** peptide mixture. In this experiment 10 X PBS pH 7 was used as a buffer.

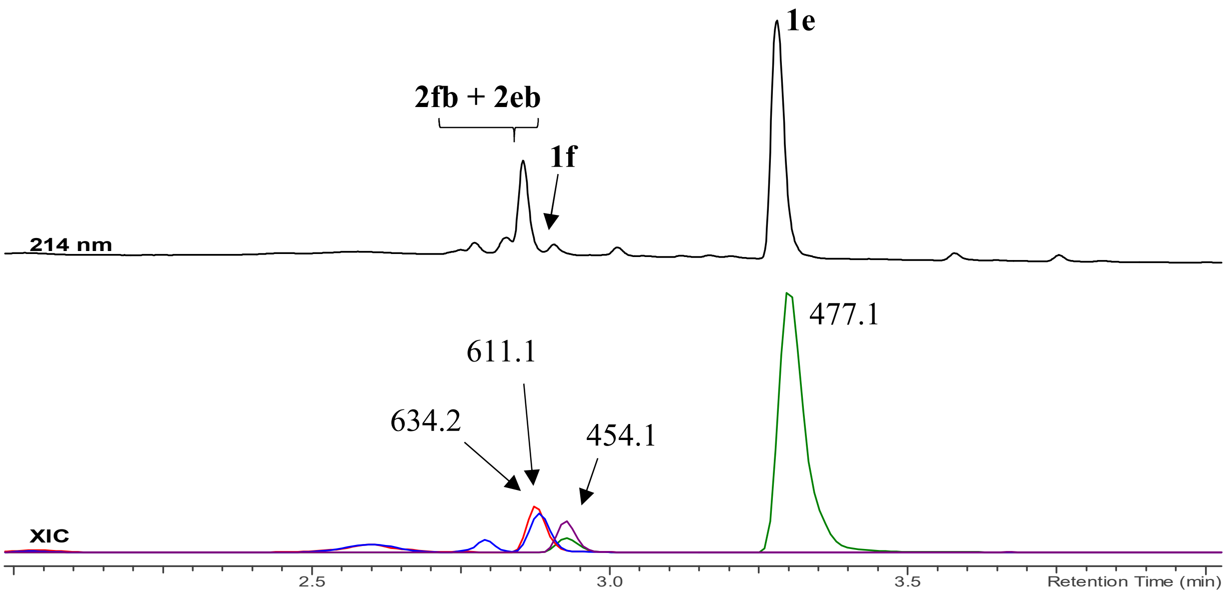

Figure S2.2.2.28 LC chromatogram (214 nm) between t_R = 2 min - 4 min (top) of **2b** conjugation of a **1e** and **1f** peptide mixture in 10 X PBS pH 7. Extracted ion chromatogram (XIC) between t_R = 2 min - 4 min (bottom) for the peptide ions **1e** (477.1 Da, green) **1f** (454.1 Da, purple) and the conjugated peptide ions **2eb** (634.2 Da, blue) and **2fb** (611.1 Da, red). Both products **2fb** and **2eb** are formed at pH 7.

Conjugation of Lys-Lys-Ser-Tyr-Leu-Ser-Pro-Arg-Thr-Ala-Leu-Ile-Asn-Phe-Leu-Val-OH (KKSYLSPRTALINFLV 1g) with DMEQ-TAD 2d (2gd). MS (ESI): (M+3)/3: 732.4 (calcd $[M+3H]^{+3}$ 732.4) retention time: 4.00 min.

Conjugation of Lys-Lys-Ser-Trp-Leu-Ser-Pro-Arg-Thr-Ala-Leu-Ile-Asn-Phe-Leu-Val-OH (KKSWLSPRTALINFLV 1h) with DMEQ-TAD 2b (2hd). MS (ESI): (M+3)/3: 740.1 (calcd $[M+3H]^{+3}$ 740.0) retention time 4.05 min

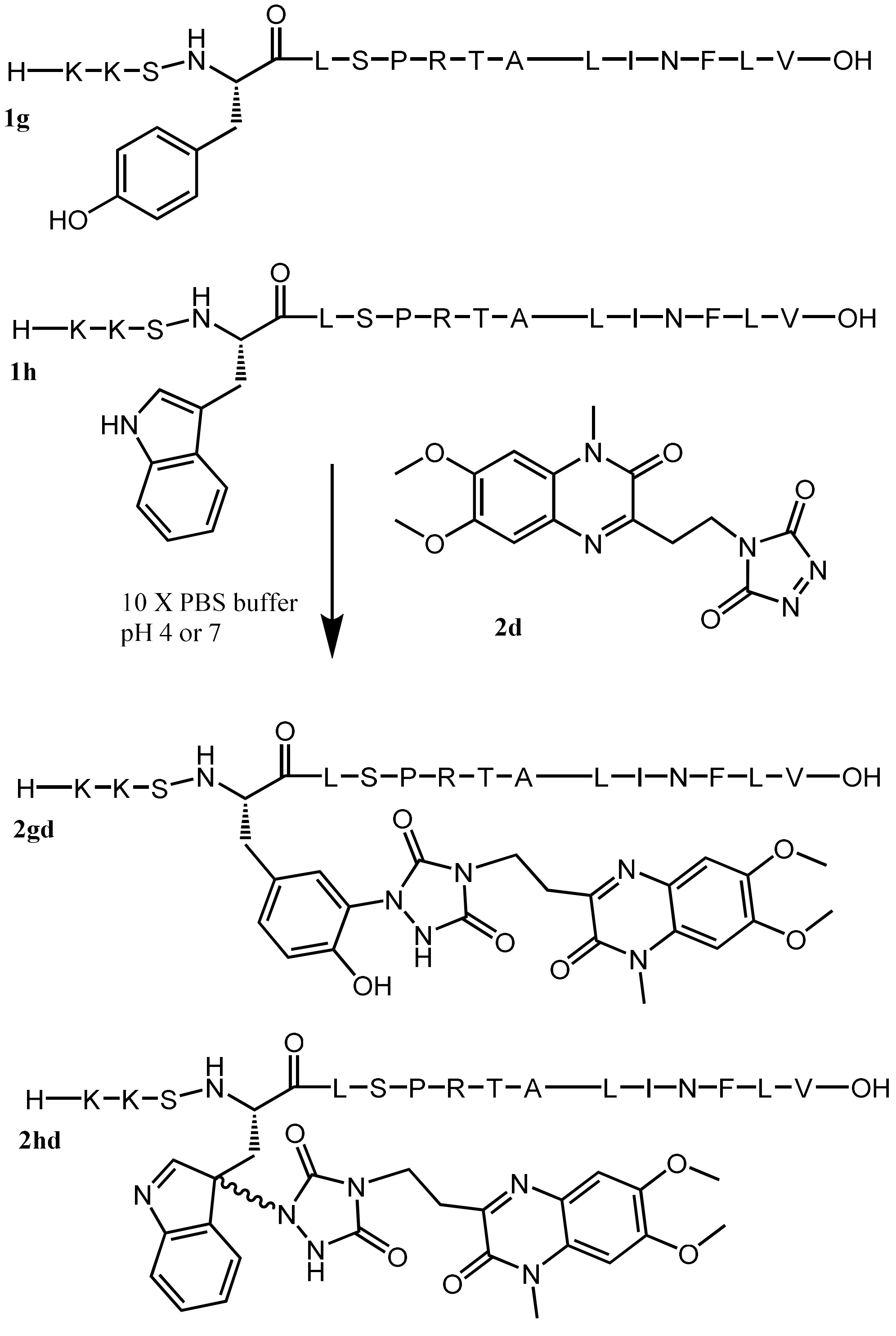

Figure S2.2.2.29 Schematic representation of the reaction of peptides **1g** and **1h** with DMEQ-TAD **2d** in 10 X PBS pH buffer.

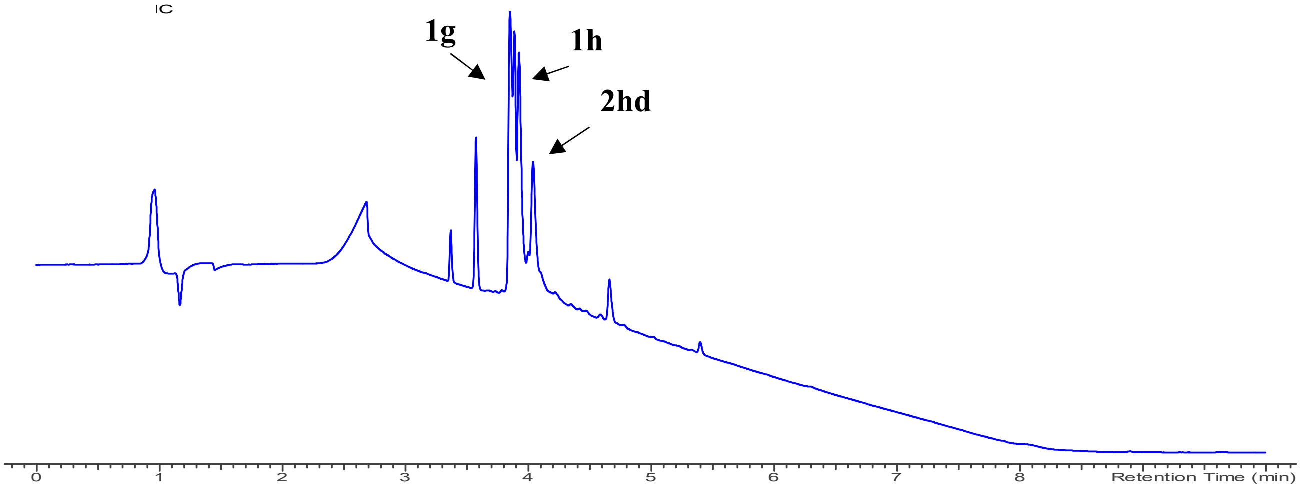

Figure S2.2.2.30 LC chromatogram (214 nm) of **2d** conjugation of a **1g** and **1h** peptide mixture. 10 X PBS pH 4 is used as buffer.

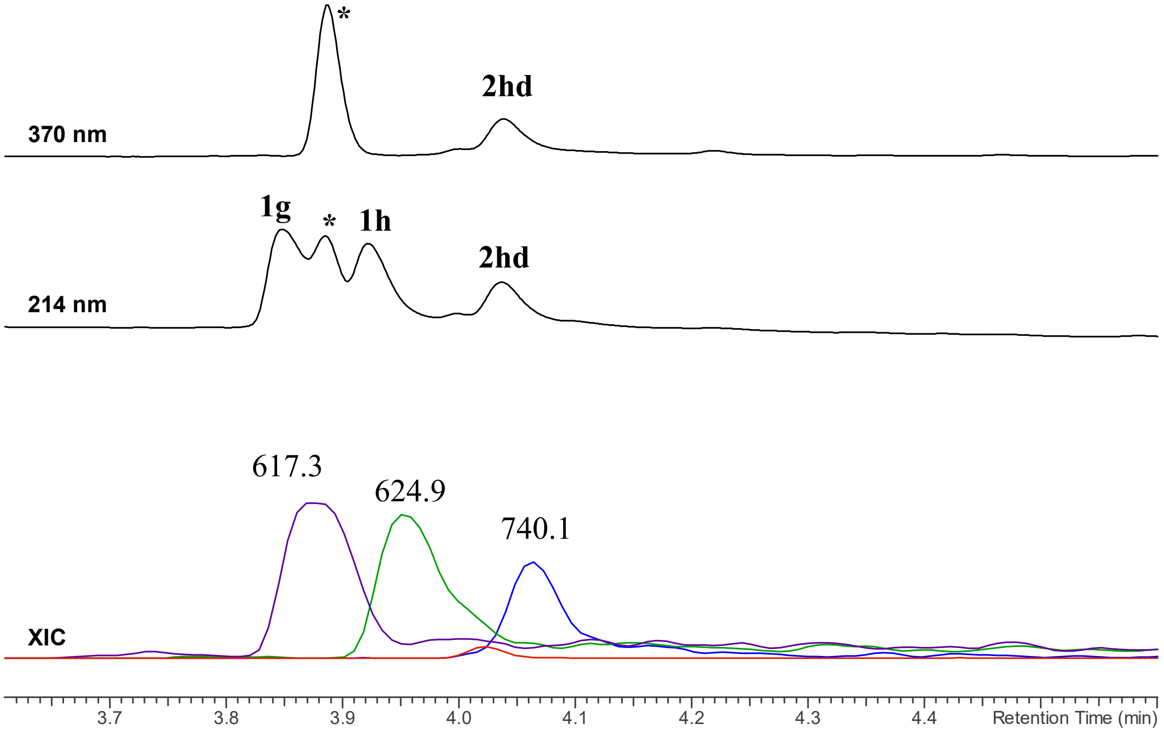

Figure S2.2.2.31 LC chromatograms (370 nm / 214 nm) between t_R = 3.6 min – 4.6 min (top) of **2d** conjugation of a **1g** and **1h** peptide mixture. Extracted ion chromatogram (XIC) between t_R = 3.6 min – 4.6 min (bottom) for the $[M+3H]^{+3}$ peptide ions of **1g** (617.3 Da, purple) and **1f** (624.9 Da, green) and the conjugated peptide $[M+3H]^{+3}$ ions **2gd** (732.4 Da, red) and **2hd** (740.1 Da, blue). 10 X PBS pH 4 is used as buffer. The peak annotated with * corresponds to DMEQ-urazole with a mass of 348 Da.

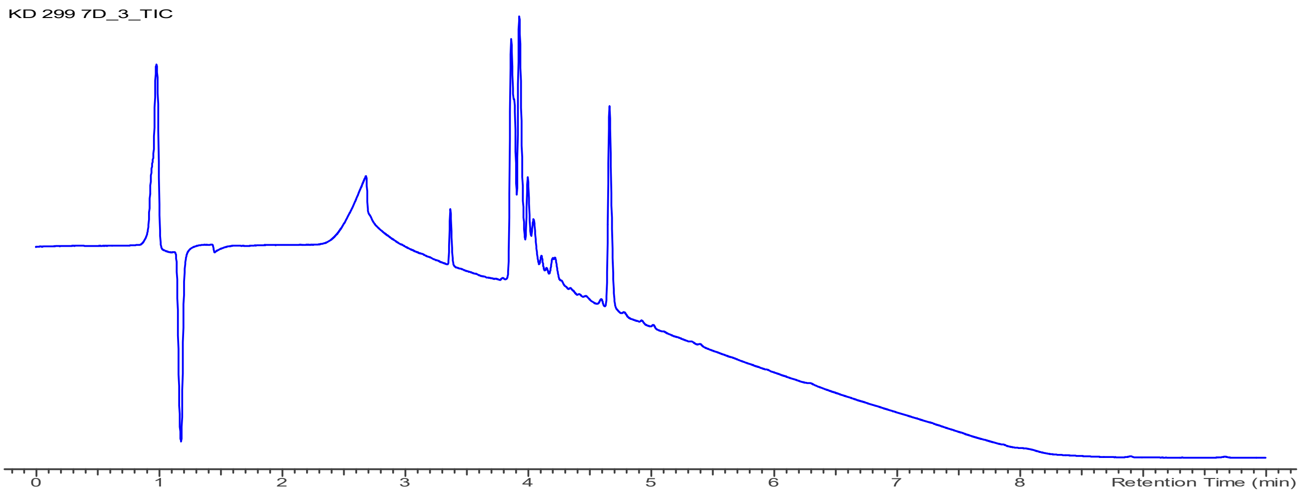

Figure S2.2.2.32 LC chromatogram (214 nm) of **2d** conjugation of a **1g** and **1h** peptide mixture. 10 X PBS pH 7 is used as buffer.

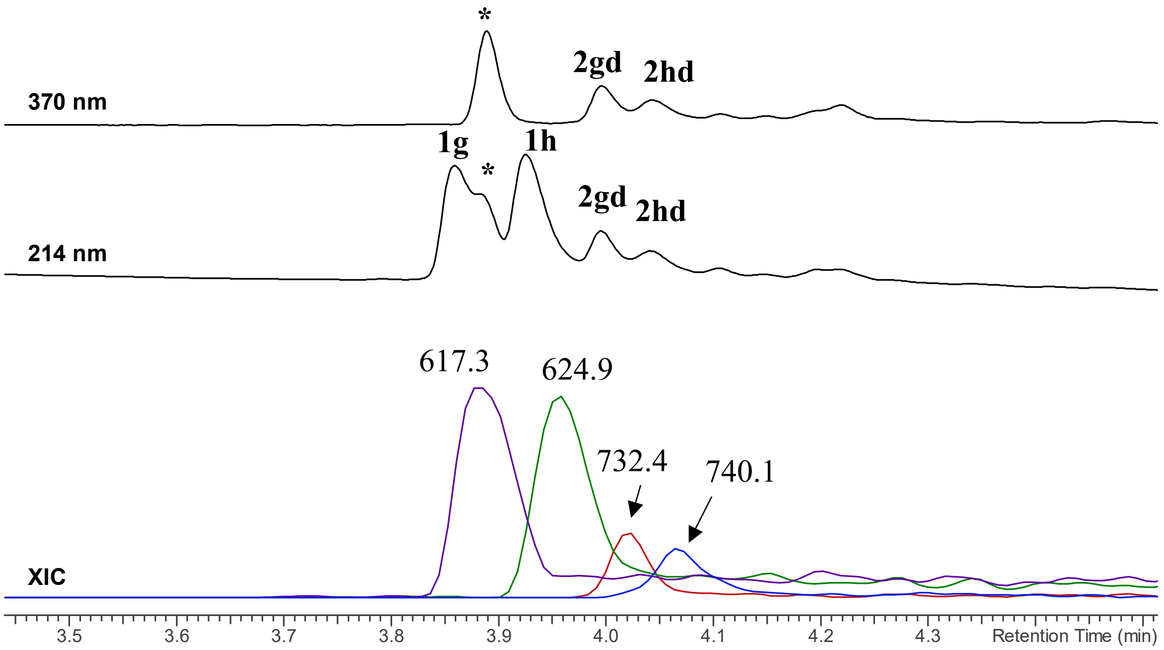

Figure S2.2.2.33 LC chromatograms (370 nm / 214 nm) between t_R = 3.6 min – 4.6 min (top) of **2d** conjugation of a **1g** and **1h** peptide mixture. Extracted ion chromatogram (XIC) between t_R = 3.6 min – 4.6 min (bottom) for the $[M+3H]^{+3}$ peptide ions of **1g** (617.3 Da, purple) and **1f** (624.9 Da, green) and the conjugated peptide $[M+3H]^{+3}$ ions **2gd** (732.4 Da, red) and **2hd** (740.1 Da, blue). 10 X PBS pH 7 is used as buffer. The peak annotated with * corresponds to DMEQ-urazole with a mass of 348 Da.

2.2.3 Peptide conjugate stability

The peptide conjugate stability was examined in 10 X PBS buffer pH 7 at room temperature. The peptide conjugation was performed as described in section **1.3**. Peptide **1e** and TAD-propanol **2b** were used for this stability analysis. Following the conjugation reaction 100 µL of the sample was diluted in 200 µL of 10 X PBS buffer pH 7.4 and the pH was adjusted to a value of 7. The peptide conjugate sample in 10 X PBS pH 7 was left on the lab bench at room temperature for 2 weeks and at several time points a HPLC sample was recorded. The results are presented in figure 2.2.3.1 where an overlay of the HPLC chromatograms (at 214 nm) is presented. The HPLC 214 nm chromatogram peak intensity for conjugate **2eb** demonstrated only minor reduction over the course of 2 weeks. This indicates that the conjugate Trp-TAD linkage is stable for extended periods of time at pH 7.

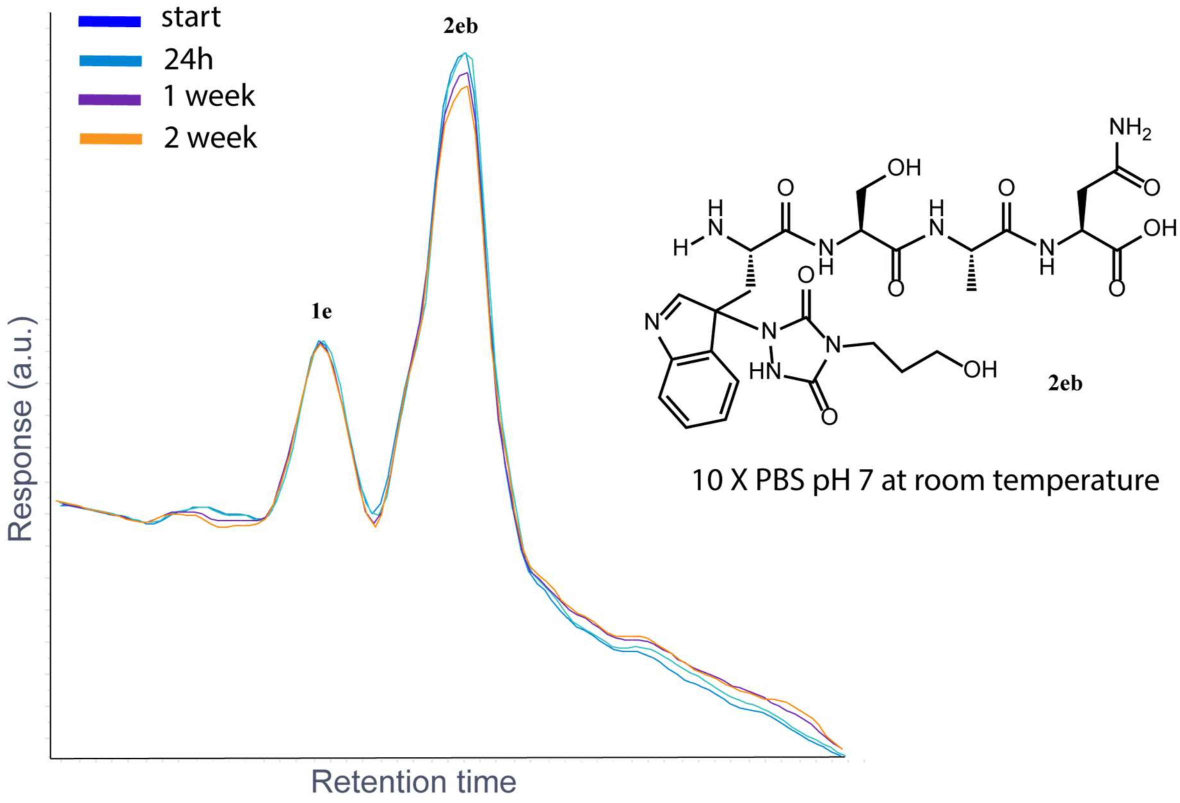

Figure 2.2.3.1 Zoom of an HPLC chromatogram overlay (214 nm) of the peptide conjugate **2eb** in 10 X PBS buffer at pH 7. The conjugate was left at room temperature and at several time points a sample was taken and a chromatogram recorded.

In a following experiment, the stability of the peptide conjugate was examined in 1X PBS buffers at different pH values. Peptide conjugates **2ed** and **2cd** were prepared according to the procedure presented in section 1.3 of this supplementary information with a higher excess of TAD compound (10 equivalents). Conjugates **2ed** and **2cd** were purified using HPLC and lyophilized. Conjugates **2ed** and **2cd** were dissolved in water (at 3 mM) and this solution was diluted (1/10) into PBS pH 4, pH 7 or pH 9. Samples were taken at different time points (t:0; t:1 day; t:1 week) and analysed on HPLC. The results are presented in figures 2.2.3.2 and 2.2.3.3 where an overlay of the HPLC chromatograms (at 214 nm) for all pH conditions is presented.

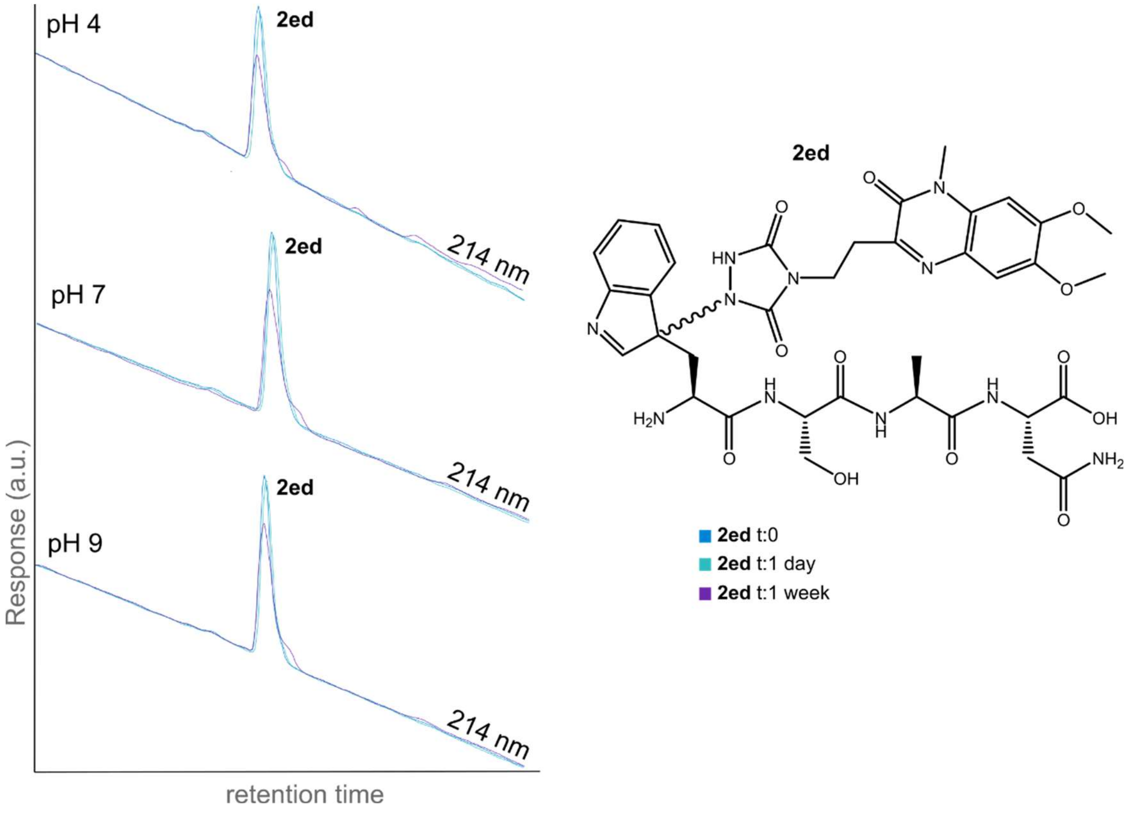

Figure 2.2.3.2 HPLC chromatogram (214 nm) overlay graphs of the peptide conjugate **2ed** in different PBS buffers at pH 4, pH 7 and pH 9. The conjugate was left at room temperature and at several time points a sample was taken and a chromatogram recorded.

The HPLC 214 nm chromatogram peak intensity for conjugate **2ed** demonstrated for all pH conditions a very similar behavior. Almost no change in peak intensity after 1 day at room temperature and a small but noticeable reduction (less than 20%) after 1 full week.

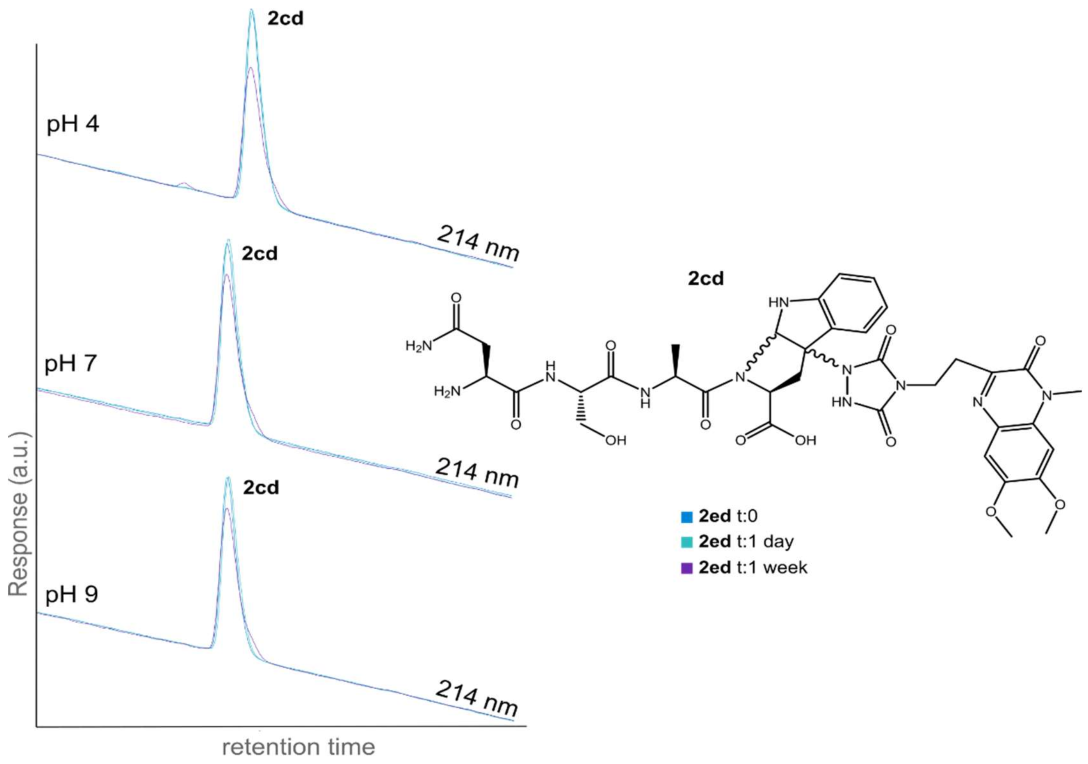

Figure 2.2.3.3 HPLC chromatogram (214 nm) overlay graphs of the peptide conjugate **2cd** in different PBS buffers at pH 4, pH 7 and pH 9. The conjugate was left at room temperature and at several time points a sample was taken and a chromatogram recorded.

The HPLC 214 nm chromatogram peak intensity for conjugate **2cd** demonstrated similar behavior as conjugate **2ed** at pH 4. Almost no change in peak intensity after 1 day at room temperature and a noticeable but still limited (less than 20%) reduction after 1 full week. For the experiments at pH 7 and pH 9 there is a difference in the peak intensity of **2cd**, after 1 day the peak intensity is almost identical to the intensity at the starting point. After 1 week at pH 7 and pH 9 there is only a small reduction of the peak intensity.

In a following experiment the peptide conjugate stability was tested in serum. Peptide conjugates **2ed** and **2cd** were prepared according to the procedure presented in section 1.3 of this supplementary information with a higher excess of TAD compound (10 equivalents). Conjugates **2ed** and **2cd** were purified using HPLC and lyophilized. Purified conjugates **2ed** and **2cd** were then dissolved in water (3 mM) and diluted with 20% human serum in PBS pH 7. The samples were placed in a thermoshaker at 37 °C and shaken for 24 h. HPLC samples were recorded at the start and after 24h, the presence of the DMEQ-group allows to follow the peptide conjugate product peaks at 370 nm.

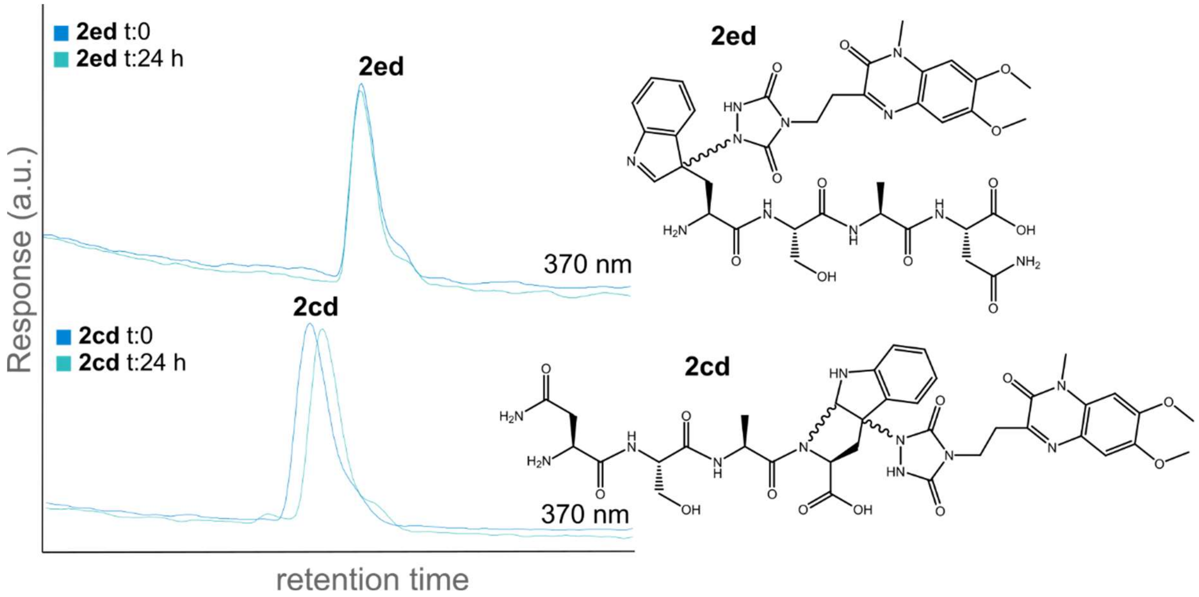

Figure 2.2.3.4 HPLC chromatogram (370 nm) overlay graphs of the peptide conjugates **2ed** (top) and **2cd** (bottom) in 20% human serum in PBS pH7. The mixture was shaken for 24 hours at 37°C and HPLC samples were recorded at the starting point and after 24 hours.

The peak intensity for the fluorescent peptide conjugates at 370 nm was almost not changed after 24 h incubation in human serum at 37 °C.

2.2.4 Peptide conjugation yield

To examine the TAD tryptophan conjugation reaction yield, several experiments were performed with a varying number of TAD equivalents. Peptide **1e** was dissolved in water (at 3 mM), and this was diluted (1/10) in PBS pH 4. Subsequently, we added different amounts of TAD compound **2c** in MeCN. Compound **2c** was prepared as described in section 1.2 of this supporting information. The reaction mixture of the different experiments was analysed via HPLC and the results are presented in figure 2.2.4.1.

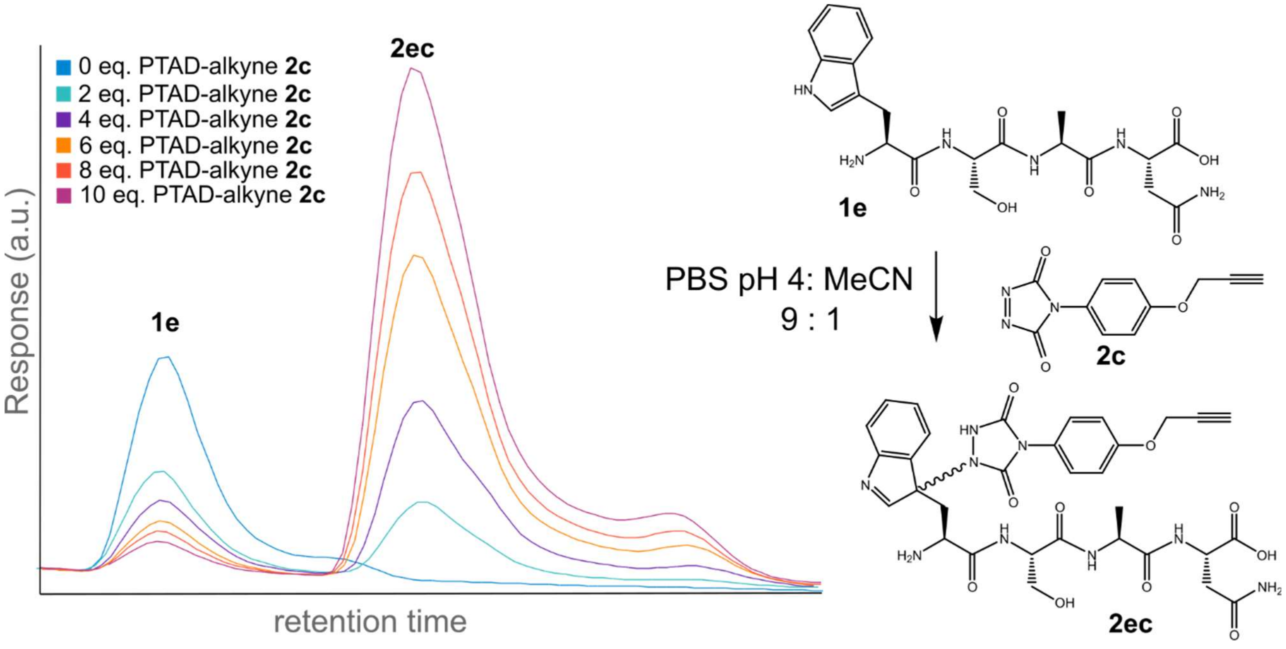

Figure 2.2.4.1 HPLC chromatogram (214 nm) overlay graphs of the peptide conjugation reactions with **1e** and different amounts of **2c** (left). Schematic representation of the reaction of peptide **1e** with **2c**.

The data in figure 2.2.4.1 demonstrate that 10 equivalents of TAD compound convert over 90% of the starting peptide material based on the HPLC peak integration.

2.3 MALDI TOF/TOF data of peptide conjugates

2.3.1 pH dependency of the intramolecular Tyr vs Trp competition: experimental and software setup

For peptides containing both a tyrosine and tryptophan residue, conjugation reactions were performed with different TAD compounds (TAD-propanol **2b,** PTAD-alkyne **2c**). Samples were analyzed via MALDI-TOF/TOF analysis on a 4800+ MALDI TOF/TOF analyser (Applied Biosystems) after HPLC purification of the peptide conjugate peaks. Peptide conjugations were performed as described in the general protocol for peptide conjugation (section 1.3).

Peptides **1i** and **1j** were used in conjugation reactions with TAD-propanol **2b** or PTAD-alkyne **2c**, conjugation reactions were done in 10x PBS buffers at pH 3, 4, 5, 6, 7. Peptide **1k** was used in conjugation reactions with TAD-propanol **2b** or PTAD-alkyne **2c**, conjugation reactions were done in 10 x PBS buffer pH 3 or pH 7. Finally peptide **1l** was used in a conjugation reaction with TAD-propanol **2b** in H_2O.

The TAD-peptide conjugate precursor ions were fragmented using the 1 kV positive method and without gas in the collision chamber (CID off). These settings are post source decay (PSD) MS/MS settings. Using these soft MS/MS settings, still a lot of TAD-modification on tryptophan falls off during MS/MS analysis (vide infra).

The resulting MS/MS spectra are annotated using the mMass software, TAD modifications are programmed in the software both for tyrosine and tryptophan residues (figure 2.3.1.1).

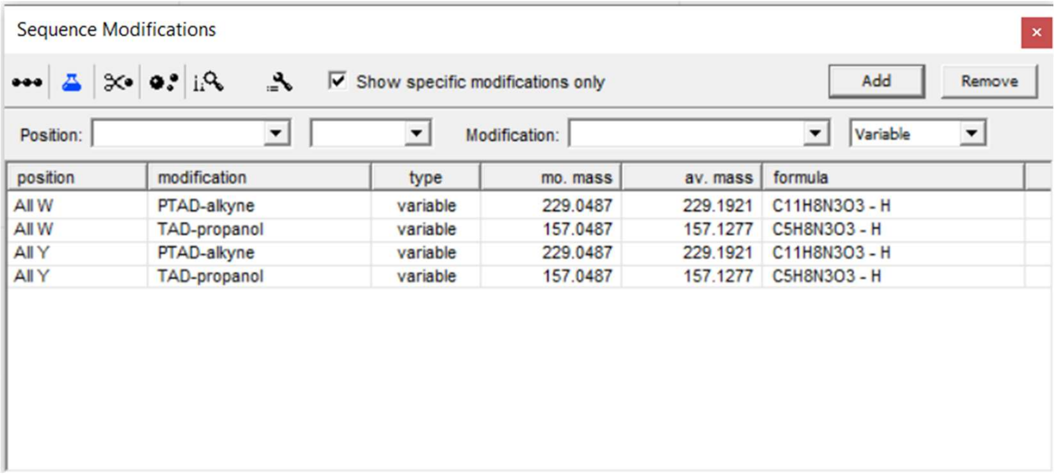

Figure 2.3.1.1 Print screen of the TAD-propanol **2b** and PTAD-alkyne **2c** modifications that are taken into account as variable modifications on both tryptophans and tyrosines. A variable modification means that it can either be present or not present.

Subsequently all possible fragment ions are calculated for the fragment ions of choice, which are M, a, b and y ions with possible loss of water and ammonia and addition of water (figure 2.3.1.2). Additionally a maximum charge can be indicated, 3 was selected since typically only 1+ and 2+ ions are found in MALDI TOF/TOF spectra.

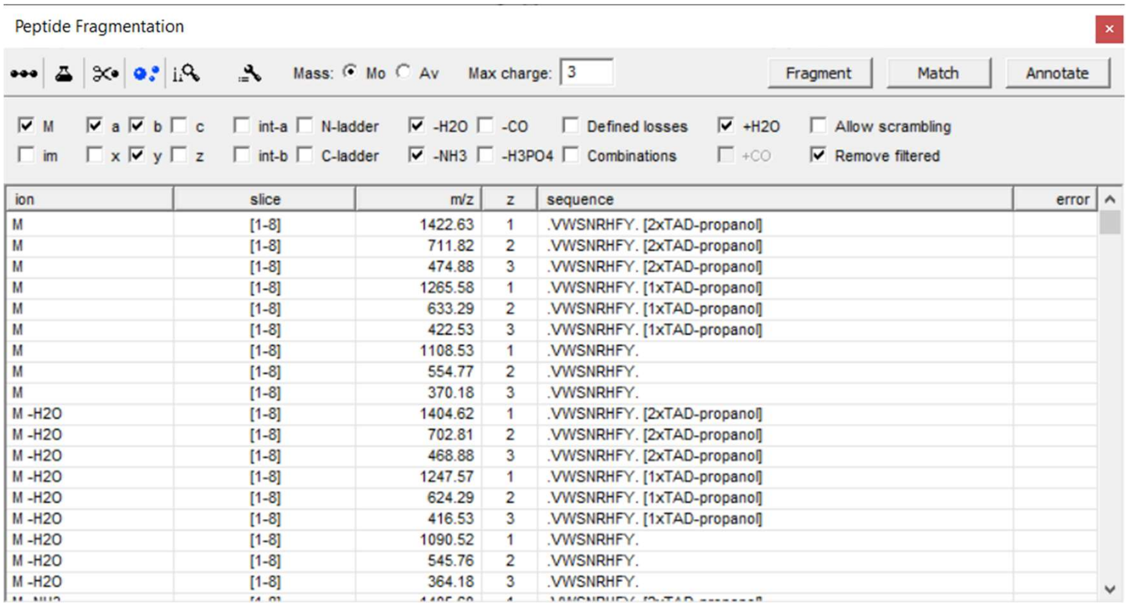

ion	slice	m/z	z	sequence	error
M	[1-8]	1422.63	1	.VWSNRHFY. [2xTAD-propanol]	
M	[1-8]	711.82	2	.VWSNRHFY. [2xTAD-propanol]	
M	[1-8]	474.88	3	.VWSNRHFY. [2xTAD-propanol]	
M	[1-8]	1265.58	1	.VWSNRHFY. [1xTAD-propanol]	
M	[1-8]	633.29	2	.VWSNRHFY. [1xTAD-propanol]	
M	[1-8]	422.53	3	.VWSNRHFY. [1xTAD-propanol]	
M	[1-8]	1108.53	1	.VWSNRHFY.	
M	[1-8]	554.77	2	.VWSNRHFY.	
M	[1-8]	370.18	3	.VWSNRHFY.	
M -H2O	[1-8]	1404.62	1	.VWSNRHFY. [2xTAD-propanol]	
M -H2O	[1-8]	702.81	2	.VWSNRHFY. [2xTAD-propanol]	
M -H2O	[1-8]	468.88	3	.VWSNRHFY. [2xTAD-propanol]	
M -H2O	[1-8]	1247.57	1	.VWSNRHFY. [1xTAD-propanol]	
M -H2O	[1-8]	624.29	2	.VWSNRHFY. [1xTAD-propanol]	
M -H2O	[1-8]	416.53	3	.VWSNRHFY. [1xTAD-propanol]	
M -H2O	[1-8]	1090.52	1	.VWSNRHFY.	
M -H2O	[1-8]	545.76	2	.VWSNRHFY.	
M -H2O	[1-8]	364.18	3	.VWSNRHFY.	

Figure 2.3.1.2 Print screen of the calculated peptide fragmentation.

The calculated peptide fragments are matched with the peaks in the MS/MS spectra. The tolerance for deviation between the calculated and detected fragments can be indicated. In this work we selected 0.5 Da as tolerance. The matches are detected fragments that are within the tolerance from the calculated fragment. The error of all the matches is plotted as function of the m/z. We found that for experiments on the MALDI-TOF/TOF with charge +1, the error typically increases slightly from values below 0.1 Da for 300 m/z to around 0.3 Da for 1000 m/z and above. If two calculated fragments incidentally match with one detected fragment the error plot is helpful to select the most suitable fragment ion. In figure 2.3.1.3 two calculated fragments match to a detected fragment around 400 m/z.

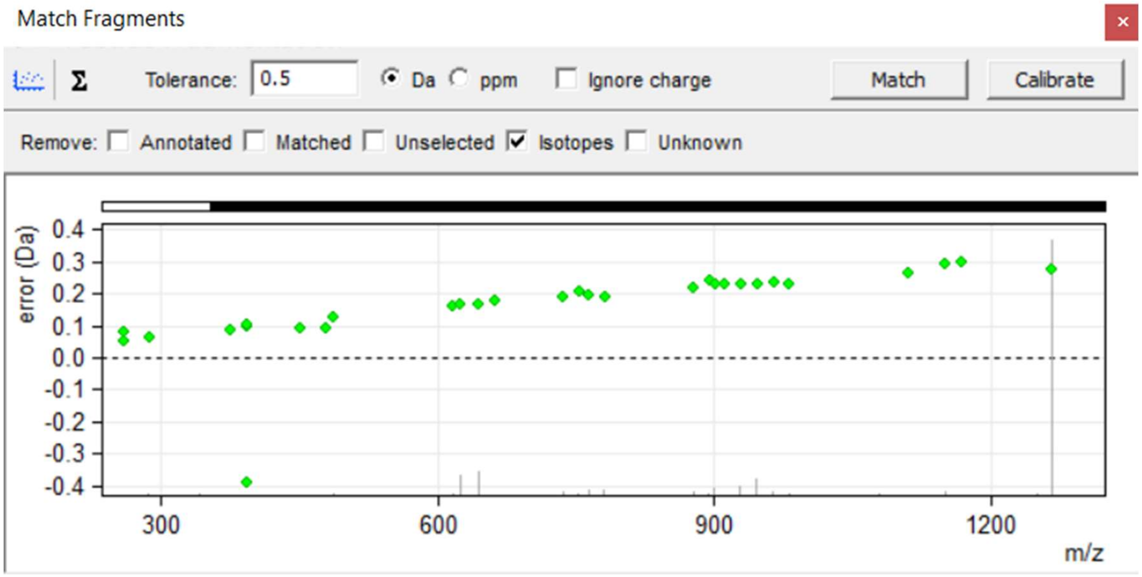

Figure 2.3.1.3 Print screen of the error vs m/z plot for the matched peptide fragments.

The annotated MS/MS spectra are provided along with the chemical structure of the peptide with the detected fragments indicated. a and b ions are displayed below the structure and start from the N-terminus. y ions are displayed above the structure and start from the C-terminus.

The analysis of these MS/MS data demonstrates that the TAD modification shifts from being selective on tryptophan, in conditions of pH 4 and below not a single fragment ion with a TAD modification on tyrosine was detected, to both modification on tyrosine and tryptophan in higher pH conditions (pH 5 and above). Additionally the data show that the TAD modification on tryptophan is not stable during the MALDI TOF/TOF analysis. This is shown by the large abundance of the ion corresponding to the complete peptide without TAD modification and this effect is seen in experiments that were done in pH 4 and below (tryptophan conjugation). Due to the fact that the TAD modification on tryptophan is not stable during MALDI TOF/TOF analysis it seems that the TAD modification is selectively taking place on the tyrosine residue in conditions of pH 5 and above. This is however not the case as we have shown that both tyrosine and tryptophan will react at pH 7, and sometimes TAD modified peptide fragments encompassing the tryptophan residue are still observed in MALDI TOF/TOF analysis when conjugation was done at pH 7.

2.3.2 MS/MS spectra of unmodified peptides

Val-Trp-Ser-Asn-Arg-His-Phe-Tyr-OH (VWSNRHFY, 1i). MS (MALDI-TOF) precursor ion: m/z 1108.8 (calcd $[M+H]^+$ 1108.5)

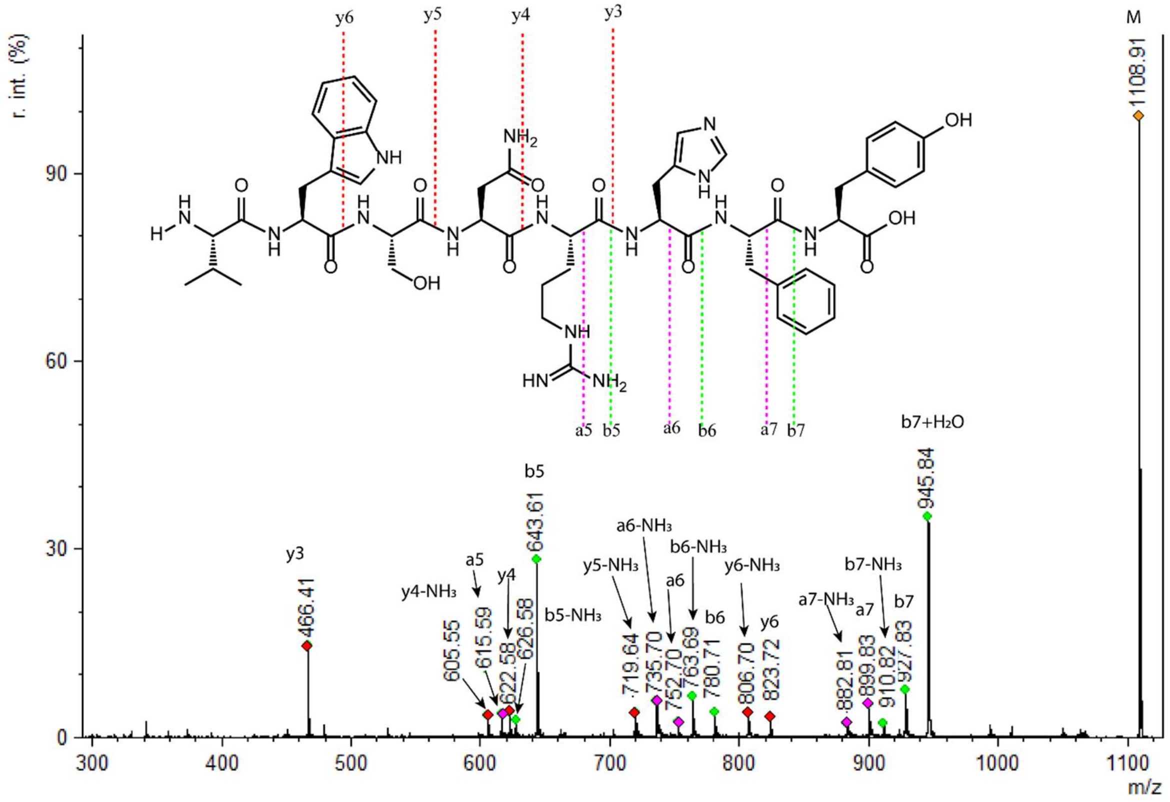

Figure 2.3.2.1 MALDI TOF/TOF spectrum of unmodified peptide **1i**.

Val-Tyr-Ser-Asn-Arg-His-Phe-Trp-OH (VYSNRHFW, 1j). MS (MALDI-TOF) precursor ion: m/z 1108.8 (calcd $[M+H]^+$ 1108.5)

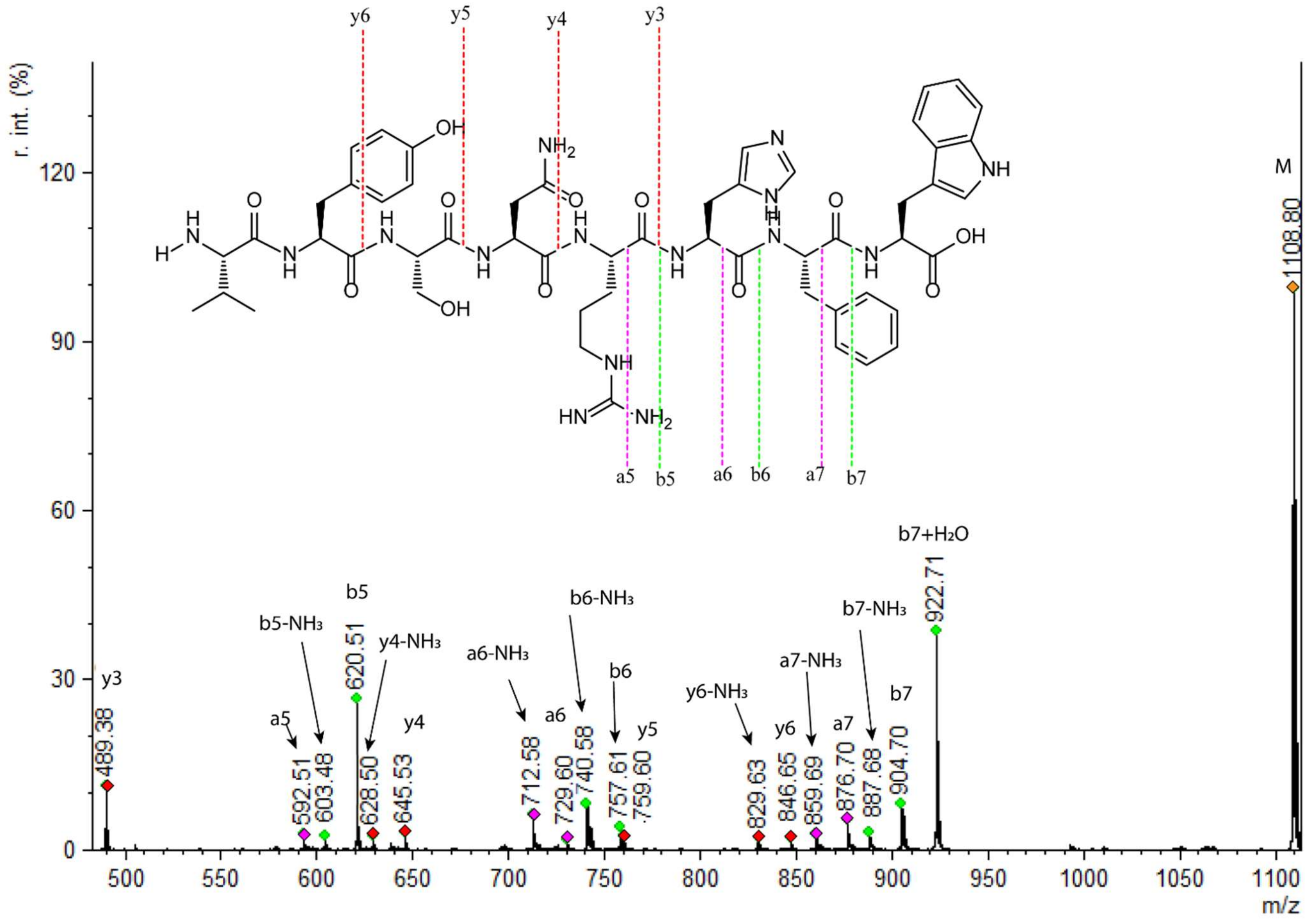

Figure 2.3.2.2 MALDI TOF/TOF spectrum of unmodified peptide **1j**.

Val-Trp-Ser-Gln-Lys-Arg-His-Phe-Gly-Tyr-OH (VWSQKRHFGY, 1k). MS (MALDI-TOF) precursor ion: m/z 1307.6 (calcd [M+H]+ 1307.7)

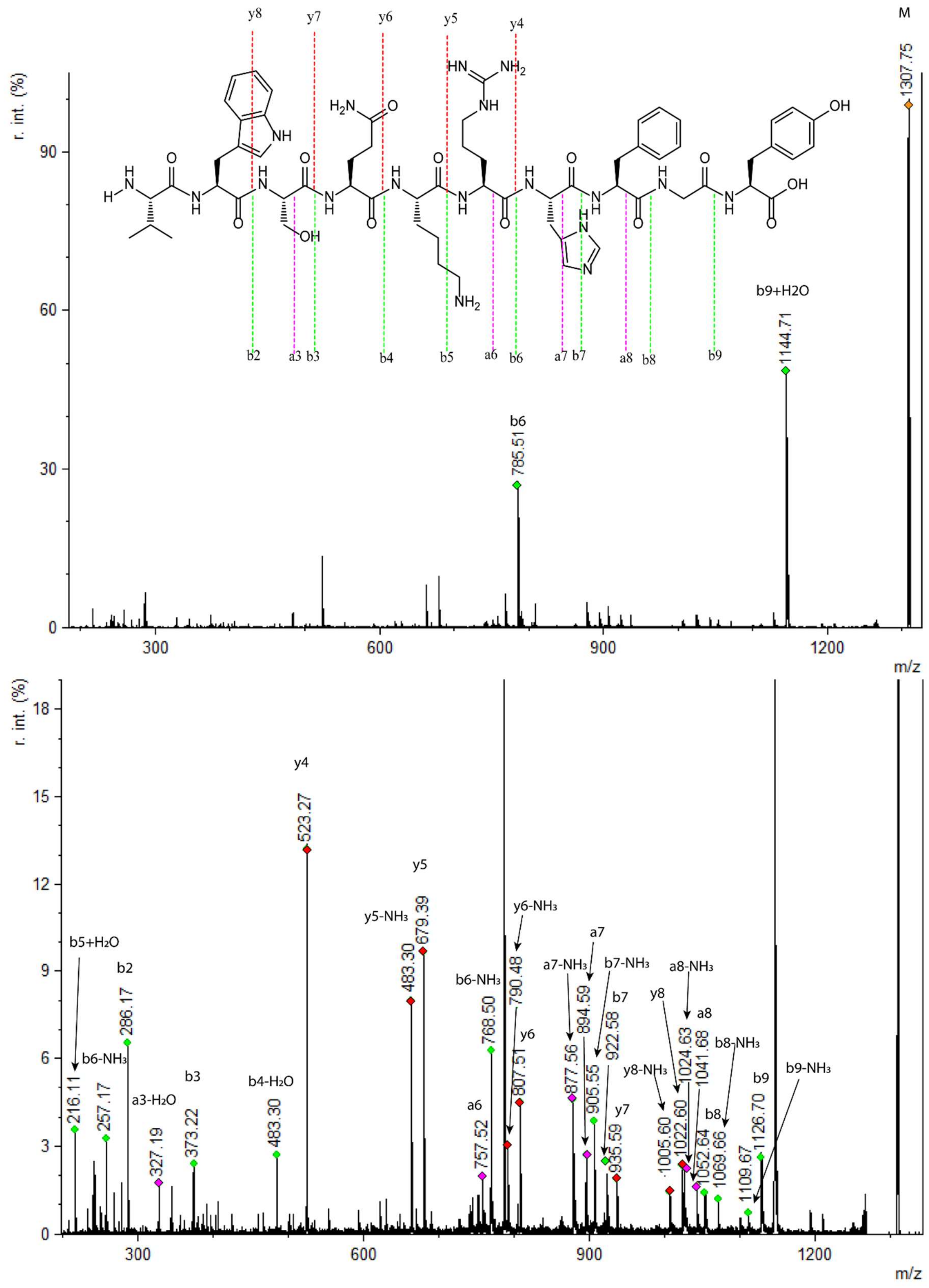

Figure 2.3.2.3 MALDI TOF/TOF spectrum of unmodified peptide **1k.**

Lys-Asp-Tyr-Trp-Glu-Cys-Ala-OH (KDYWECA, 1l). MS (MALDI-TOF) precursor ion: m/z 914.4 (calcd $[M+H]^+$ 914.4)

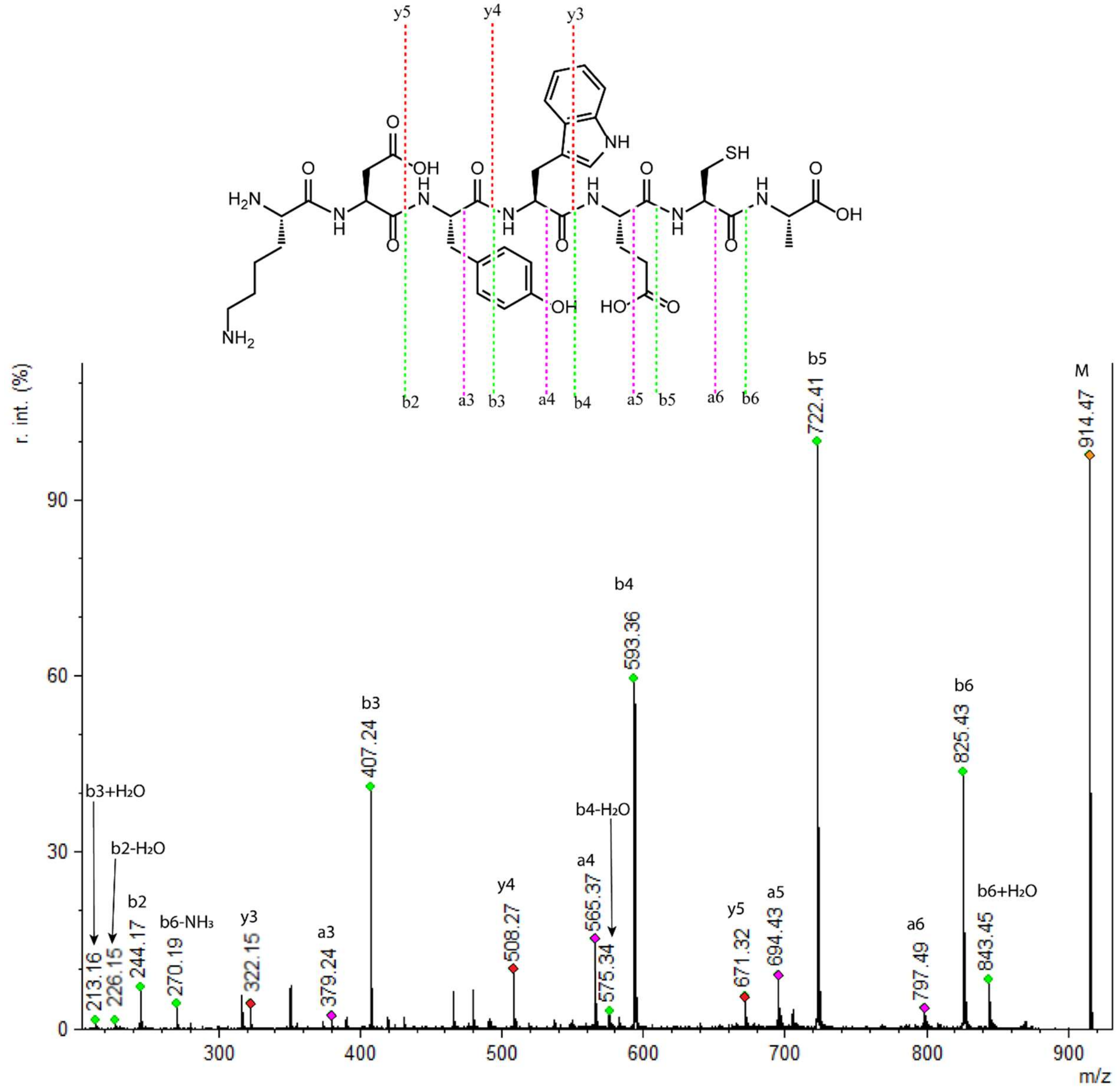

Figure 2.3.2.4 MALDI TOF/TOF spectrum of unmodified peptide **1l**.

2.3.3 MS/MS spectra of modified peptides

Conjugation product of Val-Trp-Ser-Asn-Arg-His-Phe-Tyr-OH (VWSNRHFY, 1i) with TAD-propanol 2b (2ib). MS (MALDI-TOF) precursor ion: m/z 1265.8 (calcd $[M+H]^+$ 1265.6)

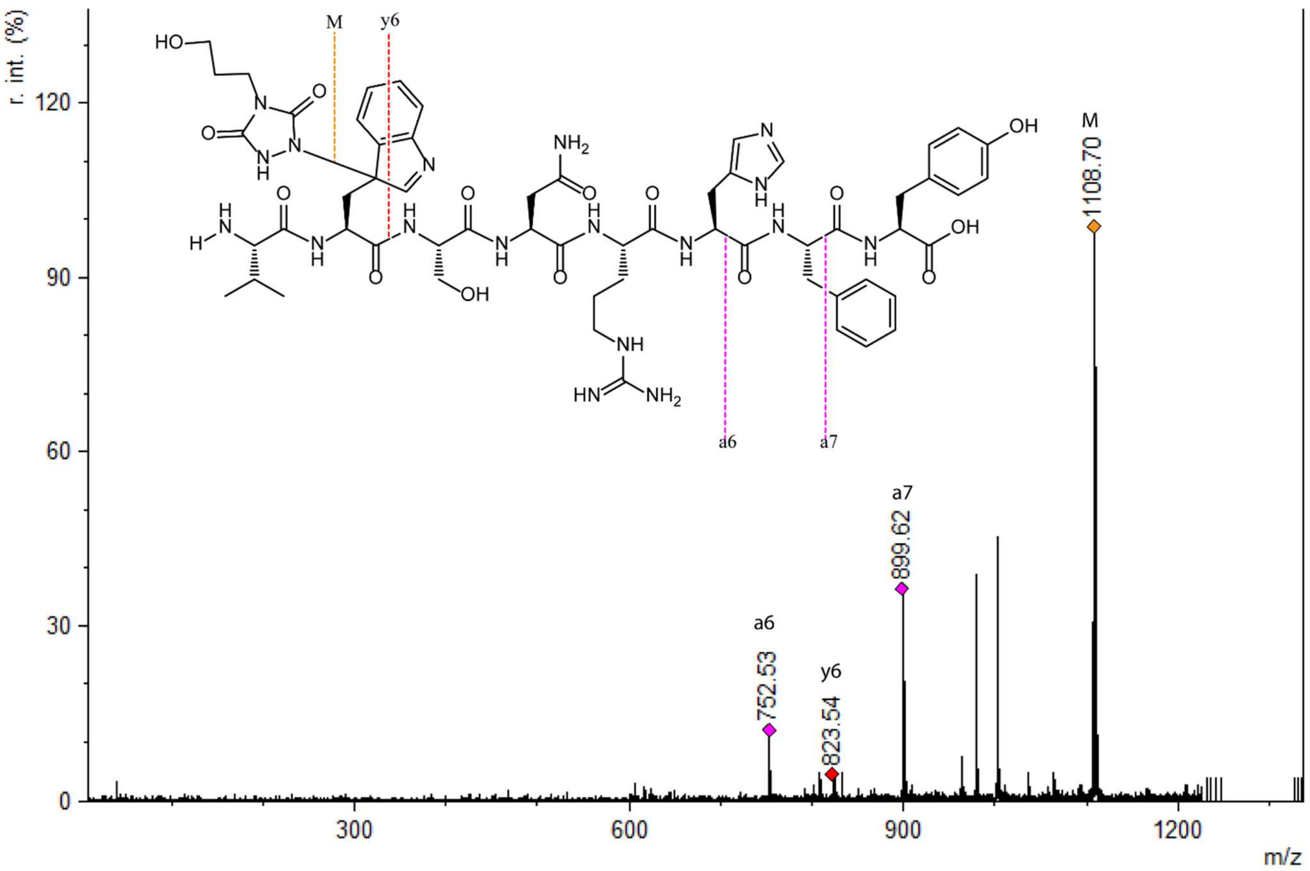

Figure S2.3.3.1 MALDI-TOF/TOF spectrum of peptide **1i** conjugated with **2b** (precursor ion: 1265.8 Da). Chemical structure of peptide **1i** with detected fragment ions is shown. Fragments with "*" are TAD modified fragments. Conjugation reaction was performed in 10x PBS pH 3.

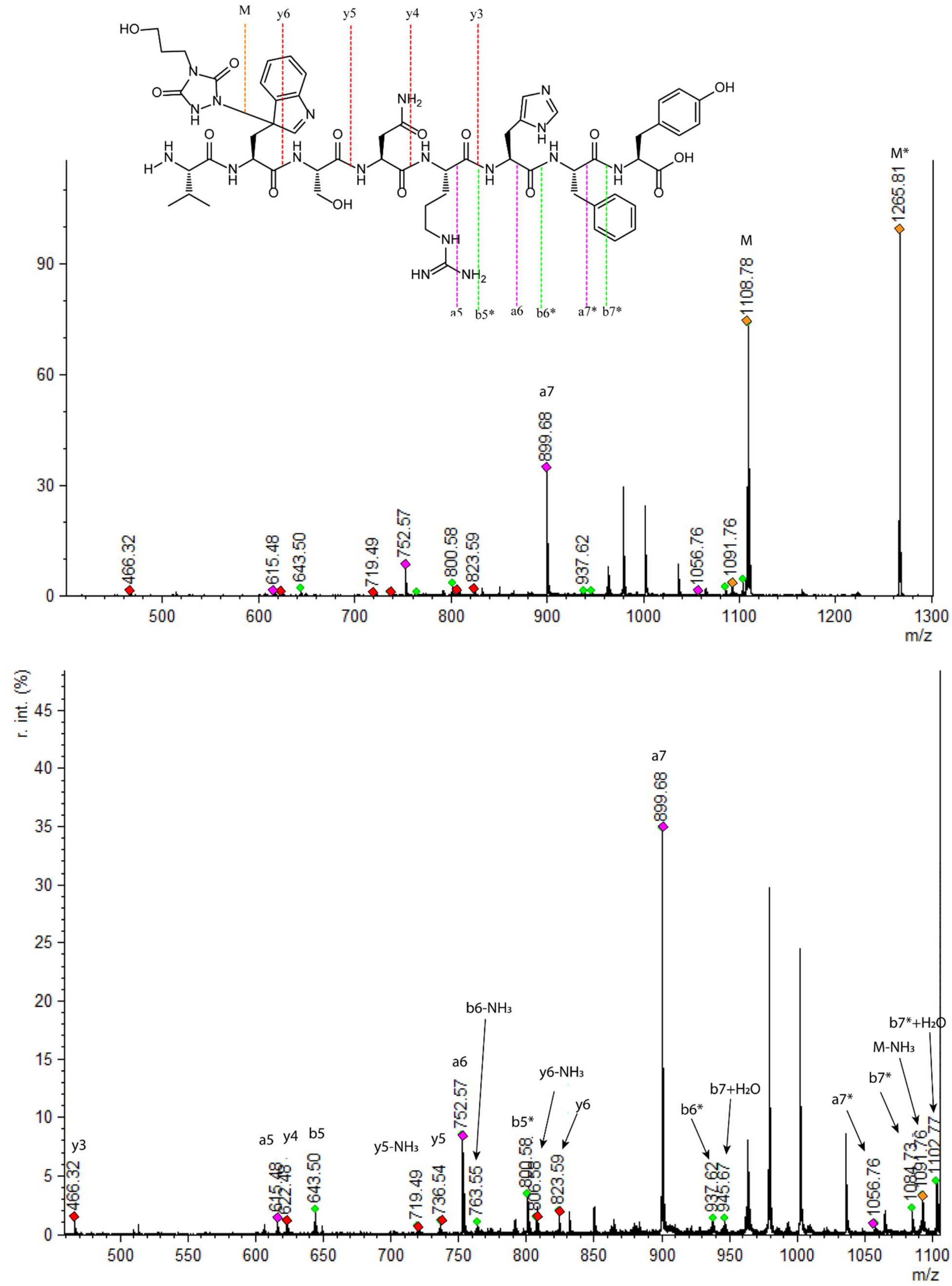

Figure S2.3.3.2 MALDI-TOF/TOF spectrum of peptide **1i** conjugated with **2b** (precursor ion: 1265.8 Da). Chemical structure of peptide **1i** with detected fragment ions is shown. Fragments with “*” are TAD modified fragments. Conjugation reaction was performed in 10x PBS pH 4.

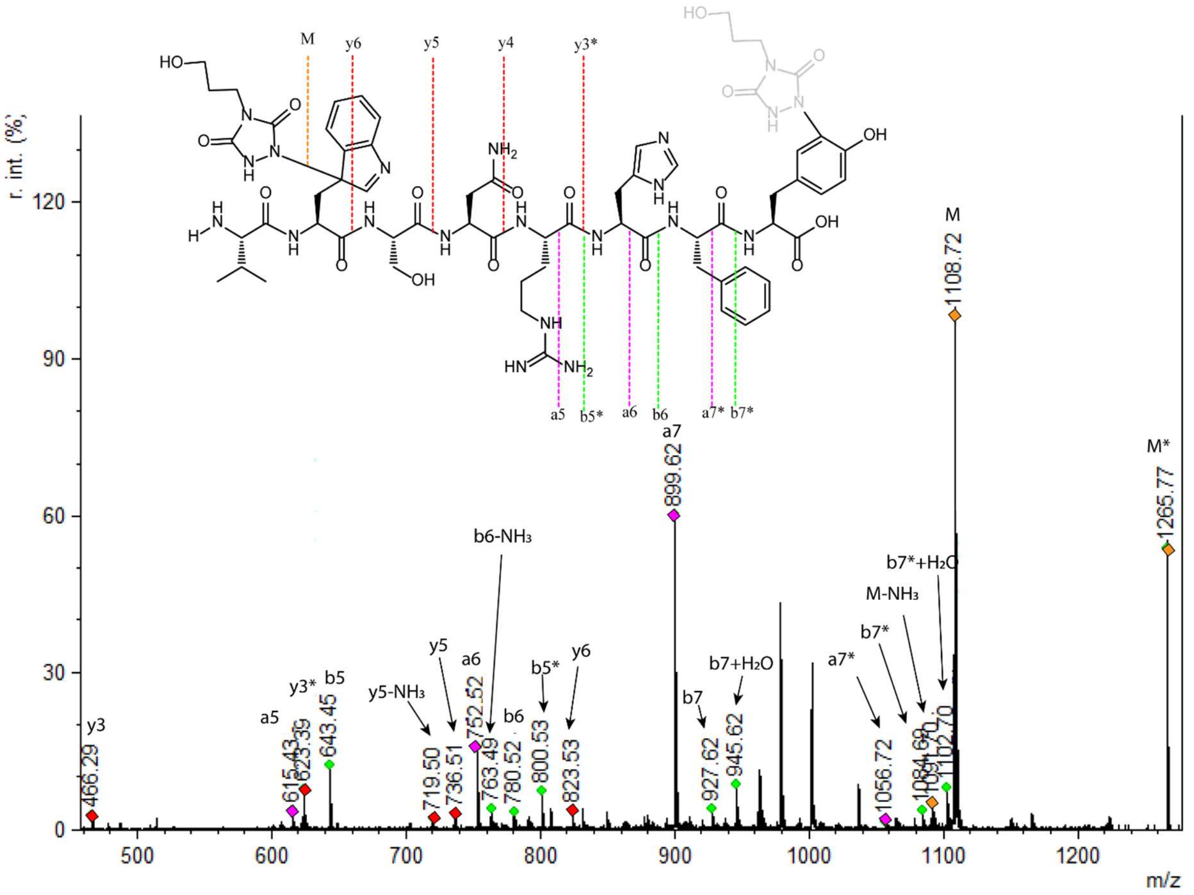

Figure S2.3.3.3 MALDI-TOF/TOF spectrum of peptide **1i** conjugated with **2b** (precursor ion: 1265.8 Da). Chemical structure of peptide **1i** with detected fragment ions is shown. Fragments with "*" are TAD modified fragments. Conjugation reaction was performed in 10x PBS pH 5.

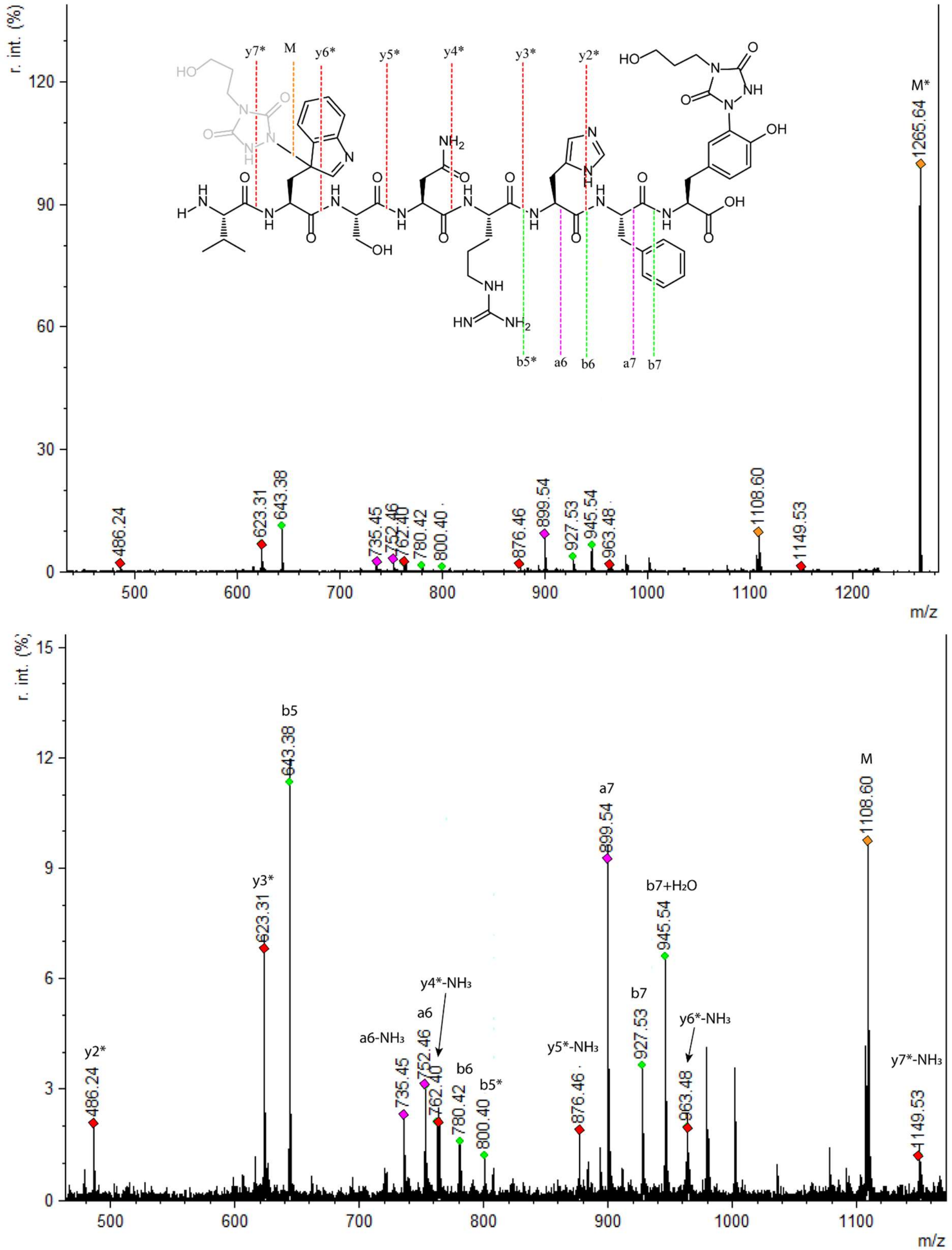

Figure S2.3.3.4 MALDI-TOF/TOF spectrum of peptide **1i** conjugated with **2b** (precursor ion: 1265.8 Da). Chemical structure of peptide **1i** with detected fragment ions is shown. Fragments with "*" are TAD modified fragments. Conjugation reaction was performed in 10x PBS pH 6.

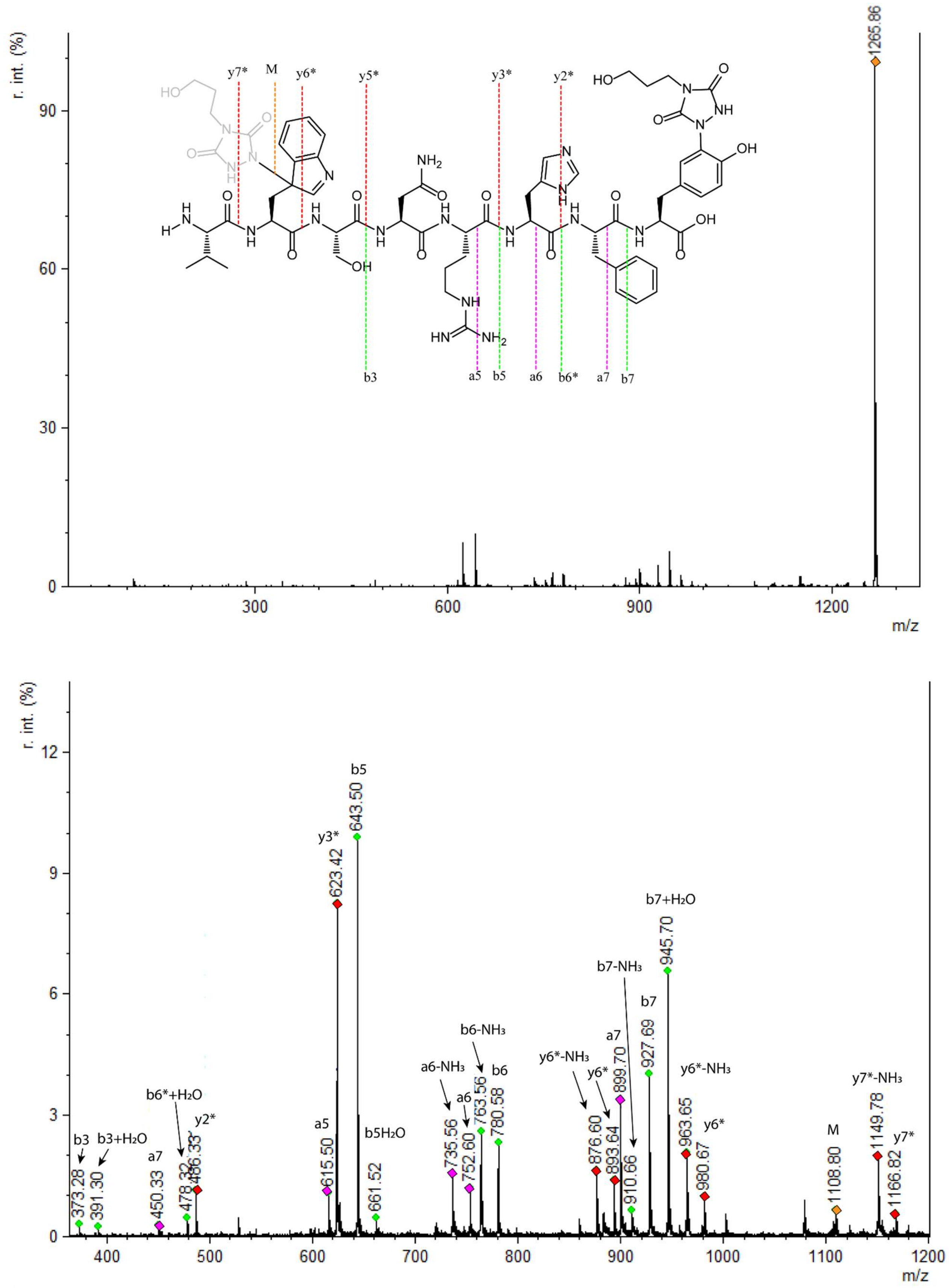

Figure S2.3.3.5 MALDI-TOF/TOF spectrum of peptide **1i** conjugated with **2b** (precursor ion: 1265.8 Da). Chemical structure of peptide **1i** with detected fragment ions is shown. Fragments with "*" are TAD modified fragments. Conjugation reaction was performed in 10x PBS pH 7.

Conjugation product of Val-Trp-Ser-Asn-Arg-His-Phe-Tyr-OH (VWSNRHFY, 1i) with PTAD-alkyne 2c (2ic). MS (MALDI-TOF) precursor ion: m/z 1337.8 (calcd $[M+H]^+$ 1337.5)

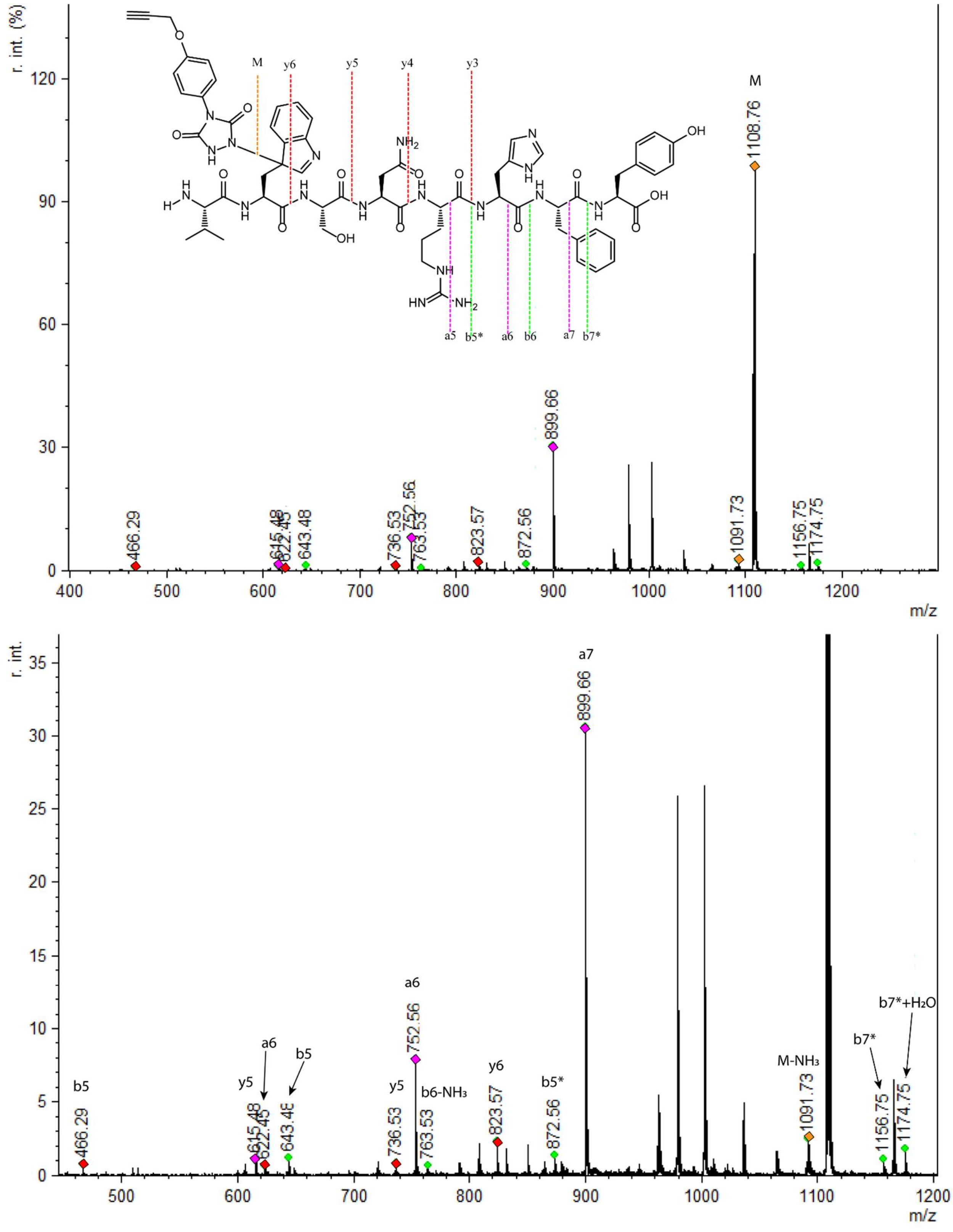

Figure S2.3.3.6 MALDI-TOF/TOF spectrum of peptide **1i** conjugated with **2c** (precursor ion: 1337.8 Da). Chemical structure of peptide **1i** with detected fragment ions is shown. Fragments with "*" are TAD modified fragments. Conjugation reaction was performed in 10x PBS pH 3.

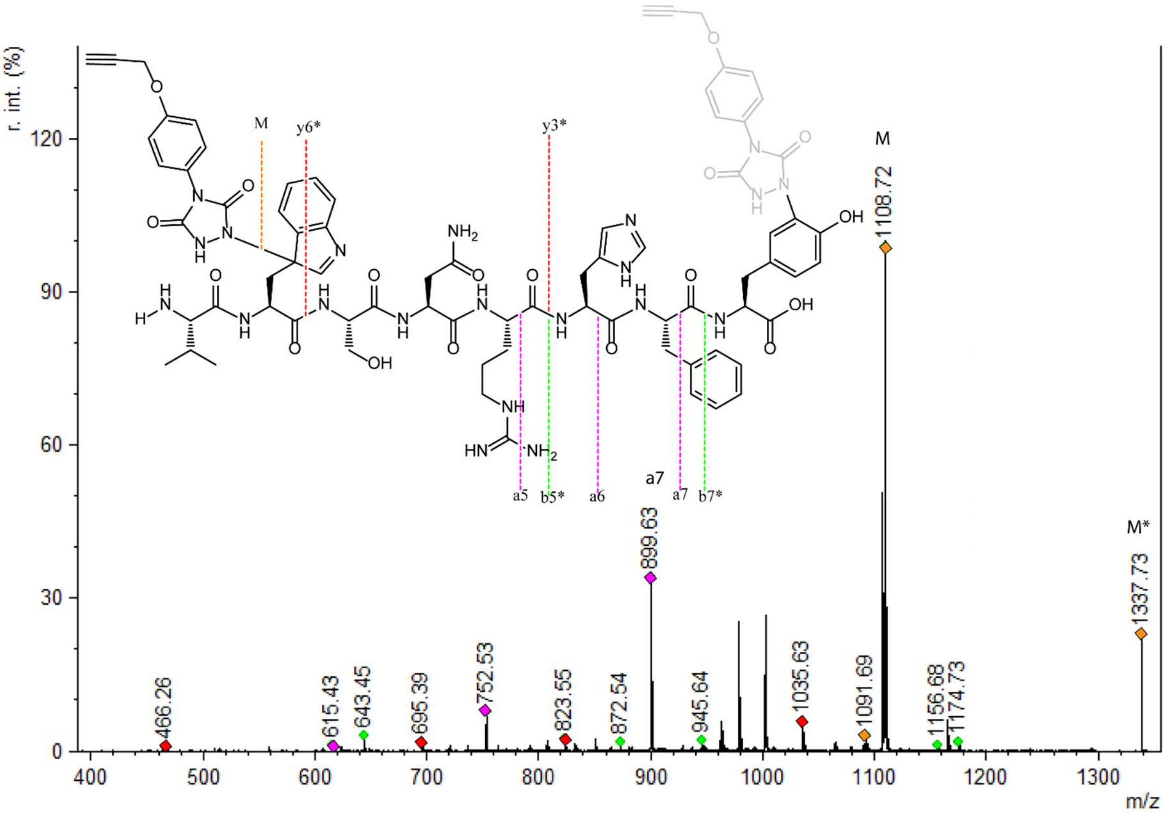

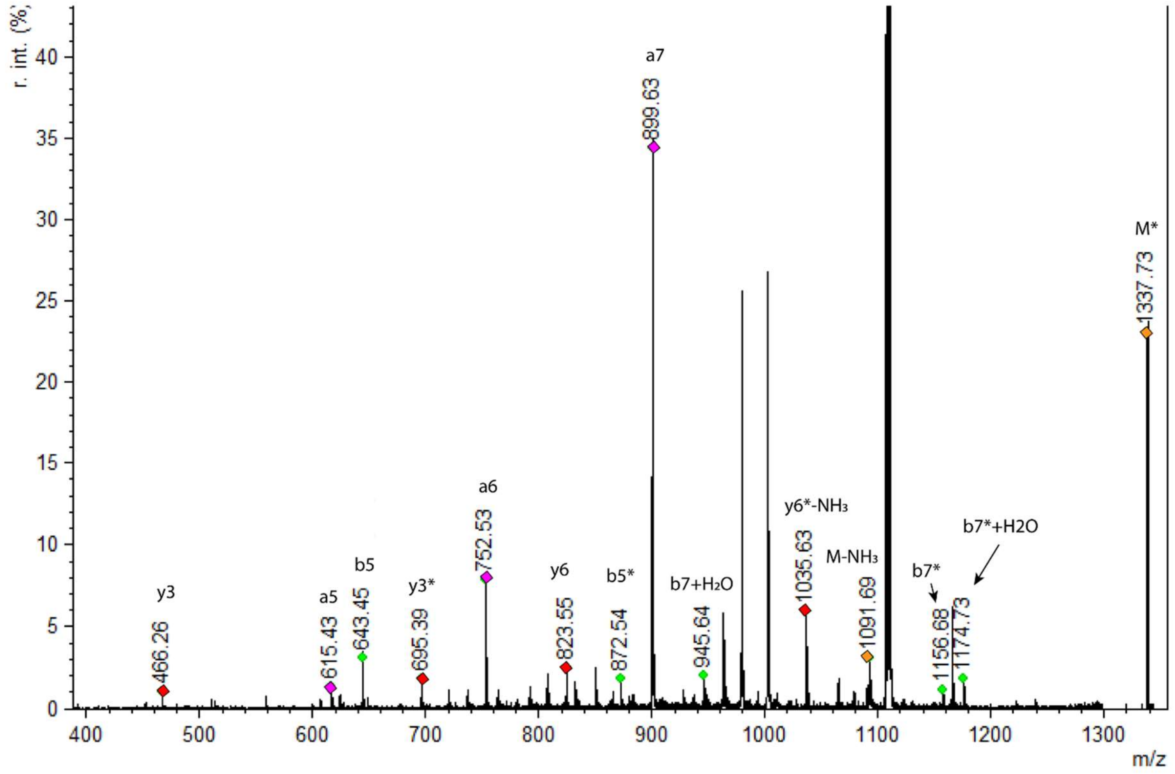

Figure S2.3.3.7 MALDI-TOF/TOF spectrum of peptide **1i** conjugated with **2c** (precursor ion: 1337.8 Da). Chemical structure of peptide **1i** with detected fragment ions is shown. Fragments with "*" are TAD modified fragments. Conjugation reaction was performed in 10x PBS pH 4.

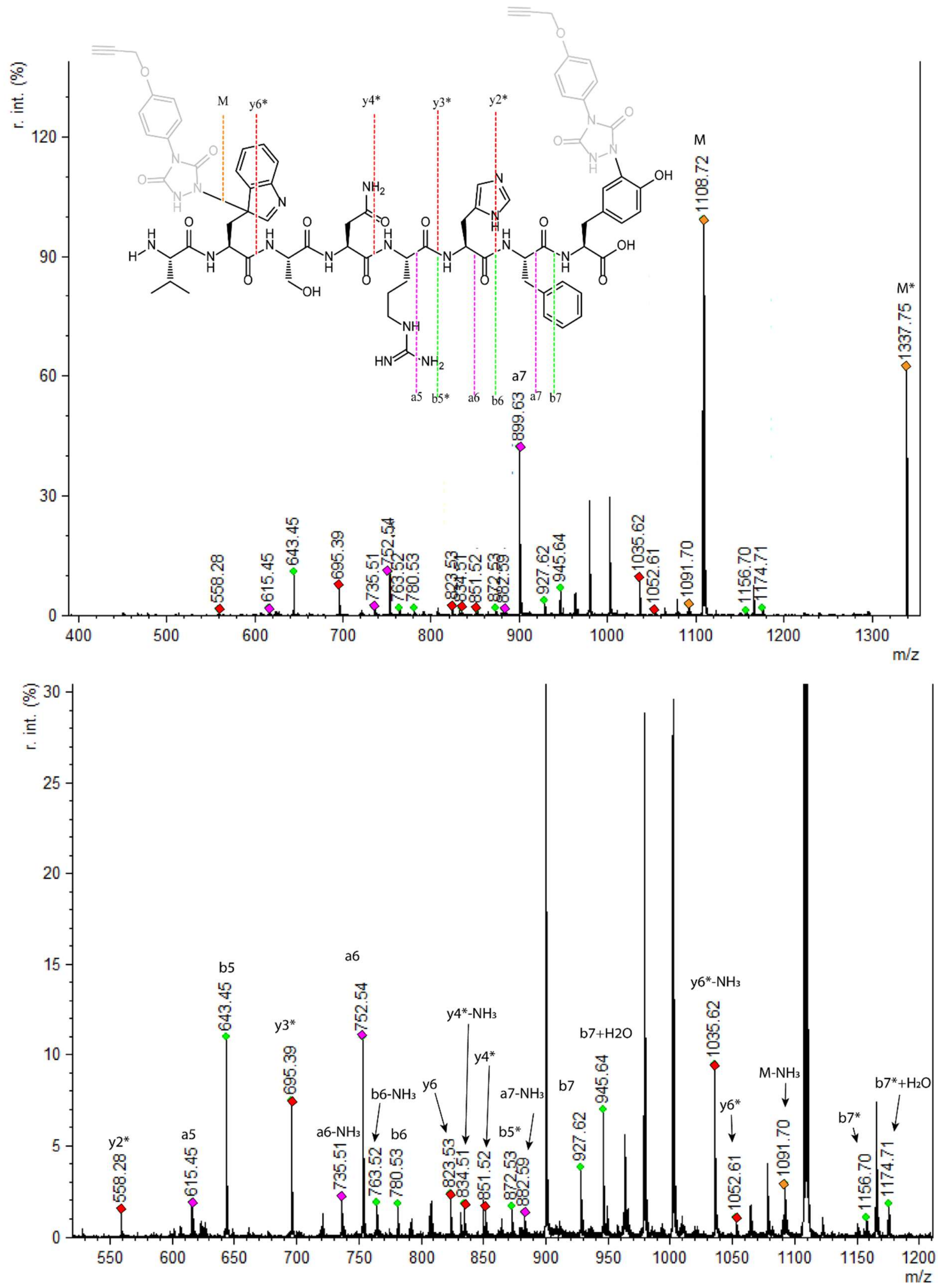

Figure S2.3.3.8 MALDI-TOF/TOF spectrum of peptide **1i** conjugated with **2c** (precursor ion: 1337.8 Da). Chemical structure of peptide **1i** with detected fragment ions is shown. Fragments with “*” are TAD modified fragments. Conjugation reaction was performed in 10x PBS pH 5.

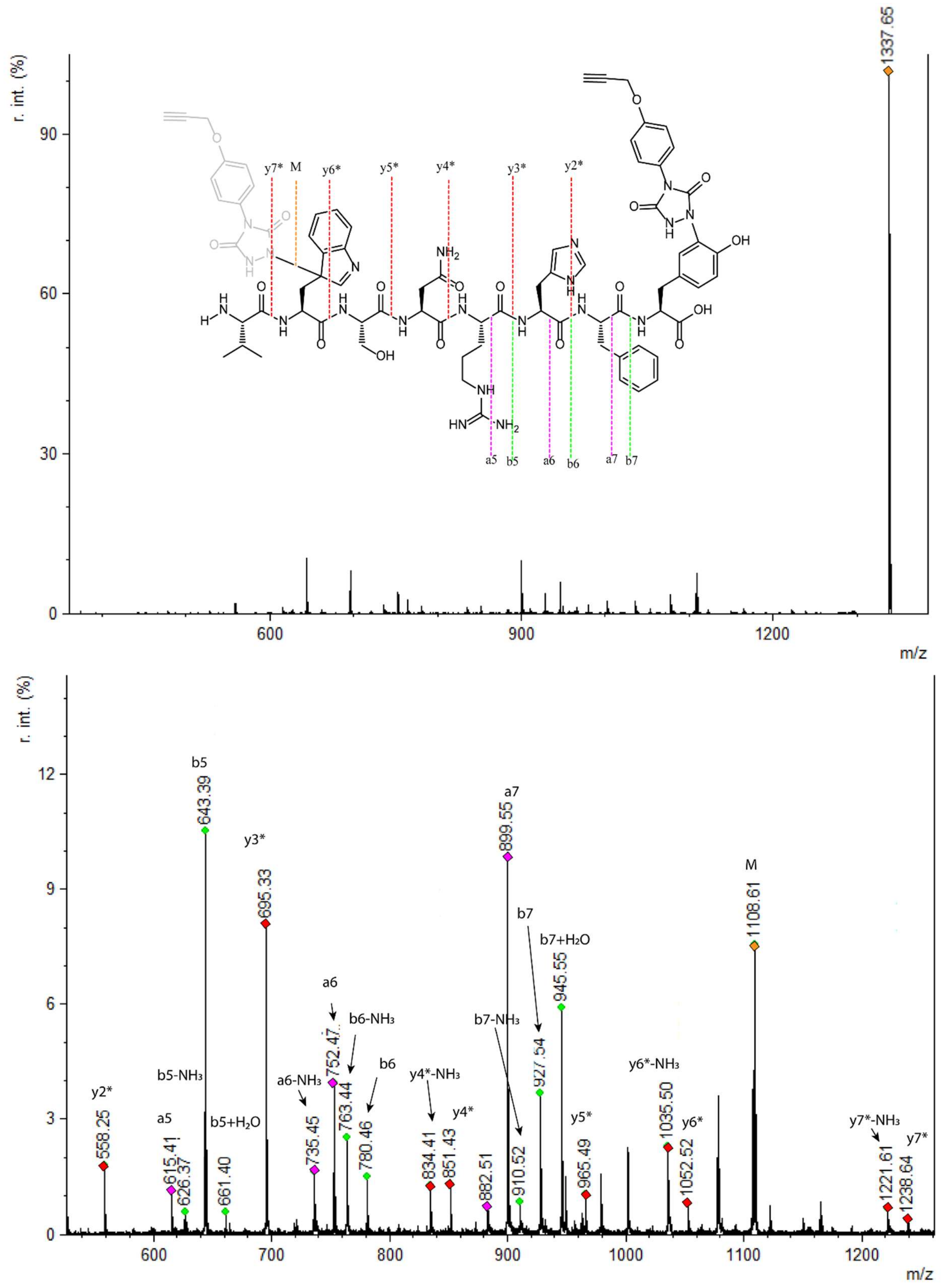

Figure S2.3.3.9 MALDI-TOF/TOF spectrum of peptide **1i** conjugated with **2c** (precursor ion: 1337.8 Da). Chemical structure of peptide **1i** with detected fragment ions is shown. Fragments with "*" are TAD modified fragments. Conjugation reaction was performed in 10x PBS pH 6.

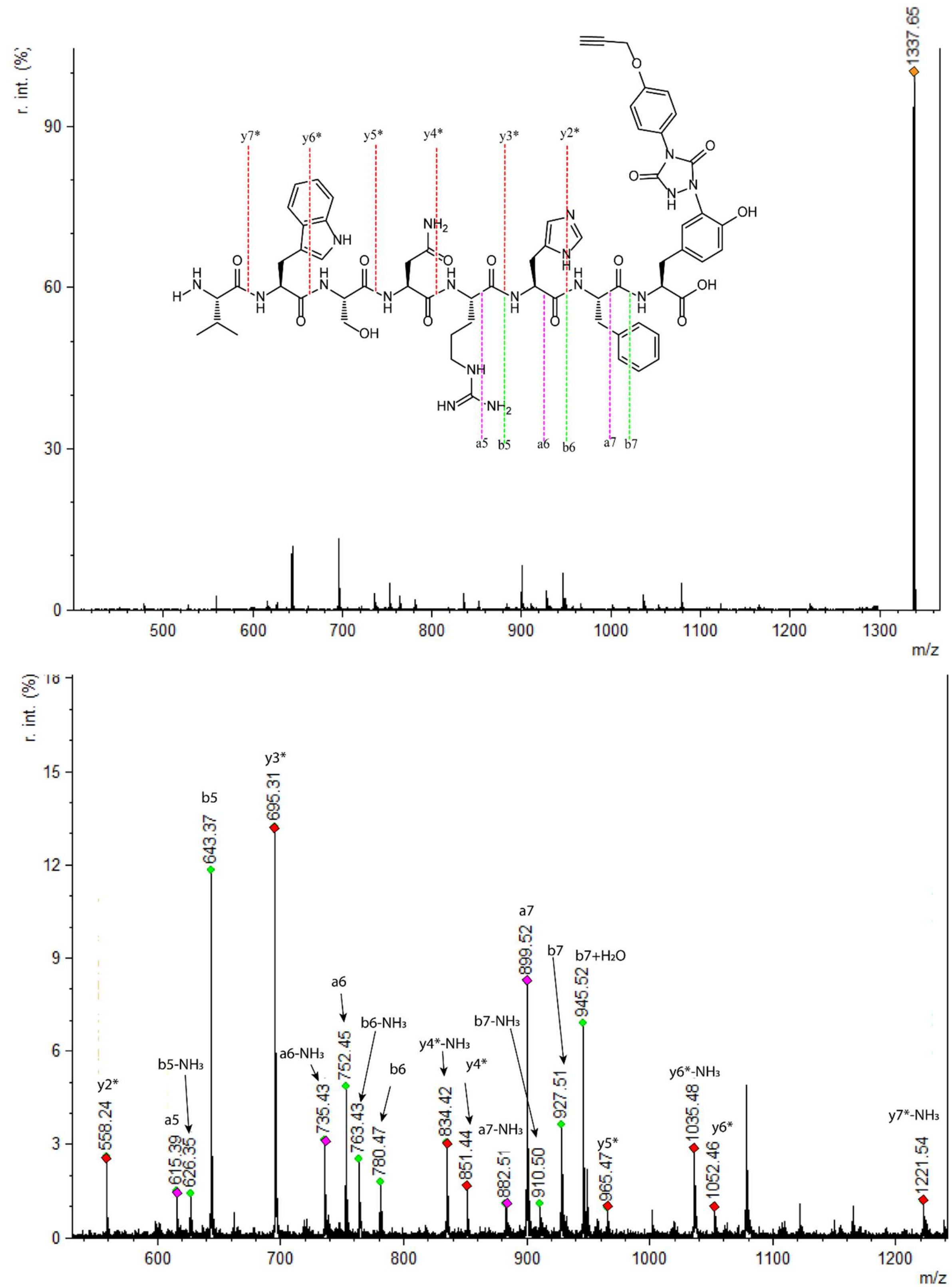

Figure S2.3.3.10 MALDI-TOF/TOF spectrum of peptide **1i** conjugated with **2c** (precursor ion: 1337.8 Da). Chemical structure of peptide **1i** with detected fragment ions is shown. Fragments with "*" are TAD modified fragments. Conjugation reaction was performed in 10x PBS pH 7.

Conjugation product of Val-Tyr-Ser-Asn-Arg-His-Phe-Trp-OH (VYSNRHFW, 1j) with TAD-propanol 2b (2jb). MS (MALDI-TOF) precursor ion: m/z 1265.8 (calcd $[M+H]^+$ 1265.6)

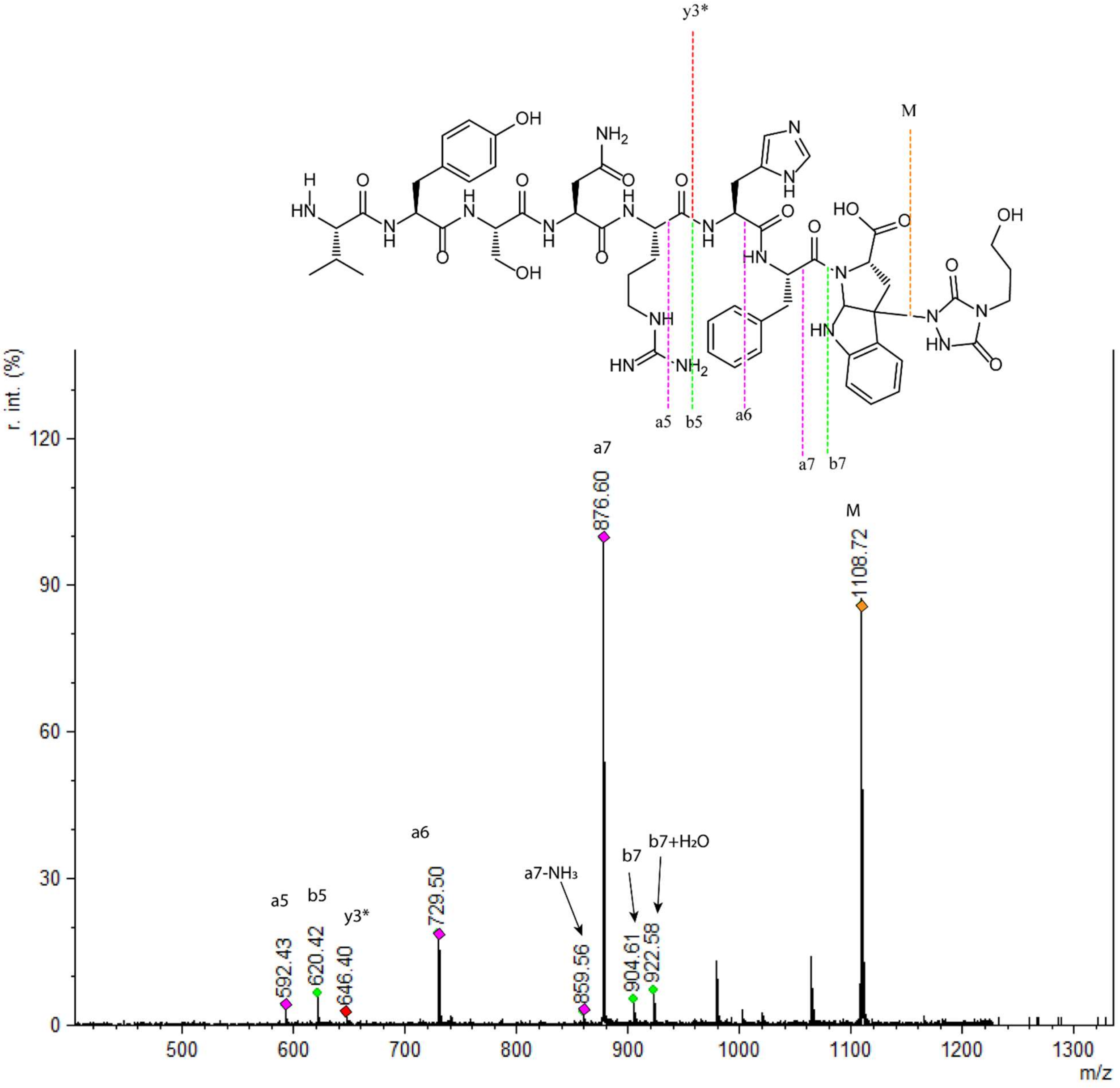

Figure S2.3.3.11 MALDI-TOF/TOF spectrum of peptide **1j** conjugated with **2b** (precursor ion: 1265.8 Da). Chemical structure of peptide **1j** with detected fragment ions is shown. Fragments with "*" are TAD modified fragments. Conjugation reaction was performed in 10x PBS pH 3.

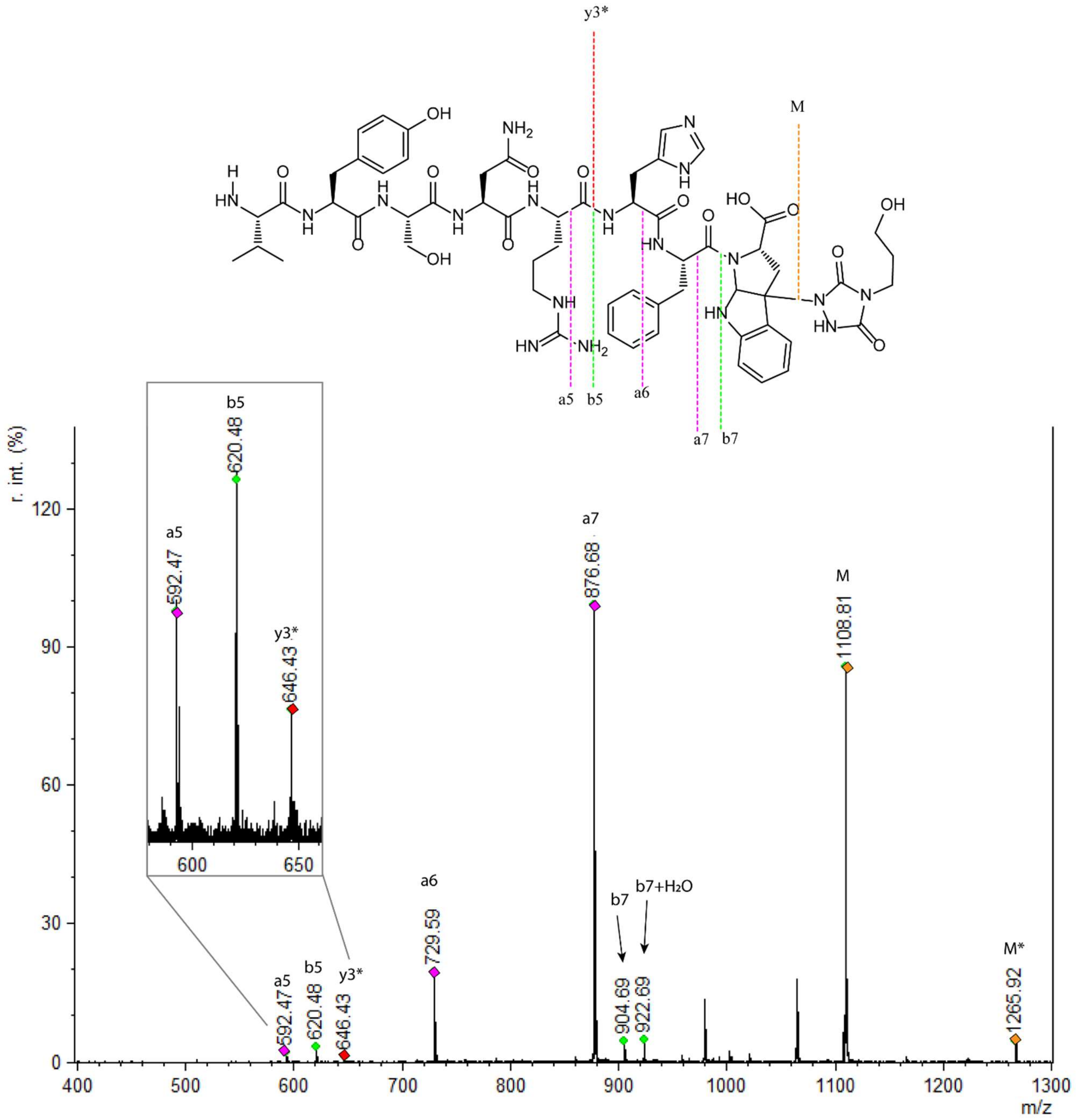

Figure S2.3.3.12 MALDI-TOF/TOF spectrum of peptide **1j** conjugated with **2b** (precursor ion: 1265.8 Da). Chemical structure of peptide **1j** with detected fragment ions is shown. Fragments with "*" are TAD modified fragments. Conjugation reaction was performed in 10x PBS pH 4.

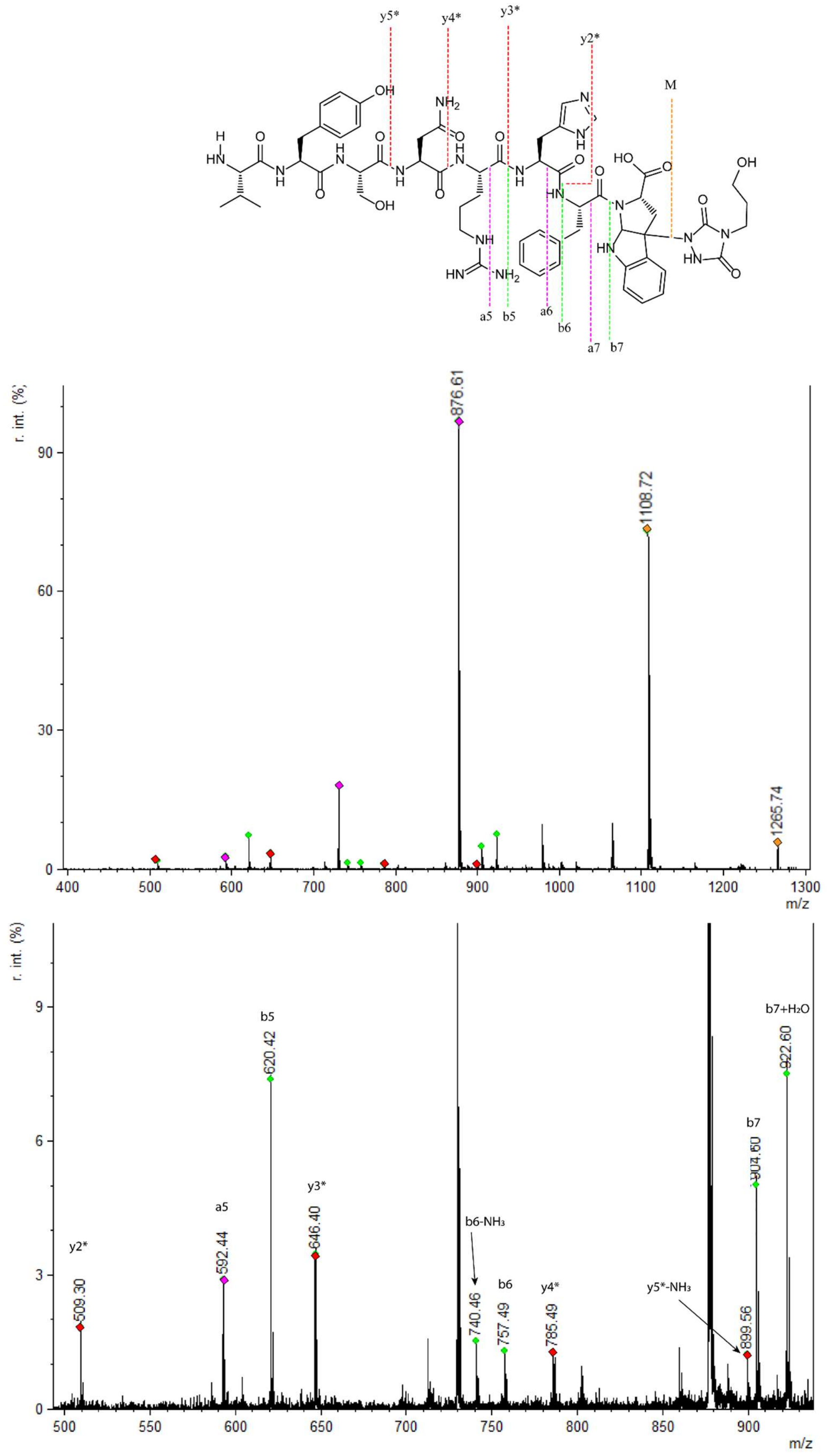

Figure S2.3.3.13 MALDI-TOF/TOF spectrum of peptide **1j** conjugated with **2b** (precursor ion: 1265.8 Da). Chemical structure of peptide **1j** with detected fragment ions is shown. Fragments with "*" are TAD modified fragments. Conjugation reaction was performed in 10x PBS pH 5.

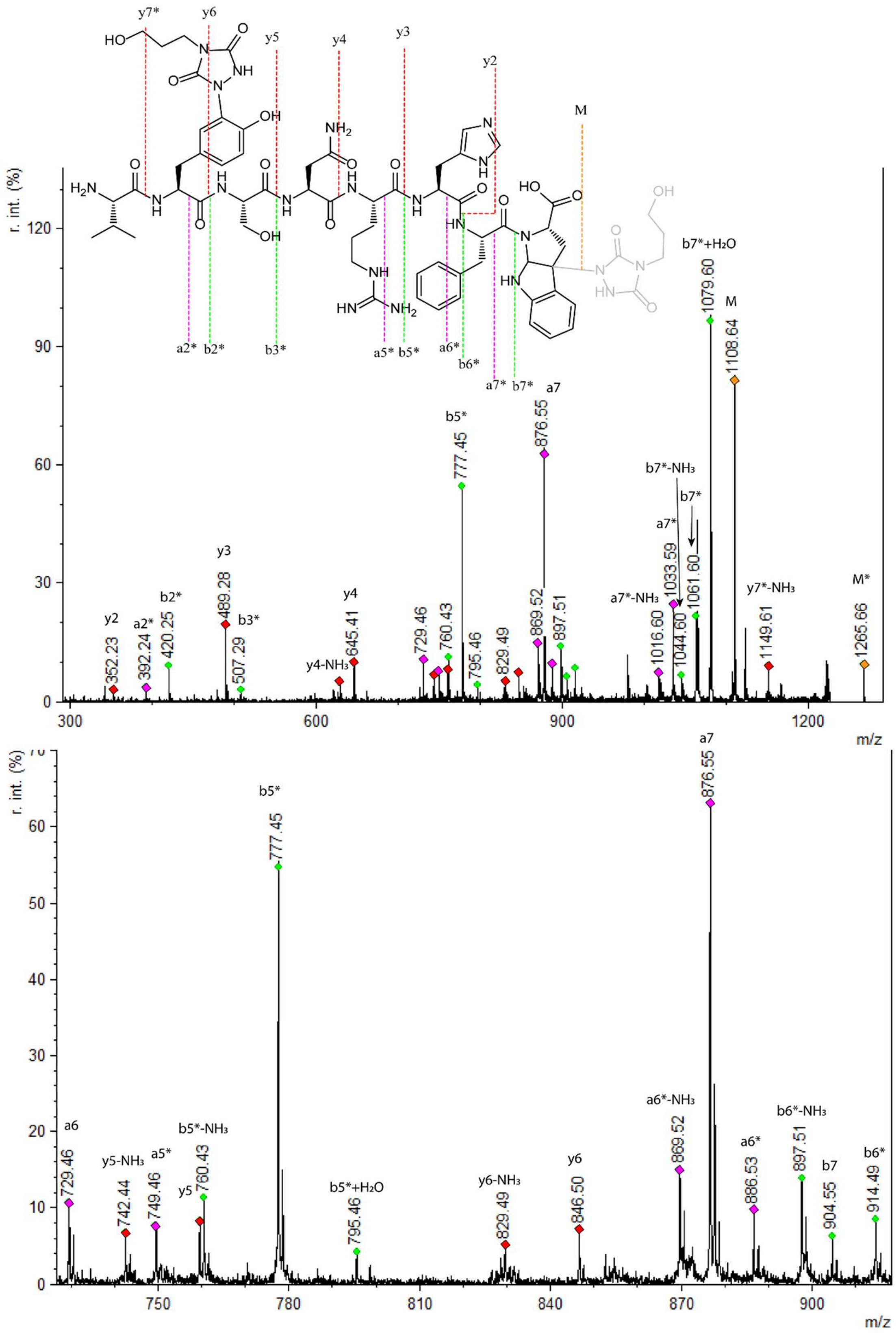

Figure S2.3.3.14 MALDI-TOF/TOF spectrum of peptide **1j** conjugated with **2b** (precursor ion: 1265.8 Da). Chemical structure of peptide **1j** with detected fragment ions is shown. Fragments with “*” are TAD modified fragments. Conjugation reaction was performed in 10x PBS pH 6.

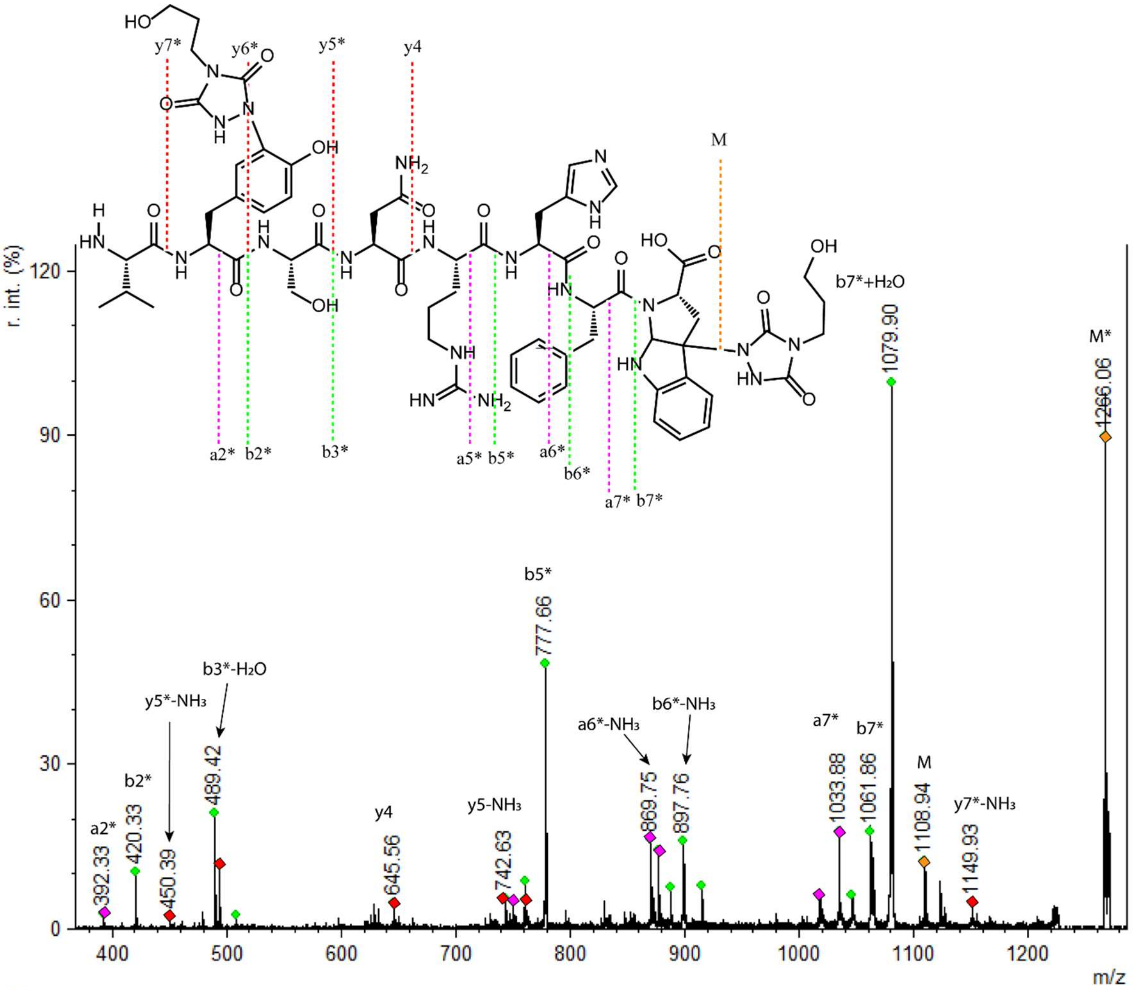
y7*
y6*
y5*
y4
M
a2* b2*
b3*
a5* b5*
a6*
b6*
a7* b7*
r. int. (%)
b7*+H₂O
1079.90
M*
1266.06
b5*
777.66
b3*-H₂O
y5*-NH₃
b2*
a2*
392.33
420.33
450.39
489.42
y4
645.56
y5-NH₃
742.63
a6*-NH₃
b6*-NH₃
869.75
897.76
a7*
b7*
1033.88
1061.86
M
1108.94
y7*-NH₃
1149.93
m/z

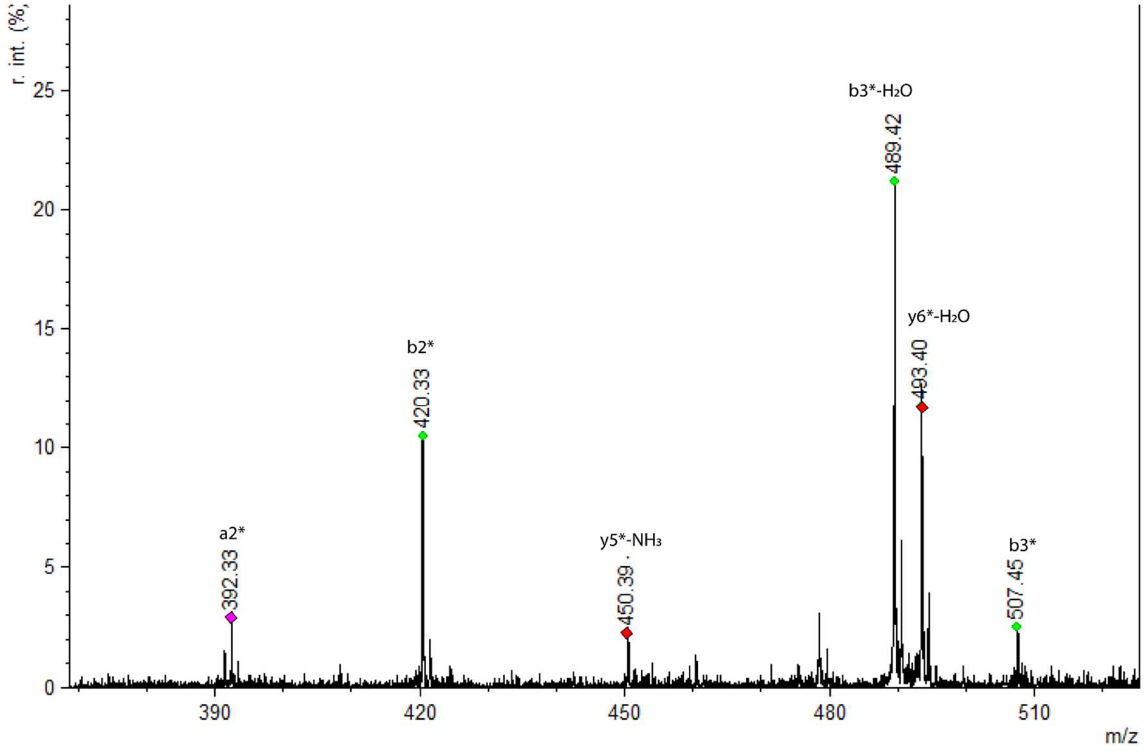
r. int. (%)
b3*-H₂O
489.42
y6*-H₂O
493.40
b2*
420.33
a2*
392.33
y5*-NH₃
450.39
b3*
507.45
m/z

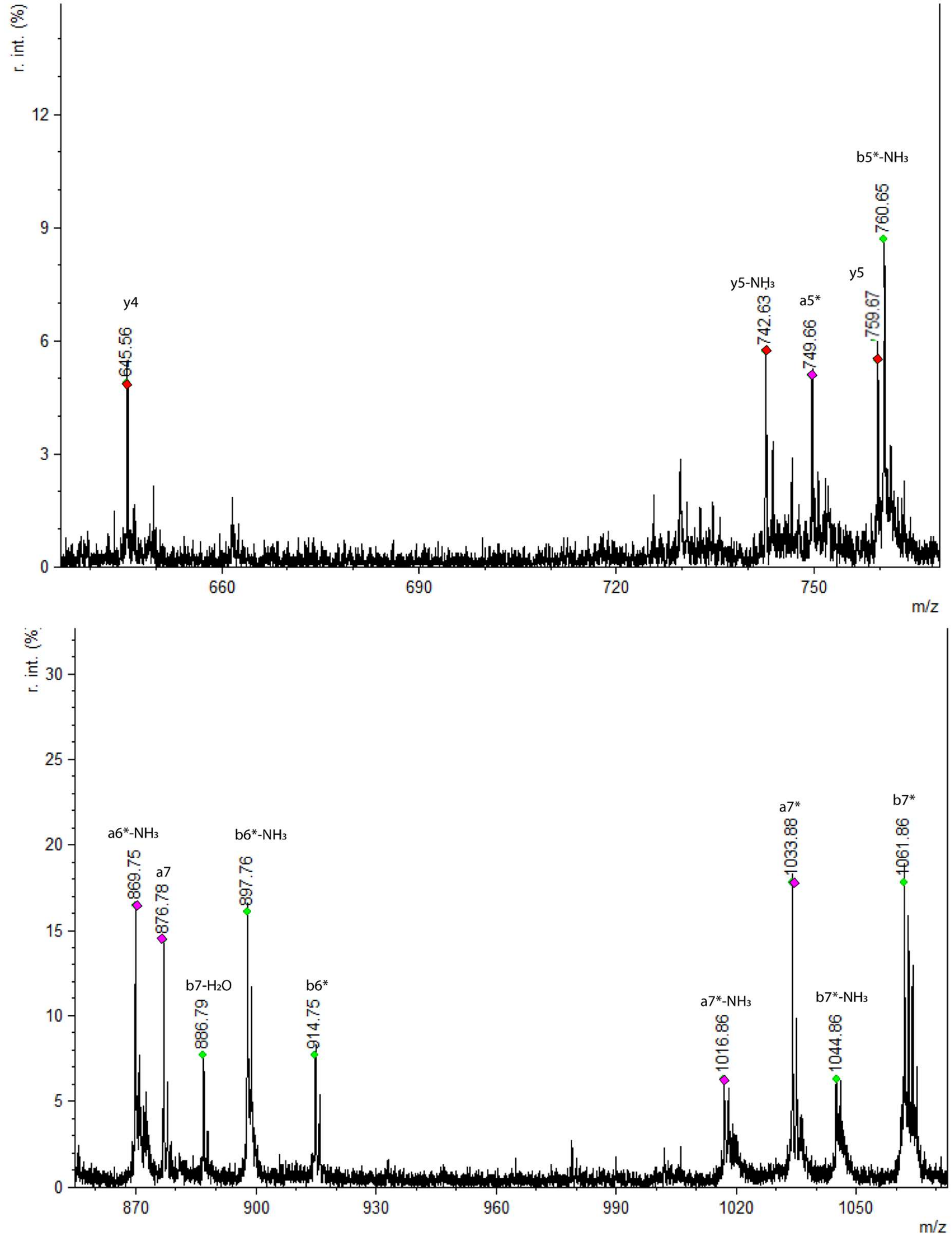

Figure S2.3.3.15 MALDI-TOF/TOF spectrum of peptide **1j** conjugated with **2b** (precursor ion: 1265.8 Da). Chemical structure of peptide **1j** with detected fragment ions is shown. Fragments with "*" are TAD modified fragments. Conjugation reaction was performed in 10x PBS pH 7.

Conjugation product of Val-Tyr-Ser-Asn-Arg-His-Phe-Trp-OH (VYSNRHFW, 1j) with PTAD-alkyne 2c (2jc). MS (MALDI-TOF) precursor ion: m/z 1337.8 (calcd $[M+H]^+$ 1337.5)

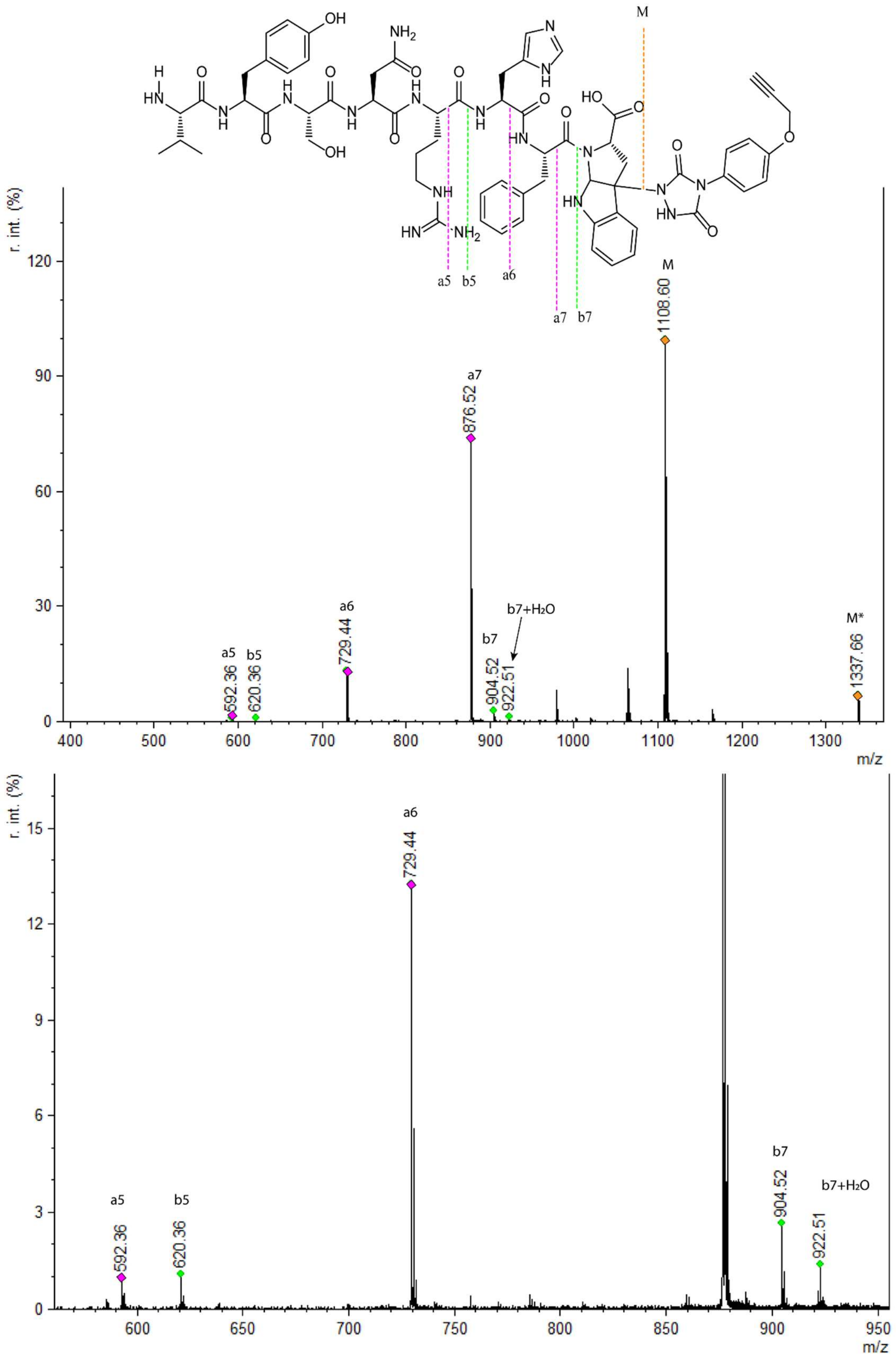

Figure S2.3.3.16 MALDI-TOF/TOF spectrum of peptide **1j** conjugated with **2c** (precursor ion: 1337.8 Da). Chemical structure of peptide **1j** with detected fragment ions is shown. Fragments with "*" are TAD modified fragments. Conjugation reaction was performed in 10x PBS pH 3.

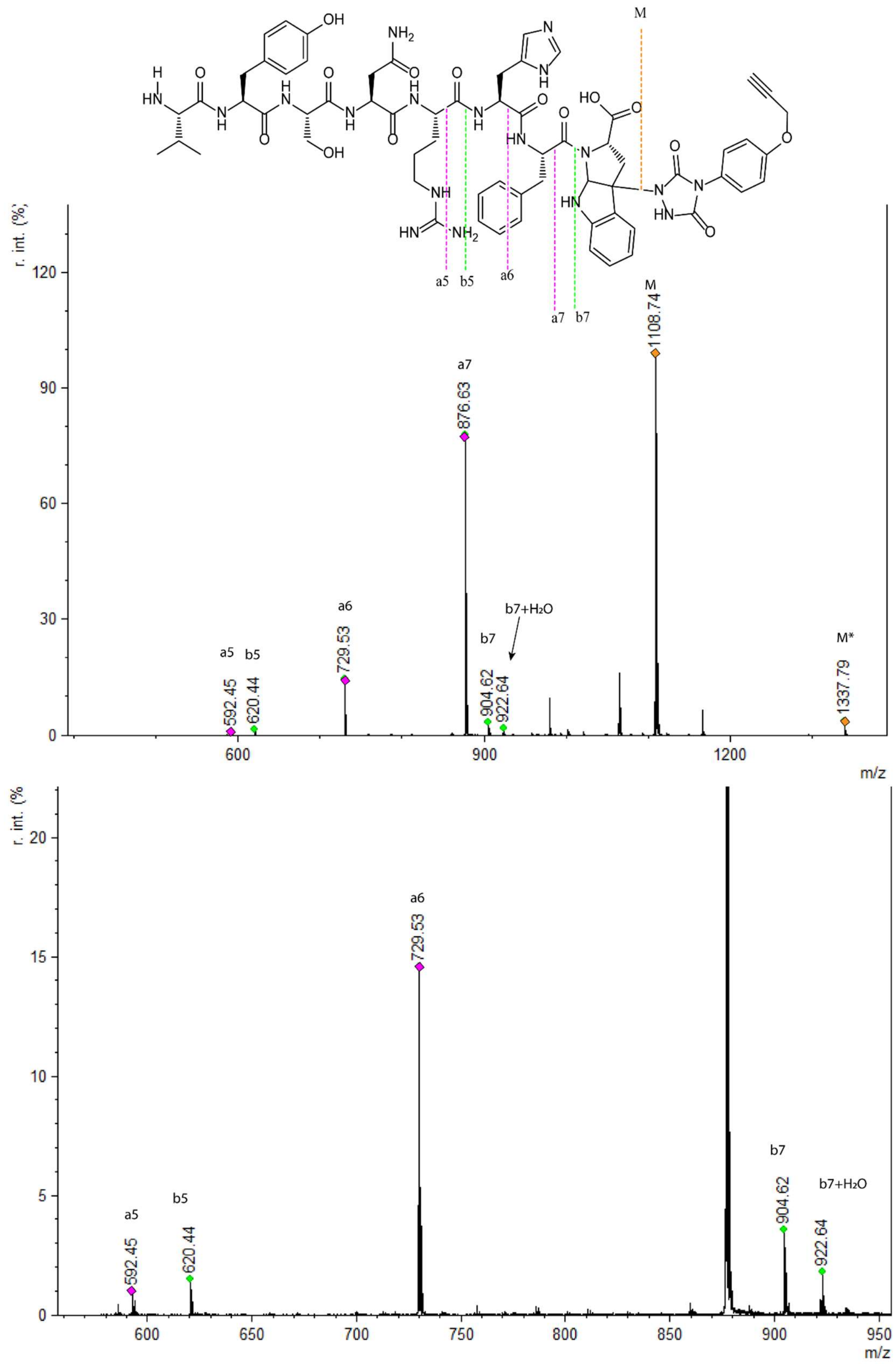

Figure S2.3.3.17 MALDI-TOF/TOF spectrum of peptide **1j** conjugated with **2c** (precursor ion: 1337.8 Da). Chemical structure of peptide **1j** with detected fragment ions is shown. Fragments with "*" are TAD modified fragments. Conjugation reaction was performed in 10x PBS pH 4.

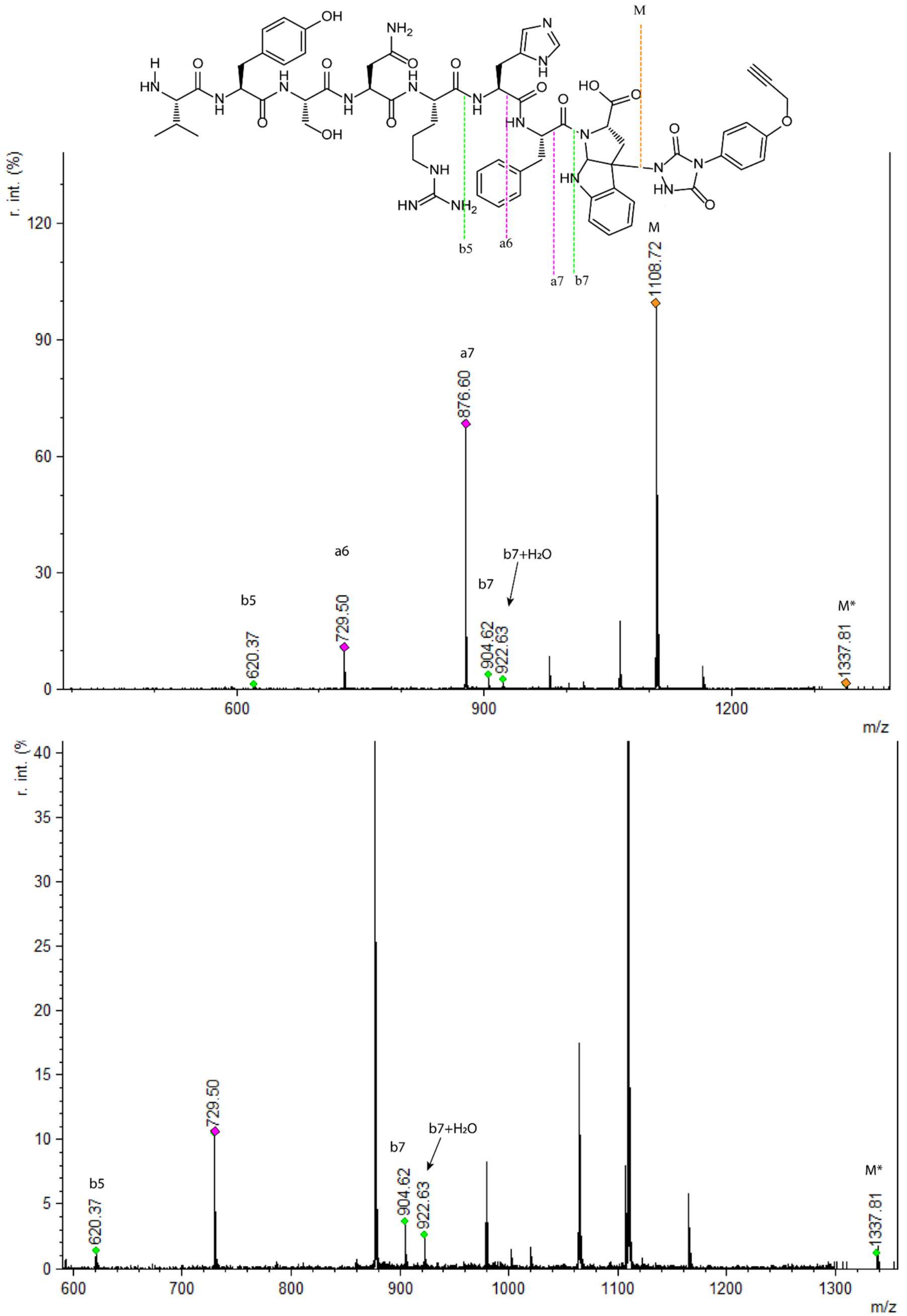

Figure S2.3.3.18 MALDI-TOF/TOF spectrum of peptide **1j** conjugated with **2c** (precursor ion: 1337.8 Da). Chemical structure of peptide **1j** with detected fragment ions is shown. Fragments with "*" are TAD modified fragments. Conjugation reaction was performed in 10x PBS pH 5.

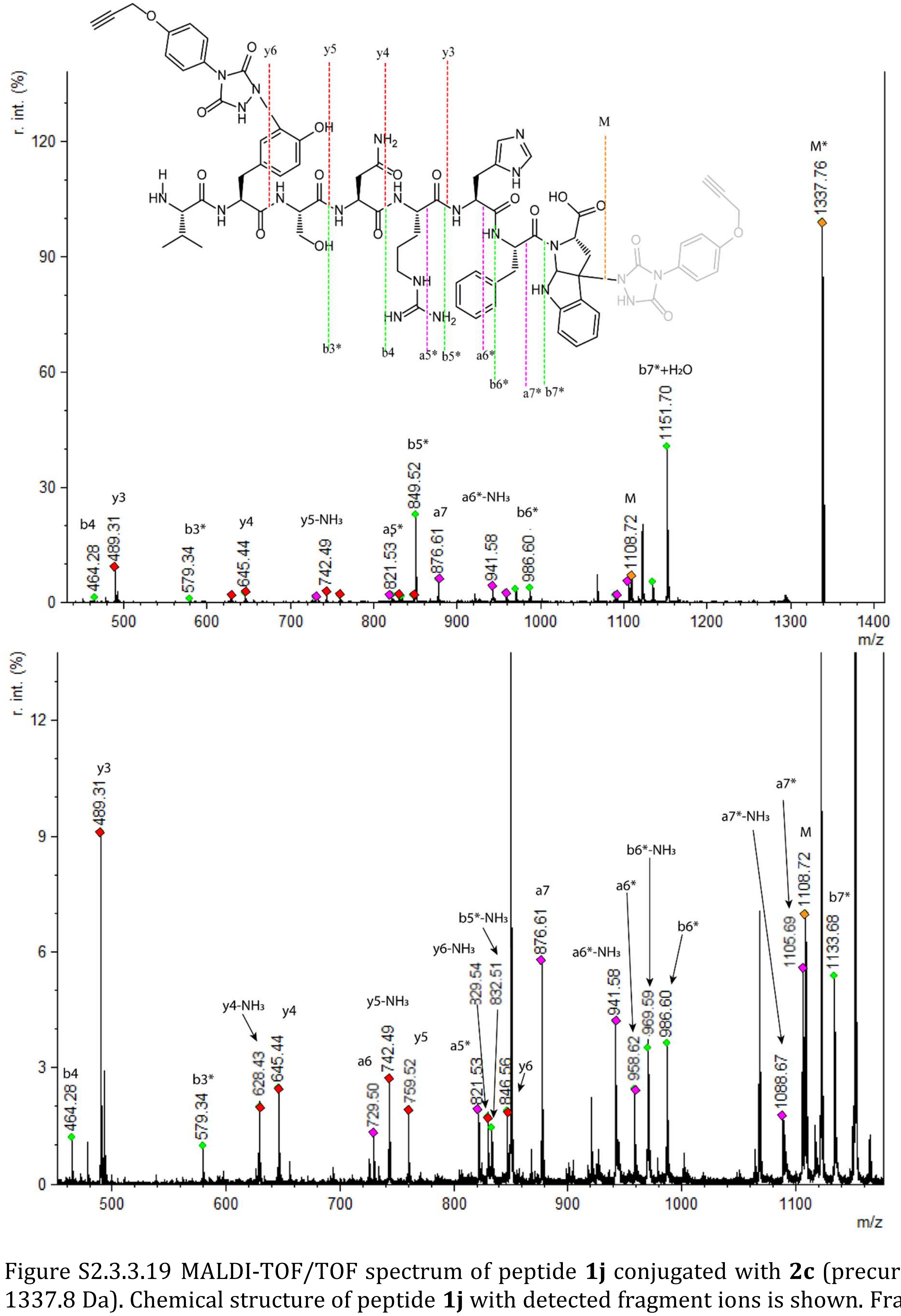

Figure S2.3.3.19 MALDI-TOF/TOF spectrum of peptide **1j** conjugated with **2c** (precursor ion: 1337.8 Da). Chemical structure of peptide **1j** with detected fragment ions is shown. Fragments with "*" are TAD modified fragments. Conjugation reaction was performed in 10x PBS pH 6.

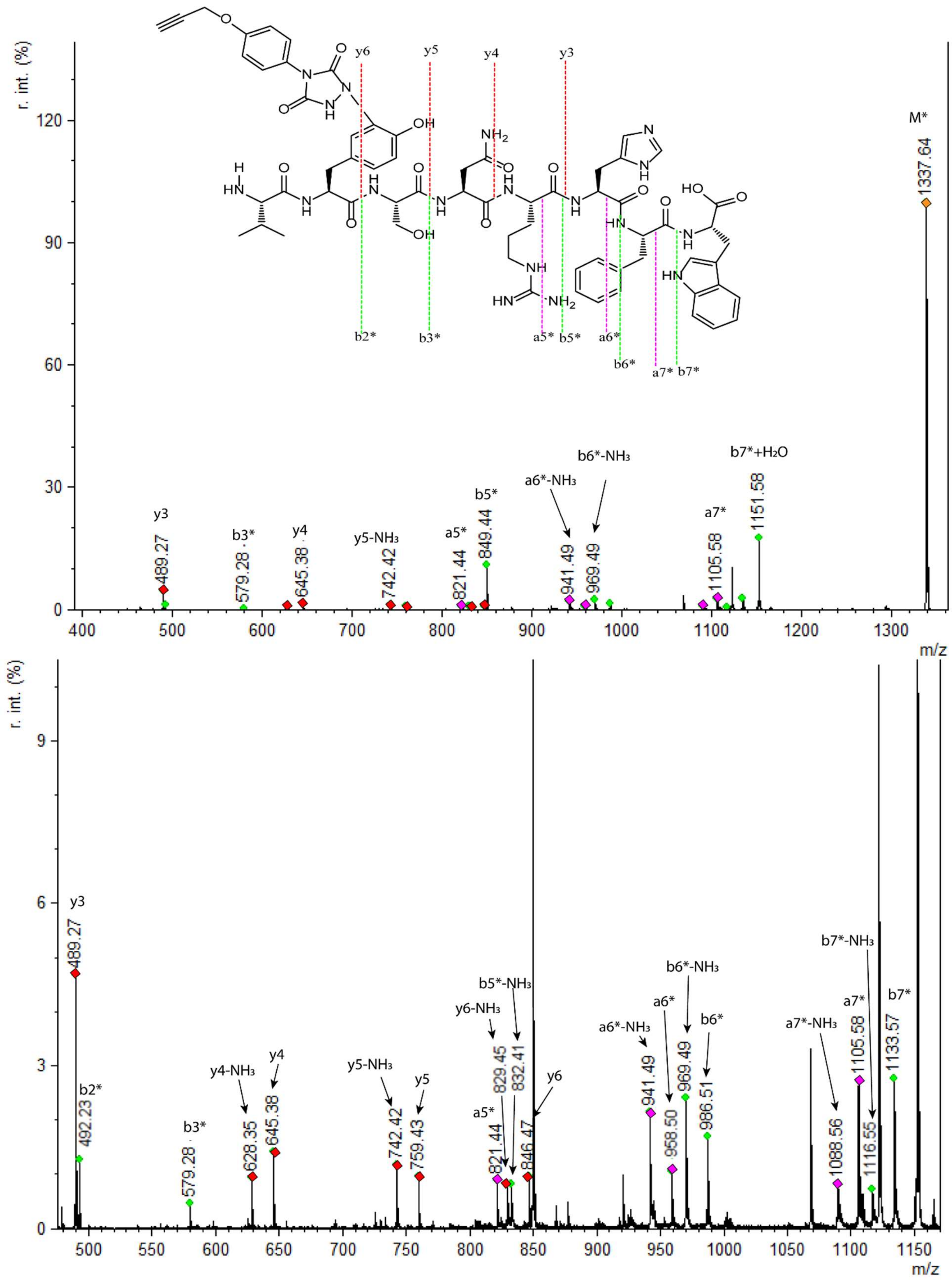

Figure S2.3.3.20 MALDI-TOF/TOF spectrum of peptide **1j** conjugated with **2c** (precursor ion: 1337.8 Da). Chemical structure of peptide **1j** with detected fragment ions is shown. Fragments with "*" are TAD modified fragments. Conjugation reaction was performed in 10x PBS pH 7.

Conjugation product of Val-Trp-Ser-Gln-Lys-Arg-His-Phe-Gly-Tyr-OH (VWSQKRHFGY, 1k) with TAD-propanol 2b (2kb). MS (MALDI-TOF) precursor ion: m/z 1464.6 (calcd $[M+H]^+$ 1464.7)

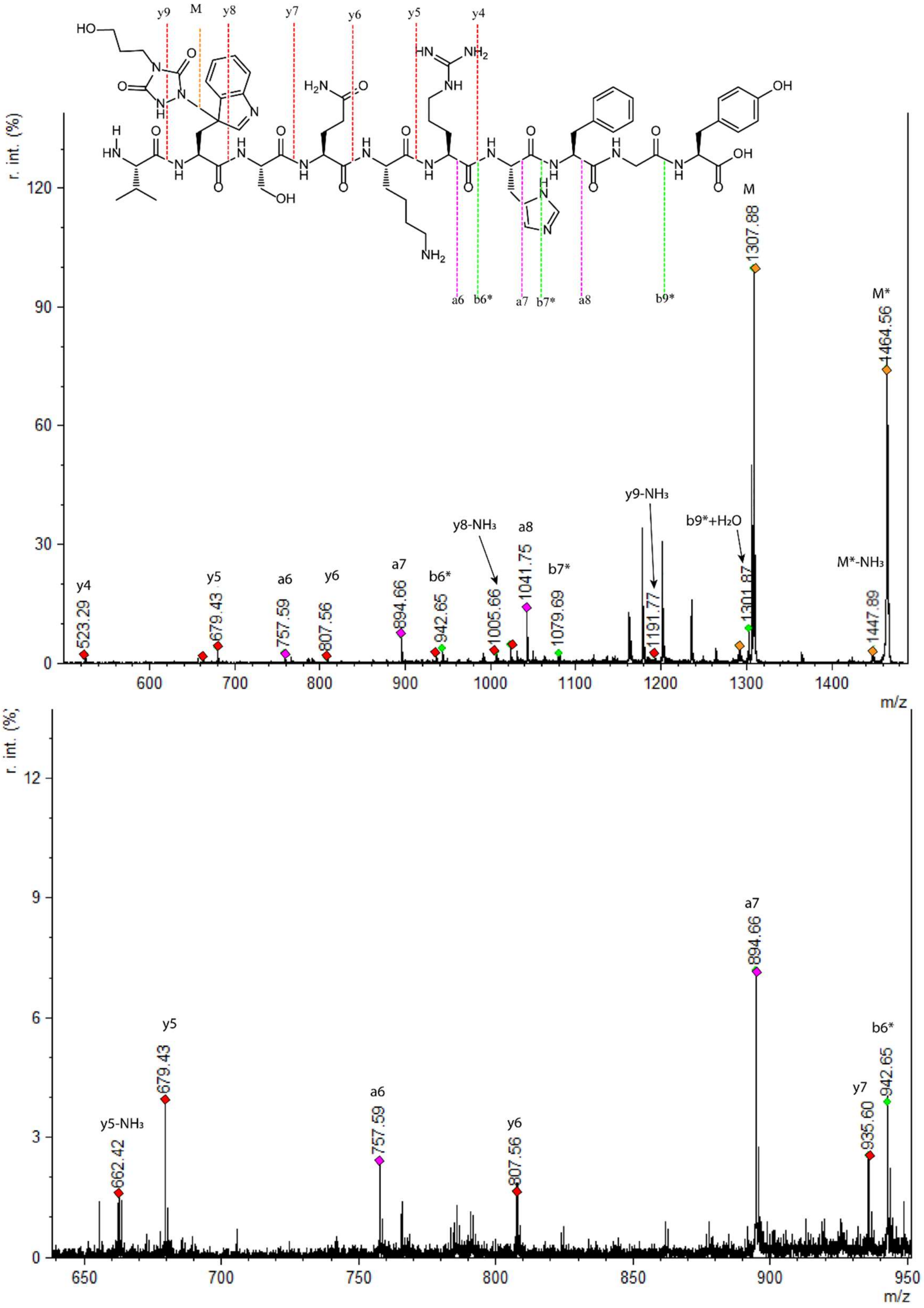

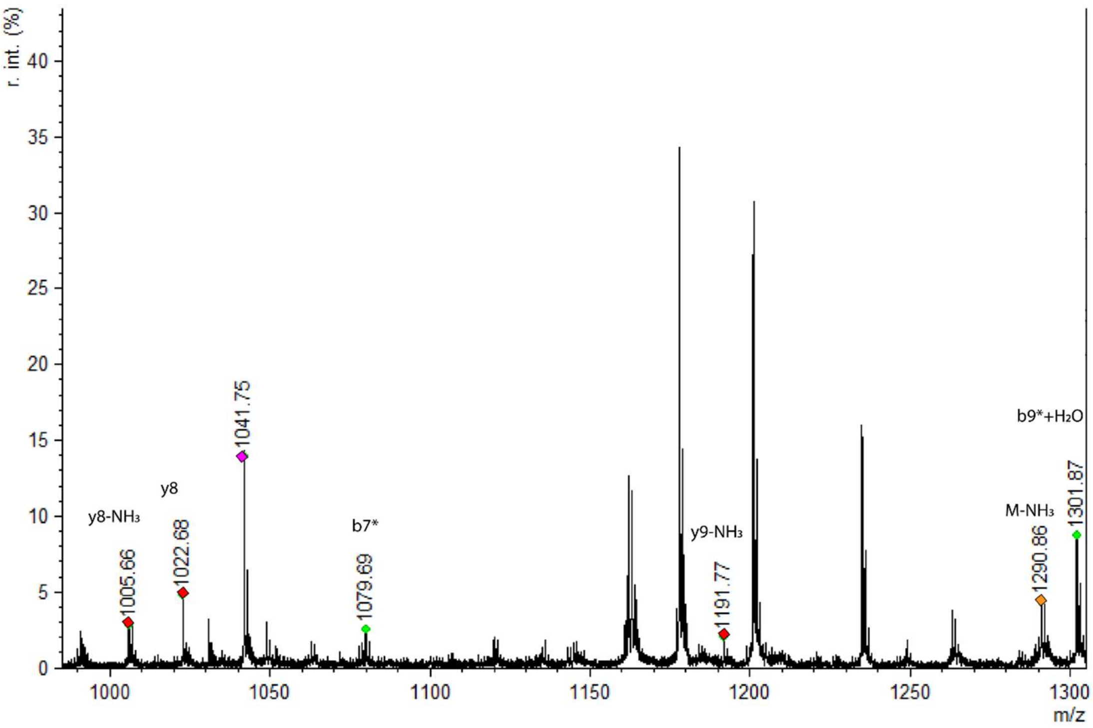

Figure S2.3.3.21 MALDI-TOF/TOF spectrum of peptide **1k** conjugated with **2b** (precursor ion: 1464.6 Da). Chemical structure of peptide **1k** with detected fragment ions is shown. Fragments with "*" are TAD modified fragments. Conjugation reaction was performed in 10x PBS pH3.

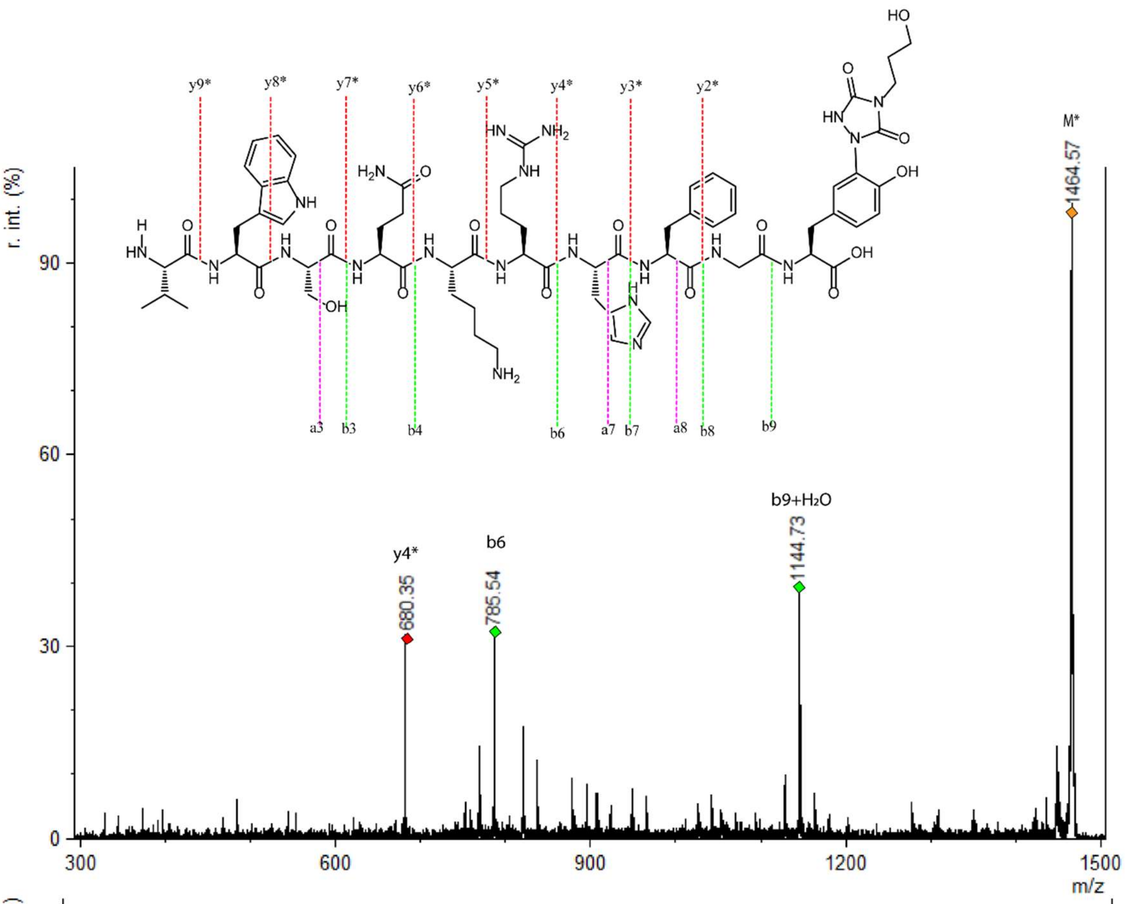

r. int. (%)
M*
1464.57
b9+H₂O
1144.73
y4*
680.35
b6
785.54
y9*
y8*
y7*
y6*
y5*
y4*
y3*
y2*
a3
b3
b4
b6
a7
b7
a8
b8
b9
m/z

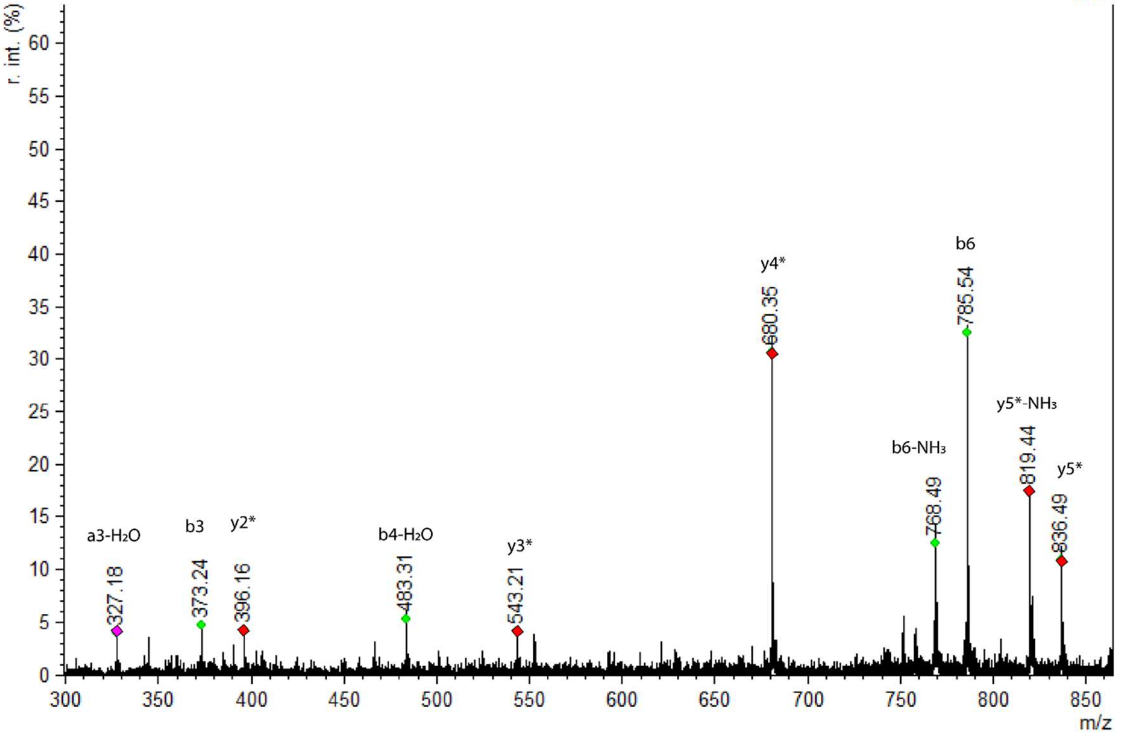

r. int. (%)
a3-H₂O
327.18
b3
373.24
y2*
396.16
b4-H₂O
483.31
y3*
543.21
y4*
680.35
b6-NH₃
768.49
b6
785.54
y5*-NH₃
819.44
y5*
836.49
m/z

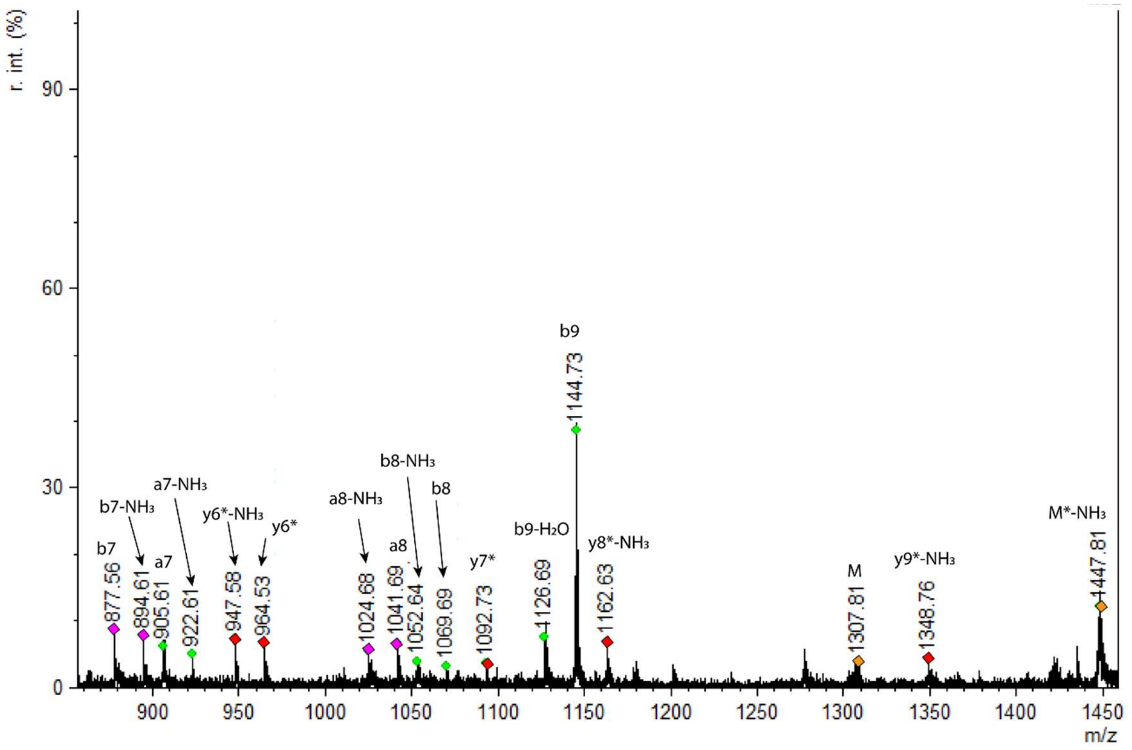

Figure S2.3.3.22 MALDI-TOF/TOF spectrum of peptide **1k** conjugated with **2b** (precursor ion: 1464.6 Da). Chemical structure of peptide **1k** with detected fragment ions is shown. Fragments with "*" are TAD modified fragments. Conjugation reaction was performed in 10x PBS pH 7.

Conjugation product of Val-Trp-Ser-Gln-Lys-Arg-His-Phe-Gly-Tyr-OH (VWSQKRHFGY, 1k) with PTAD-alkyne 2c (2kc). MS (MALDI-TOF) precursor ion: m/z 1536.6 (calcd $[M+H]^+$ 1536.7)

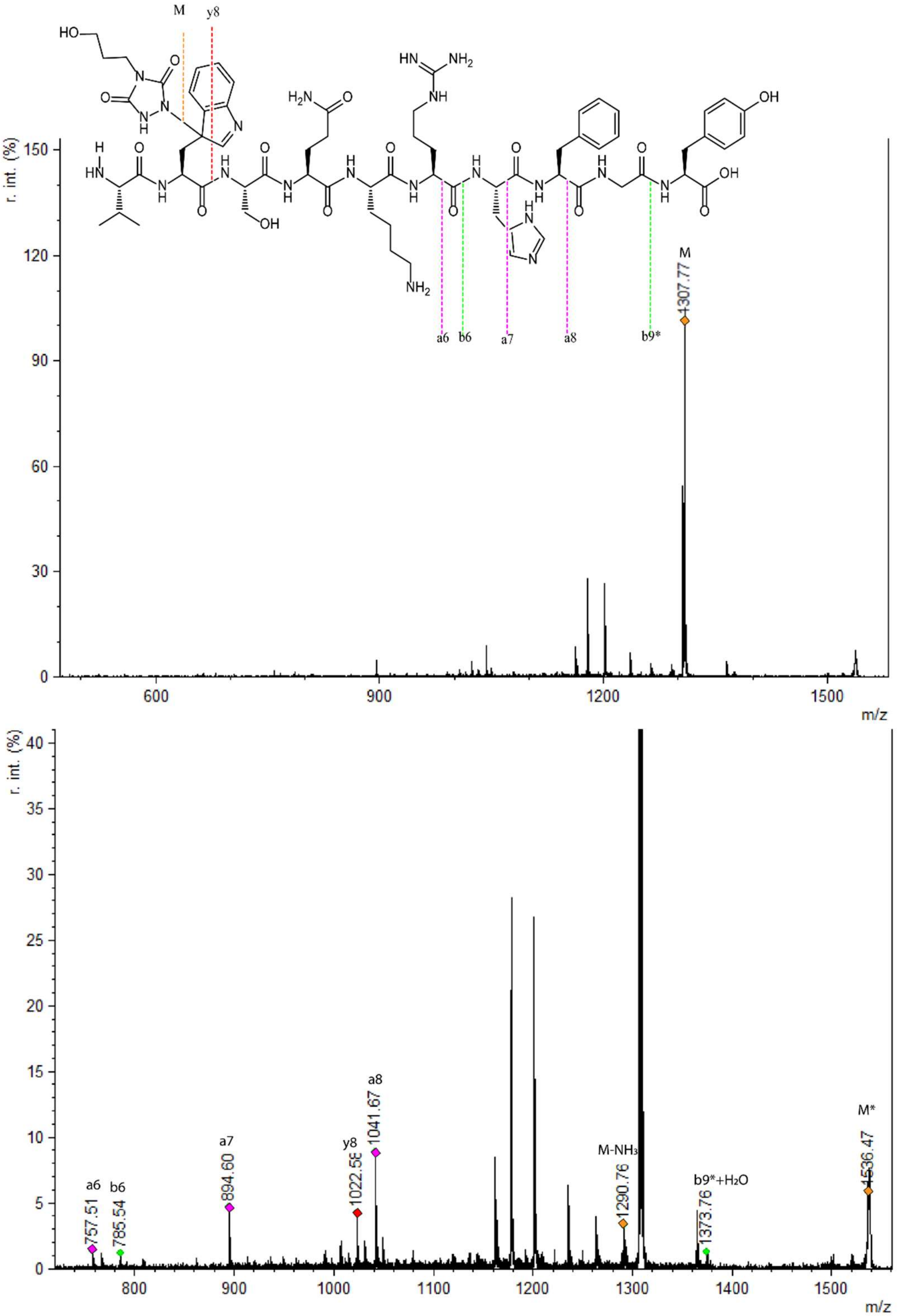

Figure S2.3.3.23 MALDI-TOF/TOF spectrum of peptide **1k** conjugated with **2c** (precursor ion: 1536.6 Da). Chemical structure of peptide **1k** with detected fragment ions is shown. Fragments with "*" are TAD modified fragments. Conjugation reaction was performed in 10x PBS pH 3.

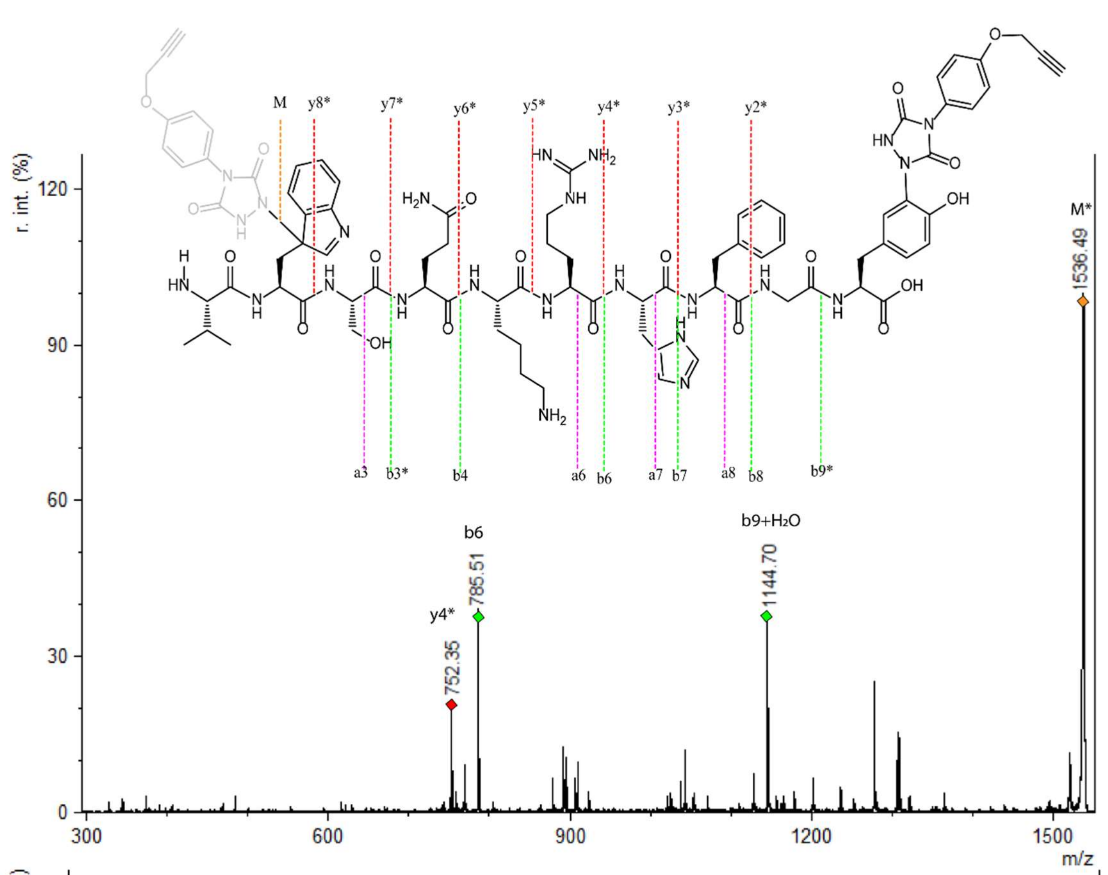
M
y8*
y7*
y6*
y5*
y4*
y3*
y2*
a3
b3*
b4
a6
b6
a7
b7
a8
b8
b9*
r. int. (%)
m/z
M*
1536.49
b6
785.51
y4*
752.35
b9+H₂O
1144.70

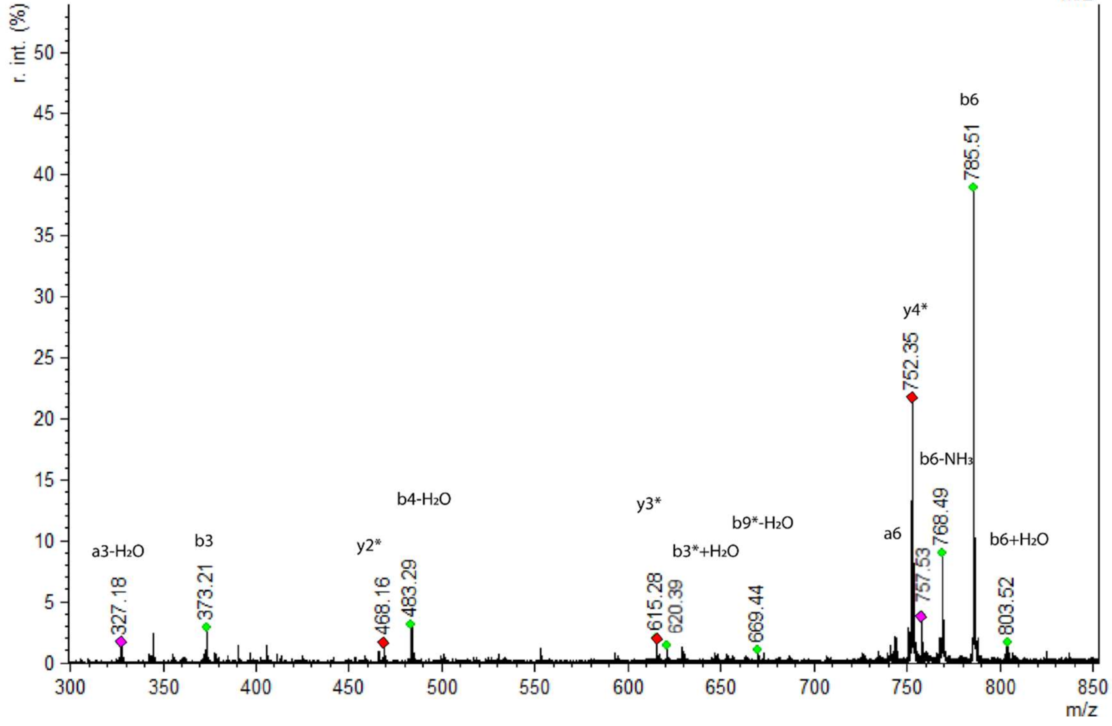
r. int. (%)
m/z
a3-H₂O
327.18
b3
373.21
y2*
468.16
b4-H₂O
483.29
y3*
615.28
b3*+H₂O
620.39
b9*-H₂O
669.44
a6
757.53
y4*
752.35
b6-NH₃
768.49
b6
785.51
b6+H₂O
803.52

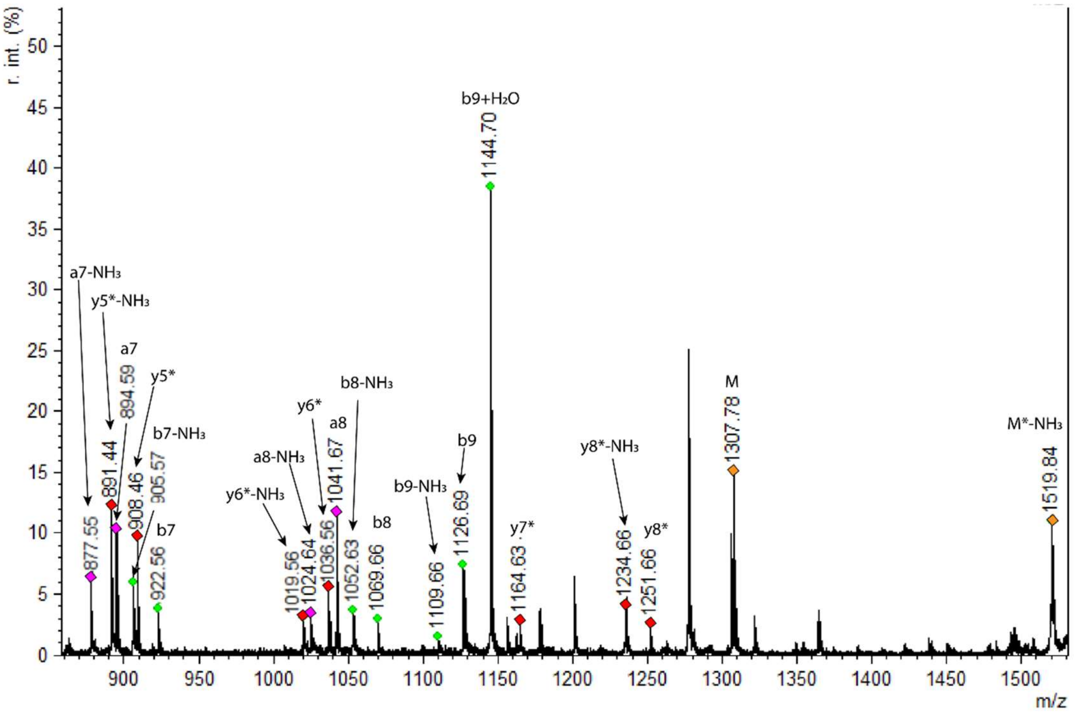

Figure S2.3.3.24 MALDI-TOF/TOF spectrum of peptide **1k** conjugated with **2c** (precursor ion: 1536.6 Da). Chemical structure of peptide **1k** with detected fragment ions is shown. Fragments with "*" are TAD modified fragments. Conjugation reaction was performed in 10x PBS pH 7.

Conjugation product of Lys-Asp-Tyr-Trp-Glu-Cys-Ala-OH (KDYWECA, 1l) with TAD-propanol 2b (2lb). MS (MALDI-TOF) precursor ion: m/z 1071.4 (calcd $[M+H]^+$ 1071.4)

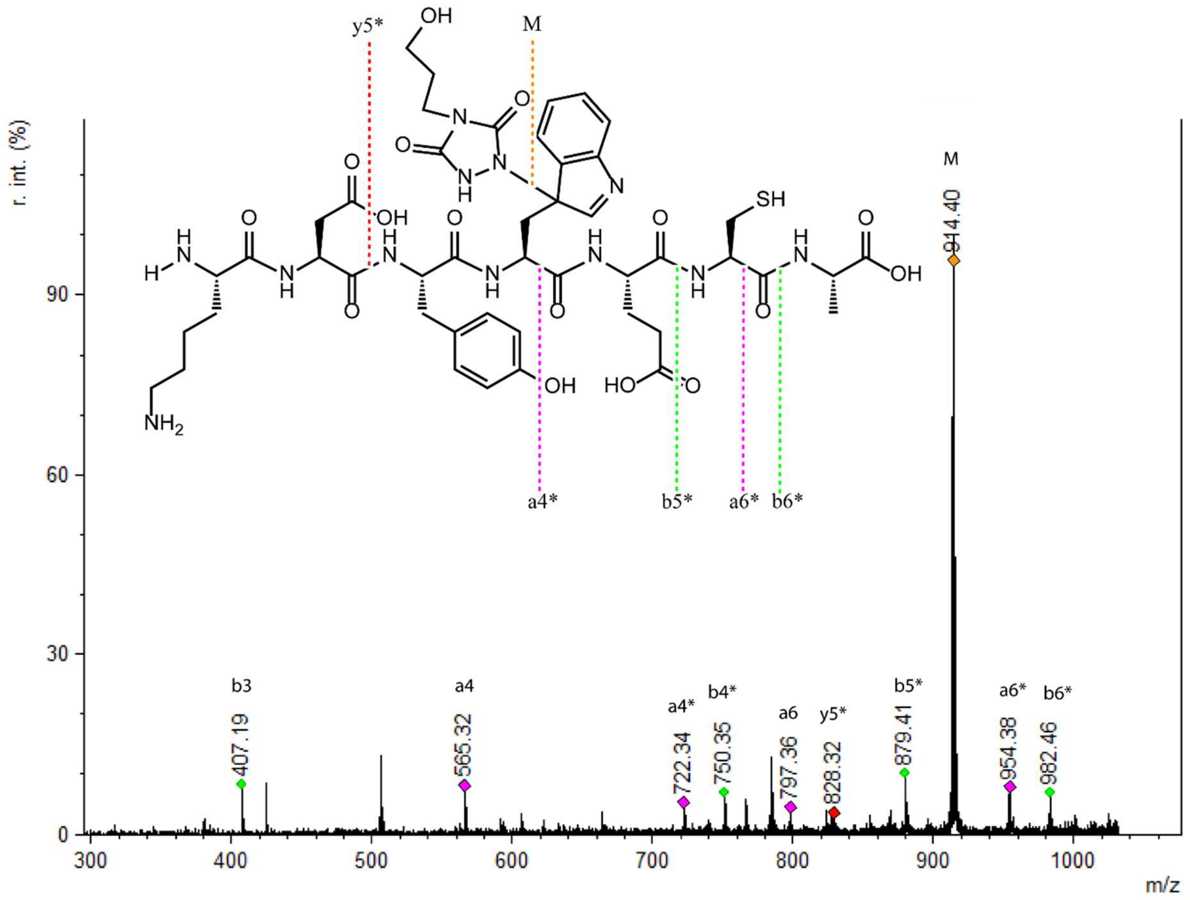

Figure S2.3.3.25 MALDI-TOF/TOF spectrum of peptide **1l** conjugated with **2b** (precursor ion: 1071.4 Da). Chemical structure of peptide **1l** with detected fragment ions is shown. Fragments with "*" are TAD modified fragments. Conjugation reaction was performed in MilliQ H_2O.

2.3.4 MS/MS tryptophan-TAD fragmentation patterns

In this section several recurring peaks in the MS/MS spectra of TAD conjugated peptides are investigated in more detail. In the MS/MS spectra of peptides containing a tryptophan residue a peak was observed after reaction with TAD corresponding with the cleavage of the Cα-Cβ bond of the labeled tryptophan residue. This mass can not be attributed to a TAD modified fragment since the same mass is found irrespective of the nature of the TAD reagent used. For peptide **1j** with a C-terminal tryptophan an additional alternative fragment was observed in the MS/MS spectra. The bond between C3 of the indole and TAD-nitrogen is cleaved as well as a decarboxylation taking place. The same mass is found in MS/MS spectra of peptides modified with different TAD reagents.

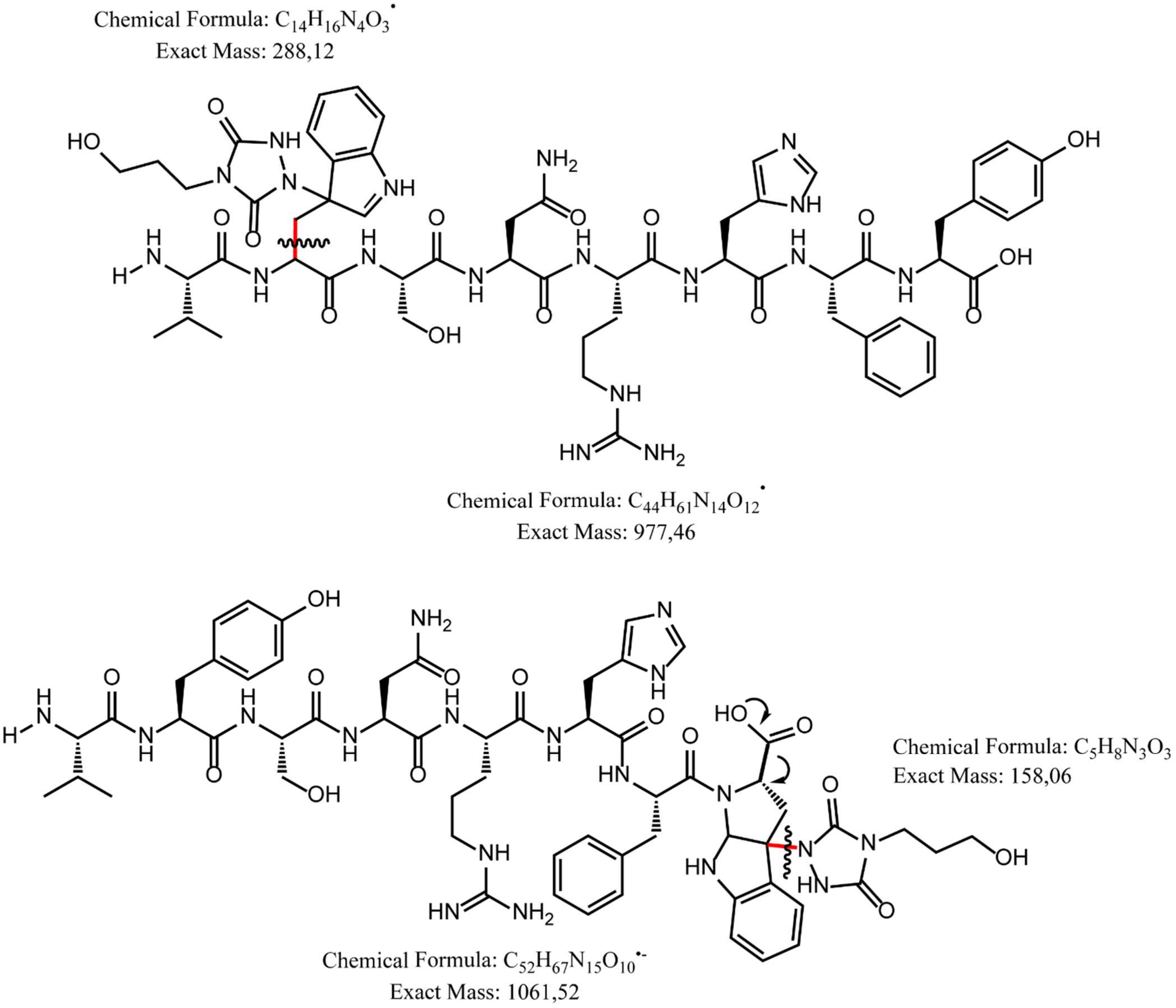

Figure S2.3.4.1 Structural representation of VWSNRHFY, **1i** modified with TAD propanol, **2b**. The Cα-Cβ bond of the tryptophan residue is cleaved and the resulting mass for the radicals is depicted (top). The expected mass for the radical is 977.5 Da and this mass can be related to a peak in the MS/MS spectrum $[M+H]^{+\cdot}$ of 978.5 Da (peak not picked up by the mMass software). This peak appears in the MS/MS spectra of **1i** conjugated with **2b** or **2c** especially when the modification was performed at lower pH (higher Trp modification). Structural representation of VYSNRHFW, **1j** modified with TAD propanol, **2b** (bottom). The bond between C3 of the indole

and TAD-nitrogen is cleaved as well as a decarboxylation. The expected mass for the fragment ion $[M]^{-\cdot}$ is 1061.5 Da and can be related to a peak in the MS/MS spectrum $[M+2H]^{+\cdot}$ of 1063.5 Da (peak not picked up by the mMass software). This peak appears in the MS/MS spectra of **1j** conjugated with **2b** or **2c** especially when the modification was performed at lower pH (higher Trp modification).

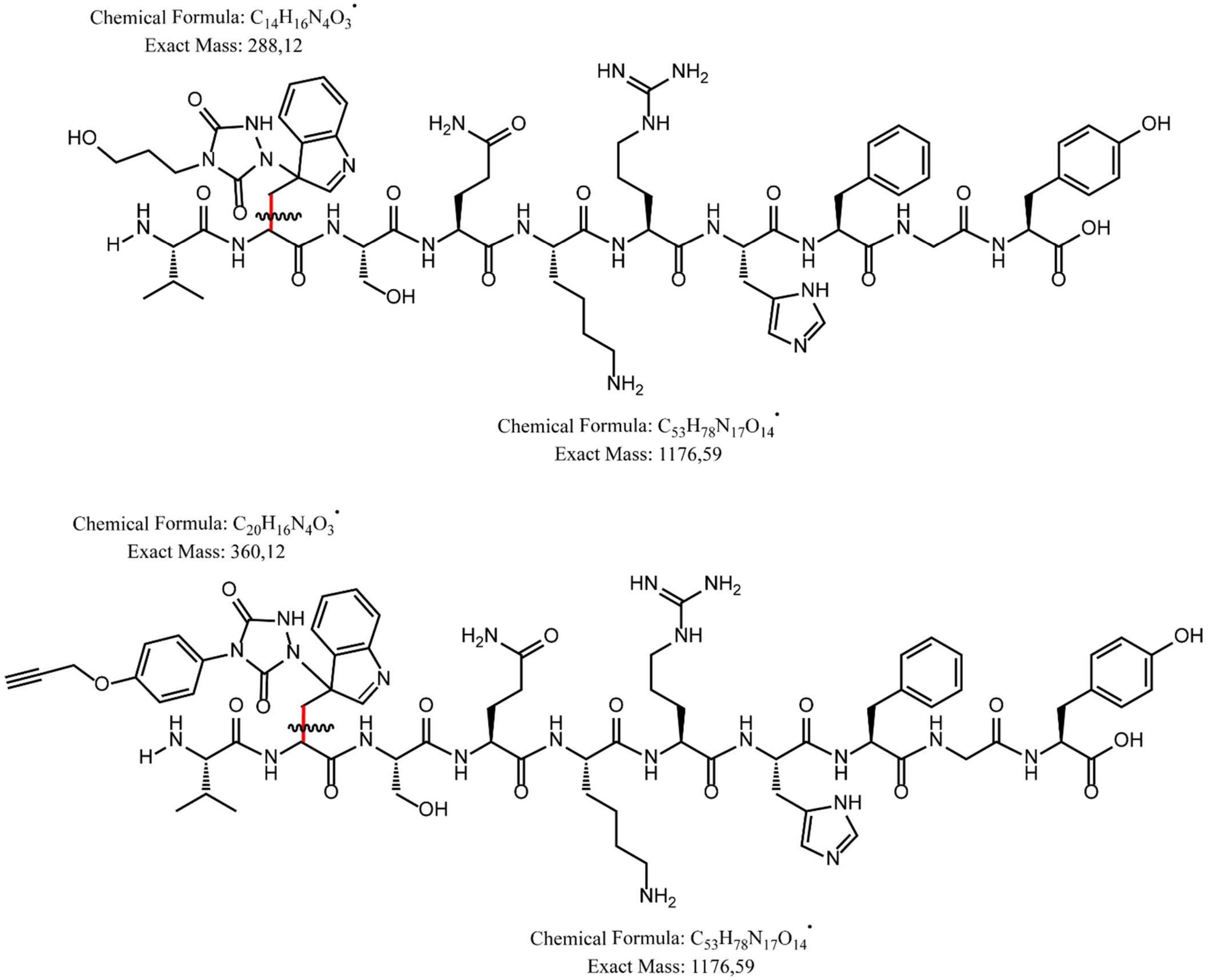

Figure S2.3.4.2 Structure representation of VWSQKRHFGY, **1k** with TAD-propanol, **2b**. The Cα-Cβ bond of the tryptophan residue is cleaved (top). Structure representation of VWSQKRHFGY, **1k** with PTAD-alkyne, **2c**. The Cα-Cβ bond of the tryptophan residue is cleaved (bottom).The expected mass for the radical is 1176.6 Da and this mass can be related to a peak in the MS/MS spectrum of 1177.7 Da (peak not picked up by the mMass software). This peak appears in the MS/MS spectra of **1k** conjugated with **2b** or **2c** when the modification was performed at lower pH (Trp modification).

2.4 LC (ESI) MS Fusion Lumos

2.4.1 VWSQKRHFGY peptide **1k** with TAD-propanol **2b**.

The peptide modification reaction was performed according to the general protocol (section 1.3) but with 3 equivalents of TAD-propanol. The mixture was analyzed on Fusion Lumos as described above.

The graphs below show the intensity of the double, triple and quadruple charged single TAD propanol modified peptide ion as a function of the retention time (Skyline software).

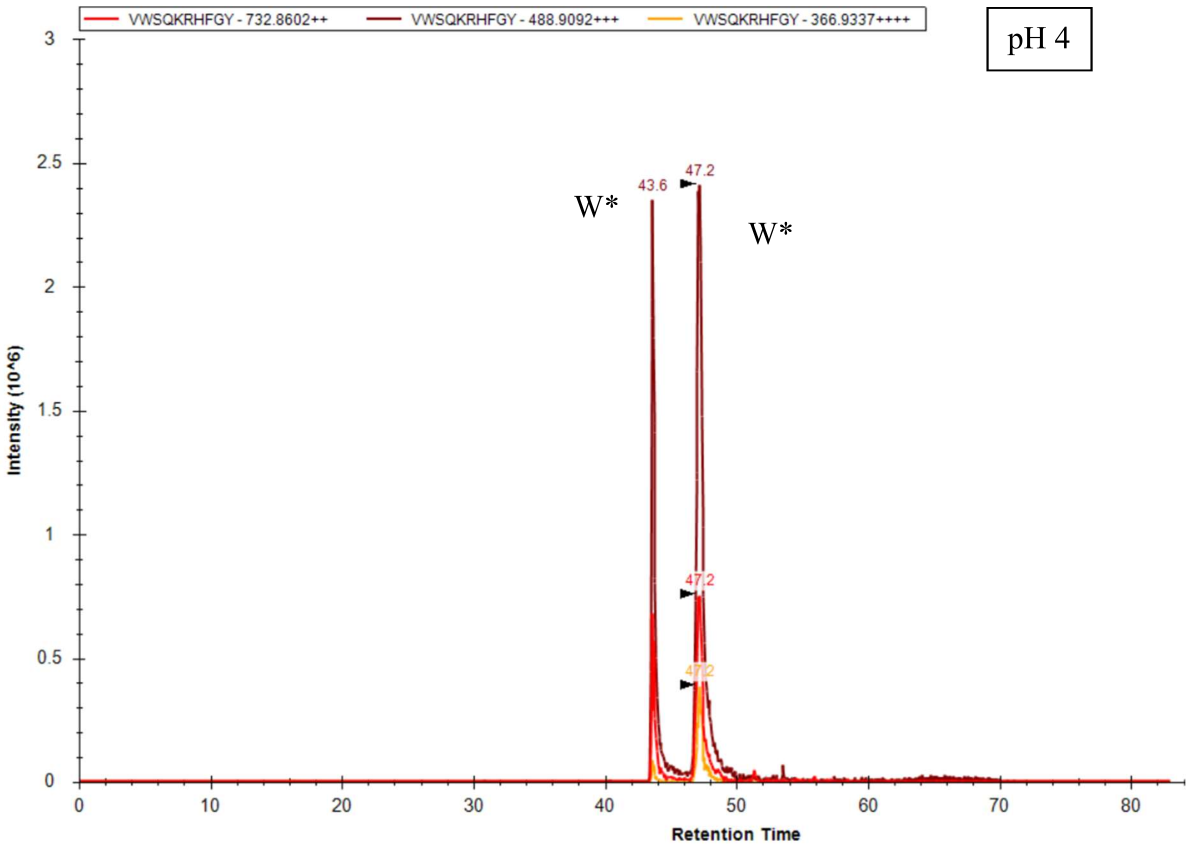

Figure 2.4.1.1 Ion chromatogram showing the double, triple and quadruple charged single TAD propanol modified peptide ions. The conjugation reaction of peptide **1k** with TAD-propanol **2b** was performed in 10 X PBS pH 4. 2 peaks corresponding with the double, triple and quadruple charged modified peptide ions at t_R = 43.6 min and at t_R = 47.2 min are observed. The ETD analysis of the triple charged modified peptide ion (488.91+++) in both peaks correspond to modification on tryptophan (see figure 2.4.1.2 and 2.4.1.3 and figure 3 (top) in the main text). These two peaks correspond with both diastereomers of the TAD modified tryptophan product.

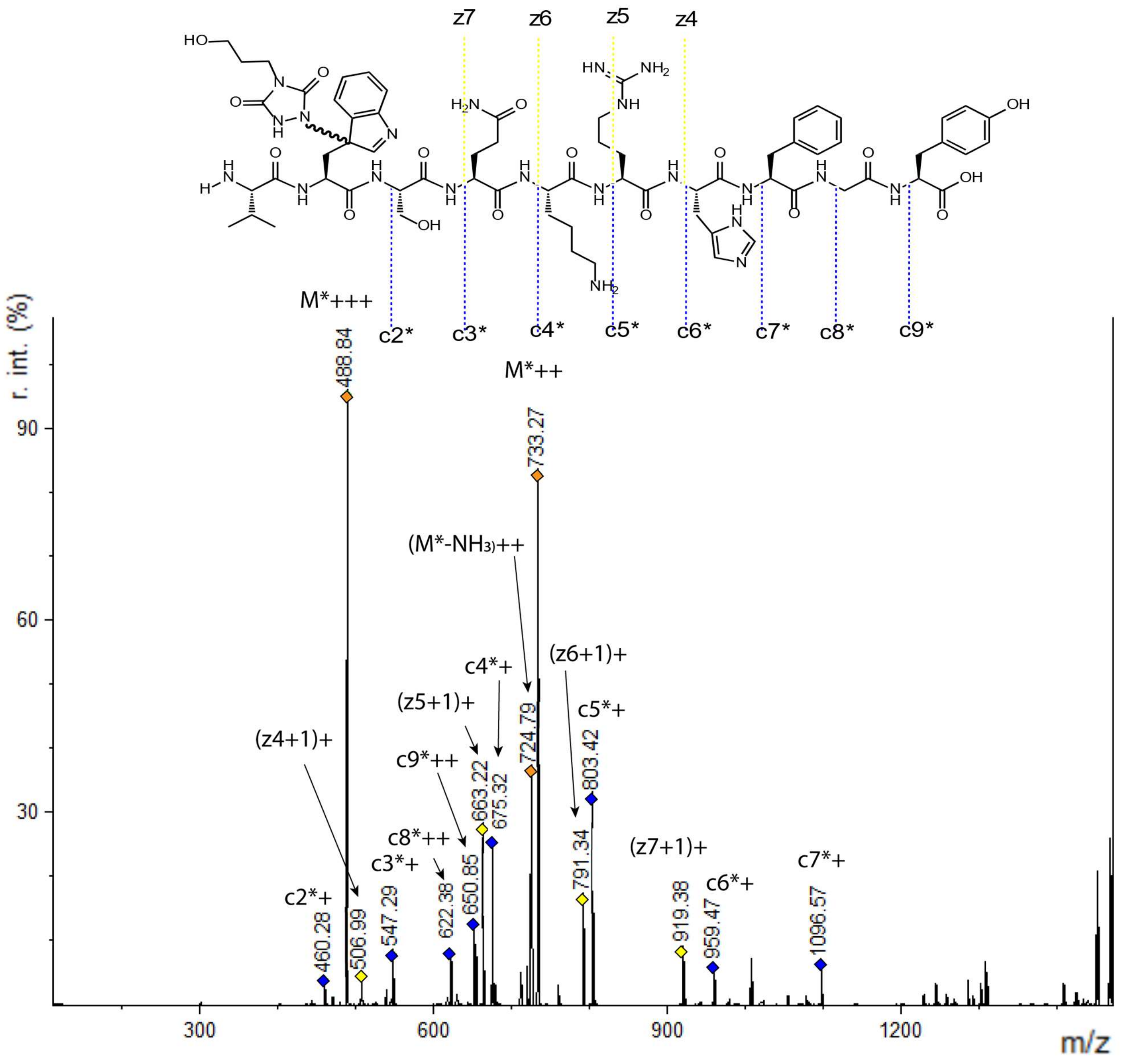

Figure 2.4.1.2 ETD MS/MS analysis at t_R = 43.62 min, precursor: TAD propanol modified peptide **1k** (488.91+++). Modification was done in 10 X PBS pH 4.

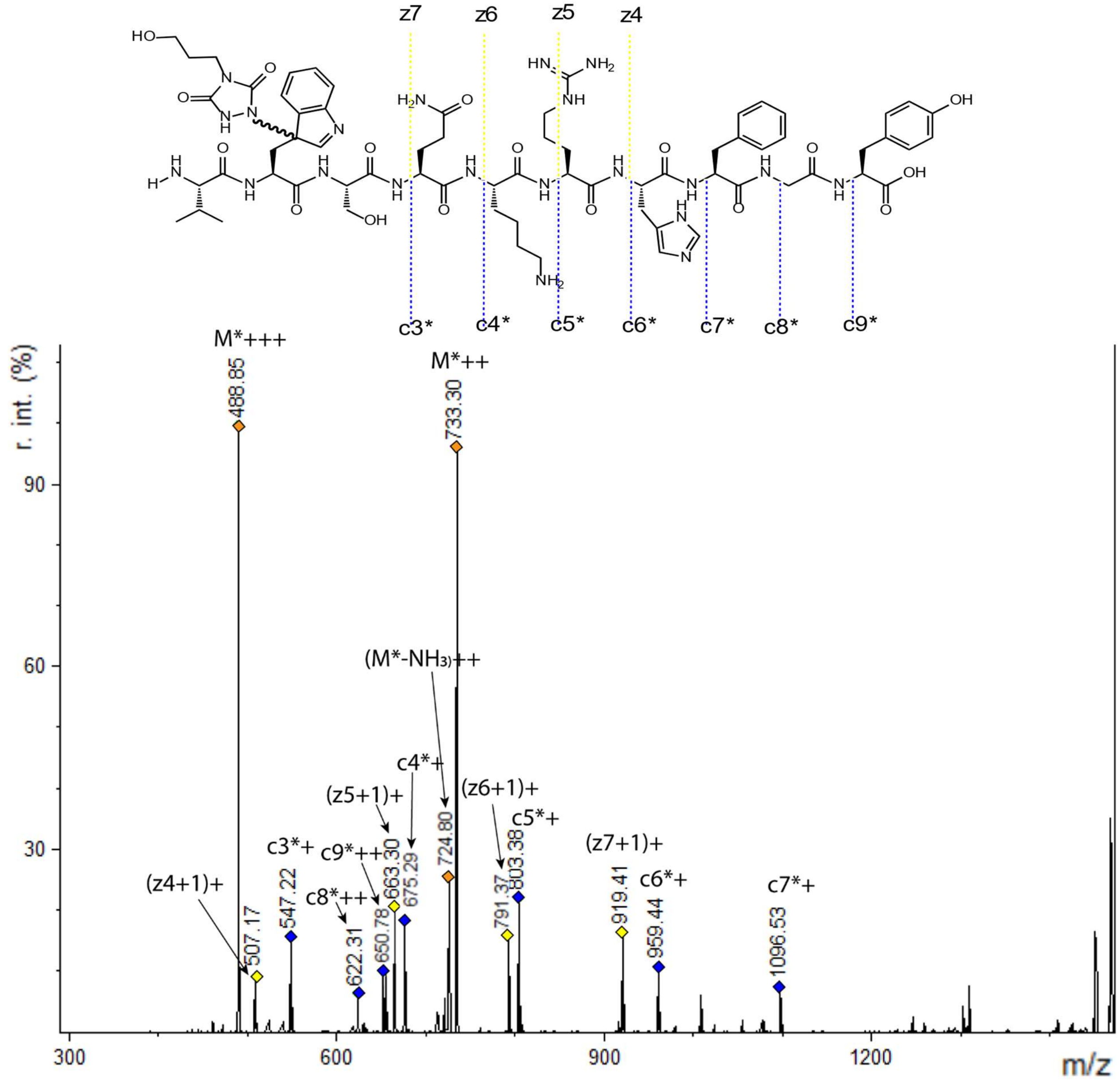

Figure 2.4.1.3 ETD MS/MS analysis at t_R = 47.16 min, precursor: TAD propanol modified peptide **1k** (488.91+++). Modification was done in 10 X PBS pH 4.

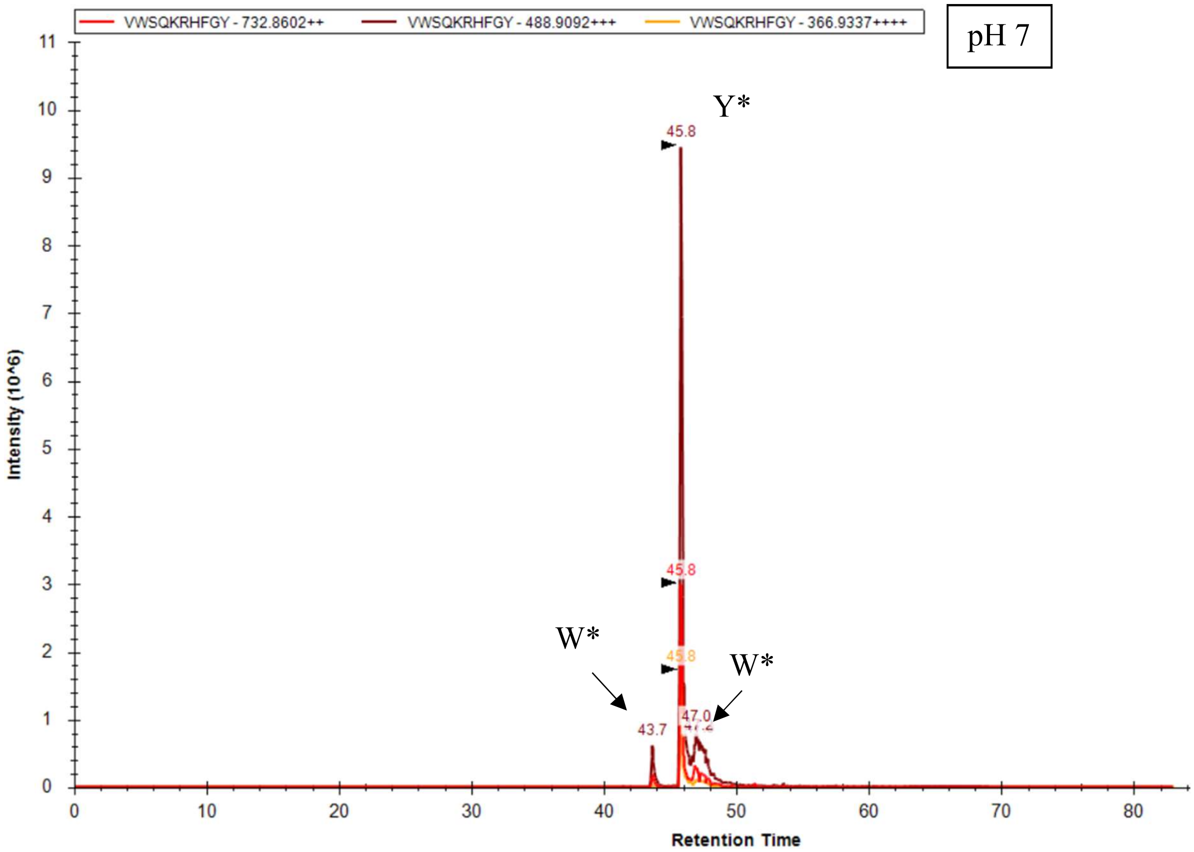

Figure 2.4.1.4 Ion chromatogram showing the double, triple and quadruple charged single TAD propanol modified peptide ions. The conjugation reaction of peptide **1k** with TAD-propanol **2b** was performed in 10 X PBS pH 7. The main peak is observed at t_R = 45.8 min and alongside this main peak 2 smaller peaks are observed at t_R = 43.7 min and 47.0 min. The ETD analysis of the triple charged modified peptide ion (488.91+++) in the main peak demonstrates tyrosine modification (see figure 2.4.1.4 below), however the smaller peaks t_R = 43.7 min and 47.0 min indicate modification on tryptophan.

The absence of a peak at t_R = 45.8 min in figure 2.4.1.1 (modification at pH 4) together with the ETD analysis pinpointing the TAD moiety to tryptophan for the peptides eluting at RT 43.62 and 47.16, confirms the chemo selectivity towards tryptophan at pH 4.

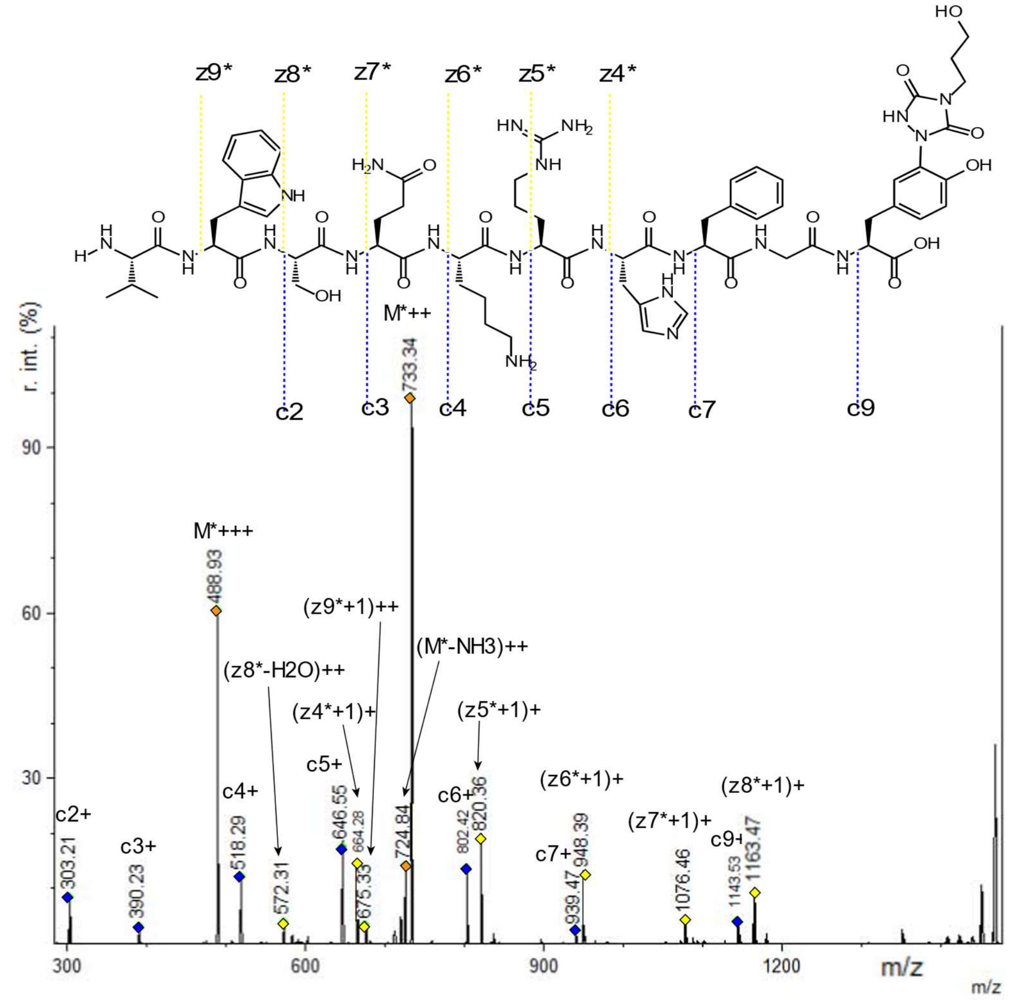

Figure 2.4.1.5 ETD MS/MS analysis at t_R = 45.82 min, precursor: TAD propanol modified peptide **1k** (488.91+++). Modification was done in 10 X PBS pH 7.

2.4.2 VWSNRHFY peptide **1i** and VYSNRHFW peptide **1j** with TAD-propanol **2b**.

The peptide modification reaction was performed according to the general protocol (section 1.3) but with 3 equivalents of TAD-propanol. The mixture was analyzed on Fusion Lumos as described above.

The graphs below show the intensity of the double and triple charged single TAD propanol modified peptide ion as a function of the retention time (Skyline software).

pH 4

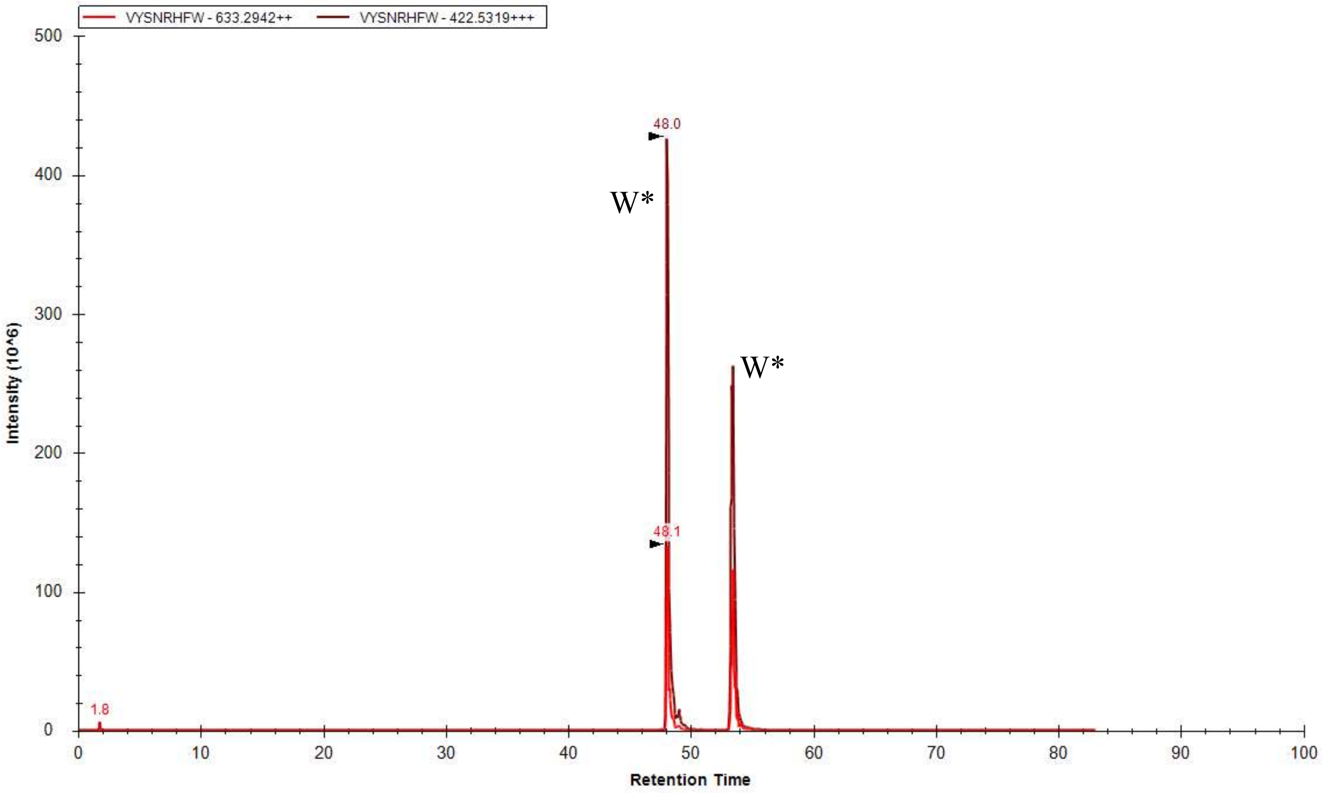

Figure 2.4.2.1 Ion chromatogram showing the double and triple charged single TAD propanol modified peptide ions. The conjugation reaction of peptide **1j** with TAD-propanol **2b** was performed in 10 X PBS pH 4. 2 peaks corresponding with the double and triple charged modified peptide ions at t_R = 48.0 min and at t_R = 53.5 min are observed. These two peaks correspond with both diastereomers of the TAD modified tryptophan product.

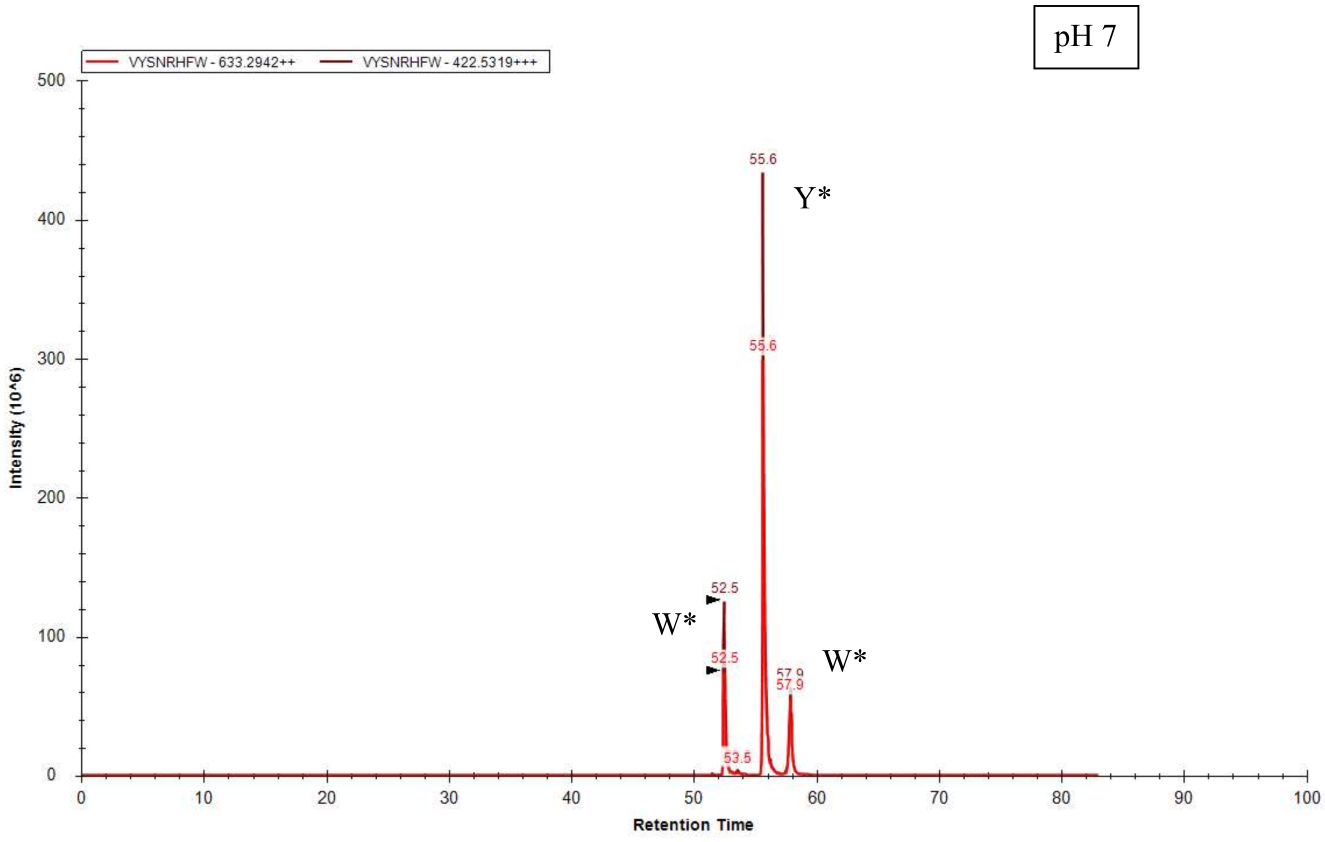

Figure 2.4.2.2 Intensity of the double and triple charged single TAD propanol modified peptide ion. The conjugation reaction of peptide **1j** with TAD-propanol **2b** was performed in 10 X PBS pH 7. 3 peaks are observed, 2 peaks correspond with the diastereomers of the TAD modified tryptophan product t_R = 52.5 min and at t_R = 57.9 min. the most intense peak corresponds with the TAD propanol modification located on tyrosine t_R = 55.6 min. Note that the retention times are slightly shifted due to the fact that the samples of pH 4 and pH 7 were measured on different columns.

The histogram below shows the peak area for the **doubly TAD propanol modified peptide ions** for peptide **1i** and peptide **1j**. The double (711.82++) and triple (474.88+++) charged ions of these peptides with 2 TAD-propanol moieties are the same for peptide **1i** and **1j** (only the relative position of Y and W is different for **1i** and **1j**). The peptide modification reaction was performed in either 10 X PBS pH 4 or 10 X PBS pH 7, according to the general protocol (section 1.3) but with 3 equivalents of TAD-propanol.

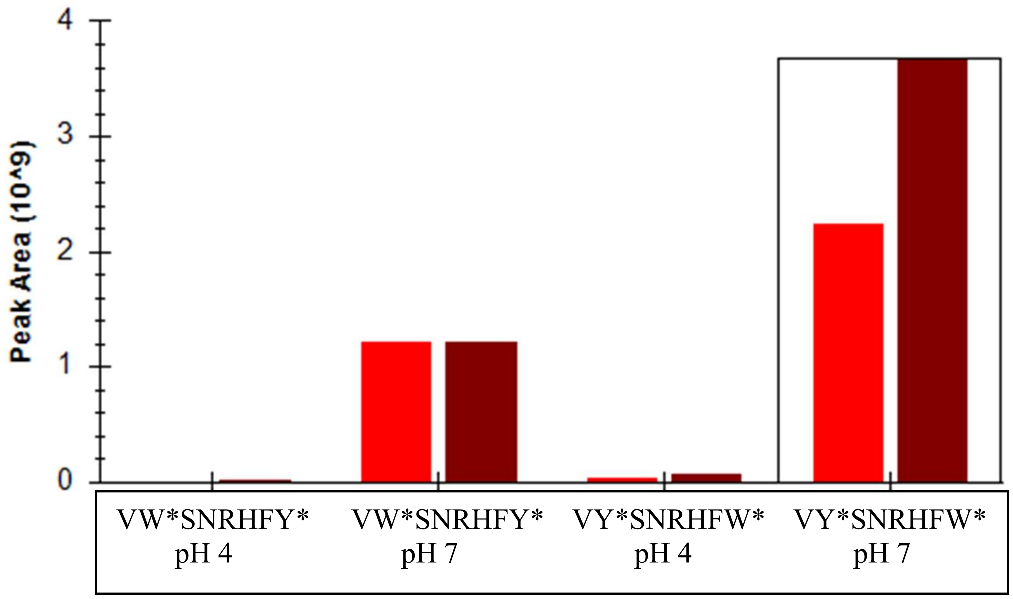

Figure 2.4.2.3 Histogram showing peak area for the double (711.82++, red) and triple (474.88+++, dark red) charged ions of these peptides with 2 TAD-propanol moieties.

To obtain a double TAD modified peptide both the tryptophan and the tyrosine in the peptide have to react with TAD. The fact that double TAD-propanol modified peptides are nearly absent at pH 4, and their presence at pH 7 further demonstrates the chemo selectivity for tryptophan at pH 4.

2.5 LC (ESI) ETD MS/MS

Previous experiments have shown that the TAD-tryptophan modification is not stable in MALDI-TOF/TOF experiments. Electron transfer dissociation (ETD) is a soft dissociation method that could preserve the TAD-tryptophan modification upon fragmentation. We have analyzed conjugation reactions with peptides **1i** and **1j** and 3 equivalents of TAD-propanol (**2b**) in 10 X PBS buffer at pH 4 and pH 7 on a Fusion™ Lumos™ mass spectrometer equipped with an ETD module. In ETD peptide fragmentation c and z ions are observed, the settings in the Mmass software were adapted to predict c and z ions as well as the molecular ions. Note that in ETD typically (z+1)+ ions are detected instead of the z+ ions because of hydrogen migragtion[4]. The triple charged molecular ion was selected as precursor ion for all ETD experiments. The results below clearly show that the combination of electrospray ionization (ESI) and ETD fragmentation is ideally suited to preserve and identify the TAD-tryptophan modification.

Conjugation product of Val-Trp-Ser-Asn-Arg-His-Phe-Tyr-OH (VWSNRHFY, 1i) with TAD-propanol 2b (2ib).

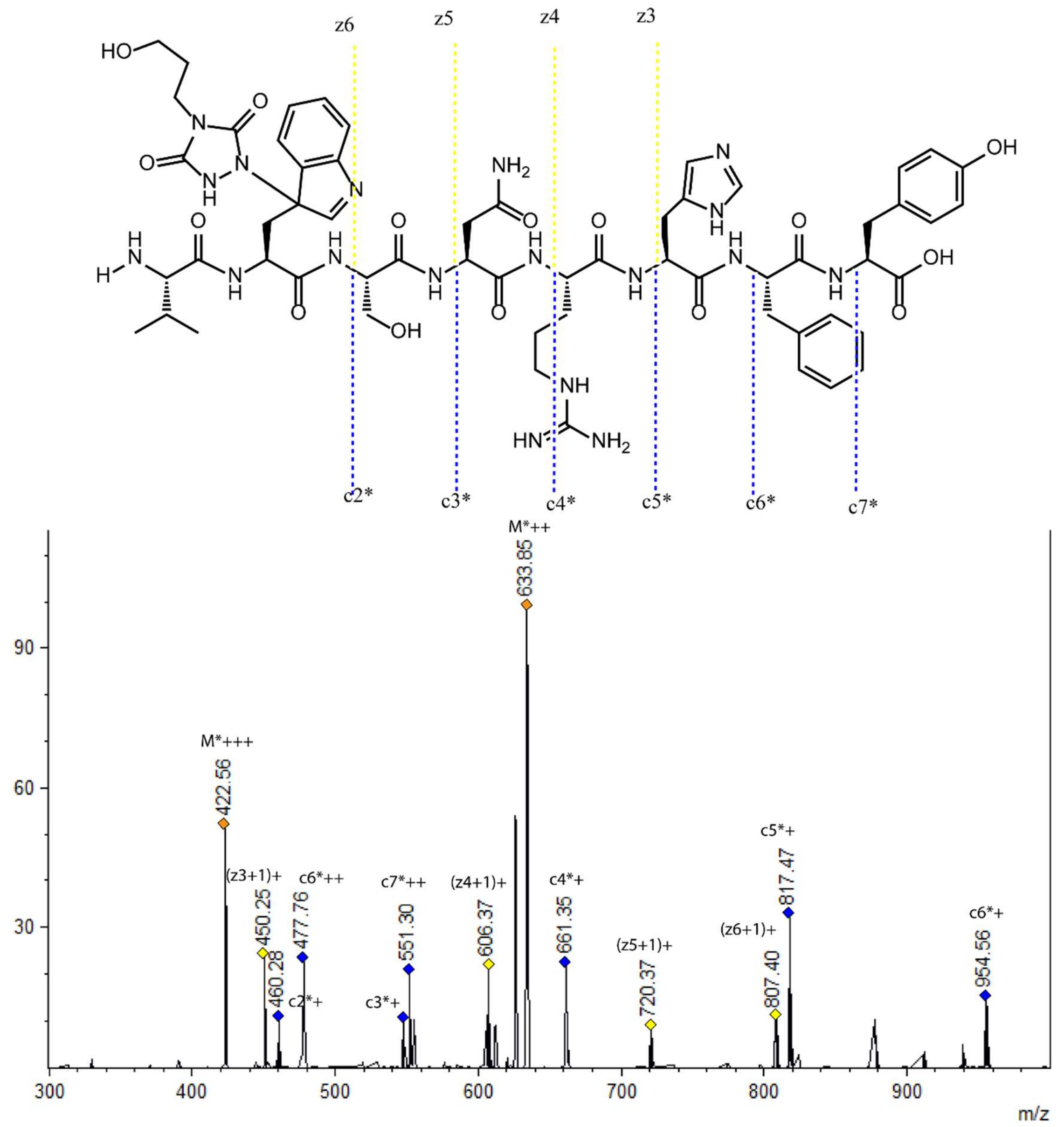

2.5.1 ETD MS/MS spectrum of peptide **1i** conjugated with **2b** (precursor ion: 422.53 +++). Chemical structure of peptide **1i** with detected fragment ions is shown. Fragments with "*" are TAD modified fragments. Conjugation reaction was performed in 10 X PBS pH 4.

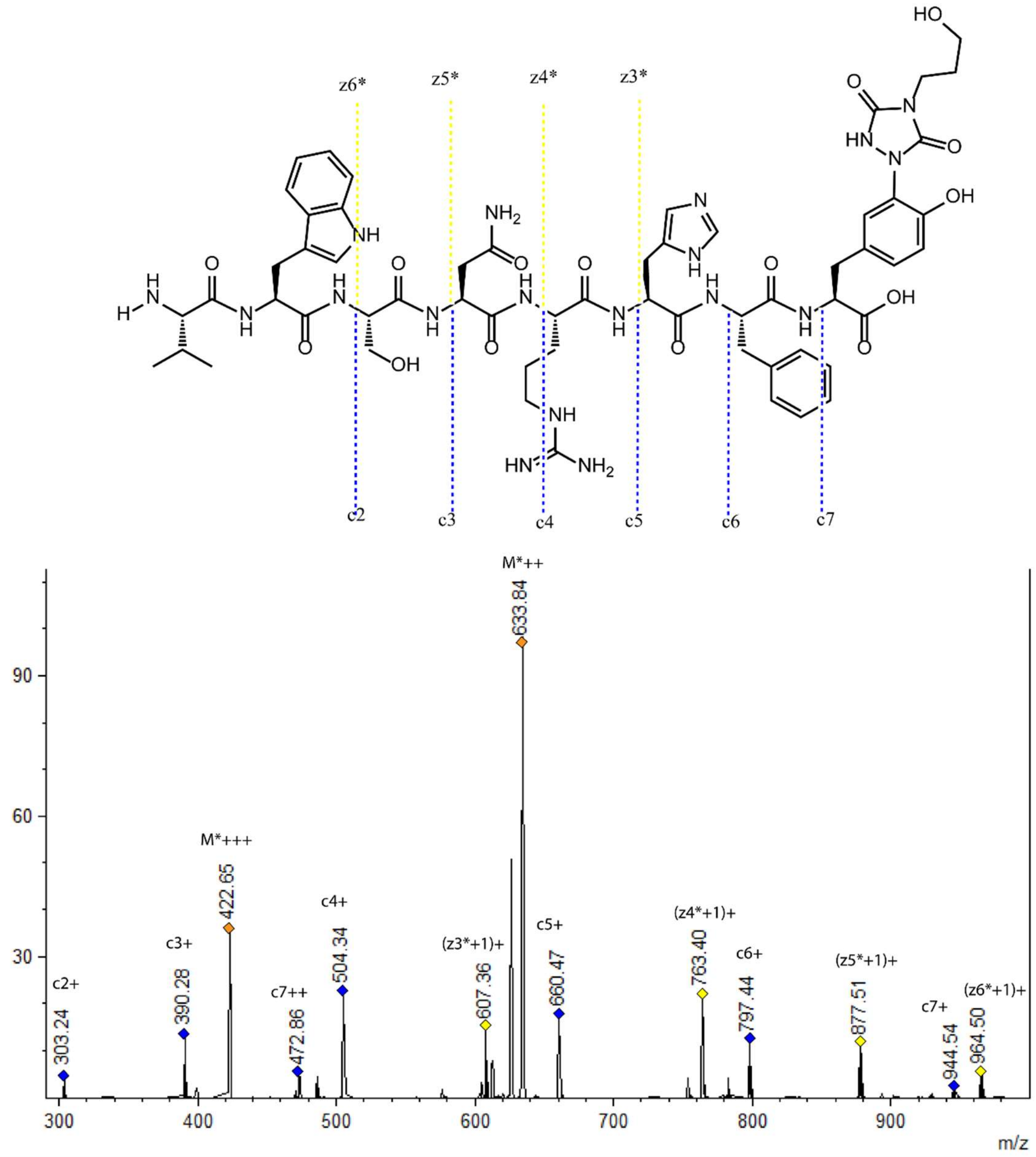

2.5.2 ETD MS/MS spectrum of peptide **1i** conjugated with **2b** (precursor ion: 422.53 +++). Chemical structure of peptide **1i** with detected fragment ions is shown. Fragments with "*" are TAD modified fragments. Conjugation reaction was performed in 10 X PBS pH 7.

Conjugation product of Val-Tyr-Ser-Asn-Arg-His-Phe-Trp-OH (VYSNRHFW, 1j) with TAD-propanol 2b (2jb).

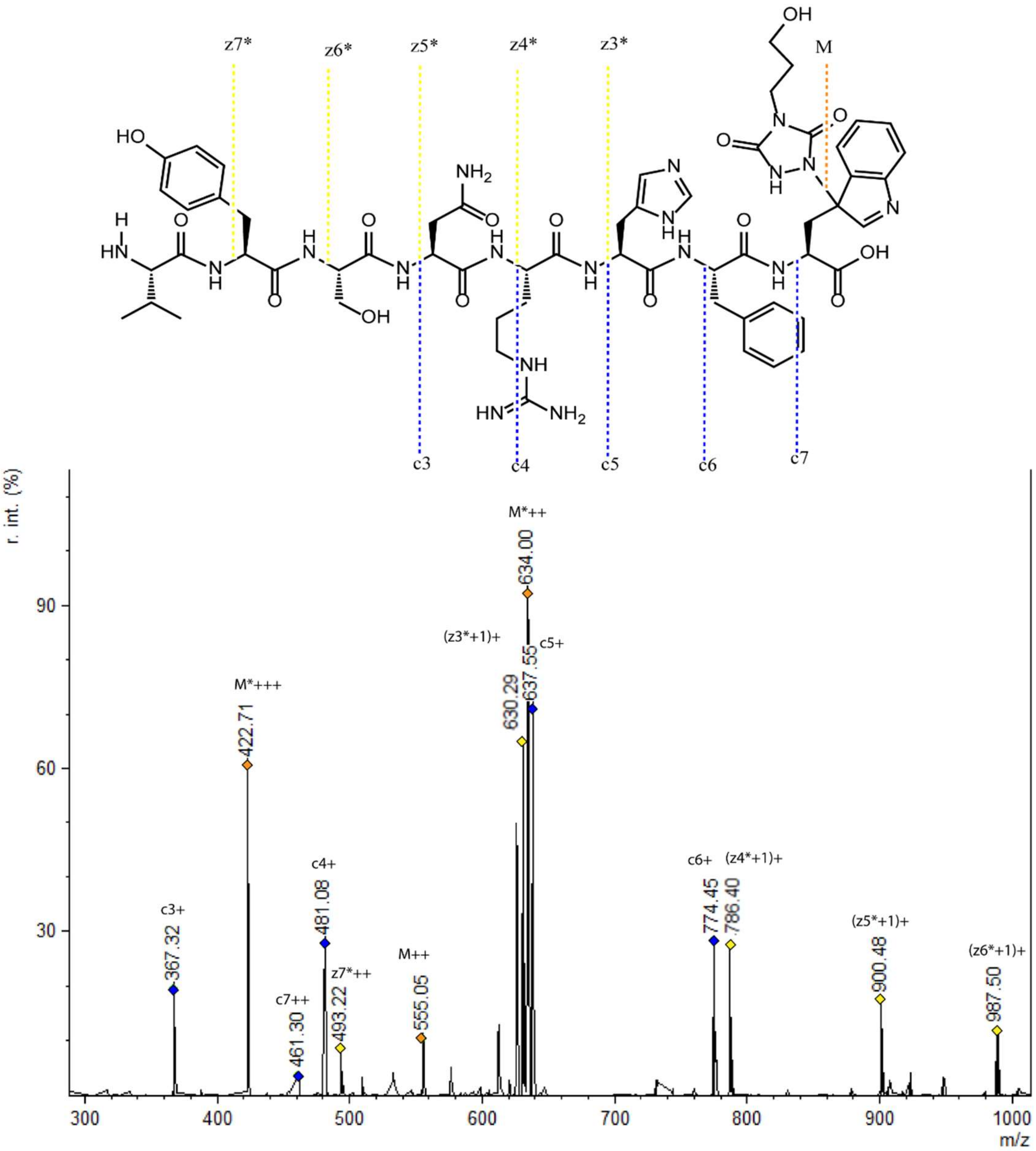

2.5.3 ETD MS/MS spectrum of peptide **1j** conjugated with **2b** (precursor ion: 422.53 +++). Chemical structure of peptide **1j** with detected fragment ions is shown. Fragments with "*" are TAD modified fragments. Conjugation reaction was performed in 10 X PBS pH 4.

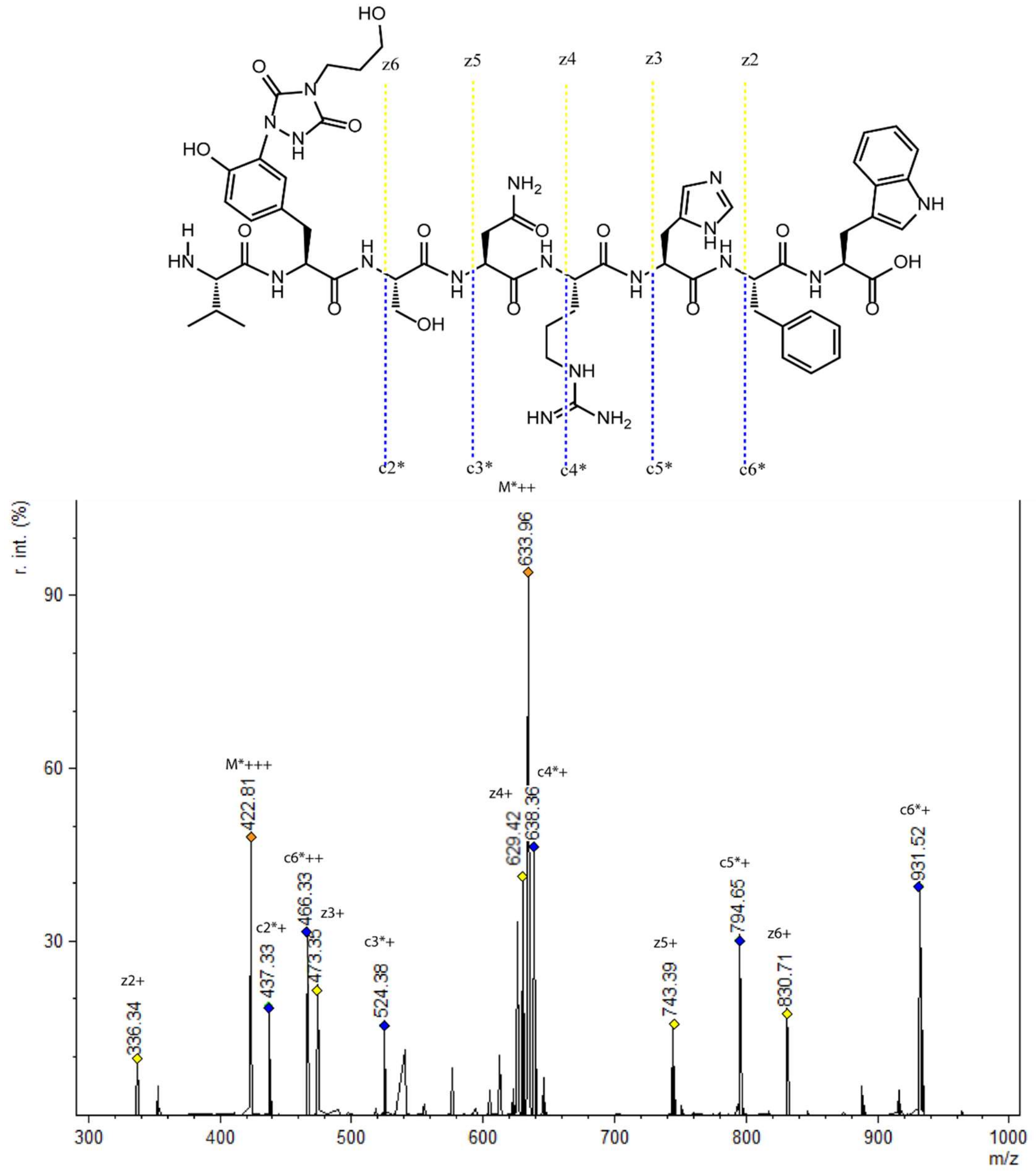

2.5.4 ETD MS/MS spectrum of peptide **1j** conjugated with **2b** (precursor ion: 422.53 +++). Chemical structure of peptide **1j** with detected fragment ions is shown. Fragments with "*" are TAD modified fragments. Conjugation reaction was performed in 10 X PBS pH 7.

2.6 LC (ESI) CID MS/MS

Since CID fragmentation is harsher compared to ETD, modifications are easily lost upon fragmentation. Furthermore, since the fragmentation energy applied is unique for the precursor ion including the modification, no further fragmentation is taking place on the precursor without the modification. Hence a low number of fragmentation ions is detected and identification cannot be established.

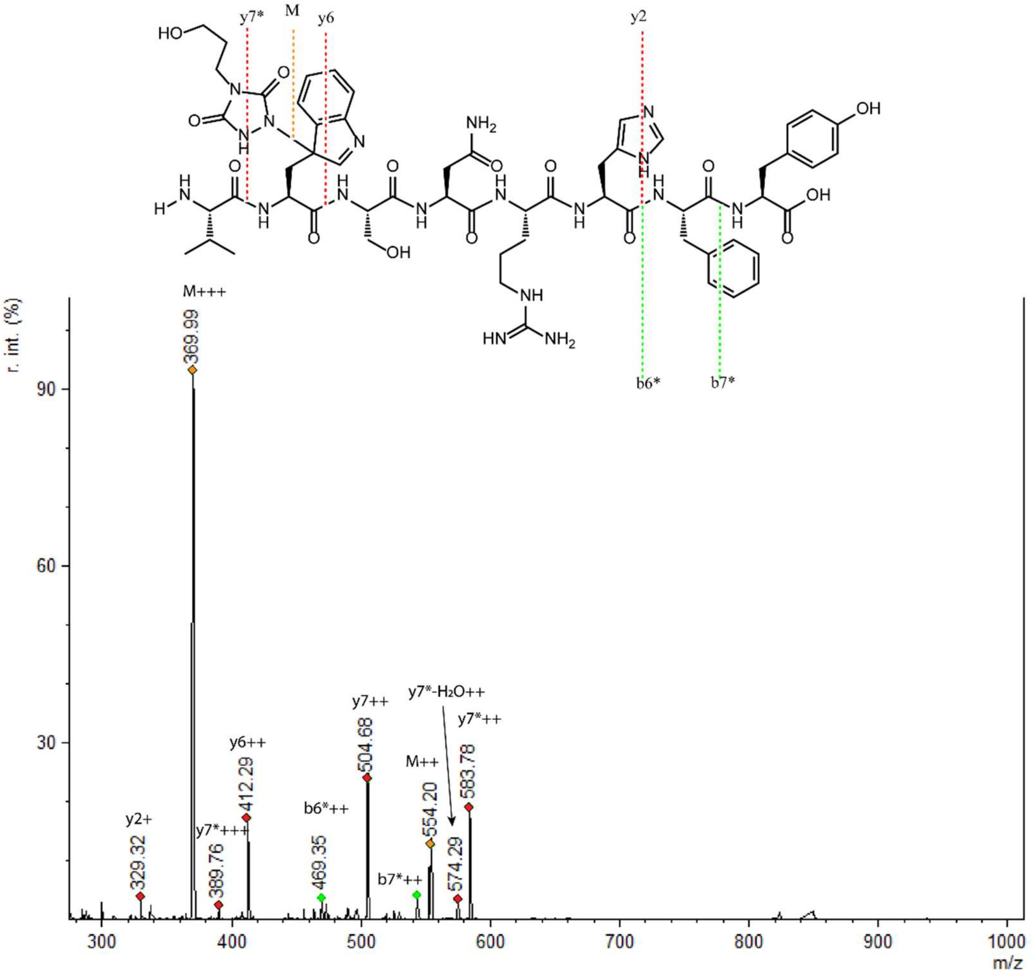

Figure 2.6.1 CID MS/MS spectrum of peptide **1i** conjugated with **2b** (precursor ion: 422.53 +++). Chemical structure of peptide **1i** with detected fragment ions is shown. Fragments with "*" are TAD modified fragments. Conjugation reaction was performed in 10 X PBS pH 4.

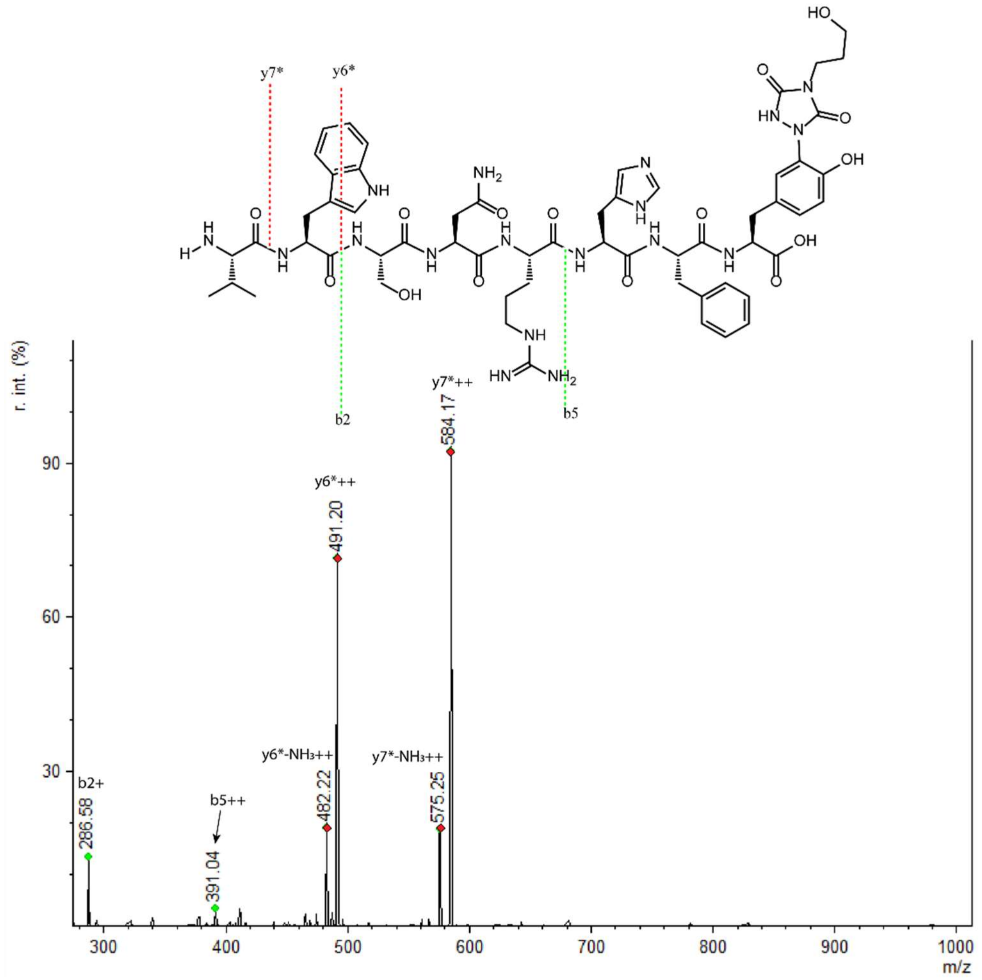

Figure 2.6.2 CID MS/MS spectrum of peptide **1i** conjugated with **2b** (precursor ion: 422.53 +++). Chemical structure of peptide **1i** with detected fragment ions is shown. Fragments with "*" are TAD modified fragments. Conjugation reaction was performed in 10 X PBS pH 7.

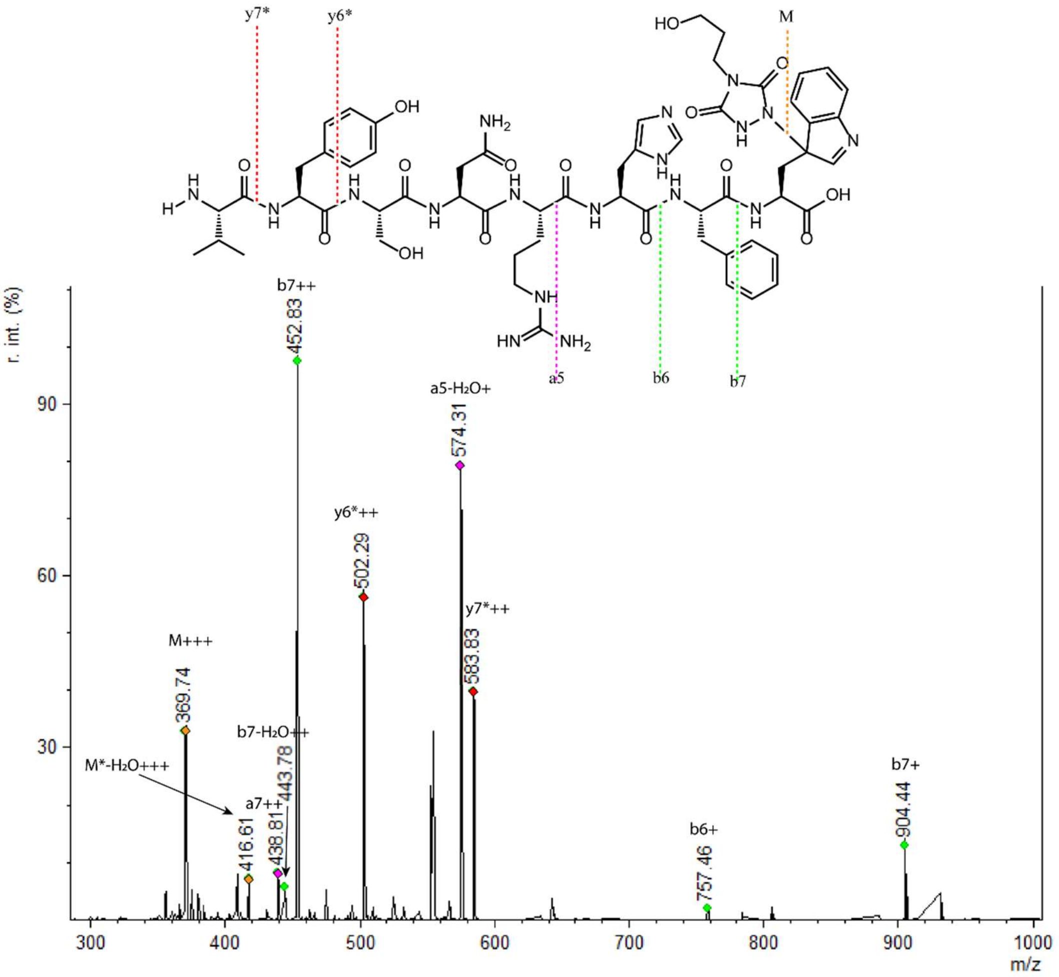

Figure 2.6.3 CID MS/MS spectrum of peptide **1j** conjugated with **2b** (precursor ion: 422.53 +++). Chemical structure of peptide **1j** with detected fragment ions is shown. Fragments with "*" are TAD modified fragments. Conjugation reaction was performed in 10 X PBS pH 4.

2.7 LC (ESI) HCD MS/MS

Since HCD fragmentation is using nitrogen as collision gas to enable fragmentation, a modification is easily lost upon fragmentation, but unlike CID fragmentation, the fragmentation of the precursor that lost the modification is still taking place. This results in a fragmentation spectrum containing fragment ions without the modification, although the precursor mass shows the presence of the modification.

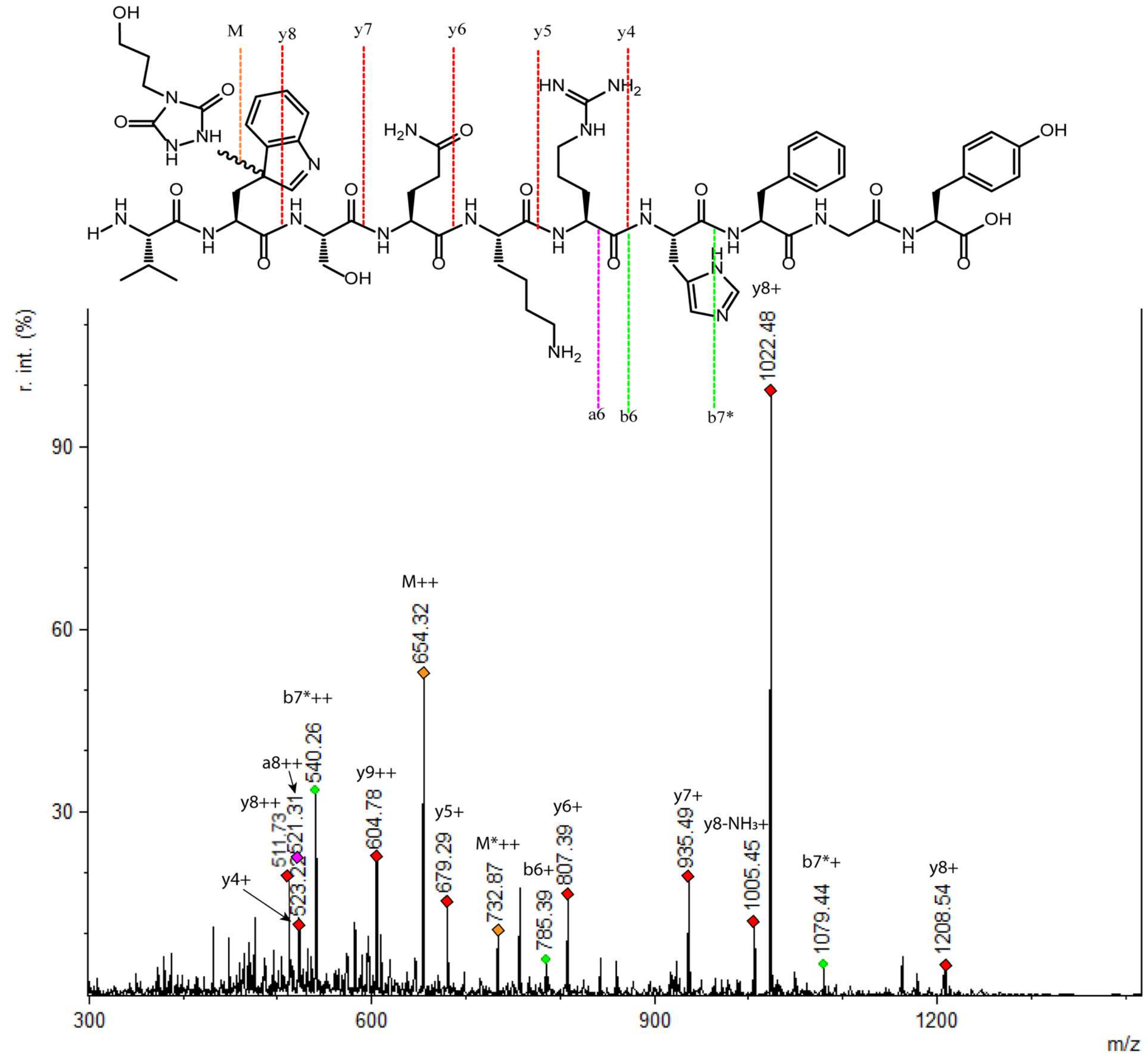

Figure 2.7.1 HCD MS /MS spectra of peptide **1k** conjugated with TAD-propanol **2b** (precursor ion: 732.86 ++). Chemical structure with detected fragment ions is shown. Fragments with "*" are TAD modified fragments. Conjugation reaction was performed in 10 X PBS pH 4.

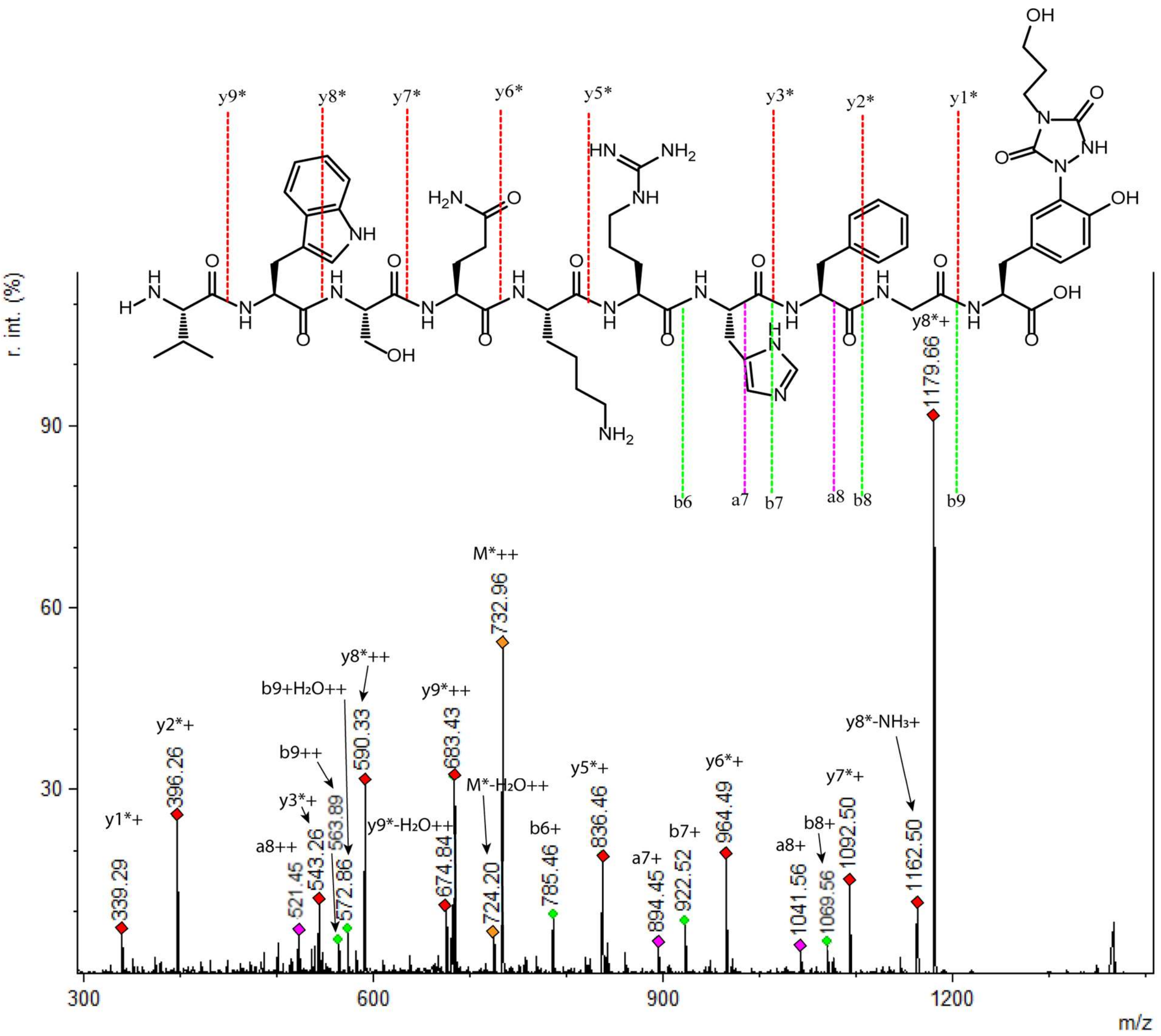

Figure 2.7.2 HCD MS /MS spectra of peptide **1k** conjugated with TAD-propanol **2b** (precursor ion: 732.86 ++). Chemical structure with detected fragment ions is shown. Fragments with "*" are TAD modified fragments. Conjugation reaction was performed in 10 X PBS pH 7.

2.8 pH dependent proteome wide tyrosine versus tryptophan selectivity

The proteome-wide selectivity of TAD towards tyrosine and tryptophan was examined using PTAD alkyne **2c** and a tryptic digest of a HeLa cellular proteome which was purchased from Pierce™. PTAD alkyne **2c** was prepared as described in section 1.2 using 1.3 mg TCCA and 4.8 mg PTAD-alkyne (urazole precursor), mixed in 500 μL MeCN and left shaking for 2 h at room temperature in the dark. 20 μg HeLa digest was dissolved in either 60 μL PBS pH 4 or PBS pH 7.2. For both pH settings, the sample was split in 3 x 20 μL. Following the oxidation reaction, the PTAD alkyne solution was diluted to a 2 mM solution in MeCN and 2 μL of this PTAD-alkyne solution was added to each of the 20 μL digest solutions (6 in total) and mixed by pipetting up and down. Before MS analysis on the Fusion Lumos system (procedure described in section 1.1), 2 μL of 10% TFA in water was added to acidify the samples for MS analysis.

In the search parameters, the PTAD alkyne modification was included as a variable modification that can occur both on tyrosine and tryptophan. For PTAD alkyne modification on tryptophan, we included the possibility of a neutral loss of the PTAD alkyne moiety upon HCD MS/MS analysis in the search, which ensures that the tryptophan-TAD modifications, which are present on the precursor peptide but fall off during HCD fragmentation, are detectable. Identifications at 99% confidence level were stored in ms-lims[5] for data management. The mass spectrometry proteomics data have been deposited to the ProteomeXchange Consortium via the PRIDE [1] partner repository with the dataset identifier PXD031607 and 10.6019/PXD031607.

Username: reviewer_pxd031607@ebi.ac.uk

Password: GVxqVX4y

When PTAD alkyne was added to the HeLa digest dissolved in PBS pH 7.2, we found that around 37% of the tyrosines in uniquely identified peptides were TAD modified, and that about 3% of the tryptophans in uniquely identified peptides were also TAD modified (figure 2.8.1).

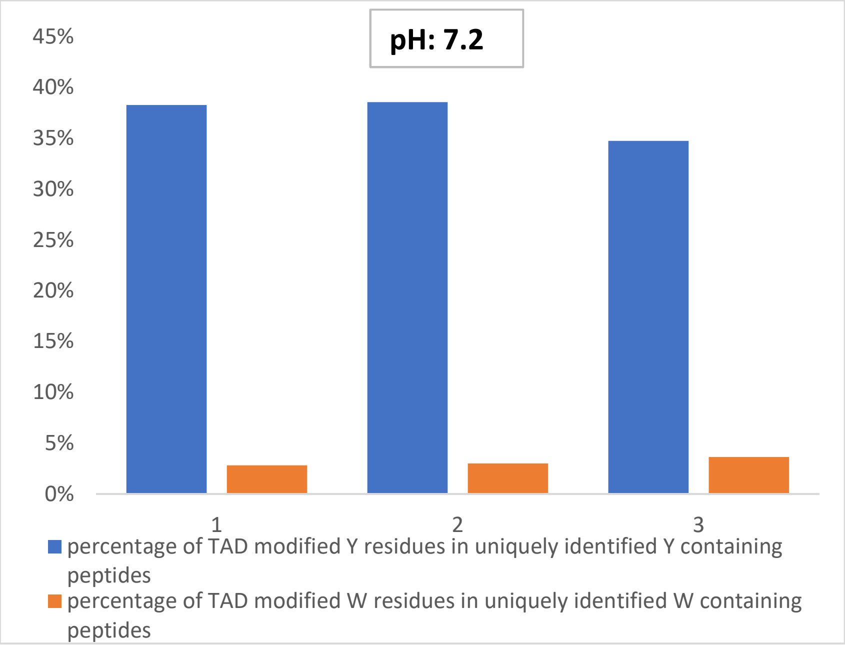

Figure 2.8.1 Bar graph representation of the percentage of TAD-modified tyrosine (blue) or tryptophan (orange) residues observed in uniquely identified peptides. TAD modification reaction performed in triplicates on HeLa digests at pH 7.2.

Remarkably, if the HeLa digest was dissolved in PBS pH 4 and the PTAD alkyne was added, the results are entirely different. At this pH, 11% of the tryptophans and only 0.09% of the tyrosines in uniquely identified peptides were TAD modified.

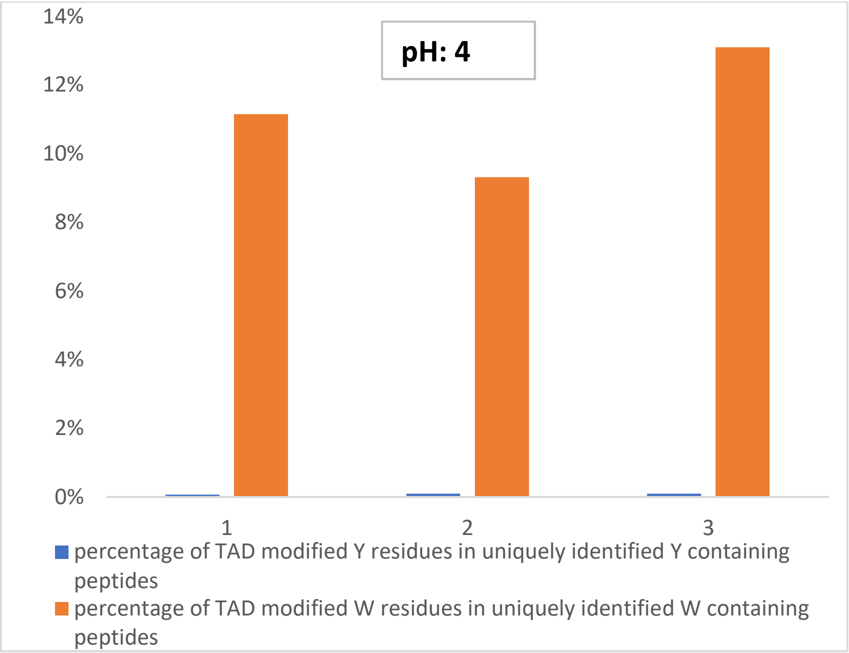

Figure 2.8.2 Bar graph representation of the percentage of TAD-modified tyrosine (blue) or tryptophan (orange) residues observed in uniquely identified peptides. TAD modification reaction performed in triplicates on HeLa digests at pH 4.

For each of the individual MS runs (six in total), peptide spectral matches (PSM), accessions (proteins), total amount of uniquely identified peptides (u.p.), Y-TAD modified u.p., Y containing u.p., % of Y-TAD in Y containing u.p., W-TAD modified u.p., W containing u.p. and % of W-TAD in W containing u.p. are presented in table 2.8.1.

Table 2.8.1 MS analysis data overview for each of the individual runs

replicate	spectra	accessions	total u.p.	Y<TAD> u.p.	Y u.p.	% Y<TAD> in Y containing u.p.	W<TAD> u.p.	W u.p.	% W<TAD> in W containing u.p.
pH4_1	19183	2480	9244	2	2934	0.07%	64	574	11.15%
pH4_2	19914	2524	9586	3	2997	0.10%	57	612	9.31%
pH4_3	18916	2457	9264	3	2975	0.10%	73	557	13.11%
pH7.2_1	19090	2351	8976	889	2323	38.27%	23	813	2.83%
pH7.2_2	19520	2366	9096	887	2301	38.55%	24	800	3.00%
pH7.2_3	19344	2434	9271	854	2459	34.73%	31	853	3.63%

3. Protein conjugation

3.1 Bovine serum albumin (reinterpretation of data from Vandewalle *et al.*)

In the manuscript of Vandewalle *et al.*[6] bovine serum albumin (BSA) is conjugated with butyl-TAD in PBS/ACN (0.28/0.72), according to the procedure described by Barbas and co-workers[7]. This was done to certify that the triazolinedione is reacting in an orthogonal way through an electrophilic aromatic substitution (S_EAr) with the phenol functionality of tyrosine residues. In this experiment 20 equivalents of butyl-TAD were used. The BSA starting material (Figure 3.3.1) and BSA-TAD conjugate (Figure 3.3.2) were subjected to a trypsin digestion followed by MALDI-TOF analysis. The raw MALDI-TOF data were provided by the authors and reinterpreted using the mMass software to predict tryptic fragments. Butyl-TAD modifications both on tyrosine and tryptophan residues were programmed in the software.

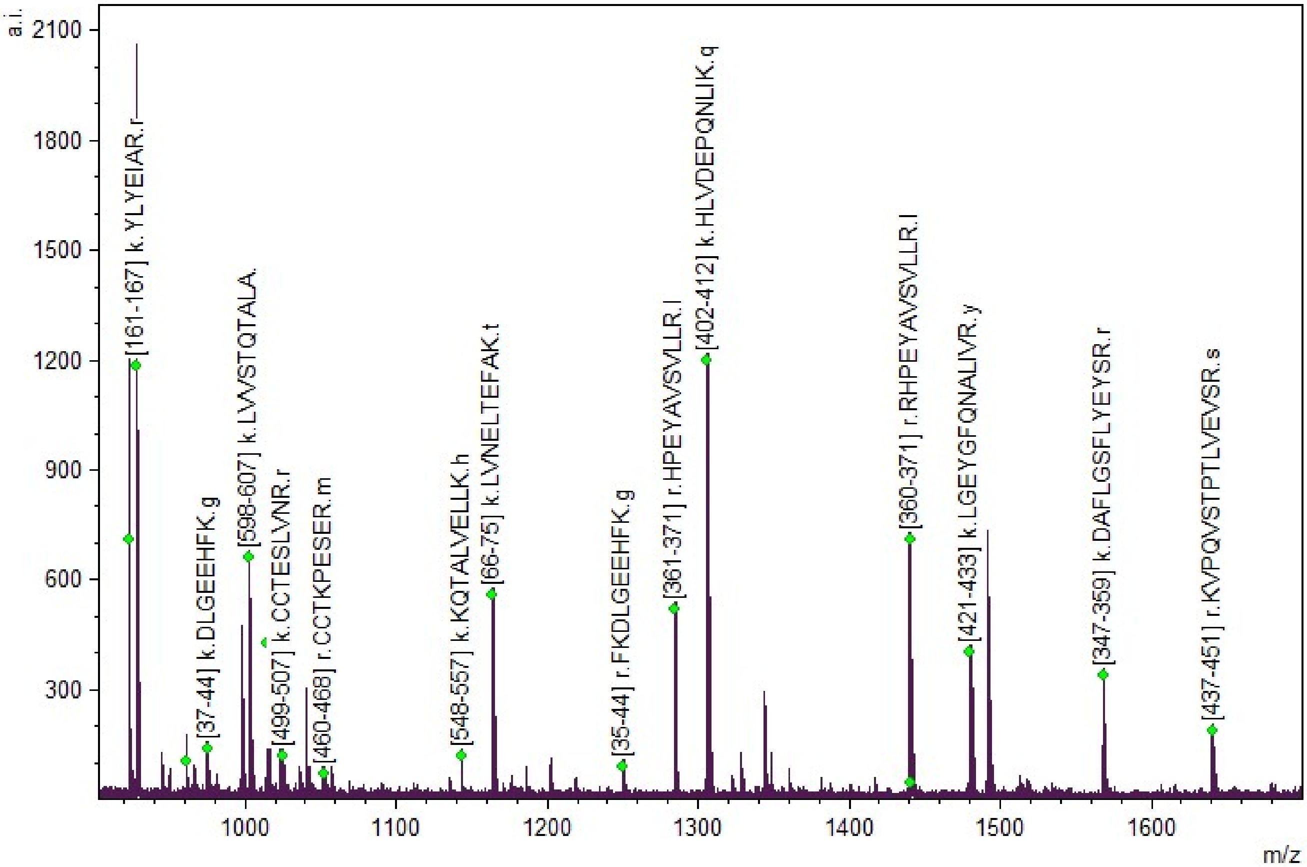

Figure S3.1.1 MALDI-TOF MS spectrum of the tryptic digest of BSA starting material. The detected tryptic peptides that were matched with predicted tryptic fragments are annotated.

Several tryptic fragments are detected and matched with predicted tryptic peptides. As was expected for the starting material no fragments with a butyl-TAD modification were detected in the spectrum.

Tryptic digest of BSA with butylTAD

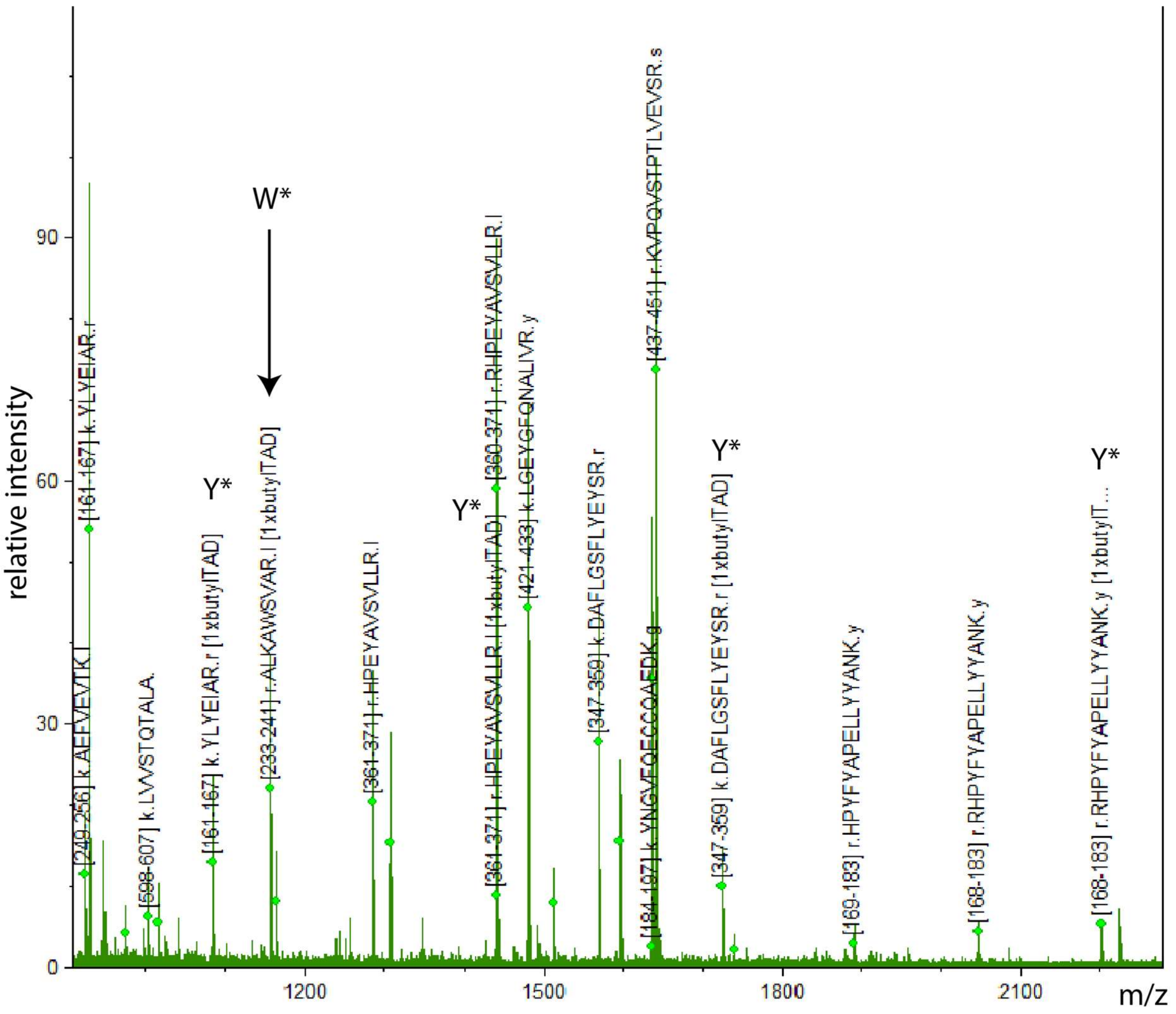

Tryptic peptide: ALKAWSVAR modified with butylTAD

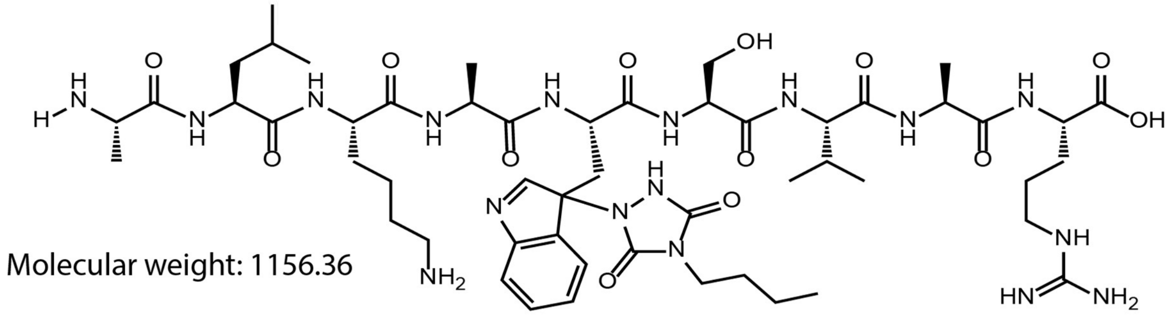

Figure S3.1.2 MALDI-TOF MS spectrum from a tryptic digest of BSA after reaction with butyl-TAD in PBS/MeCN (0.28/0.72) (top). Chemical structure of a tryptic peptide modified with butyl-TAD (bottom). These data originate from Vandewalle et Al. [2] and were reinterpreted. A peptide fragment indicated with a Y* or W* marks a fragment with a TAD modification. As described in the aforementioned article several tryptic peptides modified with butyl-TAD were detected (Y*), all these modified peptides contain at least one tyrosine residue and no tryptophan. However, after careful reanalysis also a butyl-TAD modified tryptic fragment containing one tryptophan and no tyrosine residue was observed (W*) corresponding with the peptide fragment ALKAWSVAR.

The original data were interpreted by identification of peptides in the MS spectrum of the BSA starting material (S3.1.1) and searched for the butyl-TAD modified variants of these peptides in the MS spectrum of the modified BSA. Indeed, several peptides found in the starting BSA MS spectrum have a variant in the modified BSA MS spectrum corresponding with a mass addition of 155 Da. Four peptides were selected that are subject to the mass addition of 155 Da in the MS spectrum of modified BSA. Interestingly these peptides all contain at least one tyrosine but no tryptophan. Two of these selected peptides were further characterised with MS/MS to allocate the butyl-TAD modification unambiguously to the tyrosine residue. However, in this approach, possible modification on tryptophan was not taken into account and not searched for during interpretation of the MS spectra. Reinterpretation of the butyl-TAD modified BSA MALDI-TOF data with mMass software, while actively searching for butyl TAD modification on tyrosine and tryptophan yielded a surprising result. The same four tyrosine containing peptides that were selected in the initial analysis are found along with an additional butyl TAD modified peptide. The tryptic fragment ALKAWSVAR with butyl TAD modification was detected and contains a tryptophan residue and no tyrosine. This peak at 1156 Da was even the most abundant among the modified peptides. This was likely missed in the initial analysis due to the fact that the ALKAWSVAR peptide (without butyl TAD) was not observed in the MS spectrum of the starting BSA.

3.2 Valentine alphabody

The valentine alphabody is a recombinantly produced protein of 11.5 kDa. The alphabody protein has a triple helix coiled coil structure.[8] In the primary sequence of this alphabody there are three tyrosine residues and zero tryptophan residues. In view of the presence of a solvent exposed cysteine residue, the alphabody occurs as a dimer under non-reducing conditions. This implies that in the alphabody dimer (connected via a disulfide linkage) there are 6 tyrosine residues. The experiments with the valentine alphabody were measured on Agilent 1100 series LC (ESI) MS equipment. LC-MS analysis of the valentine alphabody dimer by MS (ESI): m/z 22937 (calcd $[M+H]^+$ 22941).

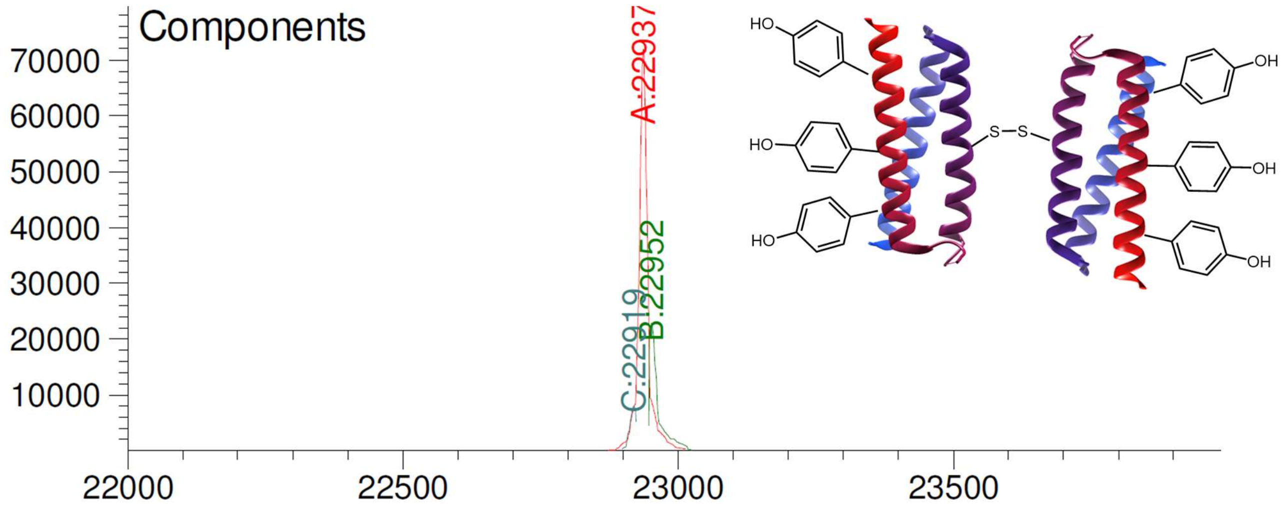

Figure S3.2.1 Deconvoluted mass spectrum from the LC-MS analysis of the valentine alphabody dimer. Cartoon representation of the alphabody dimer is shown, highlighting the bridging sulfur bond and the presence of the 6 tyrosine residues on the dimer. In addition to the peak corresponding to the alphabody dimer (22937 Da), also an oxidation product (22952 Da) and dehydration product (22919 Da) is observed.

A competition experiment was performed using a mixture of this alphabody dimer and the small tetrapeptide NSAW (**1c**). 10 µL of alphabody stock solution (4.6 mg/mL) in 10 X PBS buffer at pH 4 was added to 7 µL of 10 X PBS buffer at pH 4 and 2 µL of peptide **1c** 3 mM in water (3 eq of peptide compared to protein). Note that the ratio of tyrosine to tryptophan is 2 to 1. Subsequently, 3 eq. of TAD propanol **2b** were added. The reaction mixture was analysed with LC-MS (ESI) (Figure S3.2.2).

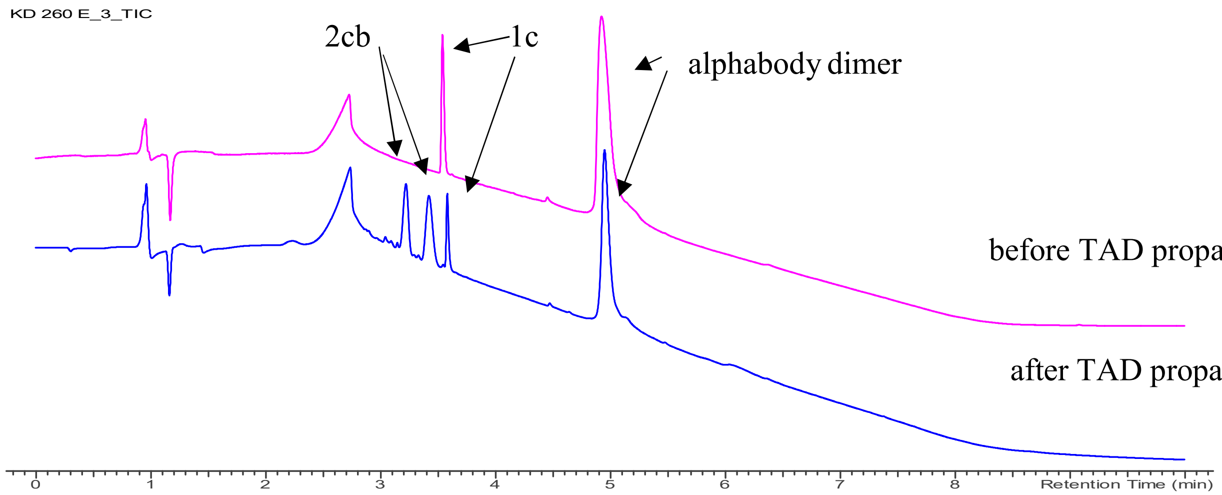

Figure S3.2.2 LC-chromatogram (214 nm) overlay of the competition experiment between TAD-propanol **2b** and a mixture of alphabody dimer and peptide **1c** in 10 X PBS pH 4. Starting mixture of **1c** and alphabody dimer before addition of TAD propanol (pink), after reaction (blue).

As shown in figure S3.2.2, peptide **1c** is largely consumed and 2 new peaks appear as expected for a TAD-adduct on a C-terminal tryptophan (*vide supra*). Peak integration of the chromatogram demonstrated a conversion of over 80% for peptide **1c**. Deconvolution of the protein peak at t_R 5 min is required to examine the effect on the alphabody dimer (Figure S3.2.3). Analysis of this result revealed no formation of any alphabody conjugate at pH 4 in accord with the absence of a Trp residue in this alphabody.

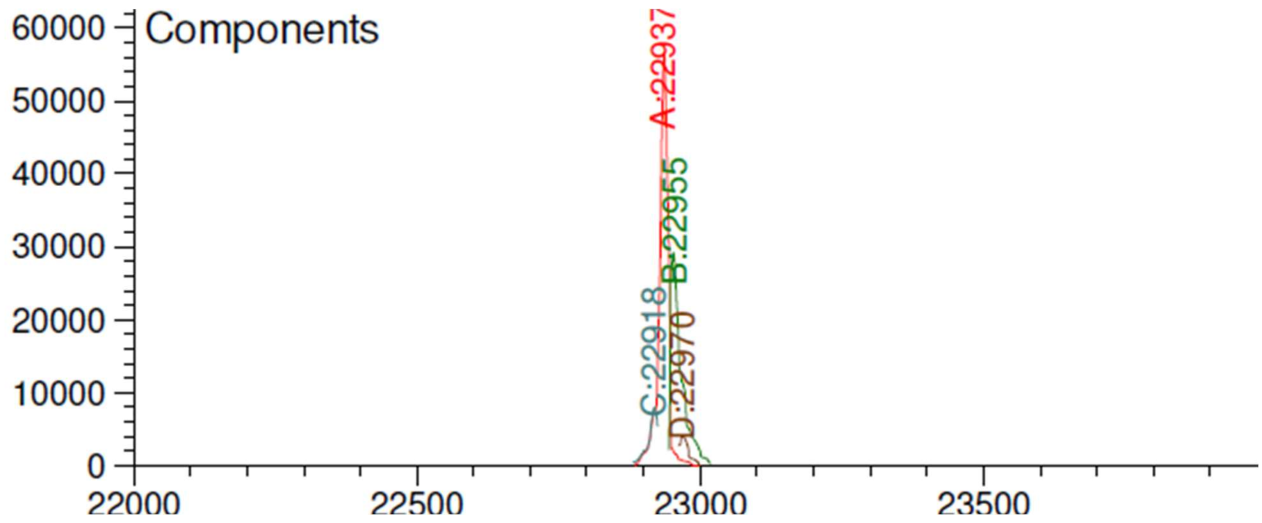

Figure S3.2.3 Deconvoluted mass spectrum of the peak between t_R 4.869 min and 5.085 min from the LC chromatogram in Figure S3.2.2. Only starting alphabody dimer is observed with a dehydration product (22918 Da) and a product with additional water (22955 Da) as well as a small amount of a product with an additional water + oxidation (22970 Da).

A competition experiment was performed using a mixture of this alphabody dimer and the small tetrapeptide NSAW (**1c**). 10 µL of alphabody stock solution (4.6 mg/mL) in 10 X PBS buffer at pH 7 was added to 7 µL of 10 X PBS buffer at pH 7 and 2 µL of peptide **1c** 3 mM in water (3 eq of peptide compared to protein). Note that the ratio of tyrosine to tryptophan is 2

to 1. Subsequently, 3 eq. of TAD propanol **2b** were added. The reaction mixture was analysed with LC-MS (ESI) (Figure S3.2.4).

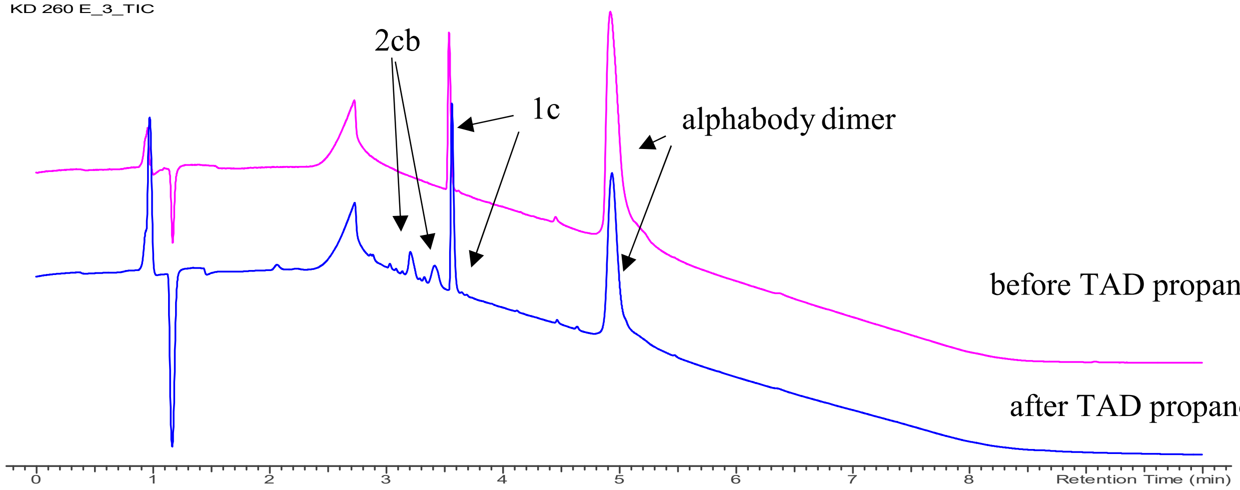

Figure S3.2.4 LC-chromatogram (214 nm) overlay of the competition experiment between TAD-propanol **2b** and a mixture of alphabody dimer and peptide **1c** in 10 X PBS pH 7. Starting mixture of **1c** and alphabody dimer before addition of TAD propanol (pink), after reaction (blue).

As shown in figure S3.1.2, peptide **1c** is partly consumed and 2 new peaks appear as expected for a TAD-adduct on a C-terminal tryptophan (*vide supra*). Peak integration of the chromatogram demonstrated a conversion of 30 % for peptide **1c**. Deconvolution of the protein peak at t_R 5 min is required to examine the effect on the alphabody dimer (Figure S3.2.5). Analysis of this result revealed formation of just under 30 % of alphabody TAD propanol conjugate at pH 7.

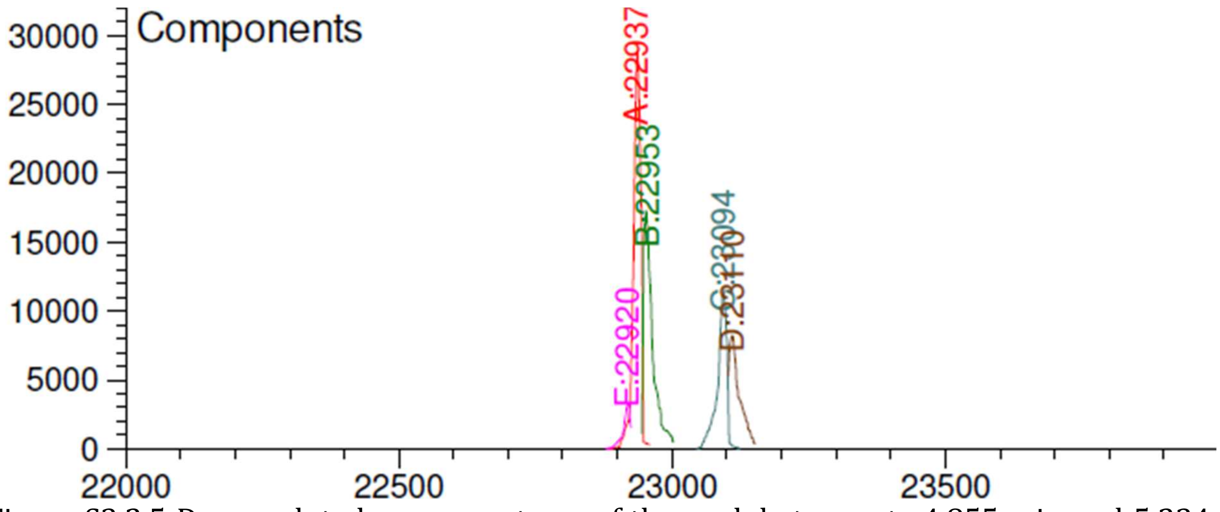

Figure S3.2.5 Deconvoluted mass spectrum of the peak between t_R 4.855 min and 5.234 min from the LC chromatogram in Figure S3.2.4. The expected mass for modification of the alphabody dimer with TAD-propanol **2b** is 23094 Da (found 23094 Da and oxidation product (+16 Da) 23110 Da).

Two control experiments were performed with the alphabody dimer in 10 X PBS buffer at pH 4 (figure 3.2.6) or pH 7 (figure 3.2.7) and TAD propanol (**2b**). 10 µL of alphabody stock solution (4.6 mg/mL) in 10 X PBS buffer at pH 4 or 7 was added to 9 µL of 10 X PBS buffer at pH 4 or 7 respectively. Subsequently, 8 eq. of TAD propanol **2b** with respect to each tyrosine (48 eq. for each alphabody dimer) were added. The reaction mixture was analysed with LC-MS (ESI). In the figures below the deconvoluted mass spectra from the alphabody dimer peak are presented. The data demonstrate that at pH 4 no alphabody TAD propanol (**2b**) conjugate is detected, at pH 7 43 % has at least one TAD propanol (**2b**) modification and over 10 % has two TAD propanol (**2b**) modifications.

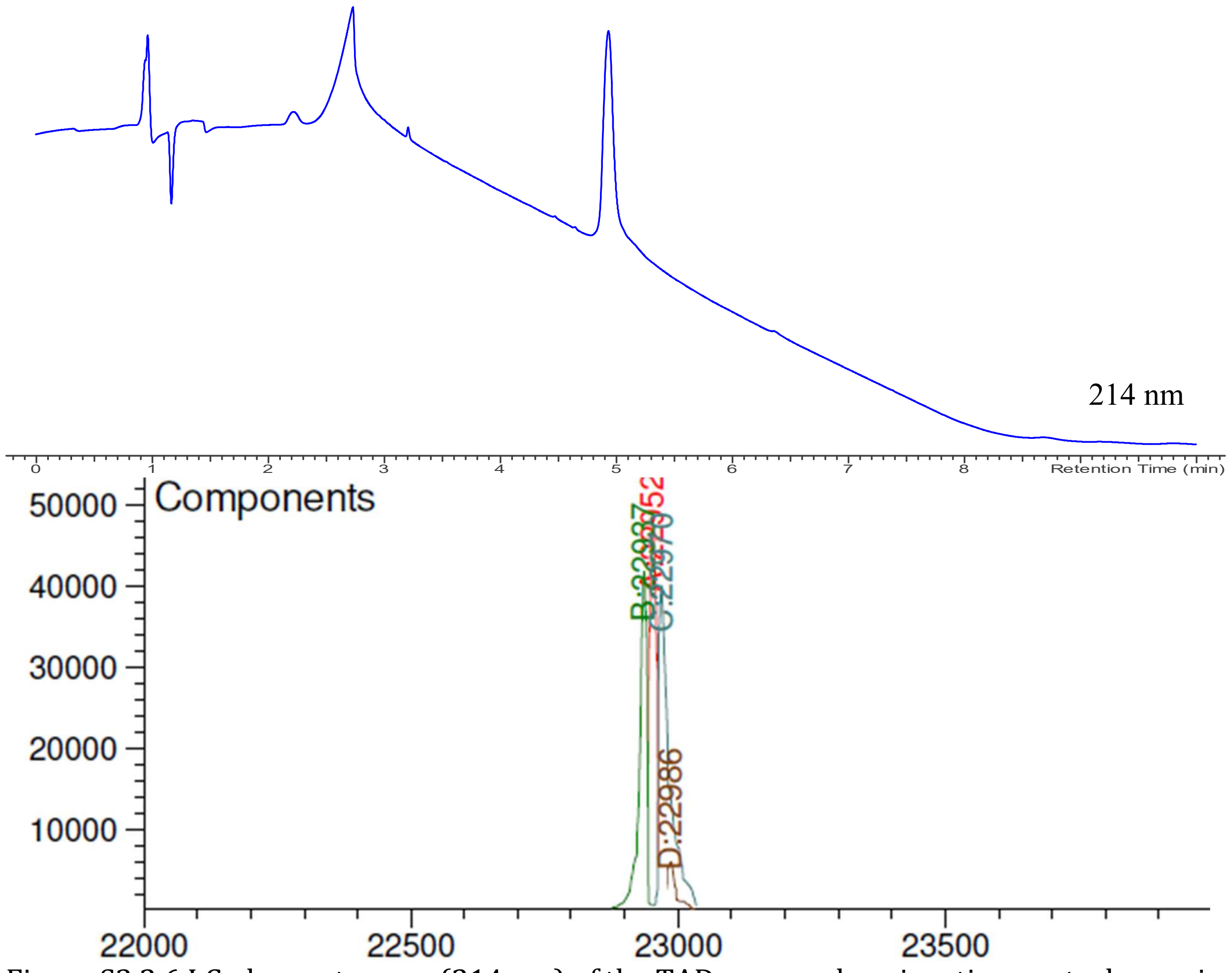

Figure S3.2.6 LC-chromatogram (214 nm) of the TAD propanol conjugation control experiment with the alphabody dimer in 10 X PBS buffer at pH 4 (top). Deconvoluted mass spectrum of the peak between t_R 4.856 min and 5.064 min (bottom). Only starting alphabody dimer (22937 Da) is observed with an oxidation product (22952 Da) and oxidation product with an additional water (22970 Da) as well as a small amount of an product with an additional water + double oxidation (22986 Da) no alphabody TAD conjugate is observed at pH 4.

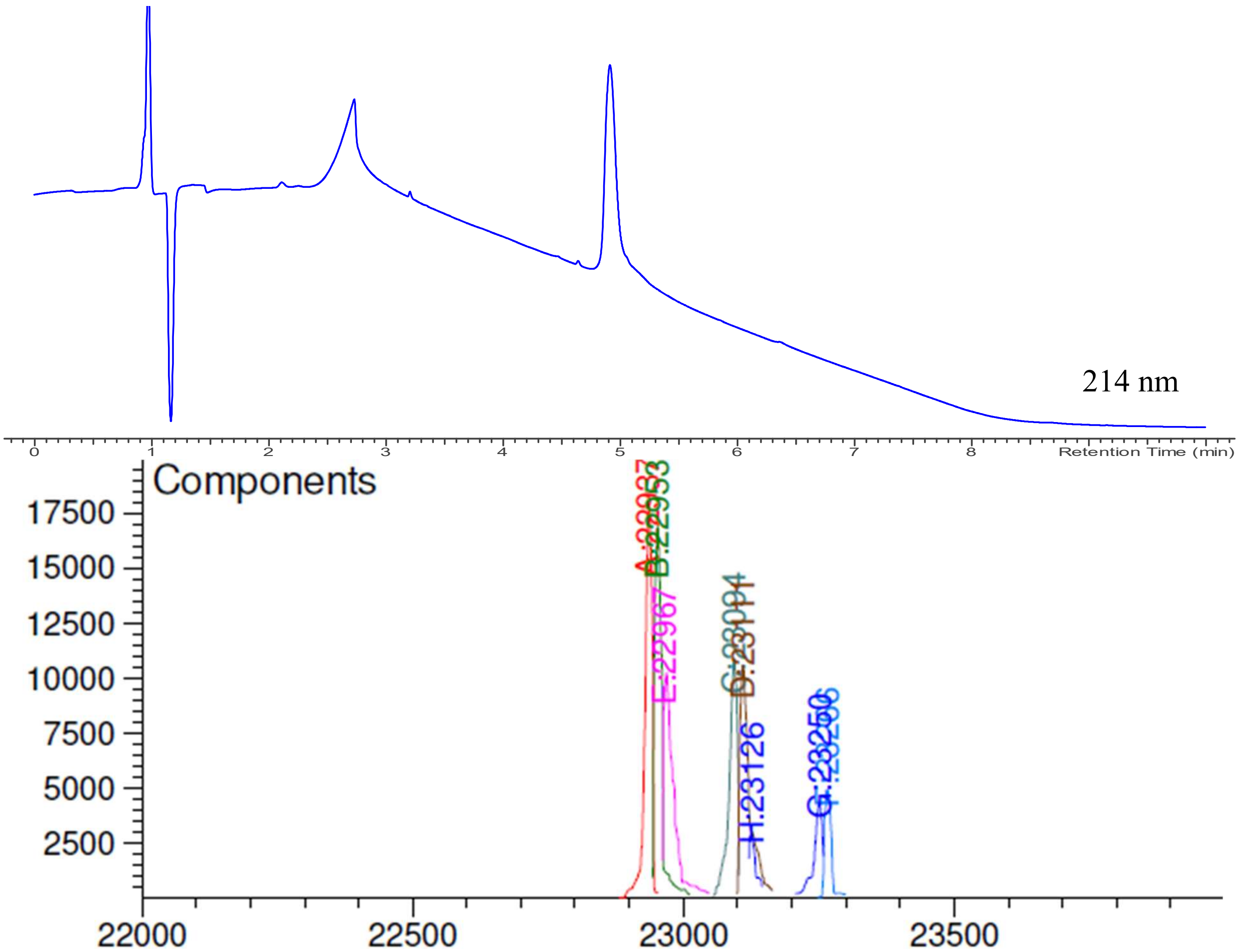

Figure S3.2.7 LC-chromatogram (214 nm) of the TAD propanol conjugation control experiment with the alphabody dimer in 10 X PBS buffer at pH 7 (top). Deconvoluted mass spectrum of the peak between t_R 4.780 min and 5.348 min (bottom). Starting alphabody dimer (22937 Da) is observed with an oxidation product (22953 Da) and double oxidation product (22967 Da). Additionally, an alphabody dimer TAD propanol conjugate is observed (23094 Da) with its single (23111 Da) and double (23126 Da) oxidation products are observed. Lastly also an alphabody dimer with double TAD-modification is observed (23250 Da) and its corresponding oxidation product (23266 Da).

3.3 Alphabody 586D

Another model protein used in this study is the alphabody 586D, like the valentine alphabody this alphabody has a triple helix coiled coil structure and it was obtained from Complix Therapeutics. 586D contains one tryptophan and three tyrosine residues in the primary amino acid sequence, all of which are solvent accessible (as estimated from 3D models). The exact sequence of 586D cannot be disclosed for confidentiality reasons. The experiments with alphabody 586D were analyzed on a LTQ-orbitrap XL (Thermo fisher scientific) by intact protein analysis. 20 µL of the protein conjugate sample (2 µg protein) was desalted on a 7.5 cm column (made in-house, 1 mm internal diameter (I.D.), 3 µm beads, C4 Reprosil, Dr. Maisch, Germany). Proteins were eluted from the column by a linear 5 min gradient ranging from 0.1% FA, 0.05% TFA in water to 70% acetonitrile. The mass spectrometer was operated in MS mode at a resolution of 30000. MS full-scans were acquired from 600 to 4000 M/Z.

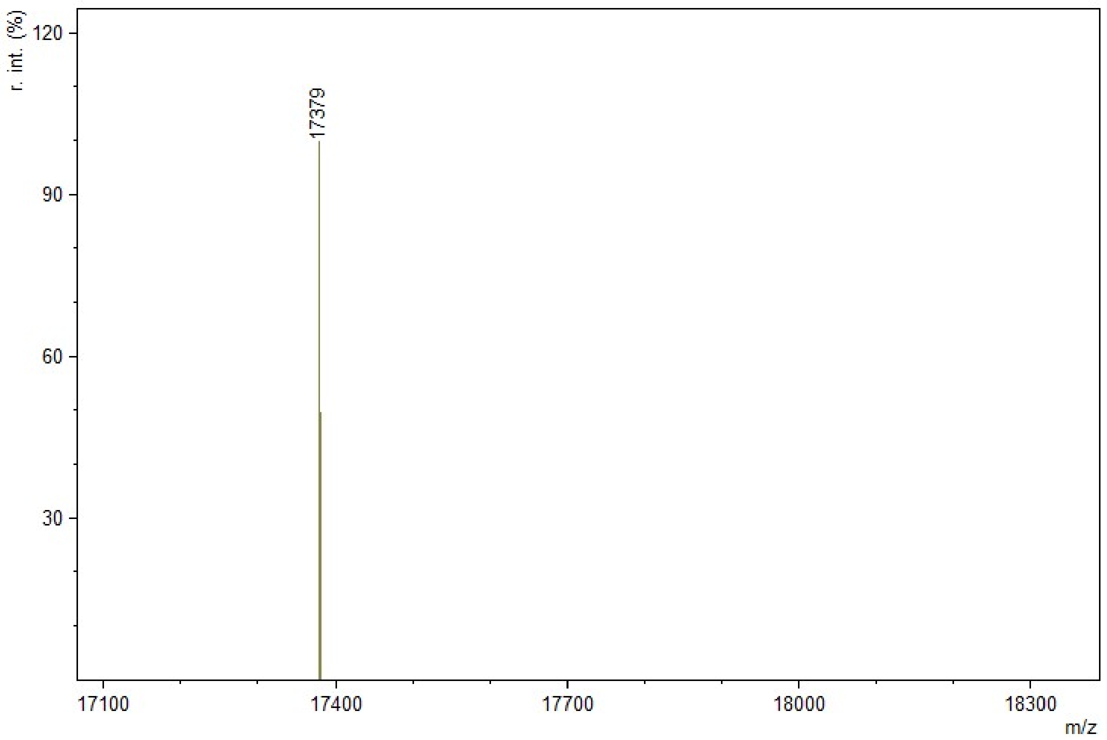

S3.3.1 Deconvoluted mass spectrum of the analysis of the alphabody 586D starting material. The detected molecular weight is 17379 Da.

Alphabody 586D conjugation with DMEQ-TAD in 10 X PBS pH 4 (20 eq.)

2 µL of the alphabody 586D stock solution (1.16 mg/mL) in 20 mM HEPES 150 mM NaCl was added to 16 µL 10 X PBS pH 4 and mixed well by pipetting up and down. 4 µL (20 eq) of a 0.7 mM DMEQ-TAD **2d** solution in MeCN was added and mixed well by pipetting up and down for several seconds.

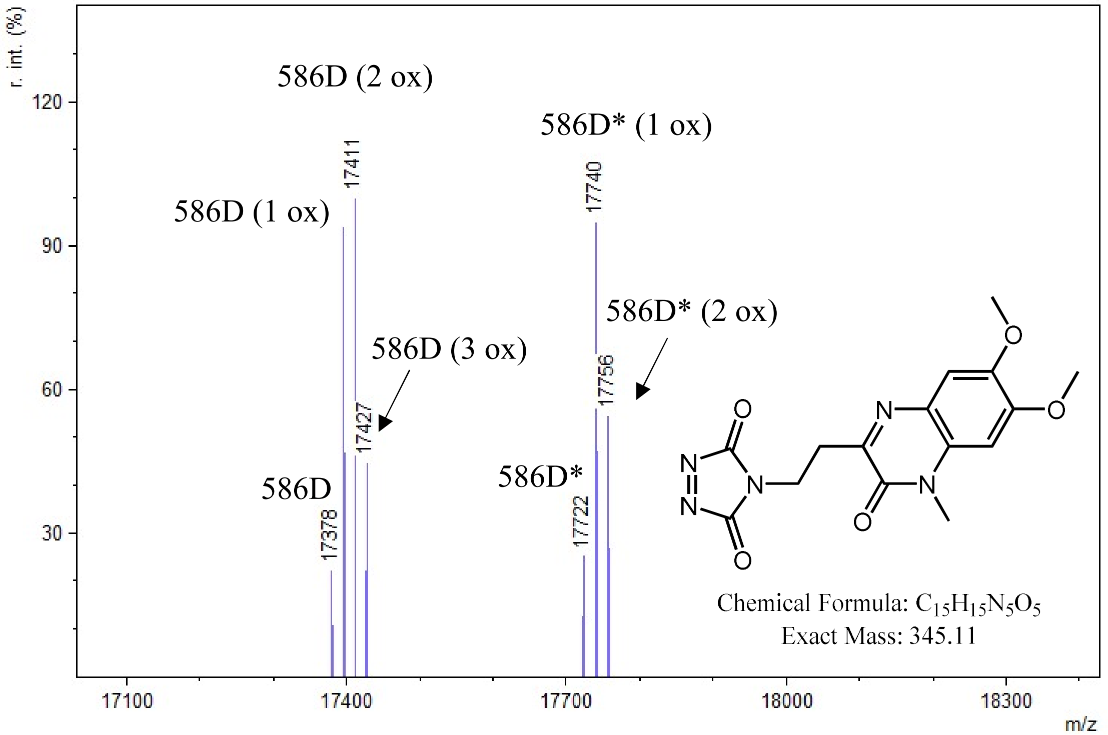

Figure S3.3.2 deconvoluted mass spectrum of the alphabody 586D protein after addition of DMEQ-TAD **2d** (20 eq) in 10 X PBS pH 4. We observe the starting 586D 17378 Da, with 1 oxidation (+16 Da) 17395 Da, with 2 (+32 Da) 17411 Da and with 3 oxidations (+48 Da) 17427 Da. Additionally we observe the DMEQ-TAD modified alphabody 586D also with 0, 1 and 2 oxidations corresponding to 17722 Da, 17740 Da and 17756 Da respectively.

Methionine oxidation

Upon TAD conjugation reaction we observe (M+16) oxidation products. This can be ascribed to the presence of methionine as the alphabody 586D recombinant protein contains 6 methionine residues in its primary sequence. We found that purging the buffer solution with argon prior to the conjugation reaction leads to reduced oxidation. Using this method in combination with DMEQ-TAD modification on alphabody 586D (6 methionine residues) we observed only one oxidation.

Alphabody 586D conjugation with DMEQ-TAD in 10 X PBS pH 4 (30 eq.)

To reduce methionine oxidation we first saturated the 10 X PBS pH 4 buffer. An open Eppendorf tube containing 10 X PBS pH 4 buffer solution was placed in a vial which was tightly closed with a septum. The vial was purged with argon for 2 hours. Afterwards the buffer was used immediately for protein conjugations. 2 µL of the alphabody 586D stock solution (1.16 mg/mL) in 20 mM HEPES 150 mM NaCl was added to 16 µL 10 X PBS pH 4 and mixed well by pipetting up and down. 4 µL (20 eq) of a 0.7 mM DMEQ-TAD **2d** solution in MeCN was added and mixed well by pipetting up and down for several seconds.

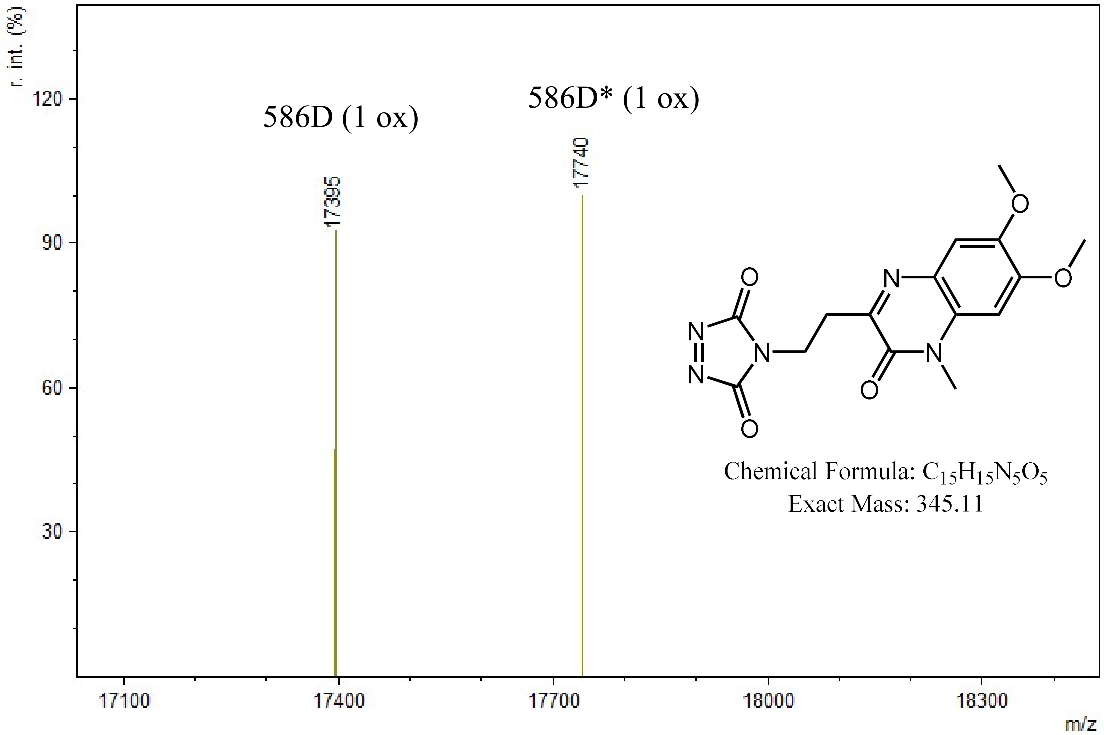

Figure S3.3.3 deconvoluted mass spectrum of the alphabody 586D protein after addition of DMEQ-TAD **2d** (30 eq) in 10 X PBS pH 4. Note that the buffer solution was saturated with argon prior to use. We observe the starting galectin protein with 1 oxidation (+16 Da) 17395 Da. Additionally we observe the DMEQ-TAD modified alphabody 586D also with 1 oxidation 17740 Da.

Trypsin digest of unmodified alphabody 586D and alphabody 586D conjugation with DMEQ-TAD propanol (20 eq) in 10 X PBS pH 4

2 µL of the alphabody 586D stock solution (1.16 mg/mL) in H_2O was added to 16 µL 10 X PBS pH 4 and mixed well by pipetting up and down. 4 µL of a 0.7 mM DMEQ-TAD solution in MeCN was added and mixed well by pipetting up and down for several seconds. For the unmodified sample, 2µL of the alphabody 586D stock was added to 18 µL 10 X PBS pH 4. 20 µL of 0.1 M Tris buffer pH 8 was added to both samples and the pH was further adjusted to 8 with 2 µL of a 0.57 M NaOH solution. 6 µL of a 0.16 mg/mL trypsin in H_2O was added and the mixture was shaken overnight in the dark. The digests were analyzed on a Fusion Lumos (Thermo) mass spectrometer using the same LC settings as for the synthetic peptides (see section 1.1). The mass spectrometer was operated in TopSpeed with a cycle time of 3s. MS1 scans were acquired with an AGC of 2E5 with a maximum ion time of 250 ms and a scan range of 300-1500 at a resolution of 120k. Precursor ions were selected for fragmentation through data dependent settings set to MIPS peptide mode, a charge state between 2-7, an intensity threshold of 5E3 and a dynamic exclusion of 60 s. Precursors were isolated with an isolation window of 1.2 Da in the ion routing multipole, fragmented in the HCD cell with a CE of 34% after obtaining an AGC of 12E3 with a maximum fill time of 40 ms. The fragment ions were detected though the ion trap analyzer in normal mode. In the Mascot search DMEQ-TAD modification of tyrosine and tryptophan was included as a variable modification. For the modification on tryptophan a neutral loss of 345.1073Da was taken into account for modified fragments in the HCD MS/MS spectra, since we have observed that tryptophan-TAD modifications are labile in HCD MS/MS analyses. Tolerance on MS1 level was set to 10 ppm, whereas the tolerance on MS2 level was set to 0.5 Da. The peak lists were generated through Mascot Distiller and searched against the SwissProt proteome v06_2020 restricted to human (containing 20433 sequences) concatenated with the alphabody 586D sequence.

As the sequence of alphabody 586D is confidential, we here show the analysis data for the tryptic peptide containing the tryptophan which also contains a tyrosine residue XXXXXXXYXXWXXX. This tryptic peptide was detected in the unmodified protein sample and was fragmented and annotated using HCD MS/MS and scored 93 (figure 3.3.4). In the sample of the alphabody 586D modified with 20 equivalents of DMEQ-TAD at pH 4, the DMEQ-TAD modified tryptic peptide XXXXXXXYXXW*XXX was detected and annotated via HCD MS/MS analysis and scored 37 (figure 3.3.5). The HCD analysis demonstrates that the DMEQ-TAD modification is located on the tryptophan residue. Besides the tyrosine residue in the same tryptic sequence as tryptophan, there are 2 more tyrosine residues. The tryptic peptides containing these tyrosines were detected as unmodified peptides.

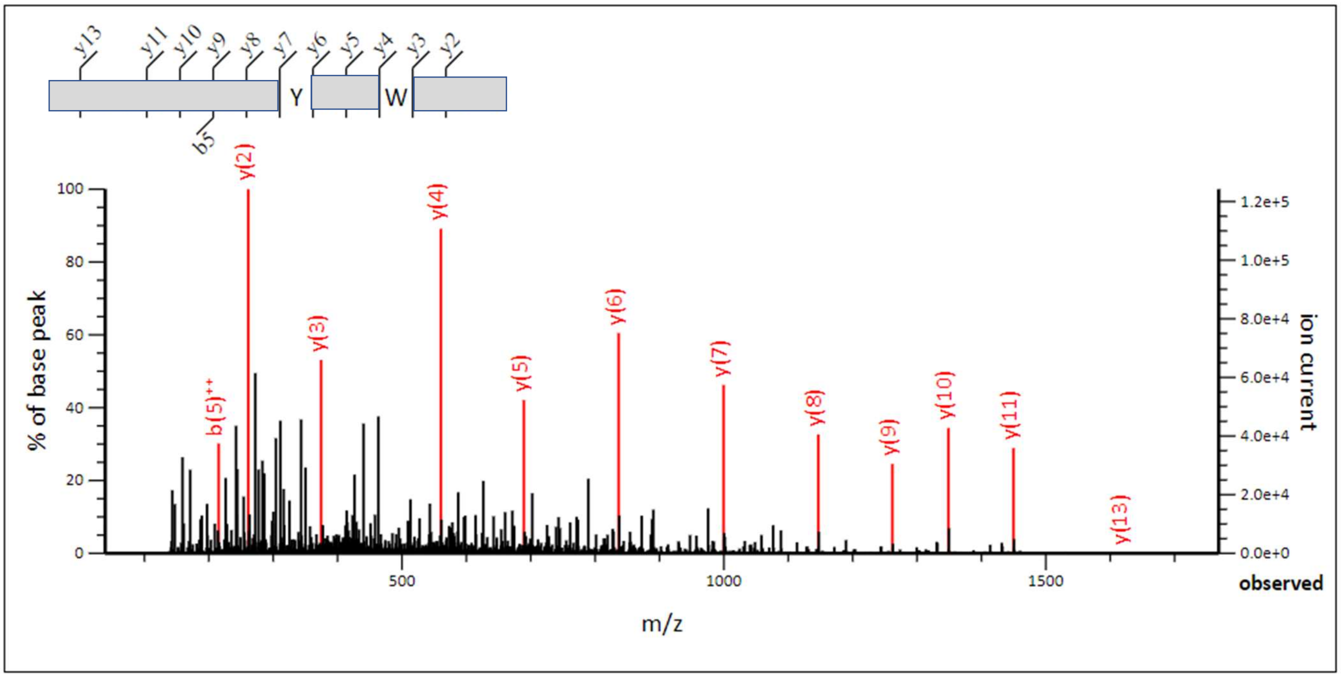

Figure 3.3.4 HCD MS/MS spectrum of the unmodified tryptic peptide (XXXXXXXYXXWXXX) (top).

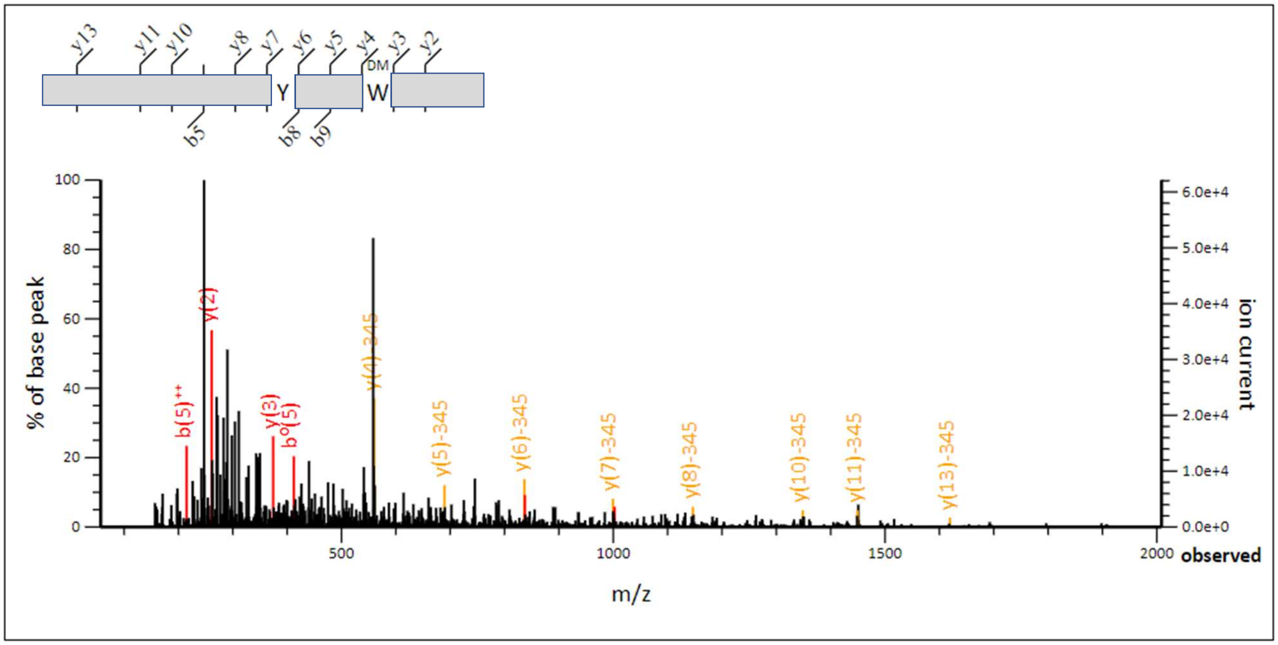

Figure 3.3.5 HCD MS/MS spectrum of the DMEQ-TAD modified tryptic peptide (XXXXXXXYXXW*XXX). The modification reaction was performed in 10 X PBS pH 4. The "DM" label above the tryptophan residue indicates the location of the DMEQ-TAD. Note that in this figure a fragment ion is marked with "^{0}" this indicates -H_2O. The annotated peaks in yellow contain -345 in their label, this indicates the loss of DMEQ-TAD modification on tryptophan as is expected in HCD MS/MS.

Alphabody 586D DMEQ-TAD conjugate stability

The peptide conjugate stability was tested on peptide **1f** and TAD-propanol, **2b**. To confirm these data on the protein level the stability of conjugate alphabody 586D DMEQ-TAD, **2d** was tested via HPLC analysis at 370 nm (DMEQ absorbance). Alphabody conjugation was performed with 20 equivalents DMEQ-TAD in 10 X PBS pH 4 as described in 3.3. 100 µL of sample was diluted with 100 µL of 10 X PBS pH 9, and the pH was adjusted to a value of 7. The sample was left on the lab bench at room temperature and analysed by HPLC right after the dilution and 24 hours later. Additionally, also 2 blanc samples were recorded: a blanc alphabody 586D sample to identify the alphabody retention time. The alphabody elutes around 4 minutes (figure 3.3.6, A: 214 nm), no absorption is detected at 370 nm for alphabody 586D (figure 3.3.6, B: 370 nm).

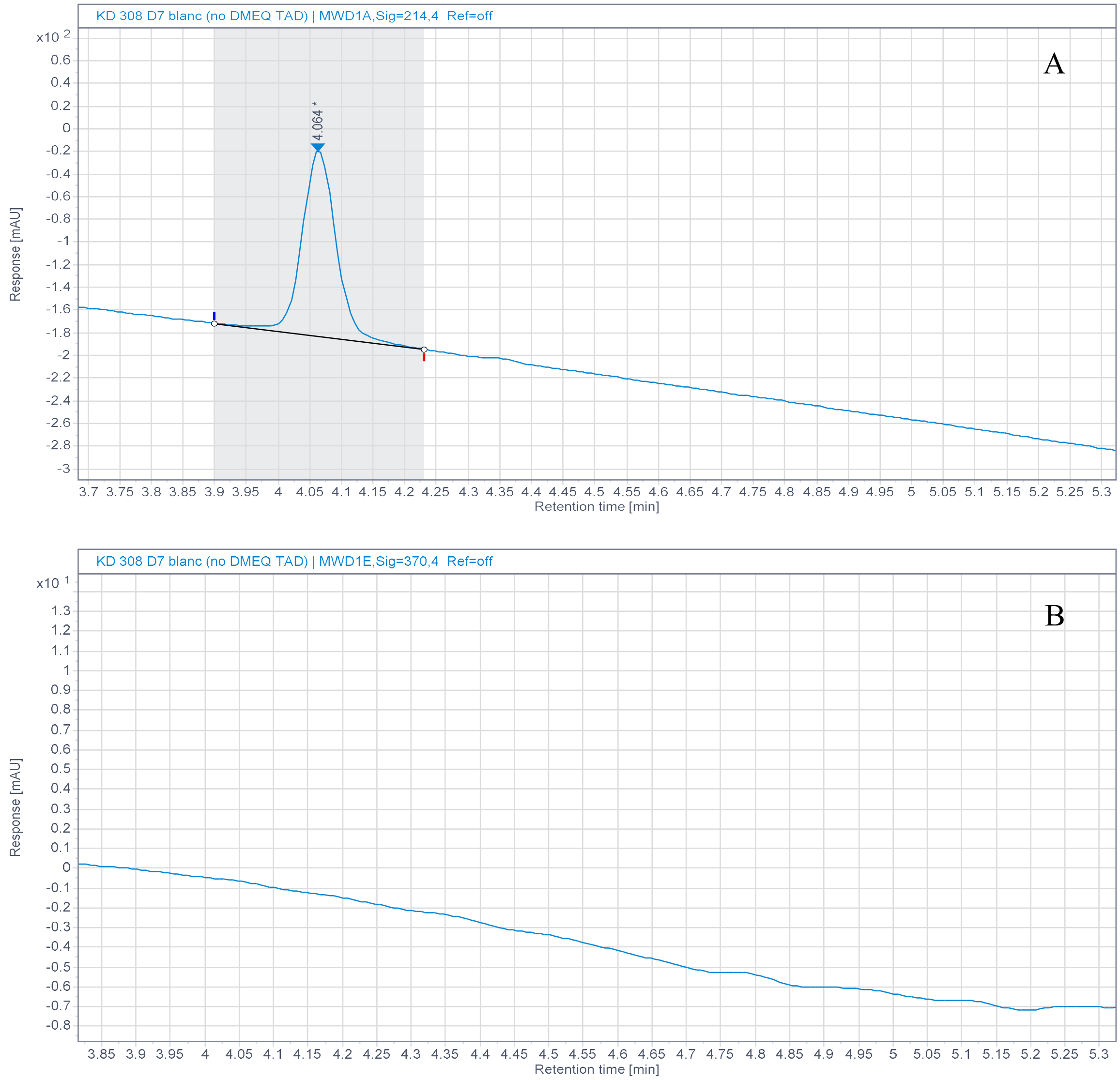

Figure 3.3.6 Zoom of the alphabody region of a HPLC chromatogram (A: 214 nm, B: 370 nm). Blanc sample: alphabody 586D in 10 X PBS pH 4, no DMEQ-TAD present.

Another blanc consists of an experiment with DMEQ-TAD, **2d** in 10 X PBS pH 4 but without alphabody present, to confirm that there is no absorption at 370 nm in the alphabody region in the blanc sample.

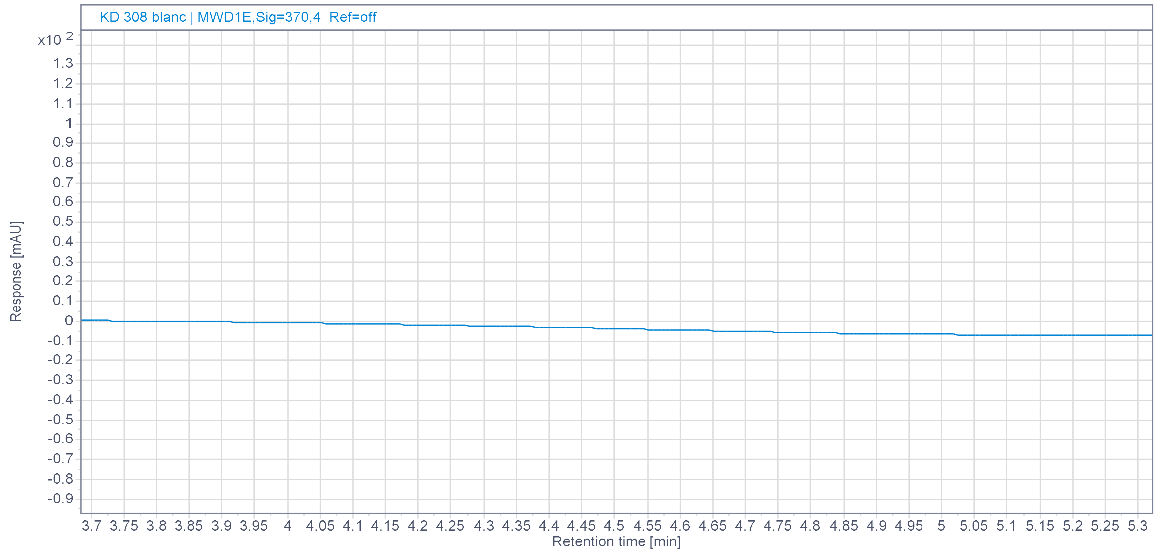

Figure 3.3.7 Zoom of the alphabody region of a HPLC chromatogram (370 nm). Blanc sample: DMEQ-TAD in 10 X PBS pH 4, no alphabody 586D present.

In the stability experiment the alphabody 586D DMEQ-TAD, **2d** conjugate was analysed by HPLC at the start of the experiment (after dilution into 10 X PBS pH 7) and 24 hours later. The peak area of the conjugate peak in both 370 nm chromatograms remained roughly the same over 24 hours at room temperature (figure 3.3.8). These findings confirm the stability of the tryptophan-TAD conjugation on proteins in 10 X PBS pH 7 at room temperature. Longer stability time measurements (up to several weeks) as we did for the peptide conjugate **2fb** were not possible for the alphabody 586D conjugate due to instability of the protein itself.

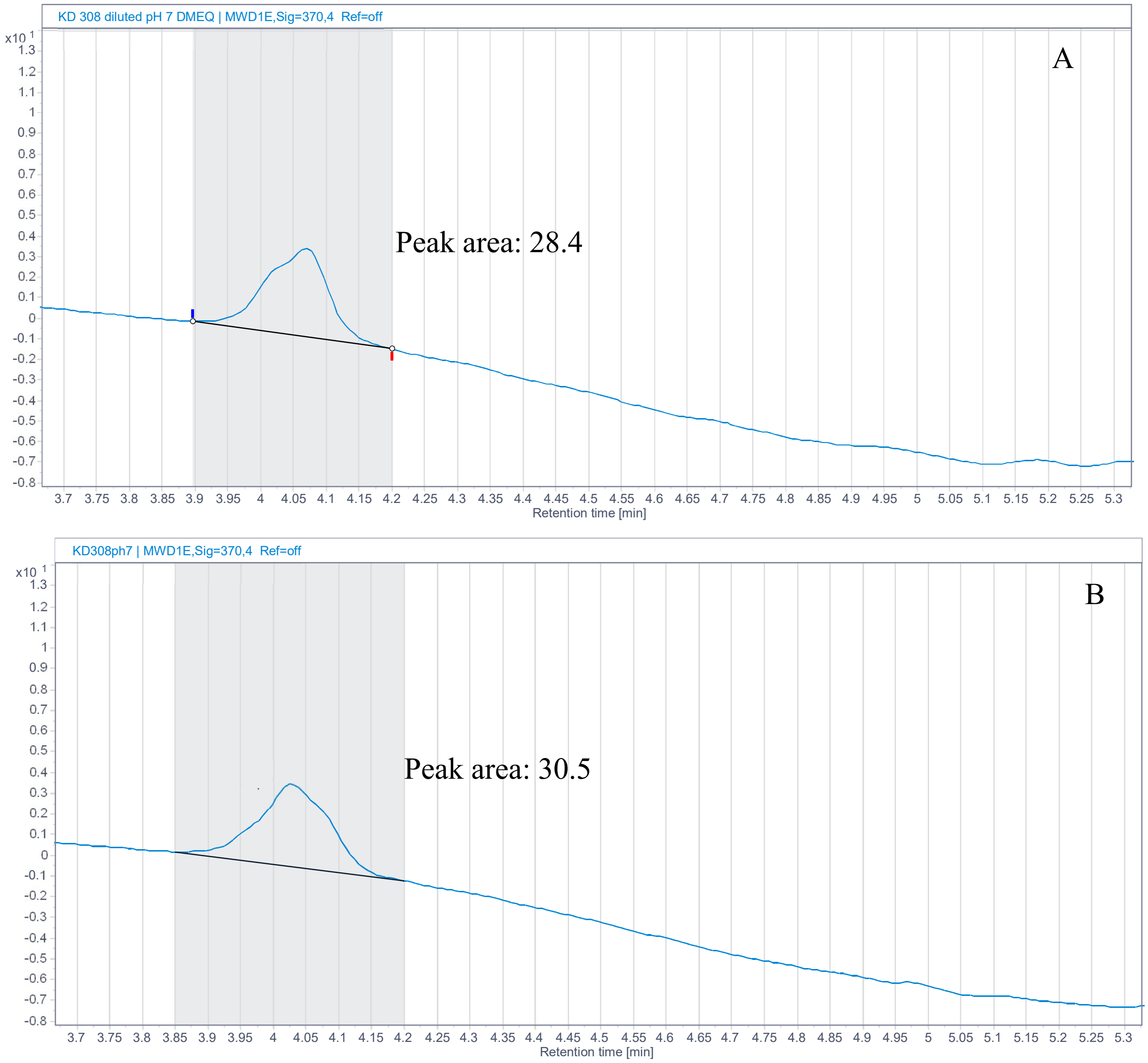

Figure 3.3.8 Zoom of the alphabody region of a HPLC chromatogram (370 nm). Alphabody 586D conjugated with DMEQ-TAD, **2d**. Conjugation performed in 10 X PBS pH 4. stability experiment in 10 X PBS pH 7 at room temperature. A: 370 nm chromatogram from HPLC analysis right after conjugation and dilution into PBS pH 7; B: 370 nm chromatogram from HPLC analysis after 24 hours.

3.4 Human galectin-7

A model protein used in this study is galectin-7,[9] a human protein with a molecular weight of 15.4 kDa, purchased from Merck (recombinantly expressed in *E. coli*). In the primary sequence of this protein one tyrosine and one tryptophan residue are present. Additionally there is a methionine residue present in the reported primary amino acid sequence of galectin-7[1]. In the recombinant galectin-7 protein obtained from Merck there are three unknown amino acids added[2]. The experiments with galectin-7 were analyzed on an LTQ-orbitrap XL (Thermo Fisher Scientific) through intact protein analysis in the same manner as the alphabody 586D as described above (section 3.3).

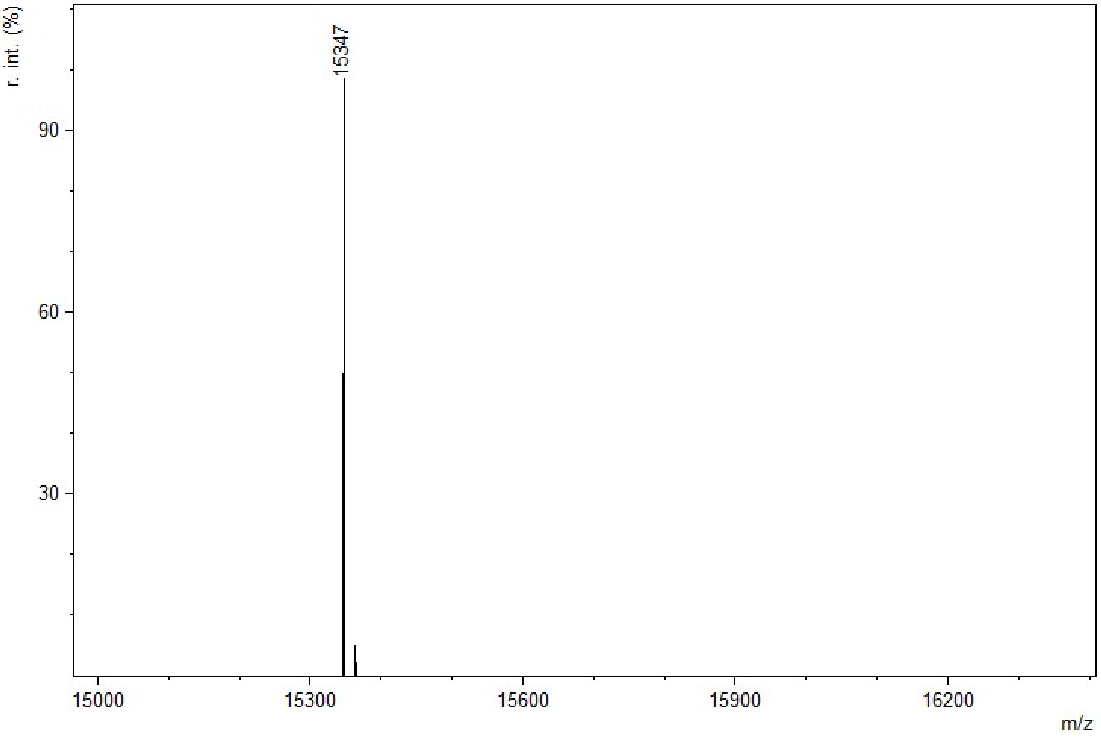

Figure

Figure S3.4.1 Deconvoluted mass spectrum of the analysis of the galectin-7 starting material. The detected molecular weight for the galectin-7 protein is 15347 Da.

[1] https://www.uniprot.org/uniprot/P47929

[2] Merck was not able to give further information on the three added amino acids which are present after HIS-tag removal. Most likely at least one extra methionine is present.

Galectin-7 conjugation with DMEQ-TAD in 10 X PBS pH 4 (10 eq.)

2 µL of the galectin-7 stock solution (1mg/mL) in H_2O was added to 16 µL 10 X PBS pH 4 and mixed well by pipetting up and down. 2 µL (10 eq) of a 0.7 mM DMEQ-TAD **2d** solution in MeCN was added and mixed well by pipetting up and down for several seconds.

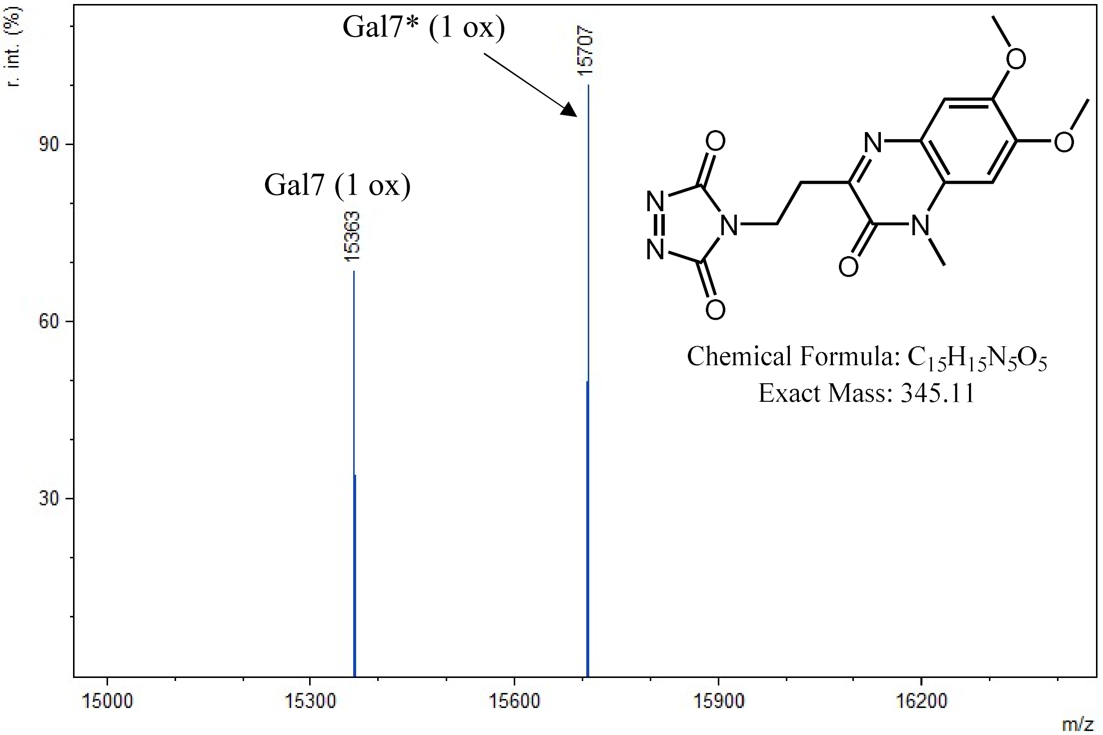

Figure S3.4.2 deconvoluted mass spectrum of the galectin-7 protein after addition of DMEQ-TAD **2d** (10 eq) in 10 X PBS pH 4. We observe the starting galectin protein with an oxidation (+16 Da, *vide supra* methionine presence) at 15363 Da. Additionally we observe the DMEQ-TAD modified galectin-7 with an oxidation at 15707 Da.

Galectin-7 conjugation with TAD-propanol in 10 X PBS pH 4 (10 eq.)

2 µL of the galectin-7 stock solution (1mg/mL) in H_2O was added to 16 µL 10 X PBS pH 4 and mixed well by pipetting up and down. 2 µL (10 eq) of a 0.7 mM TAD-propanol **2b** solution in MeCN was added and mixed well by pipetting up and down for several seconds.

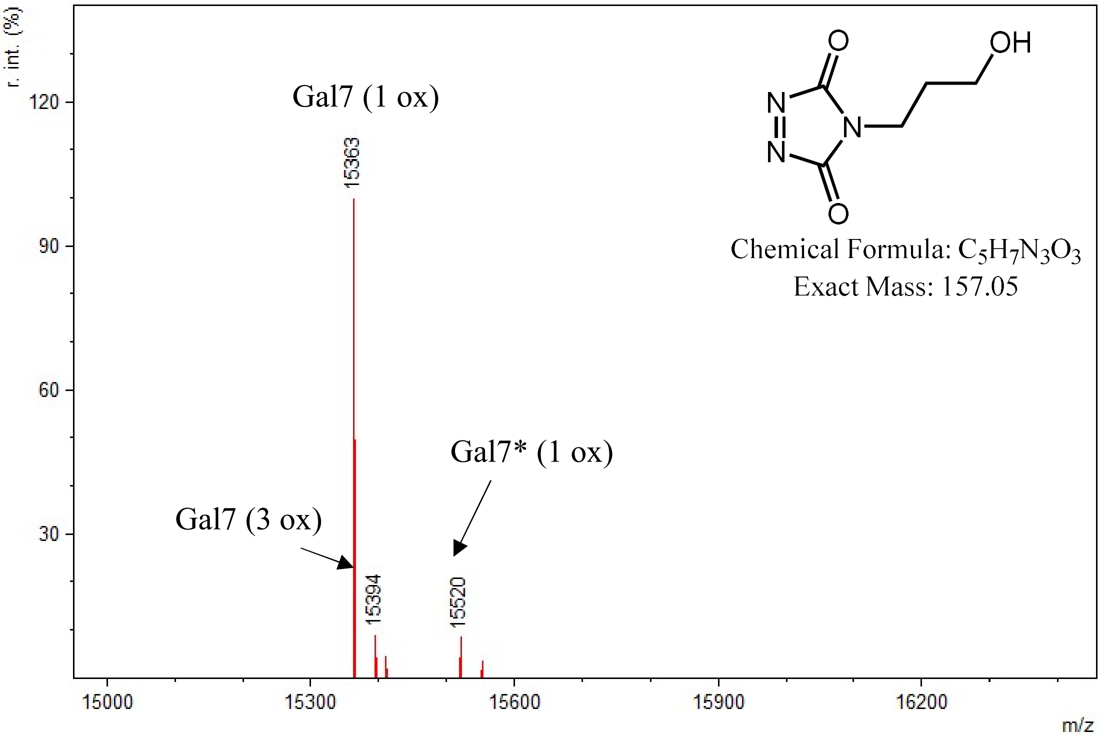

Figure S3.4.3 deconvoluted mass spectrum of the galectin-7 protein after addition of TAD-propanol **2b** (10 eq) in 10 X PBS pH 4. We observe the starting galectin protein with an oxidation (+16 Da, *vide supra* methionine presence) at 15363 Da and a small amount of galectin-7 with three oxidations (+48 Da, *vide supra* methionine presence) at 15394 Da. Additionally we observe the TAD-propanol modified galectin-7 also with an oxidation at 15520 Da.

Galectin-7 conjugation with PTAD-alkyne in 10 X PBS pH 4 (10 eq.)

2 µL of the galectin-7 stock solution (1mg/mL) in H_2O was added to 16 µL 10 X PBS pH 4 and mixed well by pipetting up and down. 2 µL (10 eq) of a 0.7 mM PTAD- alkyne **2c** solution in MeCN was added and mixed well by pipetting up and down for several seconds.

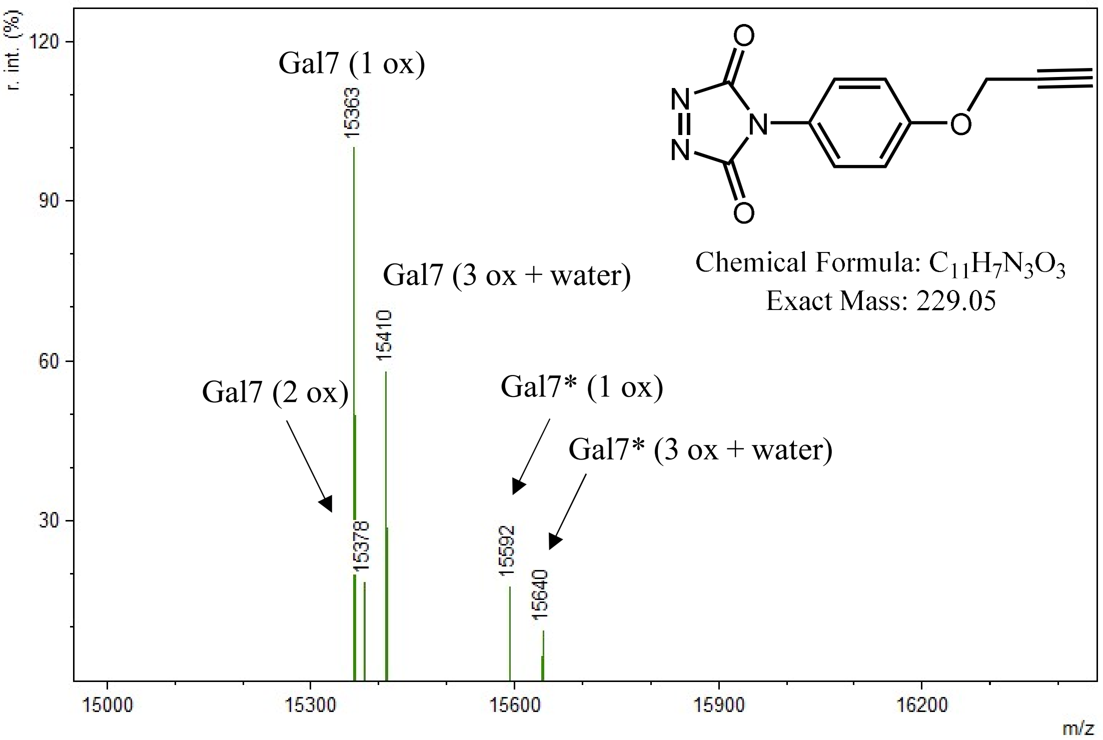

Figure S3.4.4 deconvoluted mass spectrum of the galectin-7 protein after addition of PTAD-alkyne **2c** (10 eq) in 10 X PBS pH 4. We observe the starting galectin protein with an oxidation (+16 Da, *vide supra* methionine presence) at 15363 Da, a double (+32 Da) at 15378 Da and triple oxidation + water (+66 Da) at 15410 Da. Additionally we observe the PTAD-alkyne modified galectin-7 with an oxidation at 15520 Da and with a triple oxidation + water at 15640 Da.

Trypsin digest of unmodified galectin-7 and galectin-7 conjugation with TAD propanol (20 eq) in 10 X PBS pH 4 and pH 7

2 µL of the galectin-7 stock solution (1mg/mL) in H_2O was added to 16 µL 10 X PBS pH 4 or 7 and mixed well by pipetting up and down. 2 µL of a 1.4 mM TAD-propanol solution in MeCN was added and mixed well by pipetting up and down for several seconds. For the unmodified galectin-7 sample 2 µL of protein stock solution was added to 18 µL of 10 X PBS buffer pH 4. 20 µL of 0.1 M Tris buffer pH 8 was added, for the sample in 10 X PBS pH 4 the pH was further adjusted to 8 with 2 µL of a 0.57 M NaOH solution. 6 µL of a 0.16 mg/mL trypsin in H_2O was added and the mixture was shaken overnight in the dark. The digests were analyzed on a Fusion Lumos (Thermo) mass spectrometer using the same LC settings as the synthetic peptides (see section 1.1). The mass spectrometer was operated in TopSpeed with a cycle time of 3s. MS1 scans were acquired with an AGC of 2E5 with a maximum ion time of 250 ms and a scan range of 300-1500 at a resolution of 120k. Precursor ions were selected for fragmentation through data dependent settings set to MIPS peptide mode, a charge state between 2-7, an intensity threshold of 5E3 and a dynamic exclusion of 60 s. Precursors were isolated with an isolation window of 1.2 Da in the ion routing multipole, fragmented in the HCD cell with a CE of 34% after obtaining an AGC of 12E3 with a maximum fill time of 40 ms. The fragment ions were detected though the ion trap analyzer in normal mode. To focus on the tyrosine and tryptophan peptide, a targeted inclusion method was used using the double and triple charged precursor ions of the TAD modified tyrosine and tryptophan peptide in the inclusion list. The dynamic exclusion option was left out for these targeted methods.

The TAD-propanol modified tryptic peptide containing tyrosine (AVVGDAQY*HHFR) was only detected in trace amounts in the galectin-7 TAD-propanol conjugation at pH 4. Peak area values for the double and triple charged TAD-modified tryptic peptide were normalized to peptides K.SSLPEGIRPGTVLR.I [7, 20] (missed 1) and R.HRLPLAR.V [111, 117] (missed 1) skyline software (figure S3.4.5). On the other hand, the TAD-propanol modified tryptic peptide containing tryptophan was detected in both samples. Peak area values for the double and triple charged TAD-modified tryptic peptide were normalized to peptides K.SSLPEGIRPGTVLR.I [7, 20] (missed 1) and R.HRLPLAR.V [111, 117] (missed 1) skyline software (figure S3.4.6). In the Mascot search TAD-propanol modification of tyrosine and tryptophan was included as a variable modification. For the modification on tryptophan a neutral loss of 157.0487 Da was taken into account for modified fragments in the HCD MS/MS spectra, since we have observed that tryptophan-TAD modifications are labile in HCD MS/MS analyses (figures S3.4.7 and S3.4.9). Tolerance on MS1 level was set to 10 ppm, whereas the tolerance on MS2 level was set to 0.5 Da. The peak lists were generated through Mascot Distiller and searched against the SwissProt proteome v06_2020 restricted to human (containing 20433 sequences) concatenated with the Galectin-7 sequence. In the unmodified galectin-7 experiment, the tryptophan containing peptide was not detected.

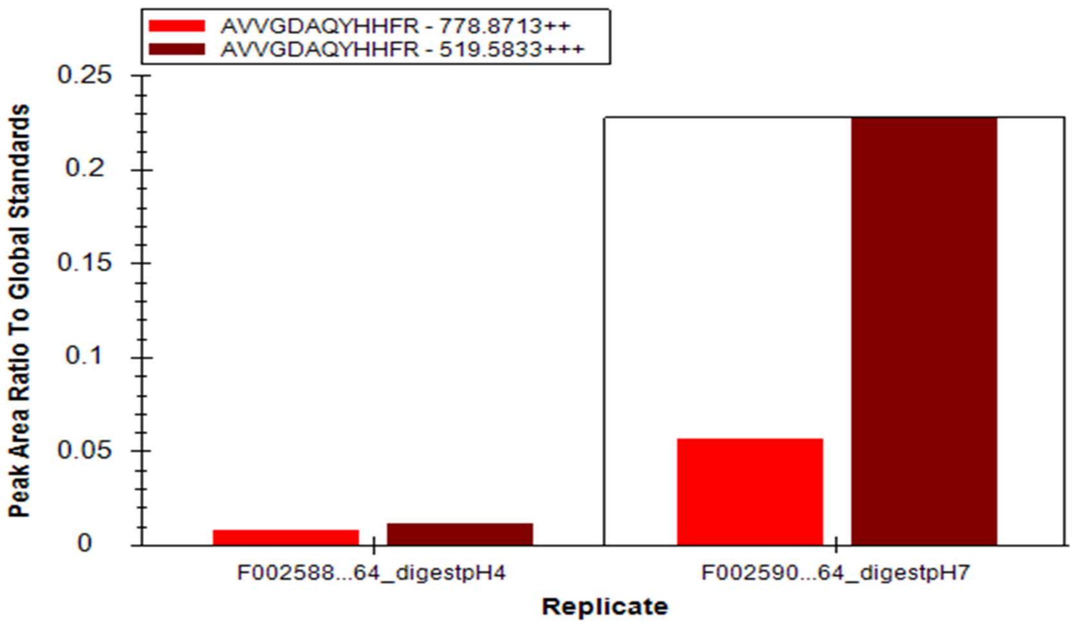

Figure S 3.4.5 Histogram representing the peak area of the TAD-propanol modified tryptic peptide containing tyrosine (AVVGDAQY*HHFR) in the samples at pH 4 left and pH 7 right.

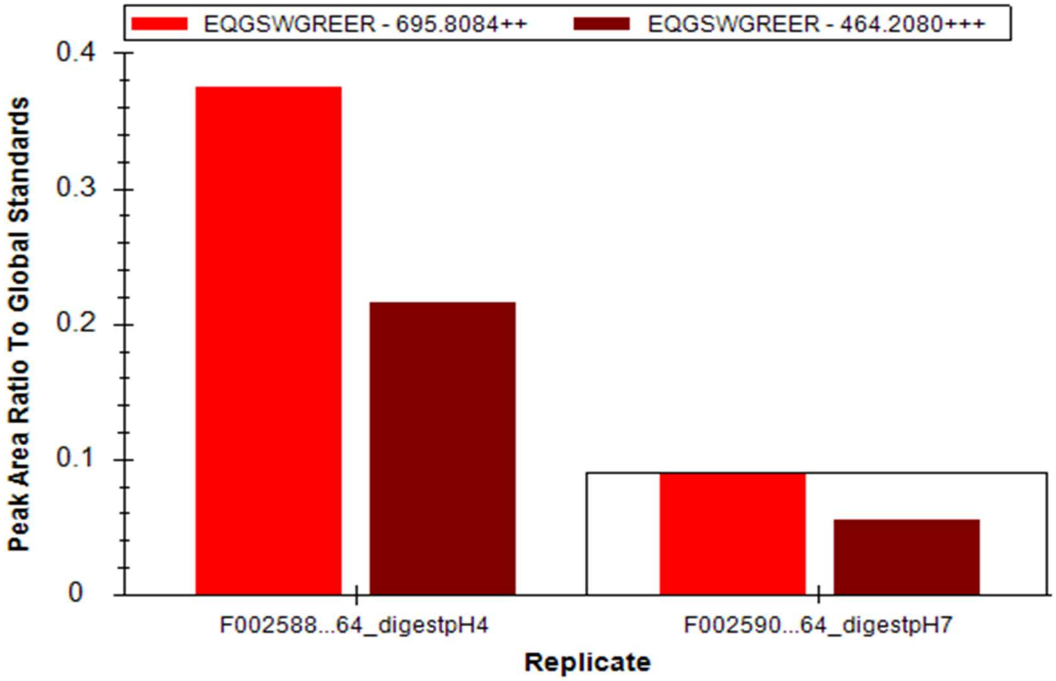

Figure S 3.4.6 Histogram representing the peak area of the TAD-propanol modified tryptic peptide containing tryptophan (EQGSW*GREER) in the samples at pH 4 left and pH 7 right.

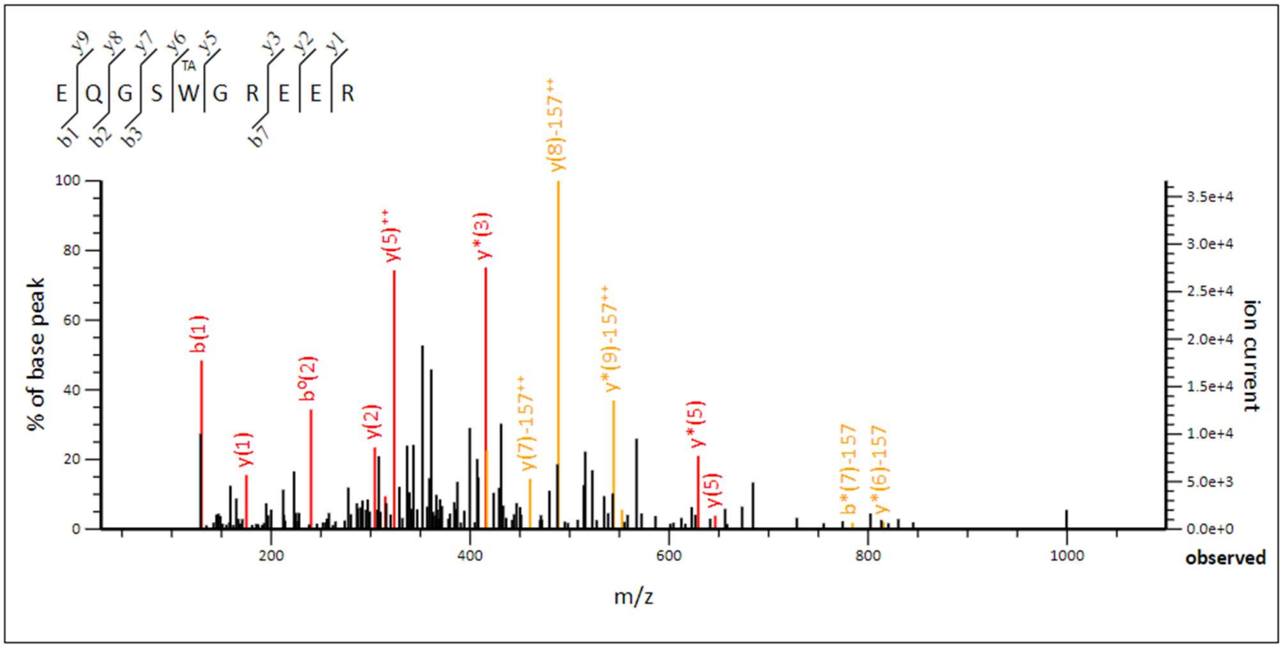

Figure S3.4.7 HCD MS/MS spectrum of the TAD-propanol modified tryptic peptide (EQGSW*GREER), MASCOT score 8. The modification reaction was performed in 10 X PBS pH 4. Note that in this figure some fragment ions are marked with "*" or "^{0}" this indicates $-NH_3$ or $-H_2O$ respectively. The "*" modification used here is not to be confused with TAD-modified peptides/proteins in the remainder of this document also indicated by "*". The annotated peaks in yellow contain -157 in their label, this indicates the loss of TAD -propanol modification on tryptophan as is expected in HCD MS/MS.

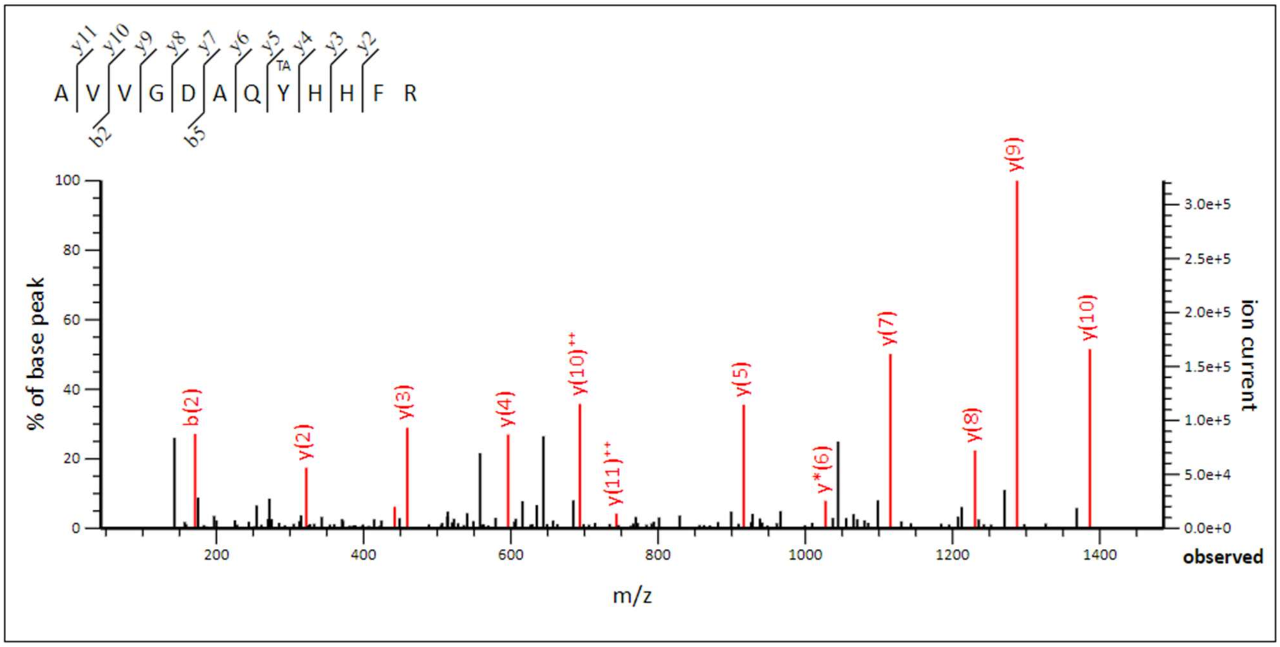

Figure S3.4.8 HCD MS/MS spectrum of the TAD-propanol modified tryptic peptide (AVVGDAQY*HHFR), MASCOT score 77. The modification reaction was performed in 10 X PBS pH 7. Note that in this figure some fragment ions are marked with "*" this indicates $-NH_3$. The "*" modification used here is not to be confused with TAD-modified peptides/proteins in the remainder of this document also indicated by "*".

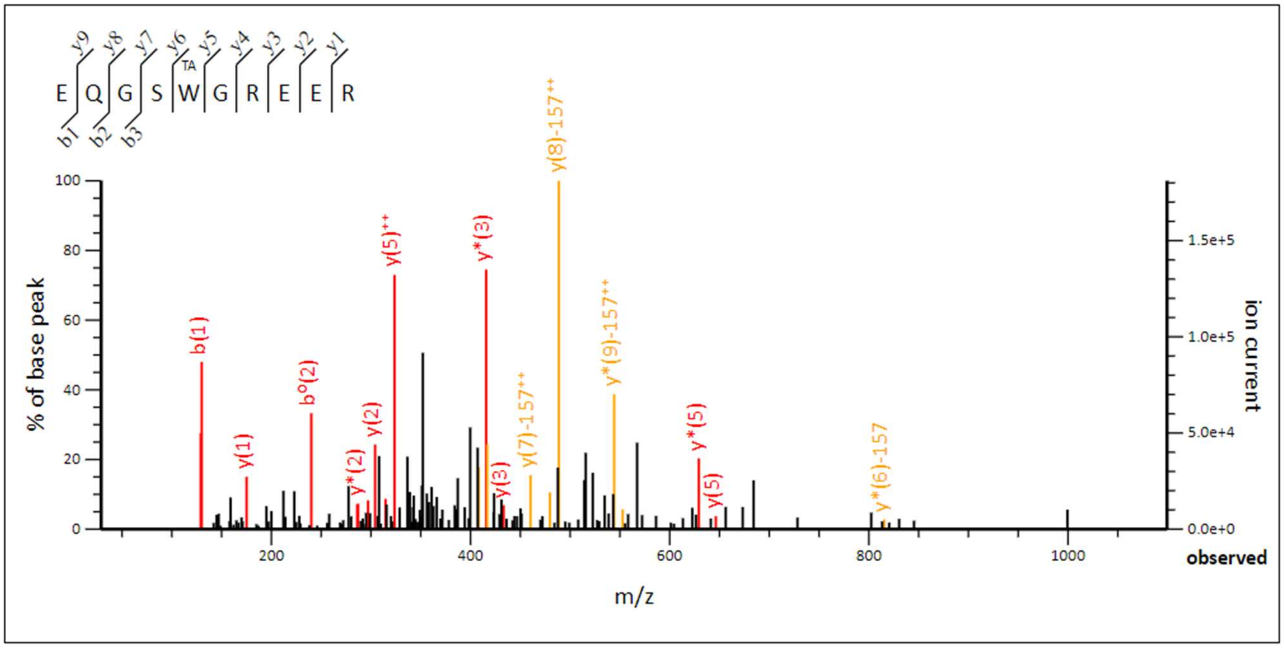

Figure S3.4.9 HCD MS/MS spectrum of the TAD-propanol modified tryptic peptide (EQGSW*GREER), MASCOT score 16. The TAD-propanol label is indicated by “TA” above the tryptophan residue. The modification reaction was performed in 10 X PBS pH 7. Note that in this figure some fragment ions are marked with “*” or “⁰” this indicates $-NH_3$ or $-H_2O$ respectively. The “*” modification used here is not to be confused with TAD-modified peptides/proteins in the remainder of this document also indicated by “*”. The annotated peaks in yellow contain -157 in their label, this indicates the loss of TAD -propanol modification on tryptophan as is expected in HCD MS/MS.

3.5 ApoE4 nanobody

A nanobody against apolipoprotein E4 was used as a model protein in this study. Apolipoprotein E4 is the most prevalent risk factor for sporadic Alzheimer's disease. The molecular weight of the apoE4 nanobody is about 15.6 kDa and this nanobody and the apoE4 antigen were a kind gift of prof. Jan Gettemans' lab (Ghent University). The nanobody contains two tryptophan residues and 11 tyrosine residues in its primary amino acid sequence.

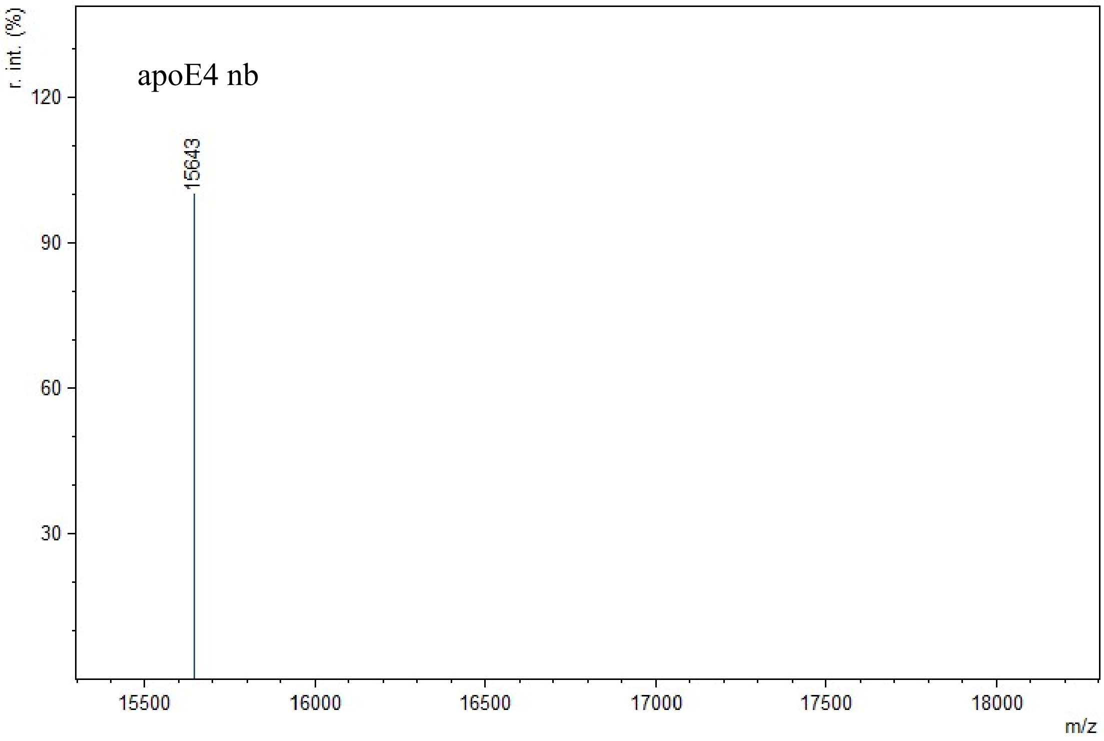

Figure S3.5.1 Deconvoluted mass spectrum of the intact mass analysis of the apoE4 nanobody starting material. The detected molecular weight for the apoE4 nanobody is 15643 Da.

ApoE4 nanobody conjugation with DMEQ-TAD in PBS at pH 4 (20 eq.)

2 µL (20 eq) of a 5 mM DMEQ-TAD **2d** solution in MeCN was added to 20 µL of nanobody solution (0.36 mg/mL) in PBS at pH 4 and mixed well by pipetting up and down for several seconds. Afterwards the sample was analysed via intact mass analysis. The deconvoluted mass spectrum demonstrated 86% conversion with (58% single modification and 28% double modification). Conversion rate estimated based on the peak intensities of the deconvoluted MS spectrum.

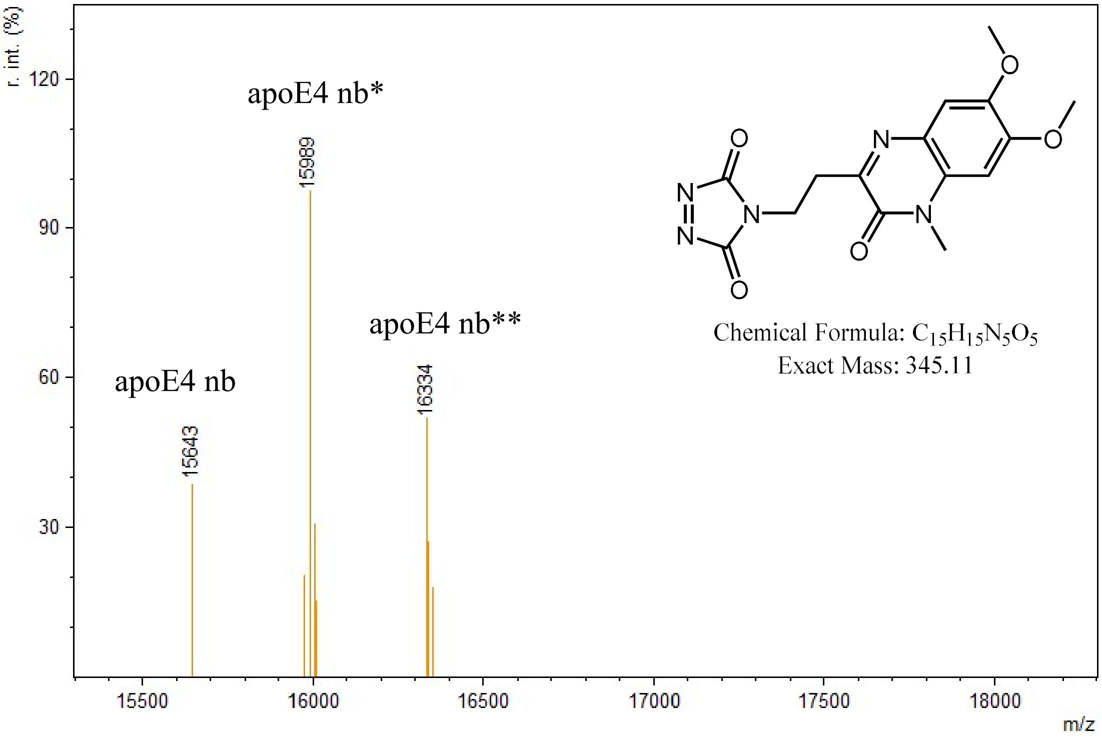

Figure S3.5.2 Deconvoluted mass spectrum of the apoE4 nanobody after addition of DMEQ-TAD **2d** (20 eq) in PBS at pH 4. We observed the starting nanobody at 15643 Da. Additionally, we observed the single DMEQ-TAD modified nanobody at 15989 Da and the double DMEQ-TAD modified nanobody at 16334 Da, both with a low intensity oxidation (+16 Da) product.

ApoE4 nanobody conjugation with DMEQ-TAD in PBS at pH 7 (20 eq.)

2 µL (20 eq) of a 5 mM DMEQ-TAD **2d** solution in MeCN was added to 20 µL of nanobody solution (0.36 mg/mL) in PBS at pH 7 and mixed well by pipetting up and down for several seconds. Afterwards the sample was analysed via intact mass analysis. The deconvoluted mass spectrum demonstrated a heterogeneous mixture containing nanobody species with 1 up to 6 modifications.

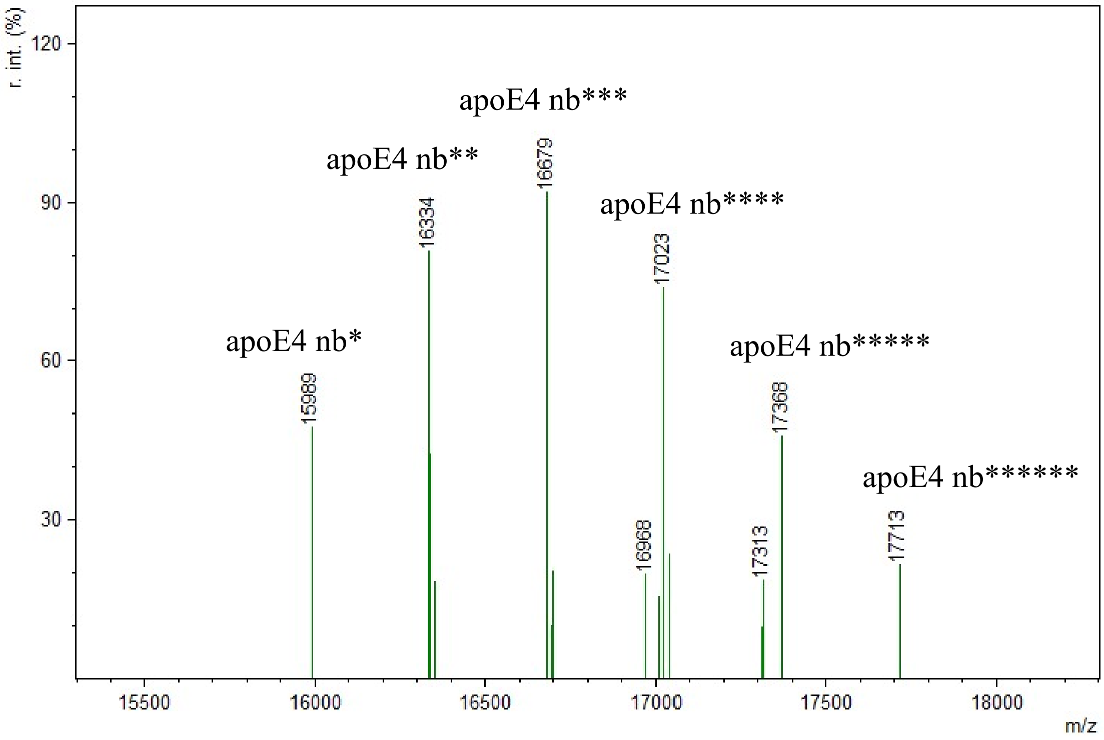

Figure S3.5.3 Deconvoluted mass spectrum of the apoE4 nanobody after addition of DMEQ-TAD **2d** (20 eq) in PBS pH 7. We observed a heterogeneous mixture of DMEQ-TAD modified nanobodies with one to six modifications (apoE4 nb*: 15989 Da; apoE4 nb**: 16334 Da; apoE4 nb***: 16679 Da; apoE4 nb****: 17023 Da; apoE4 nb*****: 17368 Da and apoE4 nb******: 17713 Da), some of these accompanied by a low intensity oxidation (+16 Da) or dehydration (-18 Da) product. Besides the oxidation and dehydration products also 2 other low intensity products (16968 Da and 17313 Da) were observed, these correspond to additional modification on lysine, where the TAD moiety first decomposes into an isocyanate which can subsequently modify a lysine residue with a mass addition of +289 Da.[10]

K_D determination after apoE4 DMEQ-TAD conjugation.

The same apoE4 nanobody conjugation samples, which were analysed via intact mass analysis (*vide supra*), were also used for the determination of their K_D values. Two experiments were performed, each comprising a labelled nanobody sample (20 µL nanobody solution in either PBS pH 4 or pH 7 labelled with 2 µL 5 mM DMEQ-TAD in MeCN) and a control nanobody sample (20 µL nanobody solution in PBS pH 7 with 2 µL MeCN). The affinity experiments were run on an Octet RED96 system (ForteBio) at 25 °C. Anti-Penta-HIS biosensors (HIS1K, ForteBio) were pre-wetted in kinetics buffer (KB, PBS (Gibco, Thermo Fisher) with 0.1% (w/v) BSA) for at least 10 min. Loading solutions, containing 10 µg/mL protein, were prepared by diluting the apoE4 nanobody conjugate sample and control nanobody sample in KB. All samples were dispensed into a 96-wellplate (Greiner Bio-One, 655209) at a volume of 200 µL. A single kinetics run entailed the following steps: Biosensor Regeneration (5 s in 0.5 M H_2SO_4 followed by 5 s in KB, for three cycles), Baseline (60 s in KB), Loading (300 s in loading solution), Baseline (60 s in KB), Association (300 s in KB containing the purified antigen apoE4) and Dissociation (600 s in KB). Flow rate was set to 1000 for all steps. For Association, three apoE4 concentrations were included (200 nM, 100 nM and 50 nM) and a single reference well without apoE4 for background subtraction. Data were processed (software version 9.0.0.4) using the reference well for subtraction, the baseline was aligned to the y-axis (last 5 s of the Baseline step) and Savitzky-Golay filtering was used to reduce noise. K_D values were generated by fitting (global fit using curves from all ApoE4 concentrations) the full association and dissociation steps to a 1:1 kinetic model.

Table S3.5.1 K_D data for the labelled (A and C) and control (not labelled, B and D) apoE4 samples.

Sample	Cycle	K_D(M)	K_D error	k_{on} (1/Ms)	k_{dis} (1/s)	Full X^2	Full R^2
apoE4 DMEQ-TAD pH4 (A)	1	8.74E-09	8.64E-11	1.10E+05	9.59E-04	0.4881	0.9875
apoE4 control (B)	1	4.81E-09	3.37E-11	7.37E+04	3.54E-04	0.5496	0.9971
apoE4 DMEQ-TAD pH7 (C)	2	1.02E-08	9.60E-11	9.82E+04	9.98E-04	0.1593	0.9911
apoE4 control (D)	2	5.33E-09	4.03E-11	6.52E+04	3.48E-04	0.2778	0.9979

These data show that after the labelling reaction with 20 eq. DMEQ-TAD at pH 4 (86% conversion, *vide supra*) the apoE4 nanobody still has a K_D value of 8.74 nM, whilst the control sample has a K_D of 4.81 nM for the apoE4 protein. Moreover, the apoE4 nanobody labeled with the same amount of DMEQ-TAD at pH 7 (conjugate mixture containing nanobody species with 1 up to 6 modifications) resulted in a slightly higher K_D value of 10.2 nM.

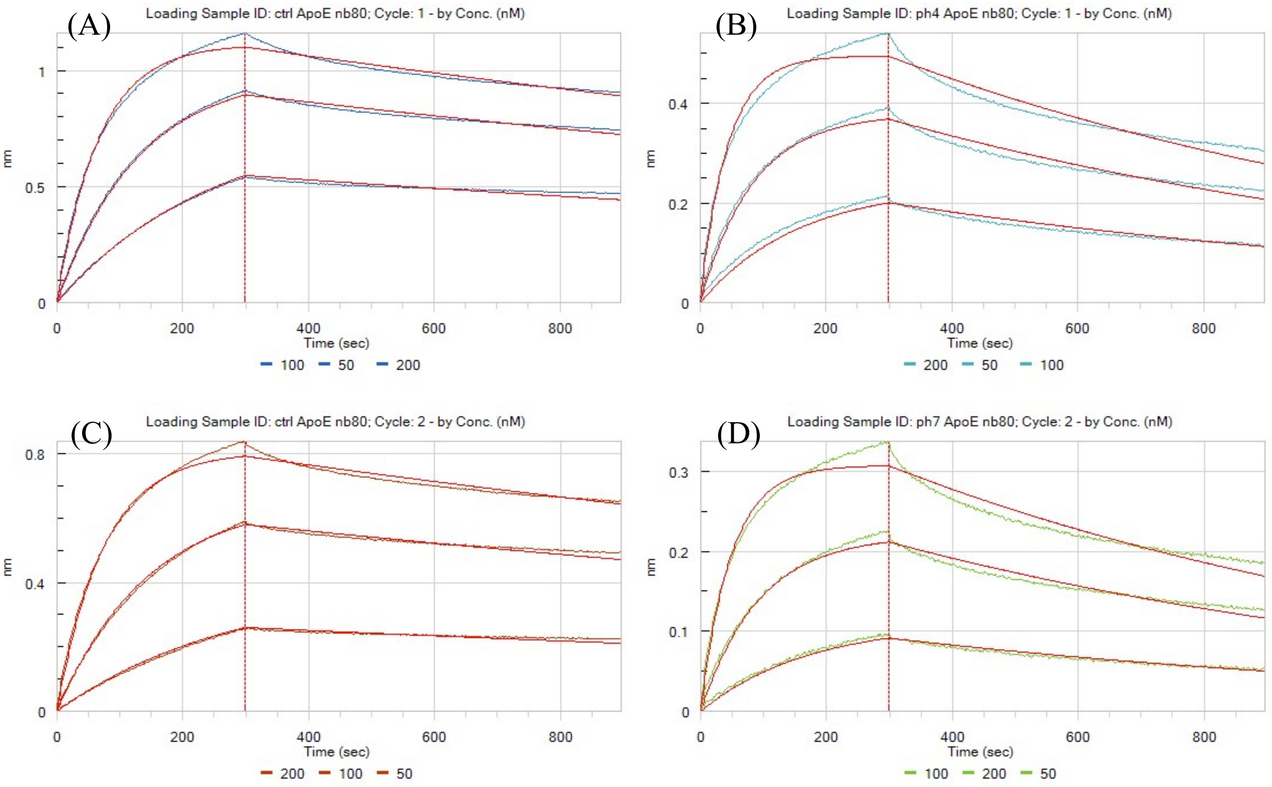

Figure S3.5.4 Association and dissociation curves for the interaction of the apoE4 Nb (labelled at pH 4 (B) or pH 7 (D)) and control (not labelled nanobody (A and C)) with ApoE4. ApoE4 Nb with a C-terminal HA-His6-tag, either labelled sample or not labelled control sample, were loaded onto biosensors coated with an anti-penta-His Ab. After measuring the background in the buffer, the tips were dipped into buffer solution containing apoE4 at various concentrations (association) and subsequently into a well containing only buffer for dissociation. The binding and release of apoE4 produced a shift in the interference pattern of reflected light, which was measured in real-time, resulting in the shown binding curves.

4. Small molecule Trp-TAD conjugation and NMR structural analyses of the obtained adducts

4.1 Boc-Trp-OH

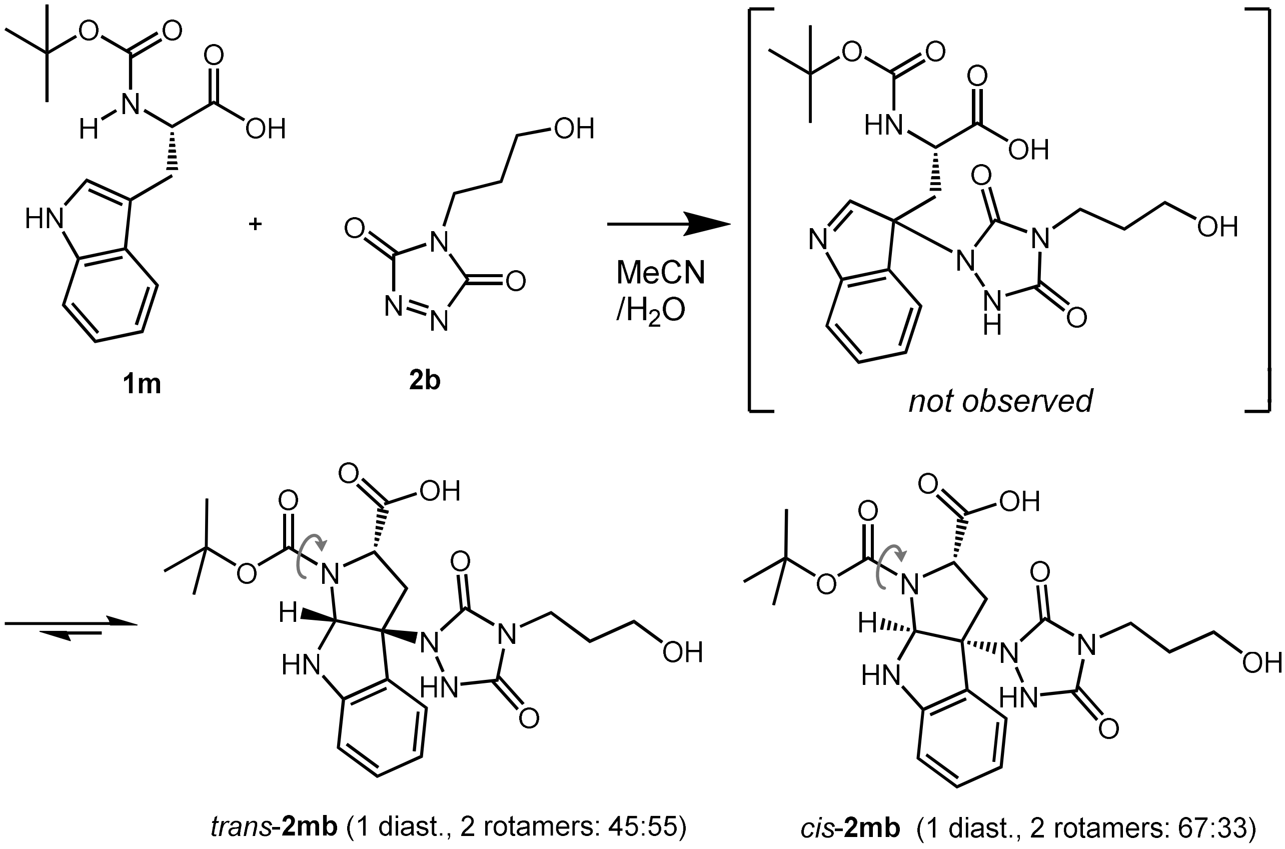

Figure 4.1.1 Structural representation of Boc-Trp-OH **1m** reaction with propanol TAD **2b**.

Compound **2b** was prepared similarly as described in S1.2. 21.8 mg of trichloroisocyanuric acid (TCCA) (0.094 mmol, 0.33 eq.) was added to 5 mL MeCN together with 49 mg propanol urazole (0.31 mmol, 1.1 eq.) and the reaction was stirred for 2 hours at room temperature. Afterwards the reaction mixture was centrifuged to precipitate any compounds insoluble in MeCN and **2b** was used directly from the supernatant. Then, 85.6 mg of Boc-Trp-OH **1m** (0.28 mmol) was dissolved in 5mL H_2O/MeCN (1:1, 0.056 M). Next, the freshly prepared bright purple solution of **2b** was added with a glass pipet in 5 portions of 1mL. The purple color of **2b** disappeared over several seconds following the addition of each portion to **1m.** LCMS analysis of the reaction of **2b** with **1m** in H_2O/MeCN showed two separate product peaks of equal intensity, both corresponding to the molecular weight of the expected adduct of **1m** and **2b**. Both peaks were well separated and analytical samples (~5 mg) were collected using a semiprep HPLC instrument equipped with a Phenomenex Luna C18 column at 35 °C with a flow rate of 16 mL/min. The column was eluted with a gradient starting at 100% H_2O containing 0.1% TFA to 100% acetonitrile in 20 min. Both samples were lyophilized and resolubilized in DMSO-d6.

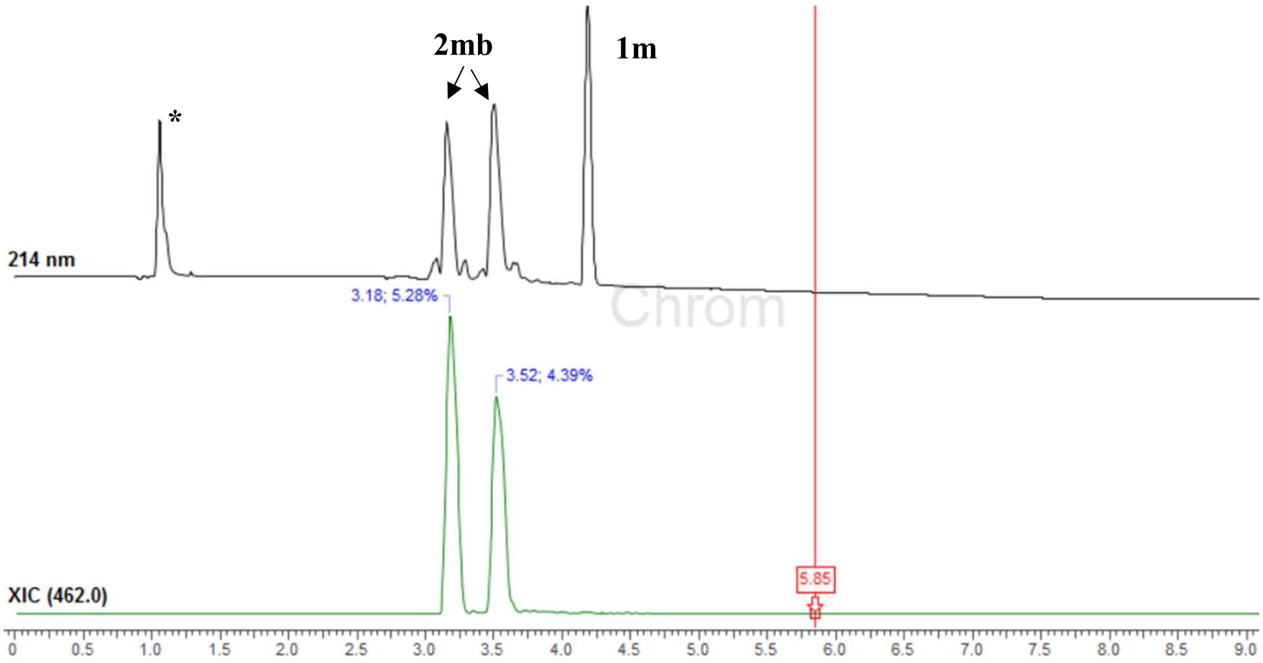

Figure 4.1.2 LC-chromatogram at 214 nm (top) and extracted ion chromatogram (XIC) for the **2mb** ion (462 Da) (bottom). The peak eluting at around t_R = 1.1 min (marked with "*") is not peptide related.

Detailed analysis of 1D proton and 2D proton carbon heteronuclear NMR measurements indicated that the first eluting fraction corresponds to a single diastereomer of the annulated adduct **2mb**, observed as 2 N-Boc-rotamers. The carboxylic acid and the urazole substituent on the pyrrolidine ring are oriented *trans* with respect to each other, while the indoline/pyrrolidine ring fusion is expected to be a *cis*-ring fusion. Similarly, the second collected fraction is most readily interpreted as two N-Boc-rotamers of the other diastereoisomer of the same adduct **2mb**, where the carboxylic acid and the urazole functions are oriented in a *cis*-fashion on the newly formed pyrrolidine ring. This formation of the additional pyrrolidine ring structure from the expected 3H-indole initial adduct via a spontaneous imine/aminal conversion was previously reported in reactions involving TAD-indoles,[3] and also in organoradical type conjugations on tryptophans.[11] This equilibrium may be solvent dependent.

The isolated fraction assigned the ***trans*-2mb** diastereomer formed consisted of a 45:55 mixture of Boc rotamers, as determined by integration of the tert-butyl protons which were the most well resolved signals in the 1D NMR spectrum. The listed carbon NMR data was only derived from observed cross-peaks in 2D HSQC and HMBC spectra, as the 1D carbon NMR spectra proved too complex due to scarcity of sample and the presence of rotamers:

^{1}H-NMR (400 MHz, DMSO-d$_6$): δ = 1.39 & 1.49 (s, 9H (55:45), OC(C*H*$_3$)$_3$), 1.59 (2H, quint(br), *J* = ~6.8 Hz, CH$_2$-C*H*$_2$-CH$_2$-OH), 2.88 & 2.91 (1H (55:45), d(br), *J* = 13.4 Hz, C*H*H-CH-CO$_2$H), 3.10 & 3.16 (1H (45:55), dd, *J* = 13.4, 9.5 Hz, CH*H*-CH-CO$_2$H), 3.32 (2H, t, *J* = 6.4 Hz, CH$_2$-C*H*$_2$-OH), 3.38 (2H, t, *J* = 7.2 Hz, N-C*H*$_2$-CH$_2$), 4.49 & 4.50 (1H (45:55), d(br), J = 9.5 Hz, C*H*-COOH), 5.72 & 5.74 (1H (45:55), s, NH-C*H*-N), 6.48 – 6.57 (band, 2H, 2 x Ar-*H*), 6.99-7.04 (band, 2H, 2 x Ar-*H*), 10.16 & 10.21 (1H(55:45), s(br), urazole-N*H*) ppm. Very broad signals are visible in the 5-6 and 11-13 ppm range, most likely belonging to OH's and NH's, integrating for about 3H in total.

^{13}C-NMR (100 MHz, DMSO-d_6, HSQC-HMBC): δ = 27.8 (C(*C*H_3)$_3$), 30.4 (CH_2*C*$H_2$$CH_2$), 35.9 (N-*C*$H_2$$CH_2$), 38.6 (*C*$H_2CHCO_2$H), 57.7 (*C*$H_2$OH), 58.4 (*C*H$CO_2$H), 79.0 (NH-*C*H-N), 79.4/79.7 (O*C*(CH_3)$_3$), 108.8 (=*C*H), 116.8 (=*C*H), 122.9 (=*C*H), 124.9 (C), 129.8 (=*C*H), 151.0 (C), 154.8 (*C*O), 155.8 (*C*O), 171.5 (*C*O_2H) ppm. The carbon corresponding to the carbamoyl group cannot be observed in the obtained spectra. The relative stereochemistry can be assigned based on the absence of a large scalar coupling between two vicinal protons in the pyrrolidine ring (only possible for a dihedral angle around 0°).

The isolated fraction assigned the ***cis*-2mb** diastereomer structure consisted of a 67:33 mixture of Boc rotamers, as determined by integration of the tert-butyl protons which were the most well resolved signals in the 1D NMR spectrum. The listed carbon NMR data was only derived from observed cross-peaks in 2D HSQC and HMBC spectra, as the 1D carbon NMR spectra proved too complex due to scarcity of sample and the presence of rotamers:

^{1}H-NMR (400 MHz, DMSO-d_6): 1.36 & 1.47 (s, 9H (67:33), OC(C*H*$_3$)$_3$), 1.57 (2H, quint(br), *J* = ~6.6 Hz, CH_2-C*H*$_2$-CH_2-OH), 2.84 (33% of 1H, d(AB)d(br), *J* = 13.3(AB), 7.2 Hz, C*H*H-CH-CO_2H), 2.95 (67% of 1H, d(AB)d(br), *J* = 13.8(AB), 7.8 Hz, C*H*H-CH-CO_2H), 3.00 (33% of 1H, d(AB)d, *J* = 13.3(AB), 7.5 Hz, CH*H*-CH-CO_2H)3,02 (67% of 1H, d(AB)d(br), *J* = 13.8(AB), 7.6 Hz, CH*H*-CH-CO_2H), 3.31 (2H, t, *J* = 6.1 Hz, CH_2-C*H*$_2$-OH), 3.37 (2H, t, *J* = 7.3 Hz, N-C*H*$_2$-CH_2), 3.84 & 3.93 (1H (67:33), t, J = ~7.7 Hz, C*H*-COOH), 5.81 & 5.83 (1H (33:67), s, NH-C*H*-N), 6.57-6.64 (band, 2H, 2 x Ar-*H*), 7.05-7.12 (band, 2H, 2 x Ar-*H*), 10.21 & 10.29 (1H(33:67), s(br), urazole-N*H*) ppm. Very broad signals are visible in the 5-6 and 11-13 ppm range, most likely belonging to OH's and NH's, integrating for about 3H in total.

^{13}C-NMR (100 MHz, DMSO-d_6, HSQC-HMBC): δ = 27.4/27.6 (C(*C*H_3)$_3$), 30.2 (CH_2*C*$H_2$$CH_2$), 35.8 (N-*C*$H_2$$CH_2$), 39.0 (*C*$H_2CHCO_2$H), 57.7 (*C*$H_2$OH), 58.3/58.6 (*C*H$CO_2$H), 79.1/79.3 (NH-*C*H-N), 79.4/79.7 (O*C*(CH_3)$_3$), 109.0 (=*C*H), 117.4 (=*C*H), 122.7 (=*C*H), 125.3 (C), 130.0 (=*C*H), 150.1 (C), 154.7 (*C*O), 155.7 (*C*O), 172.9 (*C*O_2H) ppm. The carbon corresponding to the carbamoyl group cannot be observed in the obtained spectra. The relative stereochemistry can be assigned based on two >7 Hz scalar couplings to the methine proton in carboxyl-substituted position of the pyrrolidine ring.

4.2 N-Acyl-Trp-OMe

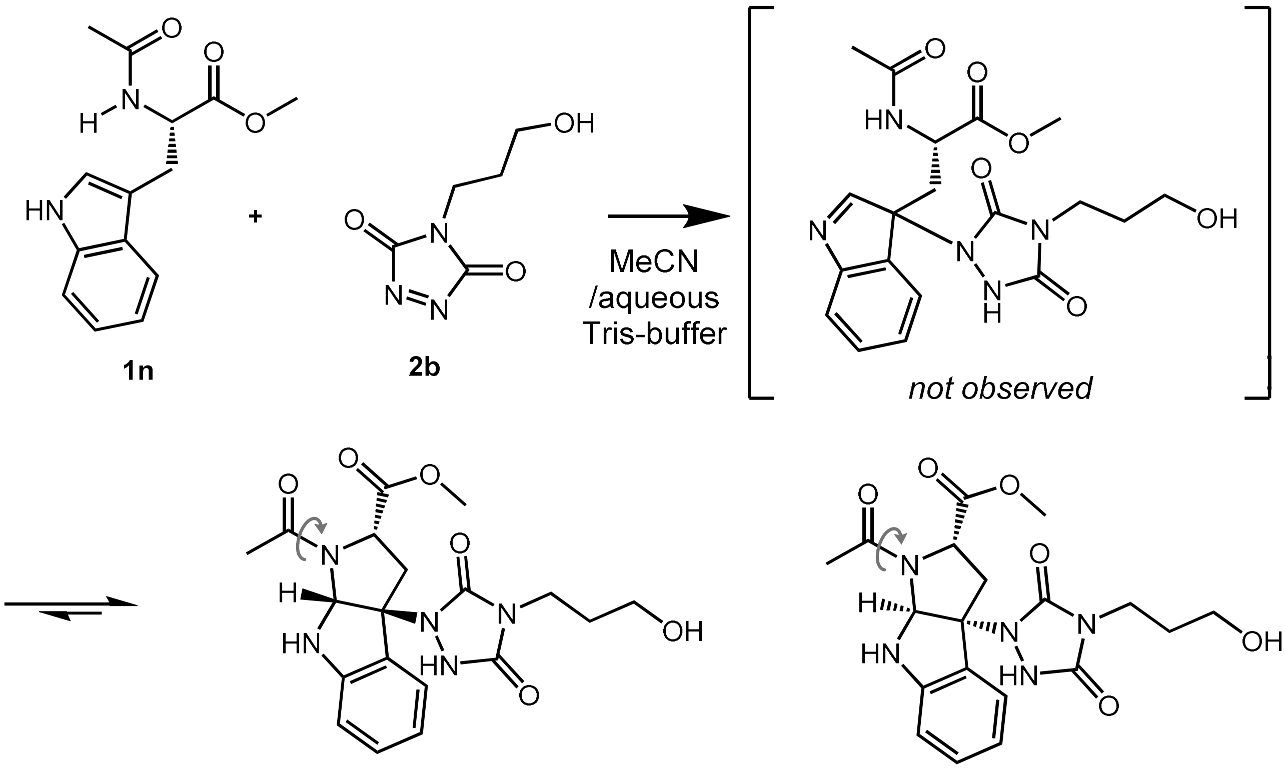

1n **2b**

trans-**2nb** (1 diast./2 rotamers: ~70:30) *not isolated*

Figure 4.2.1 Structural representation of N-Acyl-Trp-OMe **1n** reaction with propanol TAD **2b**.

Compound **2b** was prepared similarly as described in S1.2. 21.8 mg of trichloroisocyanuric acid (TCCA) (0.094 mmol, 0.33 eq.) was added to 5 mL MeCN together with 49 mg propanol urazole (0.31 mmol, 1.1 eq.) and the reaction was stirred for 2 hours at room temperature. Afterwards the reaction mixture was centrifuged to precipitate any compounds insoluble in MeCN and **2b** was used directly from the supernatant. 82 mg of N-Acyl-Trp-OMe **1n** (0.31 mmol, 0.052 M) was dissolved in 6 mL Tris/MeCN (1:1). **2b** was added with a glass pipet in portions of 1mL (5 x). The bright purple color of **2b** disappeared after several seconds upon the addition of each portion of **2b** to **1n.** LCMS analysis of the reaction of **1n** with **2b** in MeCN demonstrated a single broad product peak corresponding to the molecular weight of the adduct pf **1n** and **2b.** The reaction mixture was purified using a semiprep HPLC instrument equipped with a Phenomenex Luna C18 column at 35 °C with a flow rate of 16 mL/min. The column was eluted with a gradient starting at 100% H_2O containing 0.1% TFA to 100% acetonitrile in 20 min. HRMS (ESI): m/z calculated for **trans-2nb** $C_{19}H_{23}N_5O_6$ $[M+H]^+$ 418.1721, found 4181731 (figure 4.2.2).

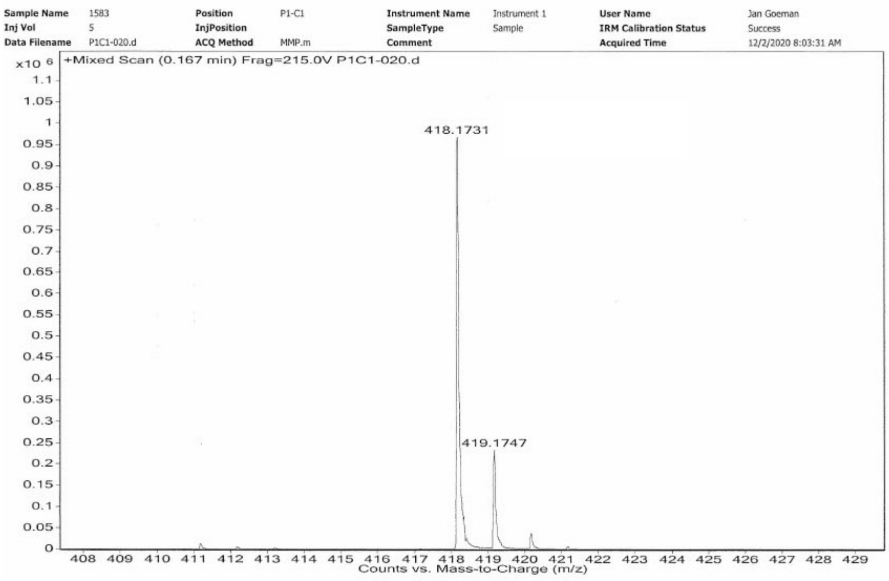

Figure 4.2.2. HRMS (ESI) analysis: m/z calculated for **trans-2nb** $C_{19}H_{23}N_5O_6$ $[M+H]^+$ 418.1721, found 418.1731.

The collected fraction was lyophilized and resolubilized in acetonitrile-d3:D_2O (1:1). Similar to the previous characterisation of **2mb**, detailed analysis of 1D proton and carbon, and 2D proton carbon heteronuclear NMR measurements, could be most readily interpreted as corresponding to the annulated adduct **2nb**, but stereochemical interpretation was hampered here by the fact that the two stereoisomers were not seperable, and integration and assignments are difficult due to the possible N-acetamide rotamers or possible other isomers. The overall similarity of the major resonances (integrating for 60:25:15) allow the tentative assignment of two rotamers for the trans-diastereomer as the major product (indoline/pyrrolidine ring fused product with a trans-relation: ***trans*-2nb**). The cis-diastereomer is not observed, but several minor peaks occur obscuring a full analysis. One possible explanation for this result is a fast, dynamic equilibrium of aminal, hemiaminal and imine products for the cis-isomer, explaining the presence of several minor peaks in the spectrum. This could not be confirmed, and the compounds showed limited solubility in non-aqueous media solvents, further hampering analysis. It seems likely that the TAD-conjugation does not proceed stereoselectively here, but rather that it is difficult to isolate one of the two possible diastereoisomers.

The purified analytical sample (5-10 mg) was assigned the overall ***trans*-2nb** structure, with a rotamer ratio of 70:30 of the N-acetamide, as determined by integration of the well resolved resonances in the 1D NMR spectrum. The listed NMR data is only that of the major rotamer, as analysis of the minor rotamer proved too complex due to overlap with the other minor isomers or possible hydrolysis product:

^{1}H-NMR (400 MHz, CD_3CN:D_2O 1:1): δ = 1.67 (2H, quint(br), *J* = ~6.4 Hz, CH_2-C*H*$_2$-CH_2-OH), 1.96 (3H, s, COC*H*$_3$), 3.17 (1H, dd(br), *J* = 13.0, 8.4 Hz, C*H*H-CH-CO_2D), 3.19 (3H, s, OC*H*$_3$) 3.29 (1H, d(br), *J* = 13.0 Hz, CH*H*-CH-CO_2Me), 3.28-3.34 (2H, m, CH_2-C*H*$_2$-OD), 3.39 (2H, t, *J* = 7.2 Hz, N-C*H*$_2$-CH_2), 4.73 (1H, d(br), J = 8.4 Hz, C*H*-CO_2Me), 5.76 (1H, s, NH-C*H*-N), 6.60 – 6.70 (band, 2H, 2 x Ar-*H*), 7.11-7.20 (band, 2H, 2 x Ar-*H*). The urazole and other OH and NH are not visible due to deuterium exchange with D_2O.

^{13}C-NMR (100 MHz, DMSO-d_6, HSQC-HMBC): δ = 21.6 (CO*C*H_3), 31.2 (CH_2*C*H_2CH_2), 36.6 (N-*C*H_2CH_2), 38.8 (*C*H_2CHCO_2H), 52.5 (O*C*H_3) 59.1 (*C*H_2OD), 61.0 (*C*HCO_2D), 79.6 (NH-*C*H-N), 109.0 (=*C*H), 117.4 (=*C*H), 122.7 (=*C*H), 123.0 (=*C*H), 135.2 (urazole *C*O), 136.6 (urazole *C*O), 150.5 (C), 150.9 (CH_3*C*O) ppm. One quaternary aromatic carbon cannot be assigned.

5. NMR spectra of compounds

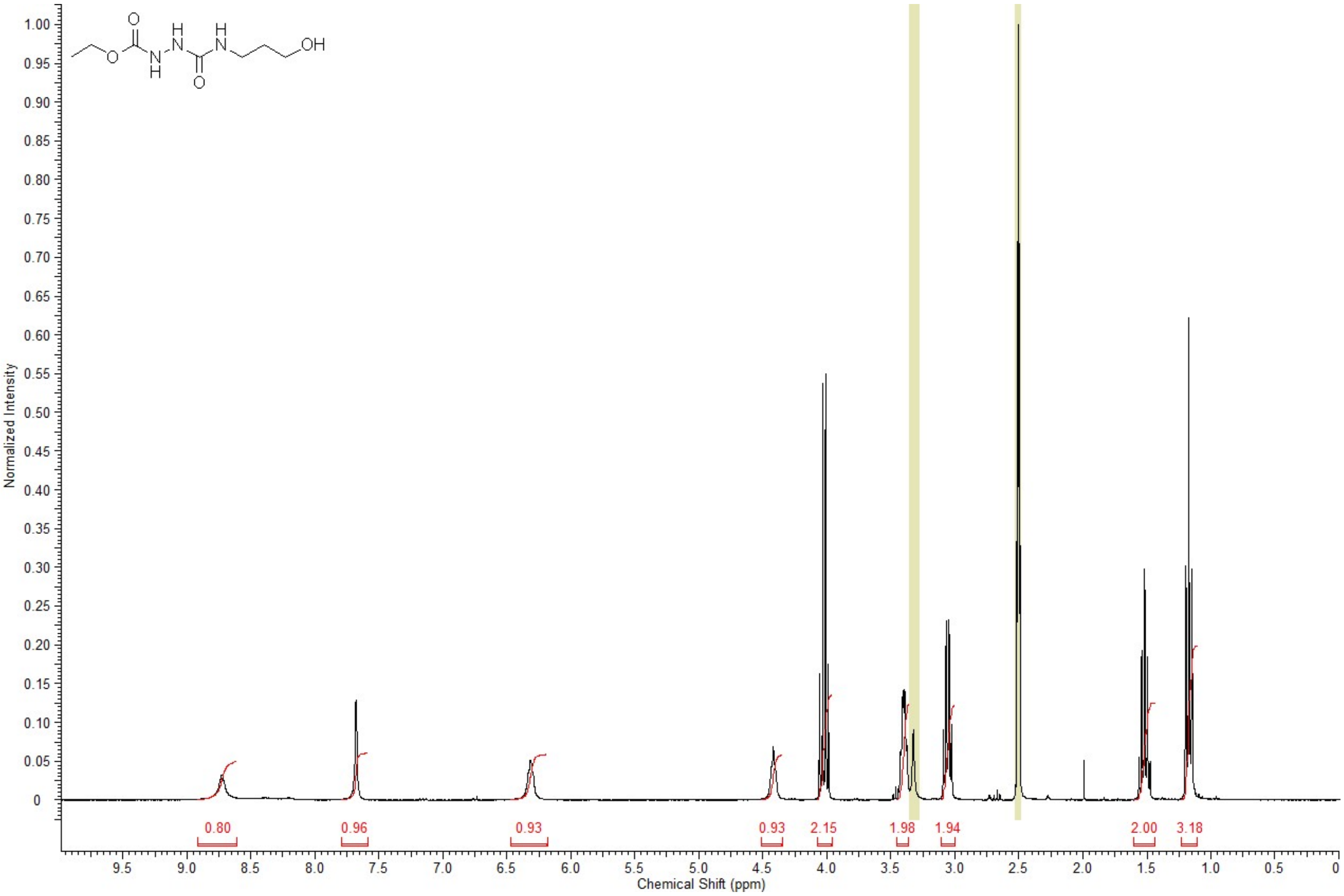

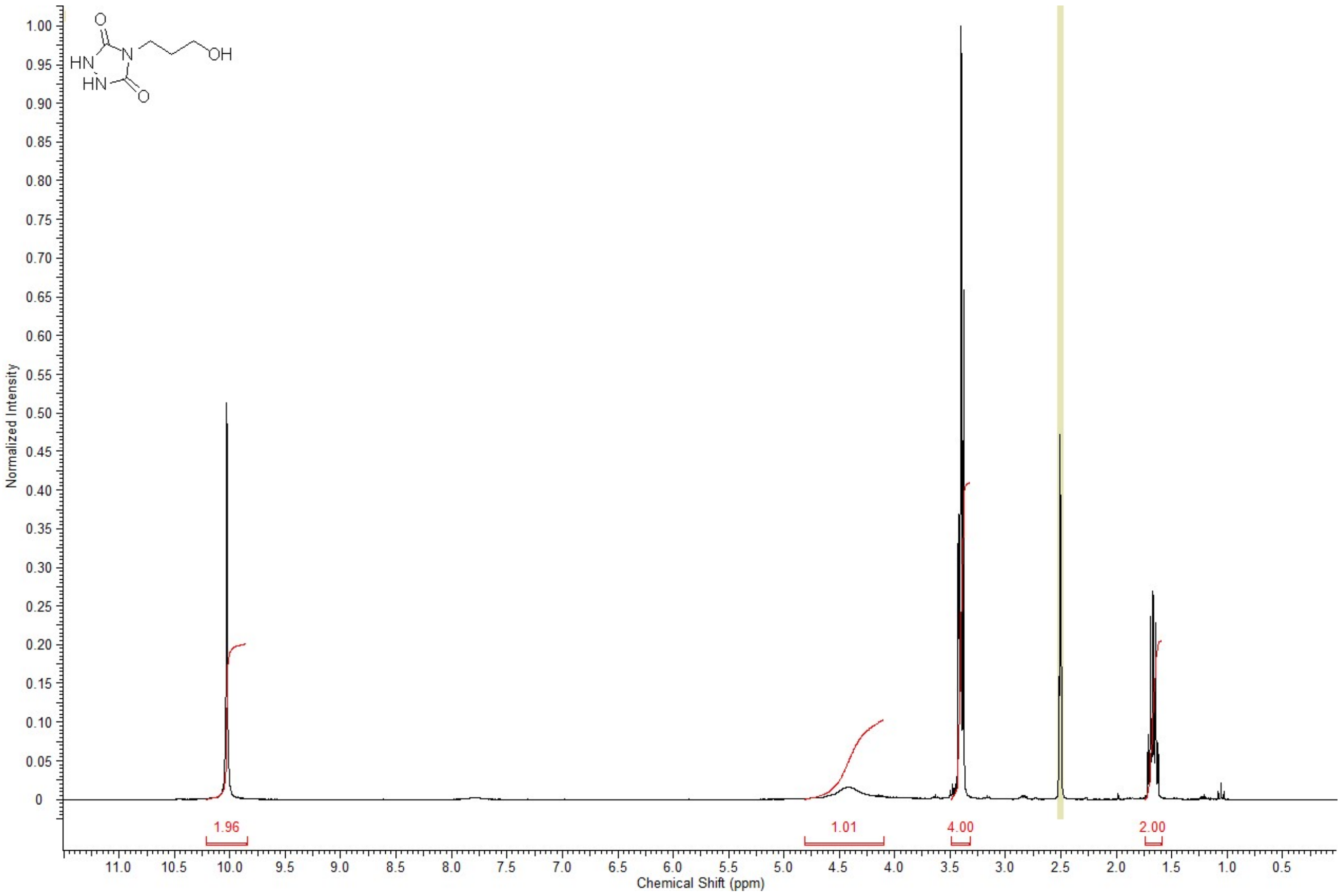

HN
HN
N
O
O
OH
Normalized Intensity
Chemical Shift (ppm)
1.96
1.01
4.00
2.00

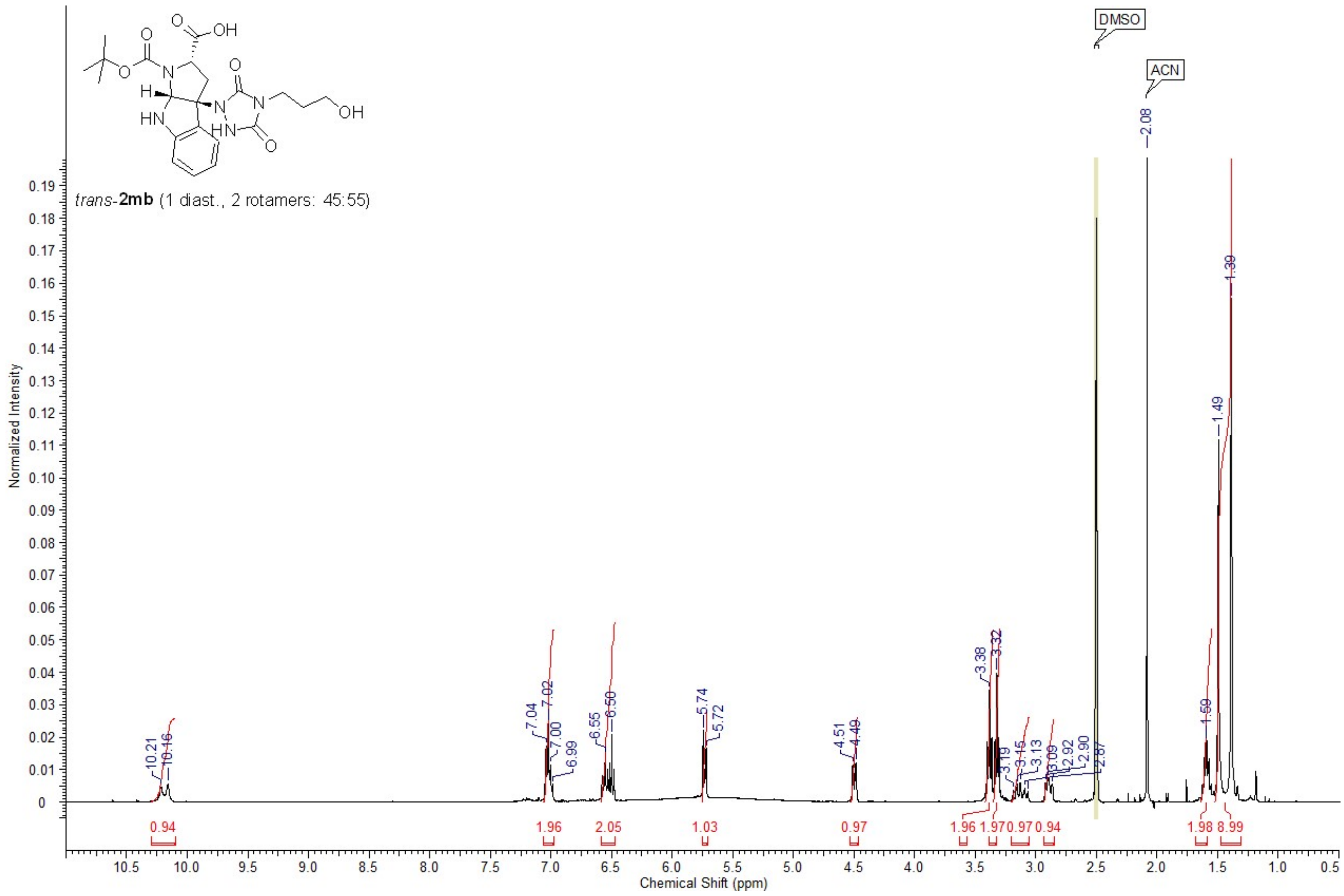

DMSO
ACN
trans-2mb (1 diast., 2 rotamers: 45:55)
Normalized Intensity
Chemical Shift (ppm)

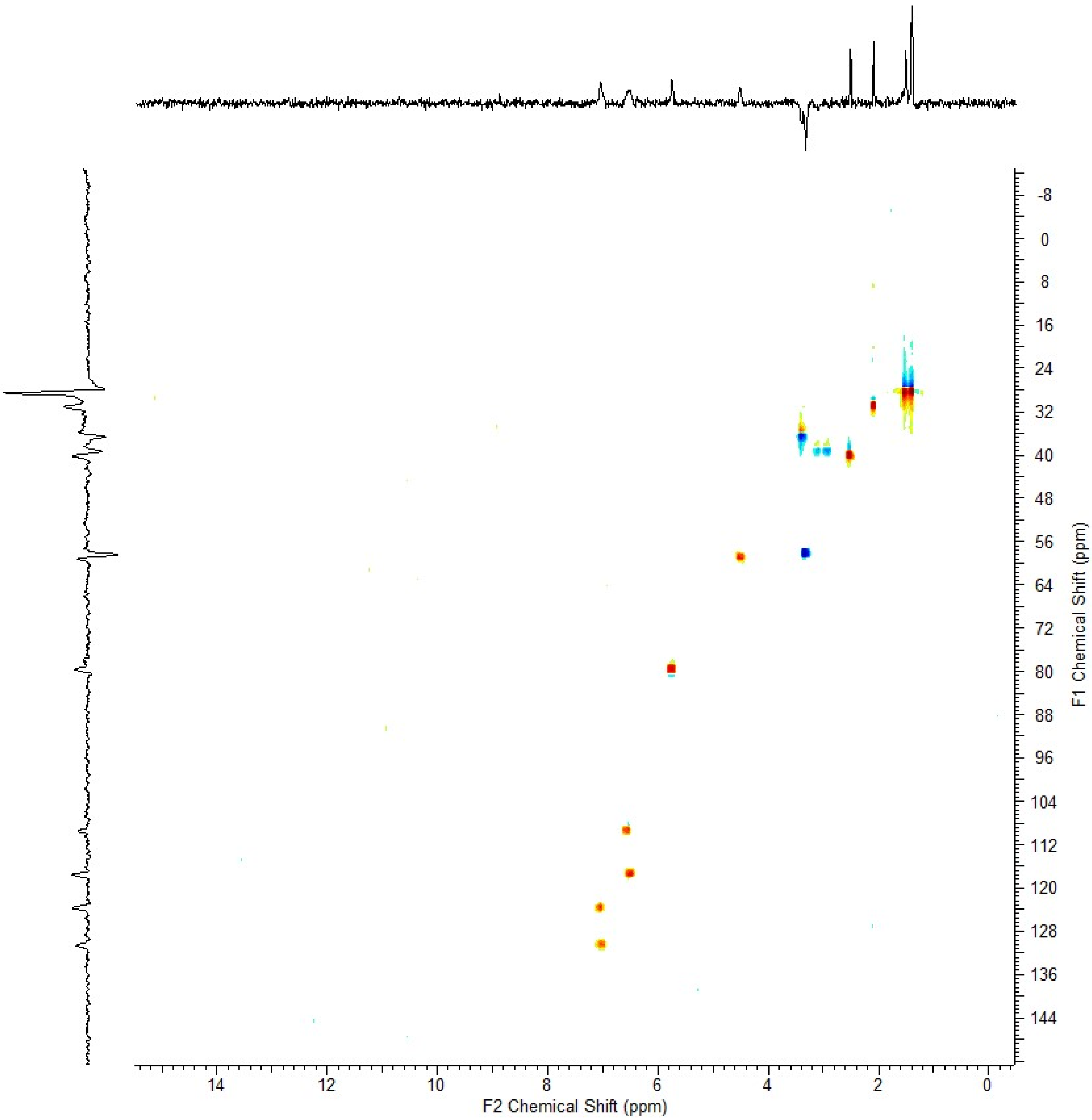

-8
0
8
16
24
32
40
48
56
64
72
80
88
96
104
112
120
128
136
144
F1 Chemical Shift (ppm)
14
12
10
8
6
4
2
0
F2 Chemical Shift (ppm)

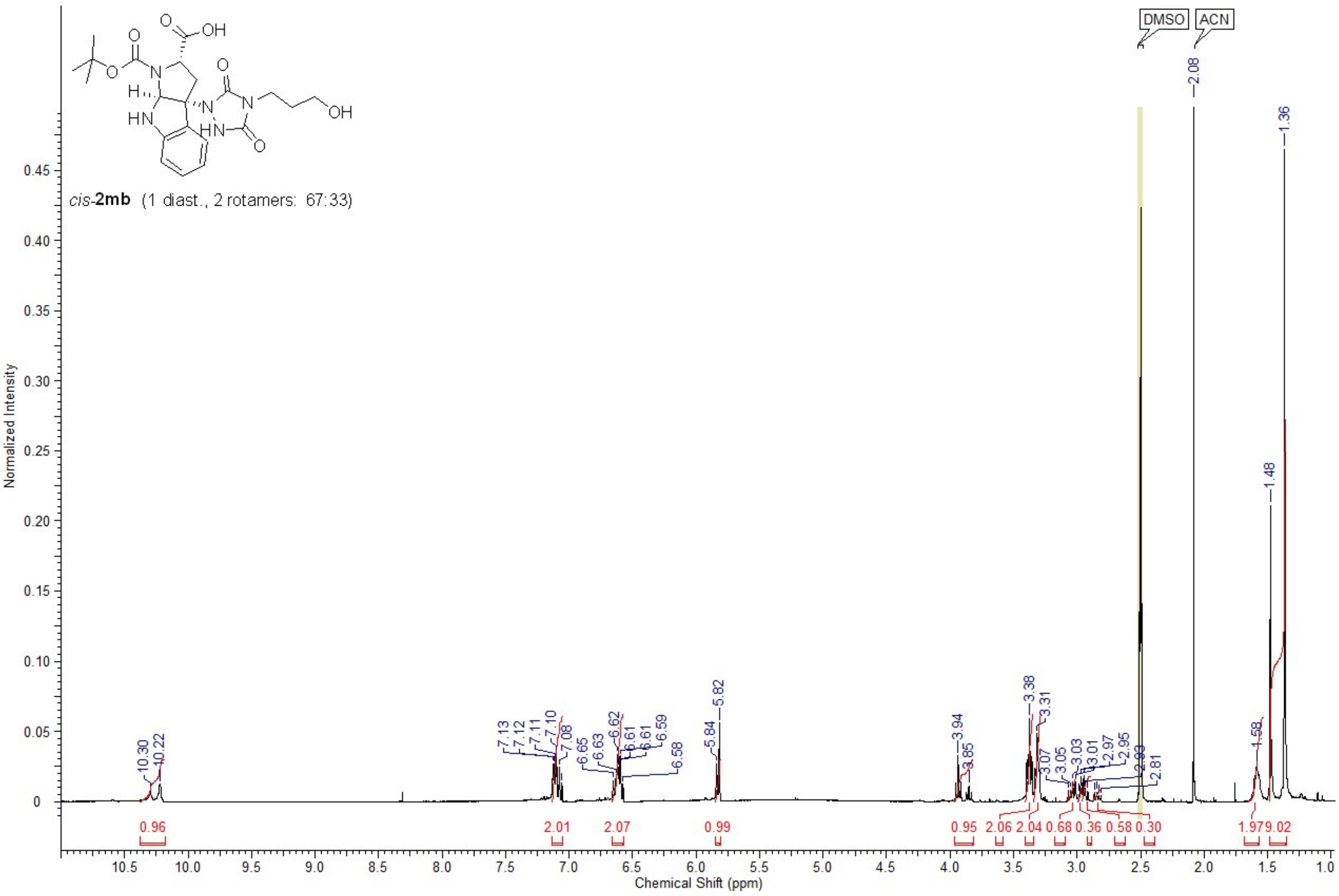
cis-2mb (1 diast., 2 rotamers: 67:33)
DMSO
ACN
Normalized Intensity
Chemical Shift (ppm)

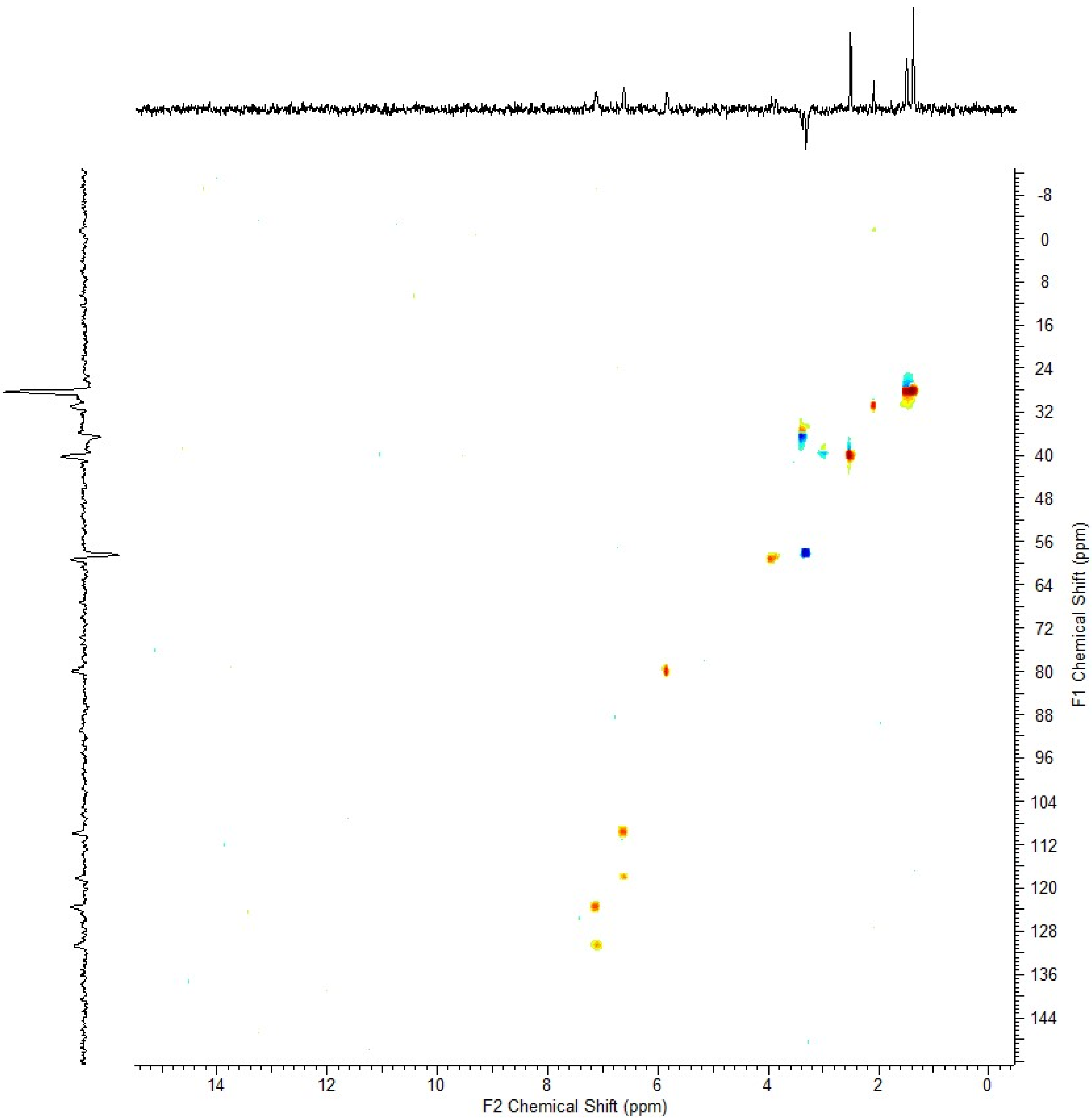
F2 Chemical Shift (ppm)
F1 Chemical Shift (ppm)

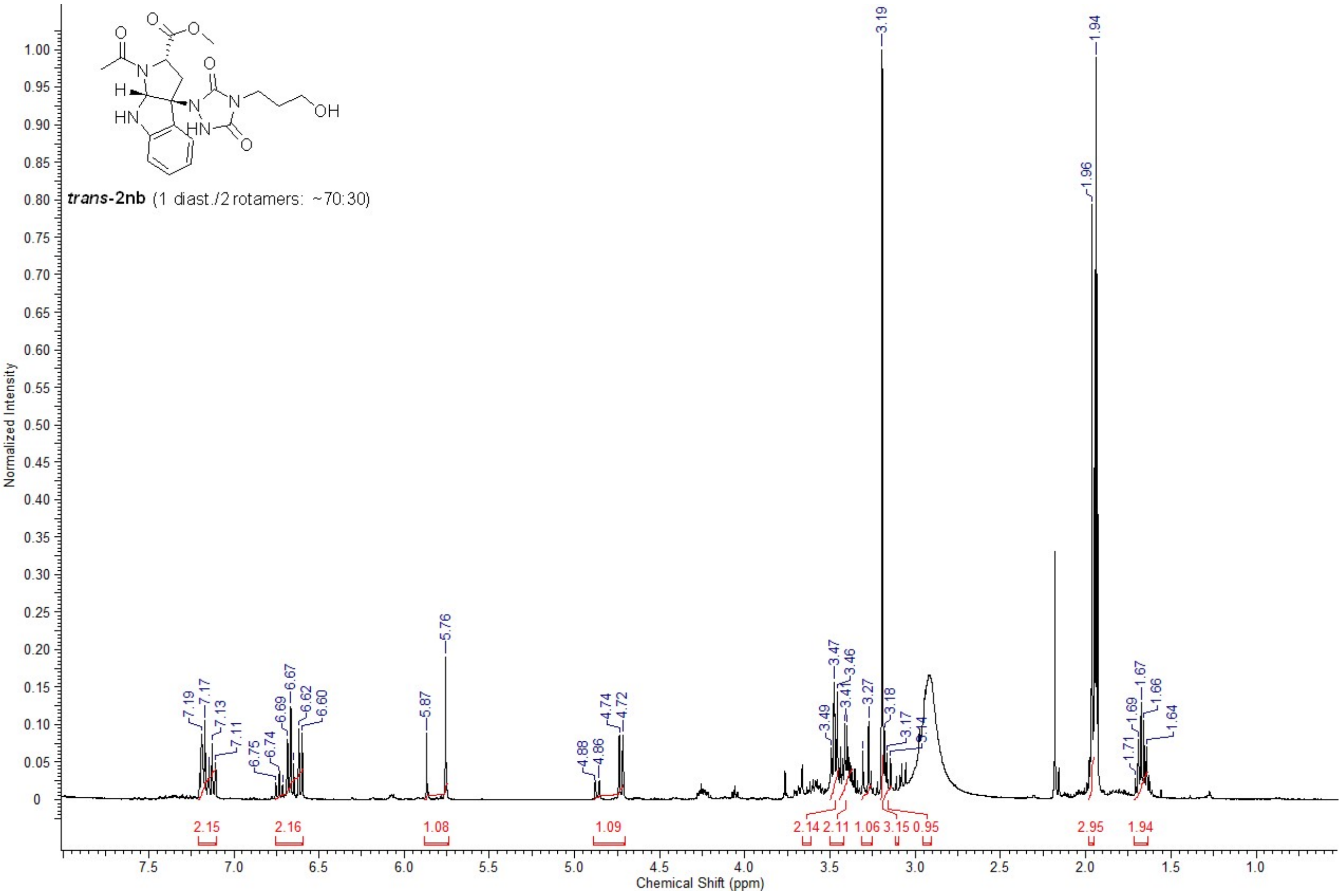
trans-2nb (1 diast./2 rotamers: ~70:30)
Normalized Intensity
Chemical Shift (ppm)
7.19 7.17 7.13 7.11 6.75 6.74 6.69 6.67 6.62 6.60 5.87 5.76 4.88 4.86 4.74 4.72 3.49 3.47 3.46 3.41 3.27 3.19 3.18 3.17 3.14 1.96 1.94 1.71 1.69 1.67 1.66 1.64
2.15 2.16 1.08 1.09 2.14 2.11 1.06 3.15 0.95 2.95 1.94

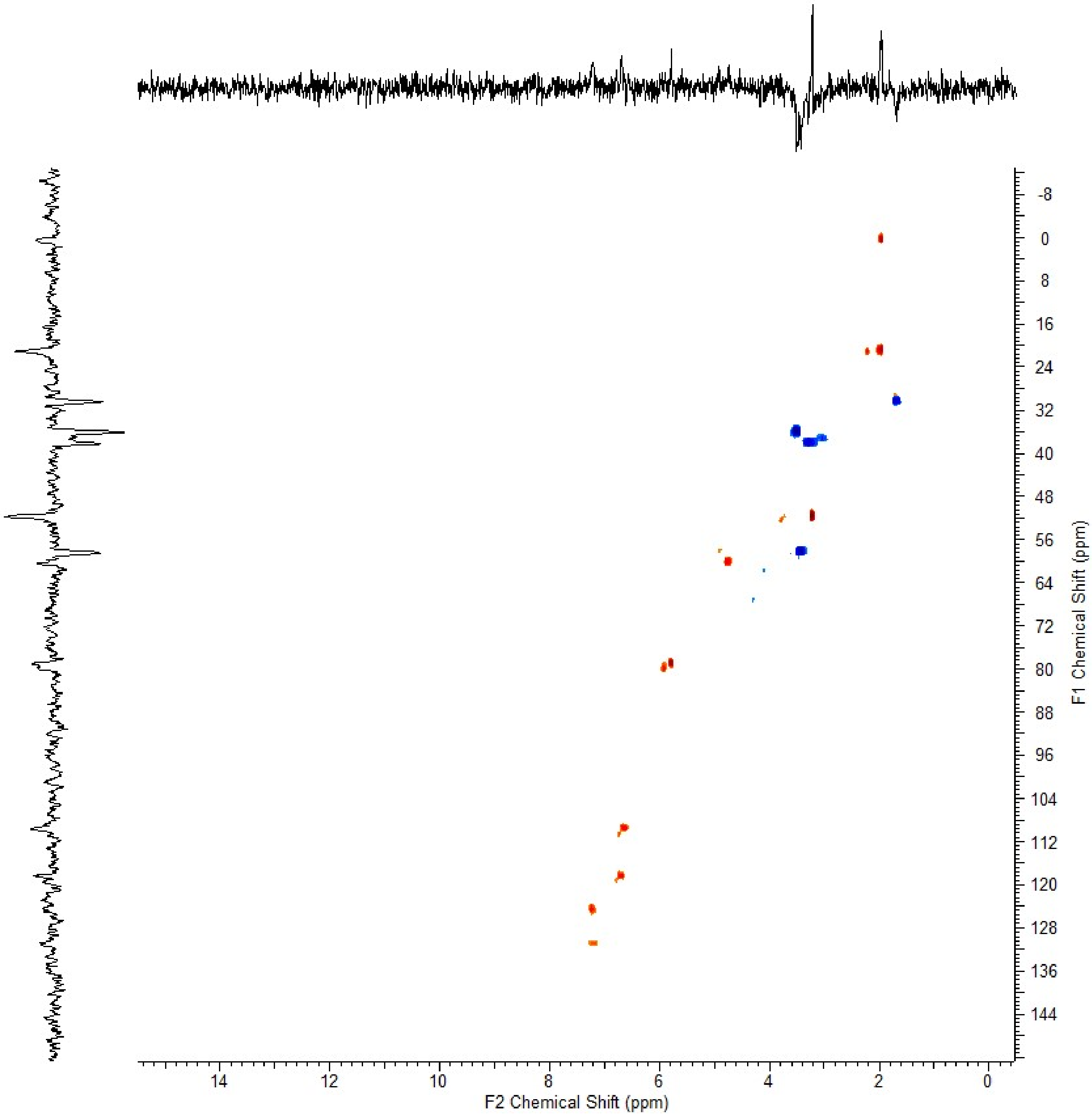

F2 Chemical Shift (ppm)
F1 Chemical Shift (ppm)
14
12
10
8
6
4
2
0
-8
0
8
16
24
32
40
48
56
64
72
80
88
96
104
112
120
128
136
144

6. References

1. Baroni A, Vlaminck L, Mespouille L, Prez F Du, Delbosc N, Blankert B. TAD Click Chemistry on Aliphatic Polycarbonates : A First Step Toward Tailor-Made Materials. 2019;1800743:1-5. doi:10.1002/marc.201800743

2. Kaiser D, Winne JM, Ortiz-Soto ME, Seibel J, Le TA, Engels B. Mechanistical Insights into the Bioconjugation Reaction of Triazolinediones with Tyrosine. *J Org Chem*. 2018;83(17):10248-10260. doi:10.1021/acs.joc.8b01445

3. Baran PS, Guerrero CA, Corey EJ. The First Method for Protection−Deprotection of the Indole 2,3-π Bond. *Org Lett*. 2003;5(11):1999-2001. doi:10.1021/ol034634x

4. Bakken V, Helgaker T, Uggerud E. Models of fragmentations induced by electron attachment to protonated peptides. *Eur J Mass Spectrom*. 2004;10(5):625-638. doi:10.1255/ejms.665

5. Helsens K, Colaert N, Barsnes H, et al. ms _ lims , a simple yet powerful open source laboratory information management system for MS-driven proteomics. Published online 2010:1261-1264. doi:10.1002/pmic.200900409

6. Vandewalle S, De Coen R, De Geest BG, Du Prez FE. Tyrosine-Triazolinedione Bioconjugation as Site-Selective Protein Modification Starting from RAFT-Derived Polymers. *ACS Macro Lett*. Published online 2017:1368-1372. doi:10.1021/acsmacrolett.7b00795

7. Ban H, Gavrilyuk J, Barbas CF. Tyrosine bioconjugation through aqueous ene-type reactions: A click-like reaction for tyrosine. *J Am Chem Soc*. 2010;132(5):1523-1525. doi:10.1021/ja909062q

8. Desmet J, Verstraete K, Bloch Y, et al. Structural basis of IL-23 antagonism by an Alphabody protein scaffold. *Nat Commun*. 2014;5(1):5237. doi:10.1038/ncomms6237

9. Saussez S, Kiss R. Galectin-7. *Cell Mol Life Sci*. 2006;63(6):686-697. doi:10.1007/s00018-005-5458-8

10. Hu QY, Allan M, Adamo R, et al. Synthesis of a well-defined glycoconjugate vaccine by a tyrosine-selective conjugation strategy. *Chem Sci*. 2013;4(10):3827-3832. doi:10.1039/c3sc51694f

11. Seki Y, Ishiyama T, Sasaki D, et al. Transition Metal-Free Tryptophan-Selective Bioconjugation of Proteins. *J Am Chem Soc*. 2016;138(34):10798-10801. doi:10.1021/jacs.6b06692
